# Global Reaction Route Mapping of C_3_H_2_O: Isomerization Pathways, Dissociation Channels, and Bimolecular Reaction with a Water Molecule

**DOI:** 10.3390/molecules30081829

**Published:** 2025-04-18

**Authors:** Dapeng Zhang, Naoki Kishimoto

**Affiliations:** Department of Chemistry, Graduate School of Science, Tohoku University, 6-3, Aoba, Aramaki, Aoba-ku, Sendai 980-8578, Japan; zhang.dapeng.c5@tohoku.ac.jp

**Keywords:** C_3_H_2_O, isomerization, conformational explorations, dissociation pathways, bimolecular reactions

## Abstract

A comprehensive theoretical investigation of the C_3_H_2_O potential energy surface (PES) was conducted, revealing 30 equilibrium structures (EQs), 128 transition state structures (TSs), and 35 direct dissociation channels (DCs), establishing a global reaction network comprising 101 isomerization pathways and dissociation channels. Particular focus was placed on the five most stable isomers, H_2_CCCO (EQ3), OC(H)CCH (EQ7), H-*c*-CC(O)C-H (EQ0), HCC(H)CO (EQ1), and HO-*c*-CCC-H (EQ12), and their reactions with water molecules. Multicomponent artificial force-induced reaction (MC-AFIR) calculations were employed to study bimolecular collisions between H_2_O and these stable isomers. The product distributions revealed isomer-specific reactivity patterns: EQ3 and EQ7 predominantly formed neutral species at high collision energies, EQ0 produced both ionic and neutral species, while EQ1 and EQ12 exhibited more accessible reaction pathways at lower collision energies with a propensity for spontaneous isomerization. Born–Oppenheimer Molecular Dynamics (BOMD) simulations complemented these findings, suggesting several viable products emerge from reactions with water molecules, including HCCC(OH)_2_H (EQ7 + H_2_O), OCCHCH_2_OH (EQ1 + H_2_O), and HO-*c*-CC(H)C(OH)-H (EQ12 + H_2_O). This investigation elucidates the intrinsic relationships between isomers and their potential products, formed through biomolecular collisions with water molecules, establishing a fundamental framework for future conformational and reactivity studies of the C_3_H_2_O family.

## 1. Introduction

The interstellar medium (ISM) contains a diverse array of molecules, demonstrating an evolutionary progression from simple to complex organic and prebiotic compounds [1]. With the growing diversity of interstellar molecules detected in ISM [2,3], extensive experimental and theoretical investigations have been conducted to elucidate potential molecular species, as well as their formation and dissociation mechanisms, chemical reactivity, and evolutionary processes [4,5]. The exploration of molecular complexity in chemical transformations remains a fundamental pursuit in both astrochemistry and physical chemistry. The formation of interstellar molecules typically proceeds through gas–grain reactions on cosmic dust surfaces (involving radiation-induced processes, photochemical activation, and thermal effects) and gas-phase reactions, playing crucial roles, as evidenced by the detection of numerous species in interstellar environments [6,7]. The investigation of molecular formation and reactivity represents a crucial area in theoretical and computational chemistry, with conformational analysis serving as a fundamental tool for understanding molecular structures and transformations. A comprehensive examination of minima, saddle points, and the connections on the potential energy surface (PES) yields essential insights into conformational behavior. Specifically, the explored equilibrium structures (EQs) reveal intrinsic relationships between molecular stability and conformation, transition state structures (TSs) elucidate reaction pathways between different conformers, and dissociation channels (DCs) illuminate potential fragmentation mechanisms, providing valuable information about molecular decomposition processes [8].

Cyclopropenone, with its cyclic structure (H-*c*-CC(O)C-H), was detected by Hollis et al. in Sagittarius B2(N) using the Green Bank Telescope (GBT) [9]. Along with cyclopropenone, the C_3_H_2_O family includes propadienone (H_2_CCCO), its most stable but undetected isomer, [10] and propynal (HCCCHO), which has been detected in ISM. [11,12,13,14,15,16] Several theoretical investigations have examined the structural properties of cyclopropenone [17,18,19,20], and its formation processes, such as O_2_ + *c*-C_3_H_2_ [21] and OH + *c*-C_3_H_2_ reactions [10,22]. Despite its simple molecular formula, there is no systematical investigation of C_3_H_2_O PES that simultaneously elucidates isomerization and dissociation pathways. A comprehensive conformational analysis of cyclopropenone is crucial for understanding intrinsic relationships among its potential isomers and their reaction pathways. In this study, we performed a thorough exploration of the cyclopropenone PES using quantum chemical calculations, mapping the identified EQs with their connections to TSs and DCs.

Building upon our comprehensive exploration of the C_3_H_2_O PES, we further investigated its collision processes with water molecules, given its potential role in providing H and O atoms in molecular evolution in the ISM environment. Several studies have demonstrated the role of the water molecule as both a reactant and a catalyst in various molecular transformations, including the glycine formation from CH_2_NH, CO, and H_2_O, [23,24], the generation of methanediol, ethanol, and methanimine through reactions between ^3^C-OH_2_ (triplet state) and OH, CH_3_, and NH_2_, respectively [25], as well as the catalytic influence of H_2_O in the CH_2_CO + H reaction [26]. To extend our conformational analyses, we conducted a systematic investigation of potential products arising from cyclopropenone and water molecule collisions, and their isomer-specific reactional properties. The collision products were calculated using randomly distributed reactants, with reactions initiated through applied forces. Multiple stable isomers were examined, revealing distinct pathways of both neutral and ionic species formation. Further simulations focused on the subsequent evolution of selected collision products to evaluate their structural stability and potential transformation processes. Our findings provide comprehensive insights into product distributions and, combined with the global reaction route mapping, enhance our understanding of the cyclopropenone PES while establishing a database of structures, conformations, and their interrelationships. The diverse isomeric structures of these products give rise to varied reaction mechanisms in water-involved processes, offering valuable insights into their potential roles in atmospheric and physical chemistry.

## 2. Results and Discussion

### 2.1. Isomerization Pathways Without Dissociation (EQx-TSn-EQy)

**Figure 1:**
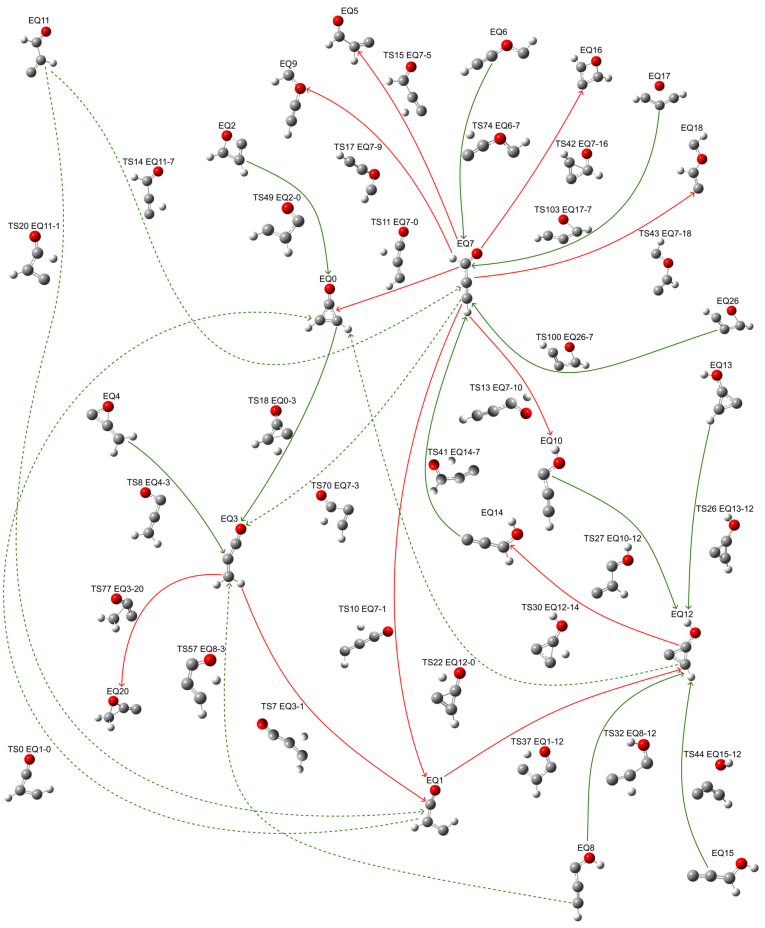
Isomerization pathways among the five most thermodynamically stable C_3_H_2_O isomers. Cyclopropenone (EQ0) served as the parent structure for conformational exploration. Isomerization processes are represented by directional arrows, with red arrows indicating transitions toward less stable isomers, and green arrows denoting transitions toward more stable configurations. Pathways of equivalent mechanistic significance are differentiated by dashed and solid lines. Atoms colored red represent oxygen, gray represent carbon, and white represent hydrogen.

Comprehensive conformational analysis revealed 30 distinct EQs and identified 128 TSs. In terms of the global reaction route map, complete atomic coordinates for all explored structures are provided in the Supplementary Materials and Zenodo repository. From these, we established 28 unique isomerization pathways, each characterized by a TS connecting a pair of EQs. Figure 1 presents the reaction route map encompassing isomerization pathways in the form of EQ*x*-TS*n*-EQ*y* for the five most thermodynamically stable isomers. The energetics and characteristics of these pathways are detailed in Table 1, with all relative energies referenced to the initial EQ0 isomer. Among the five key isomers identified in this study, EQ3 emerged as the most thermodynamically stable (−10.56 kcal/mol), followed by EQ7 (−4.91 kcal/mol), EQ0 (reference isomer, 0 kcal/mol), EQ1 (21.46 kcal/mol), and EQ12 (33.98 kcal/mol).

The geometry of the initial isomer, EQ0 (H-*c*-CC(O)C-H), was validated through comparison with both experimental and theoretical studies [17,18,19,20,27]. The consistent agreement between our calculated geometrical parameters and previously published results confirms the reliability of our chosen level of theory for conformational exploration. Detailed structural comparisons are presented in Supplementary Materials (Figure S1 and Table S1). The reaction rate constants, incorporating quantum tunneling corrections for the selected isomerization pathways, are presented in Figures S2–S4, with detailed data shown in Table S2.

The primary route to EQ3 formation involves the ring opening of H_2_C-*c*-CCO (EQ4) via C-O bond cleavage in the three-member ring. This transformation proceeded along the EQ4-TS8-EQ3 pathway with a relatively modest energy barrier of 15.69 kcal/mol. Additionally, OC(H)CCH (EQ7) was converted into H_2_CCCO via the EQ7-TS70-EQ3 pathway, which involved concurrent hydrogen atom migration and carbon atom rearrangement. However, this pathway required surmounting a substantial energy barrier of 79.21 kcal/mol, indicating its less favorable profile. Similarly, HOCCCH (EQ8) isomerized to H_2_CCCO through the EQ8-TS57-EQ3 pathway, featuring carbon reorganization with hydrogen migration and an intermediate energy barrier of 44.48 kcal/mol. In contrast, EQ3 underwent cyclization to form H_2_-*c*-COC-C (EQ20) via the EQ3-TS77-EQ20 pathway. This transformation necessitated a substantial energy barrier of 96.61 kcal/mol due to the concerted carbon atom rearrangement and hydrogen migration required, further confirming the thermodynamic stability of the EQ3 structure. The kinetic analysis corroborated these findings, yielding an extremely low-rate constant of 1.472 × 10^−58^ s^−1^ at 298 K, indicating that this cyclization pathway is effectively inaccessible under ambient conditions.

The OC(H)CCH isomer (EQ7) underwent cyclization with concurrent hydrogen atom migration via the EQ7-TS11-EQ0 pathway, transforming into the starting structure EQ0. This isomerization necessitated the overcoming of a high energy barrier of 57.33 kcal/mol. In contrast, the cyclic H-*c*-CCOC-H structure (EQ2) exhibited a significant conformational rearrangement from a four-member ring to EQ0 via the EQ2-TS49-EQ0 pathway, requiring a comparatively modest energy barrier of 29.52 kcal/mol. Nevertheless, this isomerization exhibited kinetically less favorable characteristics with a low reaction rate constant of 1.966 × 10^−9^ s^−1^ at 298 K, indicating negligible transformation under ambient conditions. Moreover, a notable ring-opening process converted the cyclic isomer EQ0 into the global stable isomer H_2_CCCO (EQ3) via the EQ0-TS18-EQ3 pathway, overcoming a barrier of 75.58 kcal/mol through concurrent carbon framework reorganization and hydrogen migration.

The HCC(H)CO isomer (EQ1), positioned 21.46 kcal/mol higher in energy relative to the reference structure EQ0, participates in several significant transformational pathways. Most notably, EQ1 underwent cyclization to form the H-*c*-CC(O)C-H isomer via TS0. This intramolecular transformation proceeded with a moderate energy barrier of 8.61 kcal/mol, resulting in the formation of a more thermodynamically stable cyclic product. The calculated rate constant of 6.286 × 10^6^ s^−1^ at 298 K indicated rapid isomerization under ambient conditions.

Multiple isomerization pathways lead to the formation of EQ1. The global stable isomer, H_2_CCCO (EQ3), can transform into HCC(H)CO (EQ1) via the EQ3-TS7-EQ1 pathway. This transformation, which proceeded through hydrogen atom migration and molecular rearrangement, required us to overcome a substantial activation energy barrier of 76.94 kcal/mol. Similarly, the OC(H)CCH isomer exhibited conformational versatility, enabling hydrogen migration and structural reorganization through the EQ7-TS10-EQ1 pathway. This isomerization process, culminating in the formation of HCC(H)CO, proceeded with a substantial activation energy barrier of 60.56 kcal/mol. Moreover, an additional pathway leads to the formation of HCC(H)CO through the isomerization of OC(H)C(H)C via the EQ11-TS20-EQ1. This transformation proceeded with a comparatively moderate activation energy barrier of 15.75 kcal/mol. Kinetic analysis reveals that this isomerization is accessible with a rate constant of 2.902 × 10^1^ s^−1^ at 298 K.

The formation of the cyclic HO-*c*-CCC-H (EQ12) occurred through multiple pathways. Primarily, the terminal hydrogen atom underwent migration, facilitating the formation of a hydroxyl moiety and subsequent cyclization to generate EQ12 via the EQ1-TS37-EQ12 pathway. This transformation proceeded with a substantial energy barrier of 98.01 kcal/mol, attributable to the significant thermodynamic instability inherent in the strained three-membered ring structure. Alternatively, EQ12 can also be derived from the HOC(H)CC isomer (EQ15) through a cyclization pathway (EQ15-TS44-EQ12), wherein the hydroxyl group migrates to facilitate ring formation. This route required the overcoming of a similarly significant activation energy barrier of 97.51 kcal/mol. The linear HOCCCH structure (EQ8) can also transform into EQ12 via an alternative isomerization pathway (EQ8-TS32-EQ12), which exhibits a lower energy barrier of 47.52 kcal/mol. This transformation corresponds to a rate constant of 1.165 × 10^−22^ s^−1^ at 298 K, indicating a kinetically inaccessible process under ambient conditions. Mechanistically, the conversion proceeded through a mechanistic sequence comprising C-C bond cleavage, conformational reorganization, and subsequent ring formation. The cyclization of the linear structure EQ10 into EQ12 via the pathway EQ10-TS27-EQ12 necessitates the overcoming of an energy barrier of 45.03 kcal/mol, which encompasses hydrogen migration, C-C bond cleavage, molecular reorganization, and subsequent ring formation, exhibiting energetic requirements comparable to other isomerization pathways leading to the EQ12 isomer.

Once formed, EQ12 can undergo several transformations. For instance, hydrogen atom migration within the hydroxyl moiety facilitates the formation of the H-*c*-CC(O)C-H structure through the EQ12-TS22-EQ0 pathway, which exhibits a high energy barrier of 50.16 kcal/mol. EQ12 can undergo ring opening and rearrangement via the EQ12-TS30-EQ14 pathway, yielding the HOC(H)CC isomer (EQ14) with a high energy barrier of 61.93 kcal/mol. An additional notable transformation involves conformational interconversion between two HO-*c*-CCC-H isomers via the EQ13-TS26-EQ12 pathway, exhibiting a minimal energy barrier of 8.33 kcal/mol, corresponding to a rapid reaction rate of 7.300 × 10^6^ s^−1^ at 298 K. This process is characterized by the hydroxyl moiety, forming bonds with different carbon atoms within the cyclic framework.

Multiple isomerization pathways of the OC(H)CCH structure (EQ7) have been identified. Several species demonstrate conversion into EQ7 through distinct mechanisms. The HOC(H)CC structure isomerizes into OC(H)CCH via the EQ14-TS41-EQ7 pathway, involving hydroxyl-to-aldehyde conversion with an activation energy of 33.75 kcal/mol. OC(H)C(H)C (EQ11) undergoes terminal hydrogen migration through pathway EQ11-TS14-EQ7, yielding OC(H)CCH with a notably low barrier of 1.41 kcal/mol. This process represents rapid isomerization at ambient conditions, with a calculated rate constant of 7.714 × 10^11^ at 298 K. HCOCCH transforms into OC(H)CCH (EQ6-TS74-EQ7) through molecular rearrangement and hydrogen migration, requiring 45.73 kcal/mol. Cyclic structures contribute to EQ7 formation through ring-opening processes. H-*c*-CCC(H)O (EQ26) isomerized into EQ7 via pathway EQ26-TS100-EQ7, involving ring opening, rearrangement, and hydrogen migration with a minimal barrier of 1.06 kcal/mol and a correspondingly rapid rate constant of 1.448 × 10^12^ s^−1^ at 298 K. Another thermodynamically and kinetically favorable isomerization (1.57 kcal/mol and 4.970 × 10^11^ s^−1^, 298 K) connects cyclic H-*c*-CCC(O)-H (EQ17) to EQ7 through the EQ17-TS103-EQ7 pathway.

Conversely, EQ7 can undergo various transformations to form different isomers. The cyclization into H-*c*-COC(H)C proceeded via EQ7-TS42-EQ16, requiring a substantial activation energy of 76.62 kcal/mol. The kinetic analysis yields a rate constant of 5.724 × 10^−44^ s^−1^ at 298 K), suggesting that this cyclization mechanism is both thermodynamically and kinetically impossible under ambient conditions. A hydrogen shift facilitates HOCCCH isomer formation (EQ10) via EQ7-TS13-EQ10, involving aldehyde-to-hydroxyl conversion with an activation barrier of 77.67 kcal/mol. Higher-energy transformations include conversion into HCOC(H)C via EQ7-TS43-EQ18, requiring 125.86 kcal/mol and the rearrangement of −C(O)H and −CCH moieties with concurrent hydrogen migration. EQ7 transforms into CC(H)C(H)O (EQ5) via EQ7-TS15-EQ5 through hydrogen migration and molecular rearrangement, surmounting a lower barrier of 44.16 kcal/mol. Additionally, EQ7 isomerizes into linear HCOCCH via EQ7-TS17-EQ9 through carbon and oxygen rearrangement, with a significant activation energy of 104.14 kcal/mol. The kinetic analysis further confirmed that the isomerization is inaccessible, yielding a negligible rate constant of 4.127 × 10^−64^ at 298 K.

### 2.2. TS-Mediated Dissociation Pathways (EQa-TSb-DC)

**Figure 2:**
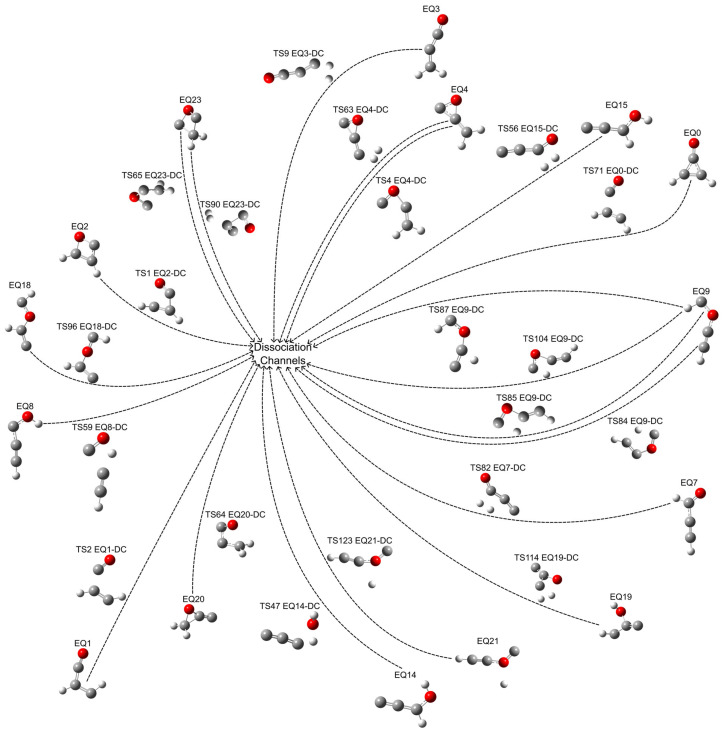
TS-mediated dissociation channels represented as EQ*a*-TS*b*-DC pathways. Atoms colored red represent oxygen, gray represent carbon, and white represent hydrogen.

Our ADDF calculations elucidated 20 distinct TS-mediated dissociation pathways across the C_3_H_2_O isomeric landscape. These pathways encompass diverse processes, including ring-opening transformation, hydrogen dissociation, and fragment decompositions, each characteristic of specific activation energies. The relative energies and energy barriers associated with these dissociation channels are comprehensively detailed in Table 2, with the corresponding isomerization processes illustrated schematically in Figure 2. Reaction rate constants for the selected TS-mediated dissociation pathways with quantum tunneling corrections are graphically depicted in Figures S5 and S6 and summarized in Table S2 of the Supplementary Materials.

Conformational analysis revealed distinct ring-opening mechanisms for cyclic isomer dissociation. The H-*c*-CCOC-H isomer underwent initial ring cleavage through the C-O bond scission in the four-membered ring via the EQ2-TS1-DC pathway with a moderate energy barrier of 25.71 kcal/mol. In contrast, the H_2_C-*c*-CCO involved C-C bond cleavage within the ring structure, isomerizing through a H_2_CCOC transition state via the EQ4-TS4-DC pathway with a higher energy barrier of 36.82 kcal/mol. Similarly, the ring opening of the H_2_-*c*-COC-C isomer yields OCCCH_2_ through the EQ20-TS64-DC pathway, requiring 36.24 kcal/mol. The kinetic analysis of this transformation revealed a rate constant of 2.899 × 10^−14^ s^−1^ at 298 K, suggesting that this dissociation would be unfavorable. Notably, the H_2_-*c*-CCOC system exhibited two distinct mechanistic pathways: a direct ring cleavage mechanism involving C-C bond scission via EQ23-TS65-DC with a negligible energy barrier of 1.76 kcal/mol and corresponding rapid rate constant of 3.909 × 10^11^ s^−1^ at 298 K, and a more complex concurrent process involving simultaneous ring opening and H_2_ formation during dissociation via EQ23-TS90-DC. This process required a substantial energy barrier of 73.43 kcal/mol. The kinetic analysis indicated this dissociation pathway to be prohibitive, with an extremely low-rate constant of 1.571 × 10^−41^ s^−1^ at 298 K.

Various hydrogen dissociation pathways were characterized across the C_3_H_2_O isomeric landscape. The cyclic H_2_C-*c*-CCO isomer (EQ4) exhibited a distinct dissociation mechanism: molecular H_2_ formation via EQ4-TS63-DC, which required a substantial energy barrier of 81.07 kcal/mol. The kinetic analysis of this pathway revealed an extremely low-rate constant of 4.267 × 10^−47^ s^−1^ at 298 K, rendering this transformation impossible under ambient conditions. Meanwhile, HCOCCH (EQ9) underwent a series of transformations, including single-hydrogen-atom dissociation via EQ9-TS85-DC and EQ9-TS84-DC with high energy barriers of 50.73 and 50.33 kcal/mol, respectively, structural rearrangement along EQ9-TS104-DC with a high energy barrier of 59.73 kcal/mol, and movements through an alternative hydrogen dissociation channel via EQ9-TS87-DC with a lower barrier of 45.78 kcal/mol. The thermodynamically favorable H_2_CCCO (EQ3) exhibited hydrogen atom dissociation through the EQ3-TS9-DC pathway, but was required to surmount a substantial energy barrier of 80.77 kcal/mol. HOC(H)CC (EQ15) and OC(H)CCH (EQ7) proceeded via EQ15-TS56-DC and EQ7-TS82-DC pathways with high (59.27 kcal/mol) and substantial (109.69 kcal/mol) energy barriers, respectively. Furthermore, HOC(C)CH (EQ19) exhibited hydrogen dissociation through EQ19-TS114-DC with a moderate energy barrier of 40.02 kcal/mol, while the COCCH^−^ + H^+^ system progressed via the EQ21-TS123-DC pathway with a negligible energy barrier of only 0.89 kcal/mol and ultrafast dissociation kinetics (2.141 × 10^12^ s^−1^ at 298 K).

Regarding fragment dissociation reactions, multiple isomeric structures demonstrated characteristic decomposition pathways with varying energetic requirements. HCC(H)CO underwent rapid dissociation (2.100 × 10^8^ s^−1^ at 298 K) via the EQ1-TS2-DC pathway to yield CO and HCCH fragments with a minimal energy barrier of 6.31 kcal/mol. The HOC(H)CC structure fragmented via the EQ14-TS47-DC pathway to form −OH and CCCH species, requiring a substantial energy barrier of 83.68 kcal/mol, whereas HOCCCH produced −COH and HCC− fragments through the EQ8-TS59-DC pathway with an energy barrier of 47.72 kcal/mol. The cyclic H-*c*-CC(O)C-H underwent dissociation via EQ0-TS71-DC with a moderate energy barrier of 36.09 kcal/mol, yielding CO and HCCH fragments. However, this dissociation exhibited kinetically unfavorable characteristics with a negligible rate constant of 3.804 × 10^−14^ s^−1^ at 298 K, indicating its inaccessibility under ambient conditions. Several species exhibited more complex behavior, with dissociation accompanied by rotational isomerization, as HCOC(H)C went through the EQ18-TS96-DC pathway, requiring a moderate energy barrier of 24.02 kcal/mol. Despite this seemingly accessible barrier, kinetic calculations revealed a relatively low-rate constant of 1.985 × 10^−5^ s^−1^ at 298 K, indicating that there was limited dissociation under ambient conditions.

### 2.3. Direct Dissociation Pathways (EQm-DCn)

**Figure 3:**
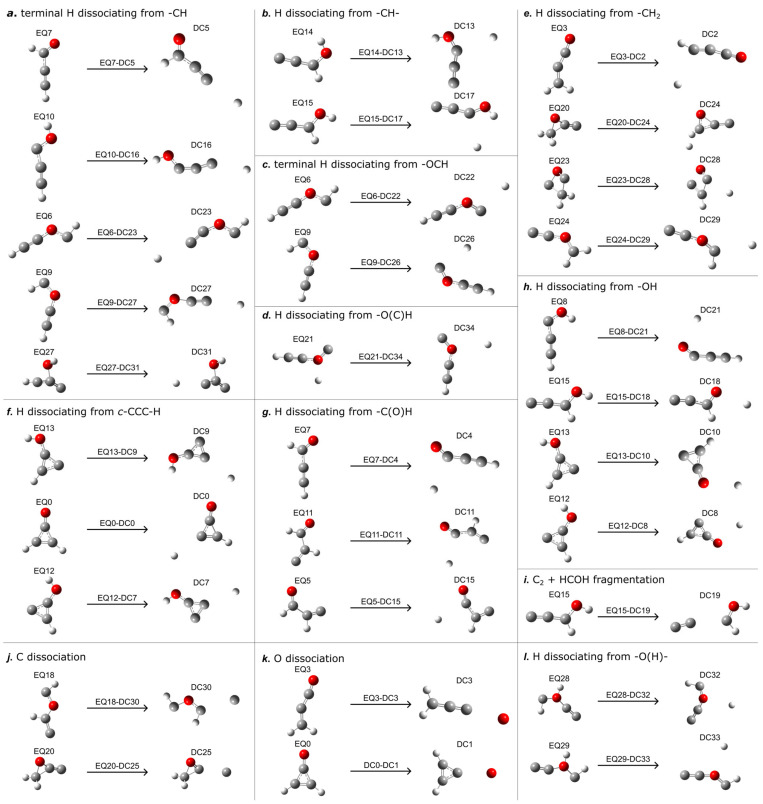
Direct dissociation channels for C_3_H_2_O isomers. The notation EQ*m*-DC*n* indicates the pathway from EQ*m* to DC*n*. Atoms colored red represent oxygen, gray represent carbon, and white represent hydrogen.

The reaction route network revealed 31 distinct direct dissociation channels. The corresponding pathways are illustrated in Figure 3, and their energetic relationships are presented in Table 3. The investigated isomers exhibited distinctly different direct dissociation behaviors, with significantly varying energy barriers. Most dissociation pathways require substantial energy barriers to be overcome, indicating that we are dealing with thermodynamically unfavorable processes unlikely to occur spontaneously without sufficient energy input. Terminal hydrogen dissociation from −CH groups (Figure 3a) occurred across multiple isomers with barriers ranging from 83.94 to 150.55 kcal/mol, while internal −CH− hydrogen elimination (Figure 3b) proceeded via pathways with barriers of 92.13–118.16 kcal/mol. The −OCH moiety (Figure 3c) underwent terminal hydrogen dissociation, with substantial energy barriers (88.72–92.39 kcal/mol).

Notably, hydrogen elimination from −O(C)H structures (Figure 3d) exhibited the lowest energy barrier (13.23 kcal/mol). The −CH_2_ groups (Figure 3e) demonstrated selective hydrogen dissociation with barriers between 87.37 and 129.19 kcal/mol, while cyclic structures (Figure 3f) displayed site-specific C-H bond cleavage with barriers of 110.63–121.27 kcal/mol. Aldehyde groups (Figure 3g) underwent selective hydrogen elimination with energy barriers of 93.01–104.51 kcal/mol.

Hydroxyl bond cleavage (Figure 3h) represented a significant pathway across several isomers with substantial energy barriers (61.47–104.33 kcal/mol), while selective hydroxyl hydrogen dissociation from bridging −O(H)− moieties (Figure 3l) proceeded with a lower energy barrier of 35.10–64.79 kcal/mol. In contrast, complete fragmentation into C_2_ and HCOH (Figure 3i) required a substantial barrier of 141.91 kcal/mol to be surmounted. Carbon atom dissociation (Figure 3j) proceeded with high activation energies (141.36–173.40 kcal/mol), while oxygen elimination (Figure 3k) exhibited the highest barriers observed in this study (180.71–210.55 kcal/mol).

### 2.4. Benchmarking and Validation of the Exploration Method

**Figure 4:**
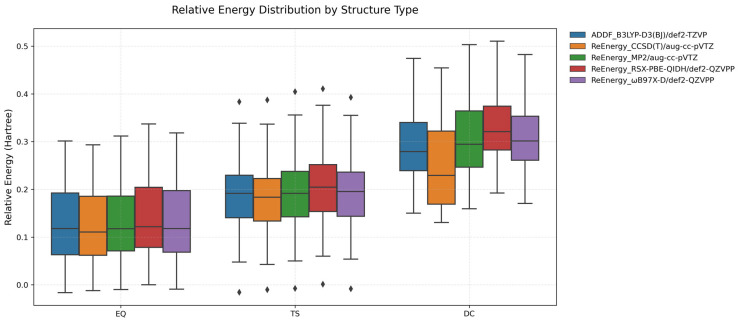
Relative energy distributions of EQ, TS, and DC structures at different levels of theory, with EQ0 as the reference.

Comparative analysis of multiple computational methods indicates the high accuracy for the current exploration method, B3LYP-D3(BJ)/def2-TZVP. The comparisons are presented in Figure 4, where box plots depict relative energy distributions across structural types and computational methods. In these plots, the rectangular box represents the interquartile range (Q_1_–Q_3_), with the internal horizontal line indicating the median. Whiskers extend to data points within 1.5 × IQR from the quartiles, while diamond-shaped markers denote statistical outliers exceeding this threshold, which are primarily observed in TSs, where electronic configuration complexity introduces greater method sensitivity.

Figures S7–S9 provide comprehensive distributions of relative energies for EQs, TSs, and DCs, respectively. The relative energy distributions exhibit method-dependent patterns that correlate with structural complexity. Most deviations were acceptable, although structures with dissociating tendencies (for example, where an atom is positioned relatively far from the backbones) showed greater variations. For EQs (Figure S7), all methods display exceptional concordance, with nearly superimposable energy profiles and minimal standard deviations (≈0.09 Hartree), validating the robust description of stable geometries across theoretical frameworks.

TSs (Figure S8) manifest moderate energetic variations among methods (σ = 0.07–0.08 Hartree). It is worth noting that these calculations incorporate both true TSs and numerical TSs in ADDF results. When this distinction is accounted for, the observed deviations can be further reduced, as numerical TSs may introduce additional variability due to their approximate nature in representing the exact saddle point. Notably, B3LYP-D3(BJ)/def2-TZVP maintains consistent relative energy trends when benchmarked against CCSD(T)/aug-cc-pVTZ, indicating that our computational protocol effectively captures the intricate electronic character of these higher-energy configurations while offering substantial computational efficiency compared to ωB97X-D/def2-QZVPP, RSX-PBE-QIDH/def2-QZVPP, and MP2/aug-cc-pVTZ methodologies.

DC structures demonstrate pronounced method-dependent variations across computational approaches, particularly in high-energy regions (e.g., DC28–DC34), as illustrated in Figure S9. This methodological sensitivity aligns with theoretical expectations, as dissociation processes necessarily entail the substantial reconfiguration of electronic structure and long-range interactions, which present formidable challenges for consistent quantitative description across diverse theoretical frameworks. The notable divergence between CCSD(T)/aug-cc-pVTZ and alternative methods for specific DC structures highlights the intrinsic difficulties in achieving the uniform characterization of bond-breaking events across different levels of theory. Nevertheless, the B3LYP-D3(BJ)/def2-TZVP adequately reproduces the fundamental features of DC structures, establishing its utility as an efficient and reliable methodology for preliminary investigations of dissociation pathways.

In conclusion, B3LYP-D3(BJ)/def2-TZVP demonstrated robust performance across all structural categories, with high fidelity in characterizing EQ geometries and successfully describing TSs. While dissociation energetics necessitate careful interpretation within the context of method-specific constraints, this approach constitutes an optimal methodology for comprehensive conformational analysis due to its favorable balance of accuracy and computational efficiency.

### 2.5. Water-Involved Bimolecular Reactions

MC-AFIR calculations were conducted to investigate the potential products of bimolecular collisions. The five most stable isomers, EQ3, EQ7, EQ0, EQ1, and EQ12, were selected for reaction with a single H_2_O molecule. Bond formation was excluded when interatomic distances exceeded 1.5 Å or fragments were only connected through shared hydrogen atoms.

#### 2.5.1. Potential Products from EQ3 (H_2_CCCO) + H_2_O

**Figure 5:**
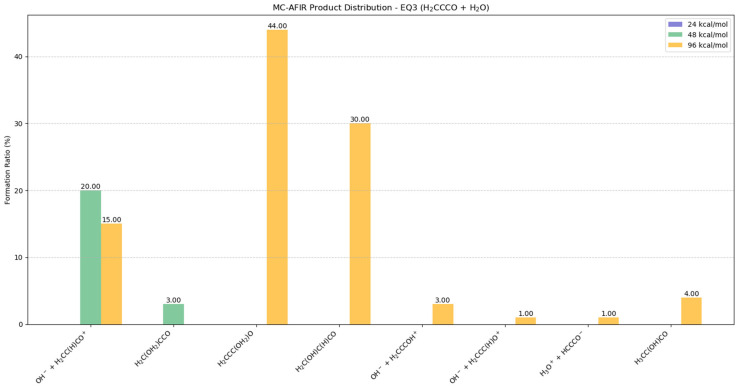
Potential distributions resulting from collisions between EQ3 (H_2_CCCO) and H_2_O molecules. Formation ratios of MC-AFIR products with distinct artificial forces are shown in different colors.

The collision exhibits a diverse product distribution with strong energy dependence, as illustrated in Figure 5 and Supplementary Materials, Table S3. As EQ3 represented the global minima, structural rearrangement required significant activation barriers to be overcome, potentially influencing the collision of two fragments.

At 24 kcal/mol, no reactions were observed in the MC-AFIR calculation.

At 48 kcal/mol, OH^−^ + H_2_CC(H)CO^+^ formation exhibits notable yields at both energy levels, reaching 20% at 48 kcal/mol and maintaining a significant presence (15%) at 96 kcal/mol. This persistent ionic product distribution suggested that the dissociative pathway remains favorable across collision energies, with slightly enhanced preference under milder conditions. The ionic species OH^−^ + H_2_CC(H)CO^+^, formed during water interactions with the carbon atom, exhibits significant instability. This transient structure underwent spontaneous dissociation within 4.61 fs, as illustrated in Figure S10. Minor products include H_2_C(OH_2_)CCO (3% at 48 kcal/mol) and several products at 96 kcal/mol with yields of approximately 1–4% OH^−^ + H_2_CCCOH^+^, OH^−^ + H_2_CCC(H)O^+^, H_3_O^+^ + HCCCO^−^, and H_3_CC(OH)CO, indicating the less favorable reaction channels involving ionic species.

At 96 kcal/mol, H_2_CCC(OH_2_)O emerged as the dominant product with a 44% yield, followed by H_2_C(OH)C(H)CO at 30%. BOMD simulations revealed that H_2_CCC(OH_2_)O exhibits dissociative behavior, with the water molecule forming only transient stable interactions with EQ3 (<5.69 fs), as shown in Figure S11. In contrast, the H_2_C(OH)C(H)CO adduct maintains structural integrity throughout the simulation, suggesting it represents a thermodynamically favorable product formed via the interaction between EQ3 and water, as shown in Figure S12.

#### 2.5.2. Potential Products from EQ7 (OC(H)CCH) + H_2_O

**Figure 6:**
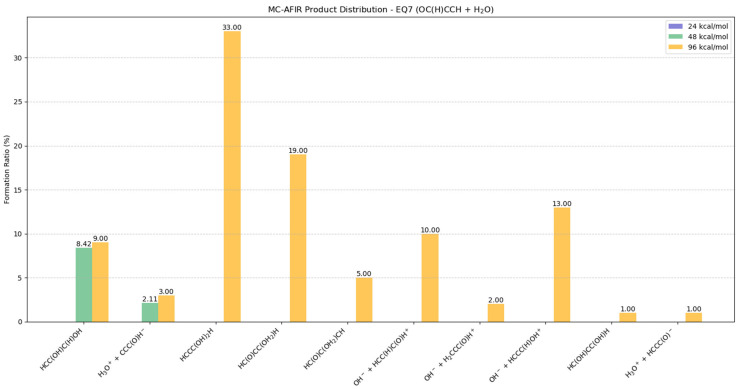
Potential distributions resulting from collisions between EQ7 (OC(H)CCH) and H_2_O molecules. Formation ratios of MC-AFIR products with distinct artificial forces are shown in different colors.

At 24 kcal/mol, no reactions were observed in the MC-AFIR calculations, as illustrated in Figure 6 and Supplementary Materials, Table S4. At moderate energy (48 kcal/mol), the system exhibited decreased reactivity, yielding primarily HCC(OH)C(H)OH forms (8.42%) with minimal ionic product (H_3_O^+^ + CCC(O)H^−^, 2.11%).

At 96 kcal/mol, HCCC(OH)_2_H emerges as the predominant product (33%), with BOMD simulations confirming its structural stability without subsequent dissociation, as illustrated in Figure S13. Secondary products include HC(O)CC(OH_2_)H (19%) and HCC(OH)C(H)OH (9%). Notable ionic products include OH^−^ + HCCC(H)OH^+^ (13%) and OH^−^ + HCC(H)C(O)H^+^ (10%). Additional minor products observed at this energy level include H_3_O^+^ + CCC(O)H^−^ (3%), HC(O)C(OH_2_)CH (5%), OH^−^ + H_2_CCC(O)H^+^ (2%), H_3_O^+^ + HCCC(O)^−^ (1%), and HC(OH)CC(OH)H (1%), arising from less energetically favorable collision geometries. Among these structures, BOMD simulations revealed that HC(O)CC(OH_2_)H exhibits dissociative behavior (<10.23 fs), as illustrated in Figure S14.

#### 2.5.3. Potential Products from EQ0 (H-c-CC(O)C-H) + H_2_O

**Figure 7:**
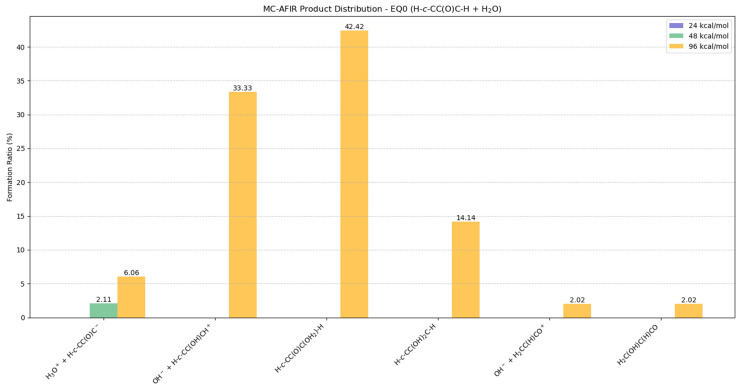
Potential distributions resulting from collisions between EQ0 (H-*c*-CC(O)C-H) and H_2_O molecules. Formation ratios of MC-AFIR products with distinct artificial forces are shown in different colors.

The EQ0 isomer exhibited remarkable stability, requiring substantial energies to initiate or facilitate an effective collision, as evidenced by the product distributions presented in Figure 7 and Supplementary Materials, Table S5. At 24 kcal/mol, no reactions were observed in the MC-AFIR calculations. In contrast, at 48 kcal/mol, the formation of H_3_O^+^ + H-*c*-CC(O)C^−^ is limited to 2.11%, indicating reduced reactivity across all potential products.

When γ = 96 kcal/mol, the reaction predominantly favors two major products: H-*c*-CC(O)C(OH_2_)-H (42.42%) and the ionic species OH^−^ + H-*c*-CC(OH)CH^+^ (33.33%). A cyclic product H-*c*-CC(OH)_2_C-H constitutes the secondary product, comprising 14.14% of the product distribution. BOMD simulations revealed that these structures exhibit dissociative behavior. Notably, the EQ0 isomer maintains its intrinsic stability, preserving its cyclic structure when interacting with water molecules, which generally did not induce EQ0 dissociation, as illustrated in Figures S15 and S16. Minor products observed at 96 kcal/mol include the ionic species OH^−^ + H_2_CC(H)CO^+^ (2.02%) and H_3_O^+^ + H-*c*-CC(O)C^−^ (6.06%), as well as the neutral species H_2_C(OH)C(H)CO, present at around a 2.02% rate, indicating their low formation propensities with a single water molecule.

#### 2.5.4. Potential Products from EQ1 (HCC(H)CO) + H_2_O

**Figure 8:**
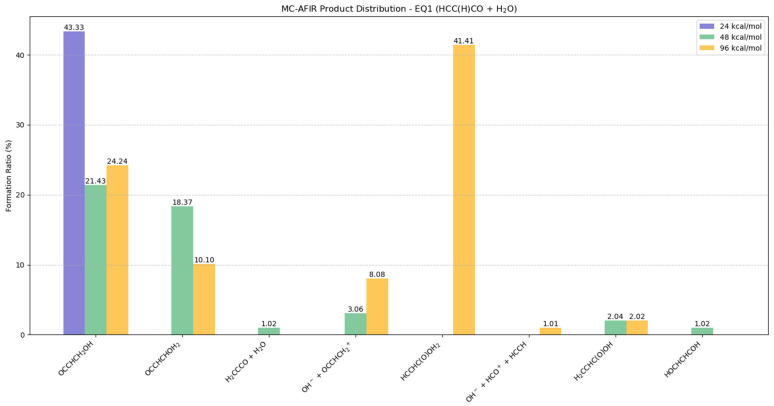
Potential distributions resulting from collisions between EQ1 (HCC(H)CO) and H_2_O molecules. Formation ratios of MC-AFIR products with distinct artificial forces are shown in different colors.

The collision of EQ1 with the H_2_O molecule exhibited complex distributions of potential products, indicating that EQ1 had a high probability of occurring reactions in the presence of water molecules, as illustrated in Figure 8 and Supplementary Materials, Table S6.

At 24 kcal/mol, OCCHCH_2_OH is the predominant product, comprising 43.33% of the distribution, though its formation propensity decreased to 21.43% and 24.24% at 48 and 96 kcal/mol, respectively. The stability of OCCHCH_2_OH was confirmed through BOMD simulations, demonstrating its viability as a potential product in reactions with water molecules, as illustrated in Figure S17.

At 48 kcal/mol, OCCHCHOH_2_ forms in significant quantities (18.37%). However, BOMD simulations indicate that the water molecule subsequently dissociates, suggesting this might not represent a stable structure (Figure S18). Several additional transformations were observed: isomerization to H_2_CCCO occurred at low yield (1.02%), while both ionic species (OH^−^ + OCCHCH_2_^+^) and neutral species H_2_CCHC(O)OH and HOCHCHCOH formed at 3.06%, 2.04%, and 1.02%, respectively.

At 96 kcal/mol, HCCHC(O)OH_2_ becomes the principal product, constituting 41.41% of the distribution. This structure exhibits dissociative properties, with no subsequent transformations observed in BOMD simulations (Figure S19). Minor products include OCCHCHOH_2_ (10.10%), the ionic pair OH^−^ + OCCHCH_2_^+^ (8.08%), and the fragmentation products OH^−^ + HCO^+^ + HCCH (1.01%) and H_2_CCHC(O)OH (2.02%).

#### 2.5.5. Potential Products from EQ12 (HO-c-CCC-H) + H_2_O

**Figure 9:**
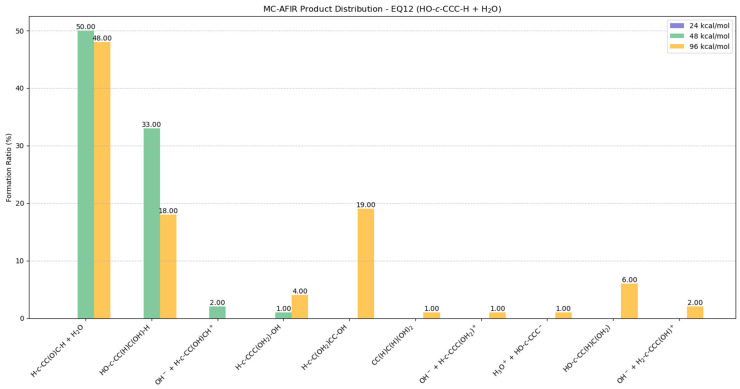
Potential distributions resulting from collisions between EQ12 (HO-*c*-CCC-H) and H_2_O molecules. Formation ratios of MC-AFIR products with distinct artificial forces are shown in different colors.

The EQ12 isomer demonstrated diminished ionic species formation while preferentially isomerizing to EQ0 in the presence of a single water molecule (Figure 9 and Supplementary Materials, Table S7). At 24 kcal/mol, no reactions were observed in the MC-AFIR calculations. The distributions revealed high selectivity for EQ0 (H-*c*-CC(O)C-H) + H_2_O formation, yielding 50% and 48% values at collision energies of 48 kcal/mol and 96 kcal/mol, respectively. BOMD simulations indicate that the subsequent reaction was unfavorable, as illustrated in Figure S20.

HO-*c*-CC(H)C(OH)-H emerges as the second predominant product, with yields of 33% at 48 kcal/mol and 18% at 96 kcal/mol. The structural integrity of this compound was confirmed through BOMD simulations, as illustrated in Figure S21, establishing its viability as a product formed via reaction with water molecules. H-*c*-C(OH_2_)CC-OH constitutes a notable minor product at 19% yield. Several additional minor products are observed with yields ranging from 1 to 6%: CC(H)C(H)(OH)_2_, OH^−^ + H-*c*-CCC(OH_2_)^+^, H_3_O^+^ + HO-*c*-CCC^−^, HO-*c*-CC(H)C(OH_2_), and OH^−^ + H_2_-*c*-CCC(OH)^+^. These minor products indicated the existence of alternative reaction pathways involving ionic species formation, although the products appeared to be thermodynamically less favorable.

## 3. Computational Details

The global reaction route mapping (GRRM) program (version GRRM17) [28,29], which interfaces with Gaussian 16 [30], was employed to conduct a comprehensive exploration of the PES of C_3_H_2_O isomers. The anharmonic downward distortion following (ADDF) algorithm [31,32,33,34,35] implemented in the GRRM program facilitates the comprehensive exploration of PES by identifying stable minima through their characteristic anharmonic downward distortion (ADD). The ADD serves as a directional indicator for pathways across TSs to other minima or DCs. In this approach, the ADDF method detects and follows ADDs, starting from local minima, to locate reaction paths. While ADDF paths are not identical to intrinsic reaction coordinate (IRC) paths, they typically pass through regions near actual transition states, providing approximate TSs as the highest energy points along these paths.

For each approximate TS identified along an ADDF path, subsequent optimization procedures are implemented to locate the corresponding true TS. Upon successful detection of a true TS, meta-IRC calculations (mass-weighted steepest descent paths) are executed from the terminal ADDF path point to determine the corresponding product structure, which is either an alternative local minimum or a DC. This systematic procedure allows for the global exploration of EQ, TS, and DC structures on the PES without requiring the entirely manual elucidation of reaction pathways. When a molecule decomposes into two or more fragments during this process, the path is classified as reaching a DC. The algorithm continues following all ADDF paths until they culminate in either minima or DCs, thereby ensuring the exhaustive mapping of the reaction network.

ADDF calculations were initialized using an optimized cyclopropenone isomer structure. Both initial geometry optimization and a conformational search were conducted at the B3LYP-D3(BJ)/def2-TZVP level of theory [36,37,38,39]. The accuracy of identified isomers was validated through benchmark single-point-energy calculations at several higher levels of theory: the range-separated hybrid functional ωB97X-D with a def2-QZVPP basis set, refs. [38,39,40] the range-separated double-hybrid functional RSX-PBE-QIDH with a def2-QZVPP basis set [38,39,41,42], the second-order Møller-Plesset perturbation theory (MP2) with an aug-cc-pVTZ basis set [43,44,45], and coupled-cluster theory with single, double, and perturbative triple excitations (CCSD(T)) with an aug-cc-pVTZ basis set [44,45,46].

The ADDF calculation changes EQ0 to EQ*x*, TS0 to TS*n*, and DC0 to DC*n*. When a TS connects two EQs, it establishes an EQ*x*-TS*n*-EQ*y* pathway. Similarly, when a TS links an EQ to a DC, it forms an EQ*a*-TS*b*-DC pathway. Additionally, direct connections between EQs and DCs are denoted as EQ*m*-DC*n*. For clarity, the variables *x*, *y*, *m*, *n*, *a*, and *b* represent specific indices in the sequence. Dissociation was defined as occurring when the distance between atom A and atom B exceeded 0.1 × *n* × 2 × (R^A^ + R^B^); *n* = 10 in this study. The GRRM algorithm halts exploration when dissociation criteria are met, either terminating without products or identifying dissociating species (DC*n*). The global reaction route of cyclopropenone was mapped using the validated EQ, TS, and DC structures. The collected routes included isomerization processes, TS-mediated dissociation mechanisms, and direct dissociation routes. All energetics in the global reaction route maps were corrected with zero-point vibrational energy (ZPVE), and calculations were performed at 0 K. Several conformations and related pathways were excluded from the final analysis based on rigorous criteria: (1) nominal TS*n*/*n* pathways that reverted to an EQ*n* structure without exhibiting genuine TSs; (2) TSs lacking well-defined connections to EQs; (3) TSs forming direct connections between two DCs; and (4) TSs that failed to establish thermodynamically viable pathways after ZPVE corrections.

The kinetic analyses in this study provide preliminary insights but necessitate methodological refinements to account for tunneling effects and reaction dynamics accurately. While the proposed isomerization pathways may exhibit limited thermodynamic feasibility at low temperatures, comprehensive kinetic investigations would elucidate the reaction mechanism. Our preliminary reaction rate estimations, conducted over the temperature range of 150–450 K, employed three different theoretical approaches: the traditional transition state theory (TST) [47], Wigner-corrected TST [48], and Eckart-corrected TST [49,50,51]. The kinetic investigation was constrained by limitations in both the GRRM output formats and the implemented tunneling correction approaches. Future studies, employing more sophisticated computational approaches, are warranted to refine the calculated rate constants, particularly under low-temperature conditions. Notably, transition states exhibiting low imaginary frequencies present a significant challenge, as Eckart tunneling corrections can increase exponentially at reduced temperatures, resulting in numerical instabilities that compromise analytical rigor. Consequently, this work only reports the numerically stable and physically meaningful rate constants. The implementation of advanced computational methodologies and more robust kinetic analyses may elucidate alternative reaction pathways that circumvent the thermodynamic constraints identified herein.

The collision reaction between stable isomers and water molecules, including potential reaction products, was systematically investigated using the multicomponent artificial force-induced reaction (MC-AFIR) algorithm [52,53,54] available within the GRRM program. Initial configurations were generated through random distributions of two fragments, allowing the sampling of the AFIR path to explore potential collision products across diverse conformational arrangements.

Collision energy parameters (γ) of 24, 48, and 96 kcal/mol were employed. To ensure reproducibility, calculations at each γ value were performed in duplicate for each selected stable isomer. The calculations were terminated upon reaching 50 AFIR paths (NSample = 50) or encountering 10 paths that failed to identify new products (NFault = 10). All MC-AFIR calculations were performed at the B3LYP-D3(BJ)/def2-TZVP level of theory.

The MC-AFIR simulations generated a diverse array of collision products between C_3_H_2_O isomers and water molecules. To further investigate the dynamic evolution of these products and explore potential subsequent transformations, including fragment recombination pathways that might lead to further isomerization, Born–Oppenheimer Molecular Dynamics (BOMD) simulations were conducted, starting from selected structures identified in the MC-AFIR calculations. The BOMD calculations were performed at the B3LYP-D3(BJ)/def2-TZVP with Gaussian 16. Trajectories were propagated for 2000 steps with the dynamic step size set as 0.03 amu^1/2^*Bohr. Simulations were conducted at 298 K, which is conducive to promoting thermal activation and facilitating chemical reactivity within the simulated system.

## 4. Conclusions

This study presented a global route mapping of the C_3_H_2_O PES, revealing an intricate reaction network of isomerization and dissociation pathways. The identification of 30 EQs, accompanied by 128 TSs and 35 direct dissociation channels, provided unprecedented insight into the conformational landscape of the C_3_H_2_O family. The global reaction route map was established through a systematic exploration of the PES via automated and efficient ADDF calculations.

Among the identified structures, H_2_CCCO (EQ3), OC(H)CCH (EQ7), H-*c*-CC(O)C-H (EQ0), HCC(H)CO (EQ1), HO-*c*-CCC-H (EQ12) emerged as the five most stable isomers. The reaction network analysis encompassed 28 isomerization processes (EQ*x*-TS*n*-EQ*y*), 20 distinct TS-mediated dissociations (EQ*a*-TS*b*-DC), and 31 distinct direct dissociation channels (EQ*m*-DC*n*); these were systematically examined, demonstrating the complex nature of the C_3_H_2_O isomerization processes.

Our investigation of single-water-molecule collisions with selected stable isomers revealed distinct reactivity patterns: while high-energy collisions were required for EQ3 and EQ7 to form predominantly neutral products, EQ0 exhibited unique behavior in generating both ionic and neutral species. Notably, EQ1 and EQ12 demonstrated more accessible reaction pathways, requiring lower collision energies and showing a propensity for spontaneous isomerization to other stable isomers.

The MC-AFIR calculation, complemented by BOMD simulations, suggests several viable products from reactions with water molecules, including HCCC(OH)_2_H, formed by EQ7 and H_2_O, OCCHCH_2_OH, formed by EQ1 and H_2_O, and HO-*c*-CC(H)C(OH)-H, formed by EQ12 and H_2_O. These findings significantly enhance our understanding of C_3_H_2_O isomerization in gas phases and provide valuable insights into potential water-involved transformation products.

## Figures and Tables

**Table 1:** Relative ZPVE-corrected energies and energy barriers for the isomerization pathways of the form EQ*x*-TS*n*-EQ*y*, where TS*n* EQ*x*-*y* indicates the TS*n*-connecting structures EQ*x* and EQ*y* at 0 K (kcal/mol).

Pathways	EQ*x*	TS*n x*-*y*	Energy Barrier	EQ*y*	Imaginary Frequency (cm^−1^)	Pathways	EQ*x*	TS*n x*-*y*	Energy Barrier	EQ*y*	Imaginary Frequency (cm^−1^)
EQ1-TS0-EQ0	21.46	30.07	8.61	0	−815.12	EQ12-TS30-EQ14	33.98	95.91	61.93	39.36	−958.30
EQ3-TS7-EQ1	−10.56	66.38	76.94	21.46	−1678.08	EQ8-TS32-EQ12	43.38	90.90	47.52	33.98	−387.95
EQ4-TS8-EQ3	58.21	73.90	15.69	−10.56	−1001.41	EQ1-TS37-EQ12	21.46	119.47	98.01	33.98	−1710.32
EQ7-TS10-EQ1	−4.91	55.65	60.56	21.46	−1102.08	EQ14-TS41-EQ7	39.36	73.11	33.75	−4.91	−1738.97
EQ7-TS11-EQ0	−4.91	52.42	57.33	0	−1239.08	EQ7-TS42-EQ16	−4.91	71.71	76.62	70.75	−467.34
EQ7-TS13-EQ10	−4.91	72.76	77.67	40.96	−2092.46	EQ7-TS43-EQ18	−4.91	120.95	125.86	117.86	−91.47
EQ11-TS14-EQ7	36.44	37.85	1.41	−4.91	−615.25	EQ15-TS44-EQ12	55.21	152.72	97.51	33.98	−758.24
EQ7-TS15-EQ5	−4.91	39.25	44.16	38.93	−496.21	EQ2-TS49-EQ0	57.93	87.45	29.52	0	−506.28
EQ7-TS17-EQ9	−4.91	99.23	104.14	77.33	−404.69	EQ8-TS57-EQ3	43.38	87.86	44.48	−10.56	−1668.23
EQ0-TS18-EQ3	0	75.58	75.58	−10.56	−420.51	EQ7-TS70-EQ3	−4.91	74.30	79.21	−10.56	−989.34
EQ11-TS20-EQ1	36.44	52.19	15.75	21.46	−659.98	EQ6-TS74-EQ7	75.25	120.98	45.73	−4.91	−311.80
EQ12-TS22-EQ0	33.98	84.14	50.16	0	−2047.35	EQ3-TS77-EQ20	−10.56	86.05	96.61	77.45	−504.66
EQ13-TS26-EQ12	36.34	44.67	8.33	33.98	−605.25	EQ26-TS100-EQ7	118.01	119.07	1.06	−4.91	−783.66
EQ10-TS27-EQ12	40.96	85.99	45.03	33.98	−305.30	EQ17-TS103-EQ7	118.16	119.73	1.57	−4.91	−383.34

**Table 2:** Relative ZPVE-corrected energy and energy barrier (in kcal/mol) for the TS-mediated pathways in the form of EQ*a*-TS*b*-DC at 0 K, where TS*b a*-DC indicates the TS*b*-connecting structures EQ*a* and the dissociation channel.

Pathways	EQ*a*	TS*b a*-DC	Energy Barrier	Imaginary Frequency (cm^−1^)	Dissociation Process
EQ1-TS2-DC	21.46	27.77	6.31	−565.83	HCC(H)CO → CO + HCCH
EQ3-TS9-DC	−10.56	70.21	80.77	−1320.69	H_2_CCCO → H^+^ + HCCCO^−^
EQ0-TS71-DC	0	36.09	36.09	−673.89	H-*c*-CC(O)C-H → CO + HCCH
EQ7-TS82-DC	−4.91	104.78	109.69	−2028.17	OC(H)CCH → H_2_·OCCC
EQ2-TS1-DC	57.93	83.64	25.71	−865.52	H-*c*-CCOC-H → HCC(H)CO
EQ4-TS4-DC	58.21	95.03	36.82	−770.26	H_2_C-*c*-CCO → H_2_CCOC
EQ4-TS63-DC	58.21	139.28	81.07	−649.19	H_2_C-*c*-CCO → C-*c*-CCO + H_2_
EQ14-TS47-DC	39.36	123.04	83.68	−1446.83	HOC(H)CC → OH^−^ + CCCH^+^
EQ15-TS56-DC	55.21	114.48	59.27	−2265.85	HOC(H)CC → H_2_·OCCC
EQ8-TS59-DC	43.38	91.10	47.72	−2345.41	HOCCCH → COH^−^ + HCC^+^
EQ20-TS64-DC	77.45	113.69	36.24	−661.85	H_2_-*c*-COC-C → OCCCH_2_
EQ23-TS65-DC	93.09	94.85	1.76	−404.27	H_2_-*c*-CCOC → H_2_CCOC
EQ23-TS90-DC	93.09	166.52	73.43	−604.55	H_2_-*c*-CCOC → CCCO + H_2_
EQ9-TS84-DC	77.33	127.66	50.33	−1475.36	HCOCCH → H^+^ + COCCH^−^
EQ9-TS85-DC	77.33	128.06	50.73	−1053.52	HCOCCH → H^+^ + COCCH^−^
EQ9-TS104-DC	77.33	137.06	59.73	−1205.31	HCOCCH → H^+^ + COCCH^−^
EQ9-TS87-DC	77.33	123.11	45.78	−172.39	HCOCCH → H^+^ + CCOCH^−^
EQ18-TS96-DC	117.86	141.88	24.02	−452.57	HCOC(H)C → CC(H)OCH
EQ19-TS114-DC	144.21	184.23	40.02	−1220.97	HOC(C)CH → H_2_·OC(C)C
EQ21-TS123-DC	180.83	181.72	0.89	−592.26	COCCH^−^ + H^+^ → COCCH^−^ + H^+^

**Table 3:** Relative ZPVE-corrected energies and energy barriers for direct dissociation channels from EQ*m* to DC*n* at 0 K (kcal/mol).

Pathways	EQ*m*	DC*n*	Energy Barrier
EQ0-DC0	0	110.63	110.63
EQ0-DC1	0	180.71	180.71
EQ3-DC2	−10.56	93.36	103.92
EQ3-DC3	−10.56	199.99	210.55
EQ7-DC4	−4.91	88.10	93.01
EQ7-DC5	−4.91	145.64	150.55
EQ12-DC7	33.98	155.25	121.27
EQ12-DC8	33.98	123.85	89.87
EQ13-DC9	36.34	153.12	116.78
EQ13-DC10	36.34	128.06	91.72
EQ11-DC11	36.44	140.95	104.51
EQ14-DC13	39.36	157.52	118.16
EQ5-DC15	38.93	133.75	94.82
EQ10-DC16	40.96	169.29	128.33
EQ15-DC17	55.21	147.34	92.13
EQ15-DC18	55.21	159.54	104.33
EQ15-DC19	55.21	197.12	141.91
EQ8-DC21	43.38	104.85	61.47
EQ6-DC22	75.25	167.64	92.39
EQ6-DC23	75.25	224.37	149.12
EQ20-DC24	77.45	196.67	119.22
EQ20-DC25	77.45	218.81	141.36
EQ9-DC26	77.33	166.05	88.72
EQ9-DC27	77.33	224.29	146.96
EQ23-DC28	93.09	180.46	87.37
EQ24-DC29	95.42	224.61	129.19
EQ18-DC30	117.86	291.26	173.40
EQ27-DC31	153.10	237.04	83.94
EQ28-DC32	178.18	242.97	64.79
EQ29-DC33	200.29	235.39	35.10
EQ21-DC34	180.83	194.06	13.23

## Data Availability

Detailed data, including structural isomers, collision-induced reaction products, molecular coordinates, and comparative analyses across computational levels of theory, have been deposited in the Zenodo repository: https://doi.org/10.5281/zenodo.14892049.

## Supplementary Materials

The following supporting information can be downloaded at: https://www.mdpi.com/article/10.3390/molecules30081829/s1, Figure S1: Explored geometric structure of cyclopropanone (EQ0), with labeled atoms showing the three-membered carbon ring (C1, C3, C5), carbonyl oxygen (O6), and hydrogen atoms (H2, H4); Table S1: Geometric parameters of EQ0 (H-*c*-CC(O)C-H) isomer: Comparison between current exploration, experimental measurements, and theoretical calculations. Bond lengths (Å) and angles (°); Figure S2: Influence of quantum tunnelling on the reaction kinetics of pathways EQ1-TS0-EQ0, EQ2-TS49-EQ0, EQ3-TS77-EQ20, and EQ7-TS17-EQ9; Figure S3: Influence of quantum tunnelling on the reaction kinetics of pathways EQ7-TS42-EQ16, EQ8-TS32-EQ12, EQ11-TS14-EQ7, and EQ11-TS20-EQ1; Figure S4: Influence of quantum tunnelling on the reaction kinetics of pathways EQ13-TS26-EQ12, EQ17-TS103-EQ7, and EQ26-TS100-EQ7; Table S2: Relative ZPVE-corrected energies (kcal/mol) for pathways of the form EQ*x*-TS*n*-EQ*y* and EQ*a*-TS*b*-DC at 298 K, where TSn represents the transition state connecting structures EQ*x* and EQ*y*, and TS*b* represents the transition state connecting EQ*a* and dissociation channels. Imaginary frequencies (cm^−1^) of transition states and rate constants calculated using transition state theory (*k_TST_*), Wigner correction (*k_Wigner_*), and Eckart correction (*k_Eckart_*) methods are included; Figure S5: Influence of quantum tunnelling on the reaction kinetics of pathways EQ0-TS71-DC, EQ1-TS2-DC, EQ4-TS63-DC, and EQ18-TS96-DC; Figure S6: Influence of quantum tunnelling on the reaction kinetics of pathways EQ20-TS64-DC, EQ21-TS123-DC, EQ23-TS65-DC, and EQ23-TS90-DC; Figure S7: Comparative statistical analysis of energy distribution methods for EQs, presented in relative energy normalized to the EQ0 reference isomer; Figure S8: Comparative statistical analysis of energy distribution methods for TSs, presented in relative energy normalized to the EQ0 reference isomer; Figure S9: Comparative statistical analysis of energy distribution methods for DC structures, presented in relative energy normalized to the EQ0 reference isomer; Table S3: Formation ratios of potential products in the MC-AFIR calculations for the reaction between EQ3 (H_2_CCCO) + H_2_O at different artificial force constants; Figure S10: BOMD trajectory snapshots of OH^−^ + H_2_CC(H)CO^+^ system within 60 fs; Figure S11: BOMD trajectory snapshots of H_2_CCC(OH_2_)O system within 60 fs; Figure S12: BOMD trajectory snapshots of H_2_C(OH)C(H)CO system within 60 fs; Table S4: Formation ratios of potential products in the MC-AFIR calculations for the reaction between EQ7 (OC(H)CCH) + H_2_O at different artificial force constants; Figure S13: BOMD trajectory snapshots of HCCC(OH)_2_H system within 60 fs; Figure S14: BOMD trajectory snapshots of HC(O)CC(OH_2_)H system within 60 fs; Table S5: Formation ratios of potential products in the MC-AFIR calculations for the reaction between EQ0 (H-*c*-CC(O)C-H) + H_2_O at different artificial force constants; Figure S15: BOMD trajectory snapshots of H-*c*-CC(O)C(OH_2_)-H system within 60 fs; Figure S16: BOMD trajectory snapshots of OH^−^ + H-*c*-CC(OH)CH^+^ system within 60 fs; Table S6: Formation ratios of potential products in the MC-AFIR calculations for the reaction between EQ1 (HCC(H)CO) + H_2_O at different artificial force constants; Figure S17: BOMD trajectory snapshots of OCCHCH_2_OH system within 60 fs; Figure S18: BOMD trajectory snapshots of OCCHCHOH_2_ system within 60 fs; Figure S19: BOMD trajectory snapshots of HCCHC(O)OH_2_ system within 60 fs; Table S7: Formation ratios of potential products in the MC-AFIR calculations for the reaction between EQ12 (HO-*c*-CCC-H) + H_2_O at different artificial force constants; Figure S20: BOMD trajectory snapshots of HO-*c*-CCC-H + H_2_O system within 60 fs; Figure S21: BOMD trajectory snapshots of HO-*c*-CC(H)C(OH)-H system within 60 fs; Complete Pathways of C_3_H_2_O Isomerization; Complete Pathways of C_3_H_2_O Isomerization; List of Equilibrium Structures; List of Transition Structures; List of Dissociated Structures; Reaction Kinetics and Rate Constants.
