# Supplementary material for: Global Reaction Route Mapping of C3H2O: Isomerization Pathways, Dissociation Channels, and Bimolecular Reaction with a Water Molecule
Source: Molecules. 2025 Apr 18;30(8):1829. doi: 10.3390/molecules30081829 (PMC12029752; doi:10.3390/molecules30081829)
Supplement: Supplementary file 1 [file molecules-30-01829-s001.zip › molecules-3514175-supplementary.pdf]

Global Reaction Route Mapping of C_3H_2O: Isomerization Pathways, Dissociation Channels, and Bimolecular Reaction with a Water Molecule

Dapeng Zhang,[†] *Naoki Kishimoto*[*†]

[†] Department of Chemistry, Graduate School of Science, Tohoku University, 6-3, Aoba, Aramaki, Aoba-ku, Sendai 980-8578, Japan

Corresponding Author

* **E-mail**: kishimoto@tohoku.ac.jp

Supporting Information

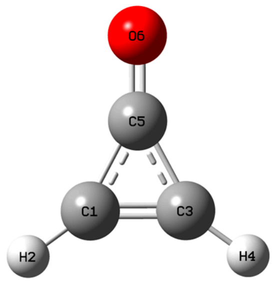

Figure S1. Explored geometric structure of cyclopropanone (EQ0), with labeled atoms showing the three -membered carbon ring (C1, C3, C5), carbonyl oxygen (O6), and hydrogen atoms (H2, H4).

Table S1. Geometric parameters of EQ0 (H-*c*-CC(O)C-H) isomer: Comparison between current exploration, experimental measurements, and theoretical calculations. Bond lengths (Å) and angles (°).

	This study	Exp1[a]	Exp2[b]	Calc1[c]	Calc2[d]	Calc3[e]	Calc4[f]	Calc5[g]	Calc6[h]	Calc7[i]
C5=O6	1.19997	1.212	1.212	1.2015	1.190	1.202	1.202	1.203	1.207	1.204
C5-C3	1.42723	1.423	1.412	1.4268	1.412	1.422	1.428	1.431	1.436	1.435
C1=C3	1.33949	1.349	1.302	1.3397	1.327	1.337	1.340	1.344	1.346	1.352
C3-H4	1.08133	1.079	1.097	1.0806	1.071	1.078	1.081	1.083	1.086	1.079
H2-C1-C5	153.92511	-	-	153.795	-	-	-	-	-	-
C1-C5-C3	55.97244	-	62.55	55.9978	-	-	62.03	61.99	62.06	62.60
H2-C1=C3	144.06123	144.30	144.92	144.2035	145.20	144.80	144.00	141.07	141.07	144.90

a: ref.1
b: ref.2
c: B3LYP/aug-cc-pVTZ, ref.3
d: MP2/6-31G*, ref.1
e: CISD/6-31G*, ref.1
f: B3LYP/cc-pVTZ, ref.4
g: B3LYP/6-311++G**, ref.4
h: B3LYP/6-31G*, ref.4
i: MP2/cc-pVTZ, ref.5

References

1. Jacobs, C. A.; Brahms, J. C.; Dailey, W. P.; Beran, K.; Harmony, M. D. Synthesis, Microwave Spectrum, and Ab Initio Calculations for Difluorocyclopropenone. *J. Am. Chem. Soc.* **1992**, *114*, 115–121. https://doi.org/10.1021/ja00027a016

2. Benson, R. C.; Flygare, W. H.; Oda, M.; Breslow, R. Microwave Spectrum, Substitutional Structure, and Stark and Zeeman Effects in Cyclopropenone. *J. Am. Chem. Soc.* **1973**, *95*, 2772–2777. https://doi.org/10.1021/ja00790a004

3. Song, X.; Wang, M.; Yang, C.; Liu, Y.; Ma, S.; Ma, X.; Pang, W. The Molecular Structure and Spectroscopic Properties of C_3H_2O and Its Isomers: An Ab Initio Study. *Spectrochim. Acta A* **2022**, *265*, 120388. https://doi.org/10.1016/j.saa.2021.120388

4. Yang, J.; McCann, K.; Laane, J. Vibrational Frequencies and Structure of Cyclopropenone from Ab Initio Calculations. *J. Mol. Struct.* **2004**, *695*, 339. https://doi.org/10.1016/j.molstruc.2003.12.046

5. Şahin, S.; Bleda, E. A.; Altun, Z.; Trindle, C. Computational Characterization of Isomeric C_4H_2O Systems: Thermochemistry, Vibrational Frequencies, and Optical Spectra for Butatrienone, Ethynyl Ketene, Butadiynol, and Triafulvenone. *Int. J. Quantum Chem.* **2016**, *116*, 444. https://doi.org/10.1002/qua.25063

Equations used in the rate calculations:

Transition state theory

$$k_{TST} = \frac{k_B T}{h} e^{\frac{-\Delta G^{\ddagger}}{k_B T}}$$

Wigner tunnelling correction

$$\kappa_{Wigner} = 1 + \frac{1}{24}(\frac{h|\nu_{TS}|}{k_B T})^2$$

$$k_{Wigner} = k_{TST} \times \kappa_{Wigner}$$

Eckart tunnelling correction

$$P(E) = \frac{cosh(a+b) - cosh(a-b)}{cos\,h(a+b) + cosh(d)}$$

$$a = \frac{4\pi\sqrt{E - E_r}}{hv}(\frac{1}{\sqrt{V_{max}}} + \frac{1}{\sqrt{V_{max} - \Delta V}})^{-1}$$

$$b = \frac{4\pi\sqrt{E - E_r - \Delta V}}{hv}(\frac{1}{\sqrt{V_{max}}} + \frac{1}{\sqrt{V_{max} - \Delta V}})^{-1}$$

$$d = 2\pi\sqrt{\frac{4V_{max}(V_{max} - \Delta V)}{(hv)^2} - \frac{1}{4}}$$

$$\kappa_{Eckart} = \frac{1}{k_B T}\int_{E_0}^{E_{max}} P(E) e^{-\frac{E - E_{TS}}{k_B T}} dE + e^{\frac{E_{TS} - E_{max}}{k_B T}}$$

$$k_{Eckart} = k_{TST} \times \kappa_{Eckart}$$

- *h*: Planck constant (6.62607015×10^{-34} J·s)

- k_B: Boltzmann constant (1.380649×10^{-23} J/K)

- N_A: Avogadro constant (6.02214076×10^{23} mol^{-1})

- *T*: Temperature (298.15 K)

- *R*: Gas constant (8.31446261815324 J/(mol·K))

- *c*: Speed of light (2.99792458×10^{10} cm/s)

- $|\nu_{TS}|$: Absolute value of the imaginary frequency of transition state (cm^{-1})

- *ΔG*: Activation free energy (kcal/mol in table, converted to J/mol for calculations)

- 1 kcal/mol = 0.0433641 eV

- 1 kcal/mol = 4184 J/mol

Figure S2. Influence of quantum tunnelling on the reaction kinetics of pathways EQ1-TS0-EQ0, EQ2-TS49-EQ0, EQ3-TS77-EQ20, and EQ7-TS17-EQ9.

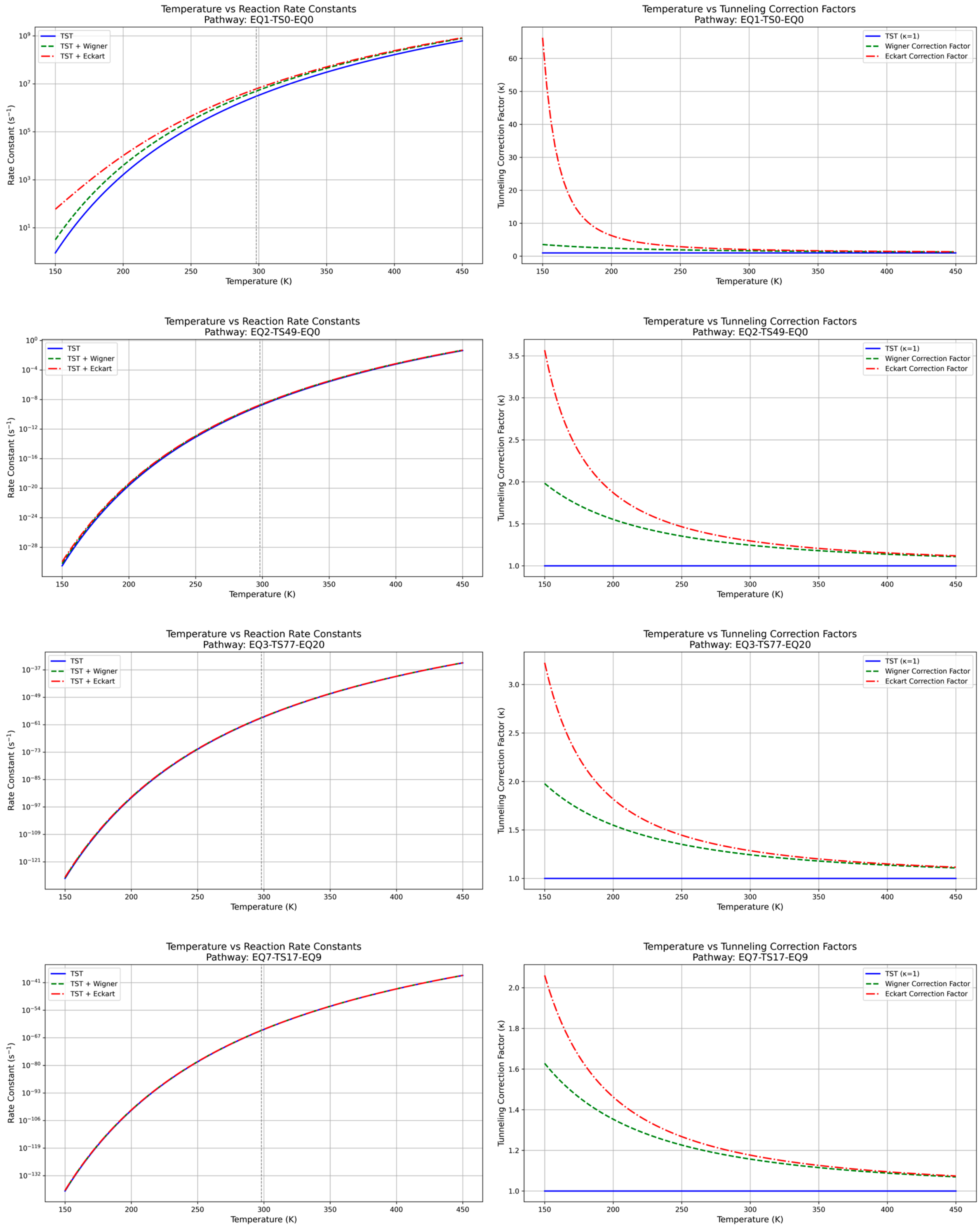

Figure S3. Influence of quantum tunnelling on the reaction kinetics of pathways EQ7-TS42-EQ16, EQ8-TS32-EQ12, EQ11-TS14-EQ7, and EQ11-TS20-EQ1.

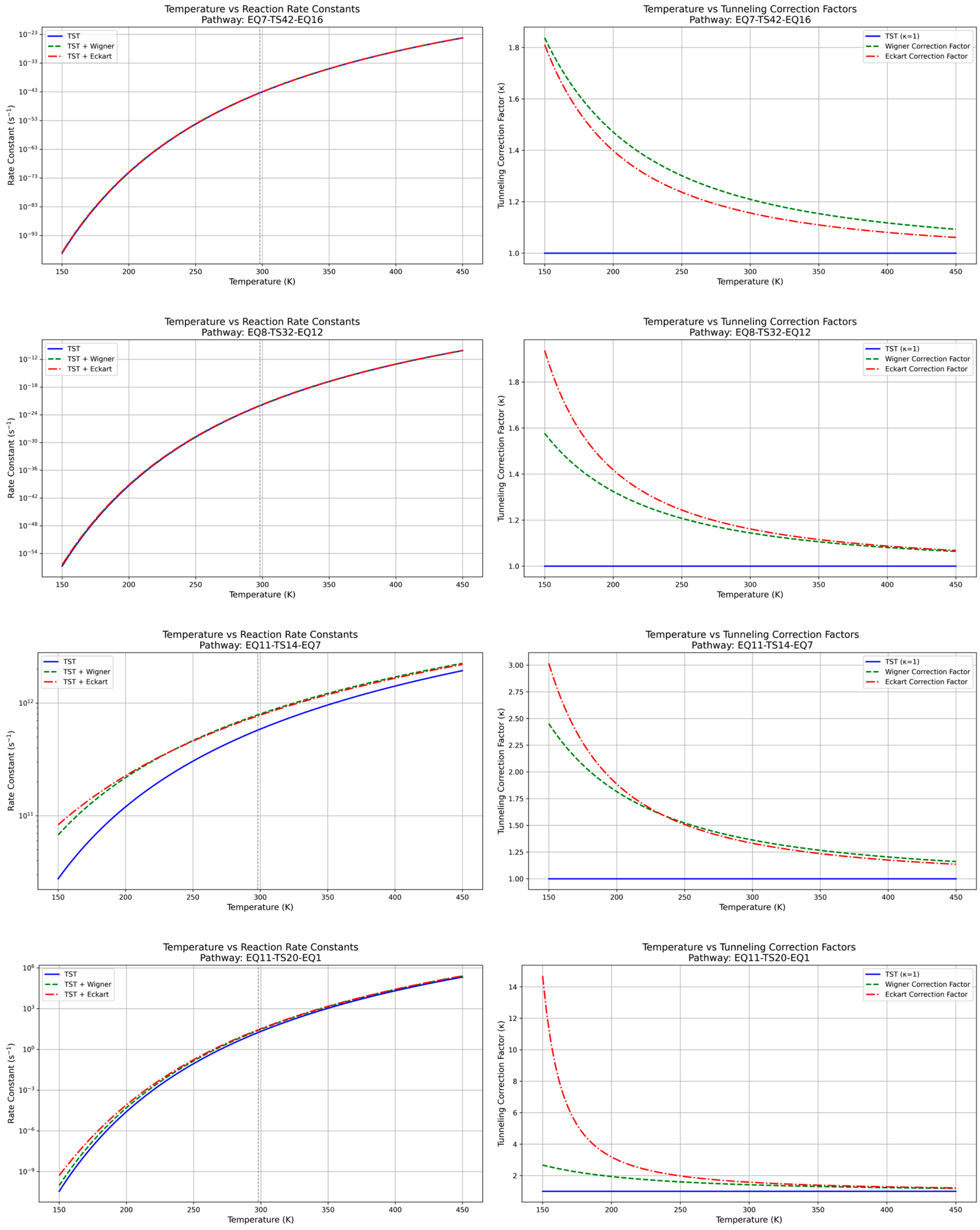

Figure S4. Influence of quantum tunnelling on the reaction kinetics of pathways EQ13-TS26-EQ12, EQ17-TS103-EQ7, and EQ26-TS100-EQ7.

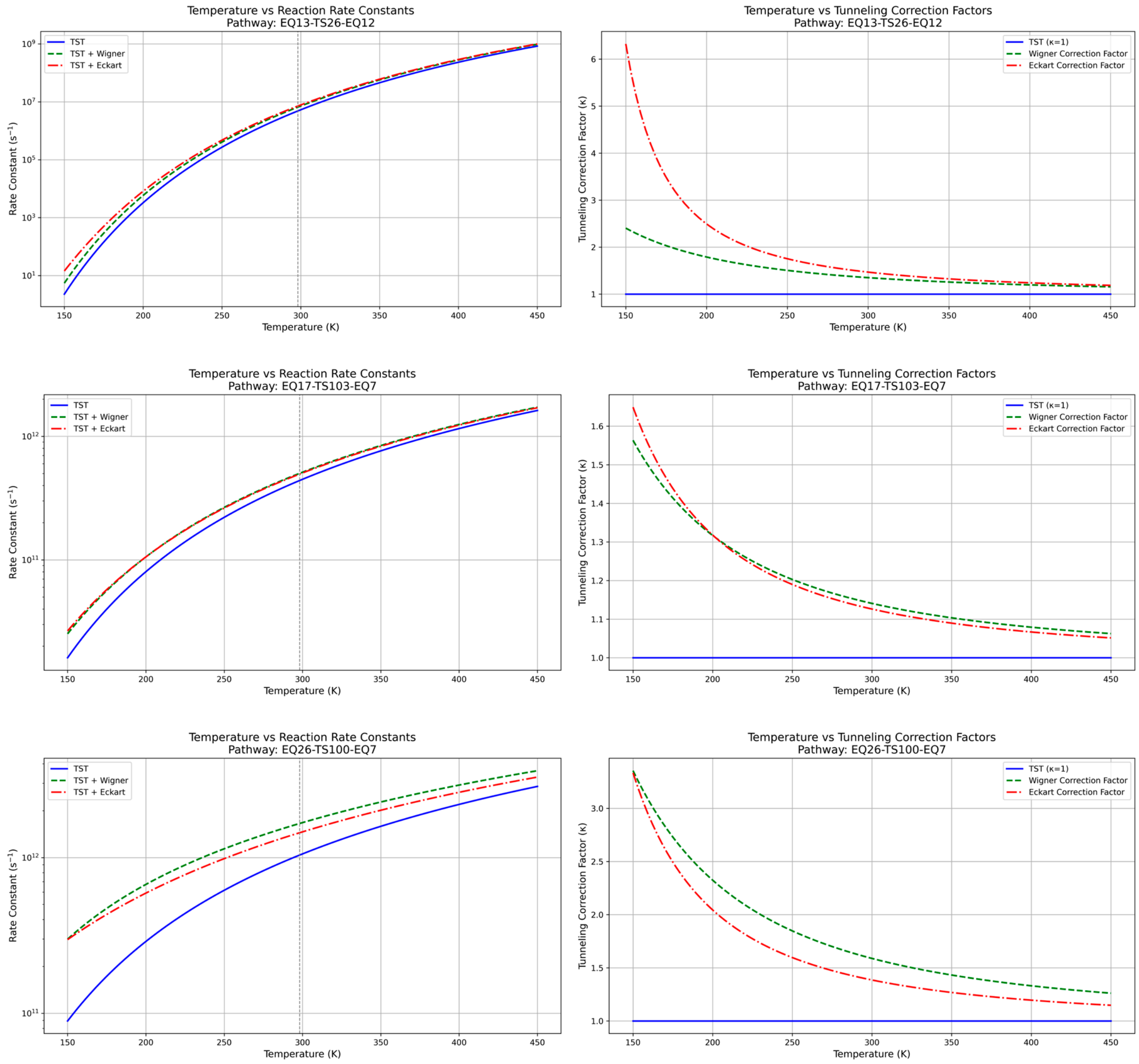

Table S2. Relative ZPVE-corrected energies (kcal/mol) for pathways of the form EQ*x*-TS*n*-EQ*y* and EQ*a*-TS*b*-DC at 298 K, where TS*n* represents the transition state connecting structures EQ*x* and EQ*y*, and TS*b* represents the transition state connecting EQ*a* and dissociation channels. Imaginary frequencies (cm^{-1}) of transition states and rate constants calculated using transition state theory (k_{TST}), Wigner correction (k_{Wigner}), and Eckart correction (k_{Eckart}) methods are included.

Pathways	EQ*x*	TS*n x-y*	EQ*y*	Imaginary Frequency (cm^{-1})	k_{TST} (s^{-1})	k_{Wigner} (s^{-1})	k_{Eckart} (s^{-1})
EQ1-TS0-EQ0	21.46	30.07	0	-815.12	3.088×10^{6}	5.074×10^{6}	6.286×10^{6}
EQ11-TS14-EQ7	36.44	37.85	-4.91	-615.25	5.773×10^{11}	7.888×10^{11}	7.714×10^{11}
EQ7-TS17-EQ9	-4.91	99.23	77.33	-404.69	3.501×10^{-64}	4.056×10^{-64}	4.127×10^{-64}
EQ11-TS20-EQ1	36.44	52.19	21.46	-659.98	1.828×10^{1}	2.598×10^{1}	2.902×10^{1}
EQ13-TS26-EQ12	36.34	44.67	33.98	-605.25	4.951×10^{6}	6.706×10^{6}	7.300×10^{6}
EQ8-TS32-EQ12	43.38	90.90	33.98	-387.95	1.001×10^{-22}	1.147×10^{-22}	1.165×10^{-22}
EQ7-TS42-EQ16	-4.91	71.71	70.75	-467.34	4.942×10^{-44}	5.987×10^{-44}	5.724×10^{-44}
EQ2-TS49-EQ0	57.93	87.45	0	-506.28	1.513×10^{-9}	1.888×10^{-9}	1.966×10^{-9}
EQ3-TS77-EQ20	-10.56	86.05	77.45	-504.66	1.142×10^{-58}	1.423×10^{-58}	1.472×10^{-58}
EQ26-TS100-EQ7	118.01	119.07	-4.91	-783.66	1.041×10^{12}	1.661×10^{12}	1.448×10^{12}
EQ17-TS103-EQ7	118.16	119.73	-4.91	-383.34	4.408×10^{11}	5.035×10^{11}	4.970×10^{11}
EQ1-TS2-DC	21.46	27.77	-	-565.83	1.492×10^{8}	1.954×10^{8}	2.100×10^{8}
EQ0-TS71-DC	0	36.09	-	-673.89	2.340×10^{-14}	3.369×10^{-14}	3.804×10^{-14}
EQ4-TS63-DC	58.21	139.28	-	-649.19	2.727×10^{-47}	3.840×10^{-47}	4.267×10^{-47}
EQ20-TS64-DC	77.45	113.69	-	-661.85	1.817×10^{-14}	2.588×10^{-14}	2.899×10^{-14}
EQ23-TS65-DC	93.09	94.85	-	-404.27	3.200×10^{11}	3.706×10^{11}	3.909×10^{11}
EQ23-TS90-DC	93.09	166.52	-	-604.55	1.070×10^{-41}	1.449×10^{-41}	1.571×10^{-41}
EQ18-TS96-DC	117.86	141.88	-	-452.57	1.610×10^{-5}	1.929×10^{-5}	1.985×10^{-5}
EQ21-TS123-DC	180.83	181.72	-	-592.26	1.387×10^{12}	1.858×10^{12}	2.141×10^{12}

Figure S5. Influence of quantum tunnelling on the reaction kinetics of pathways EQ0-TS71-DC, EQ1-TS2-DC, EQ4-TS63-DC, and EQ18-TS96-DC.

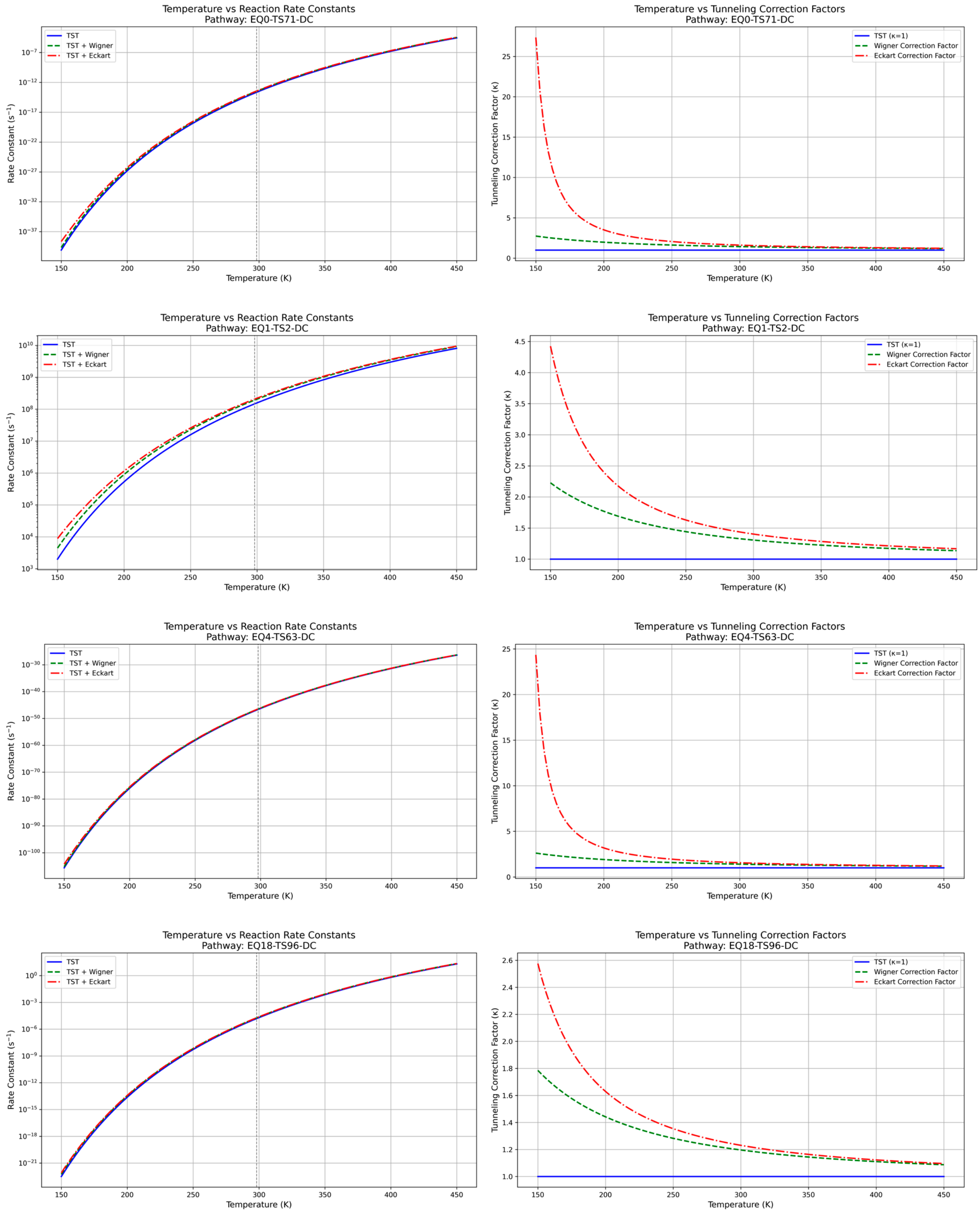

Figure S6. Influence of quantum tunnelling on the reaction kinetics of pathways EQ20-TS64-DC, EQ21-TS123-DC, EQ23-TS65-DC, and EQ23-TS90-DC.

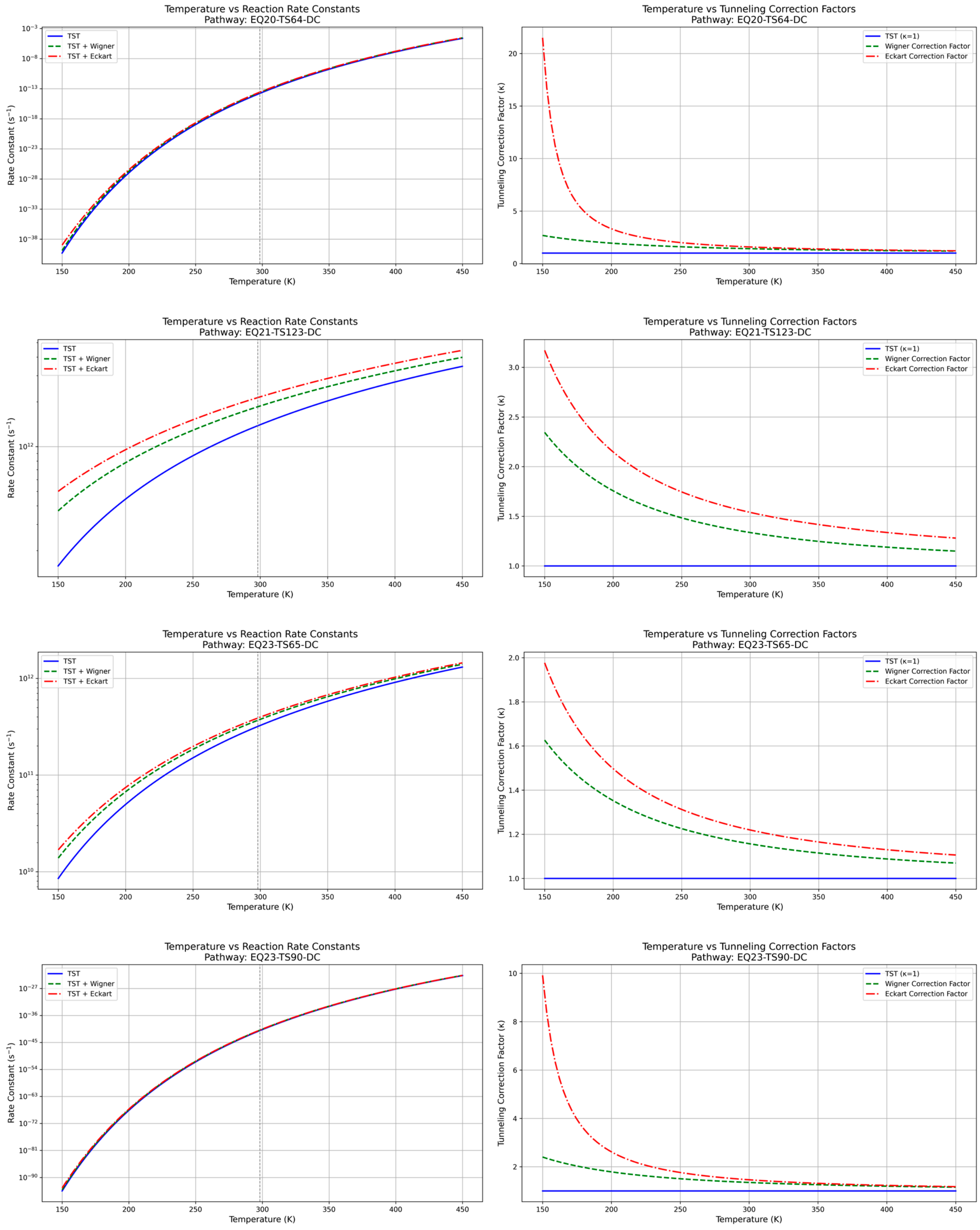

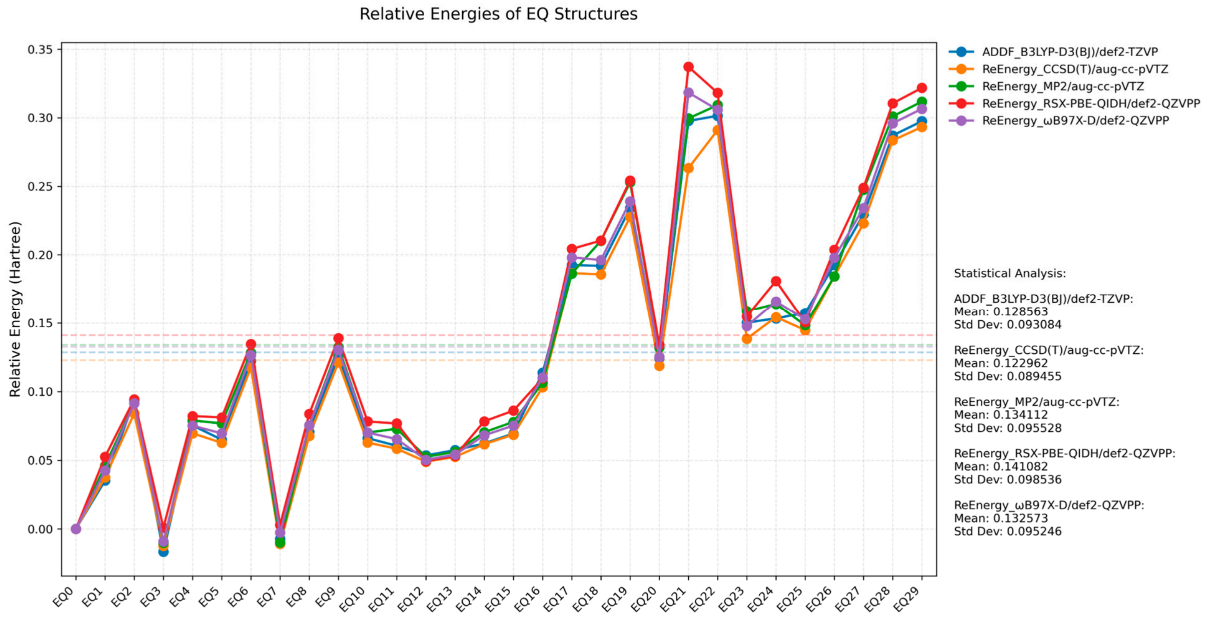

Figure S7. Comparative statistical analysis of energy distribution methods for EQ structures, presented in relative energy normalized to the EQ0 reference isomer.

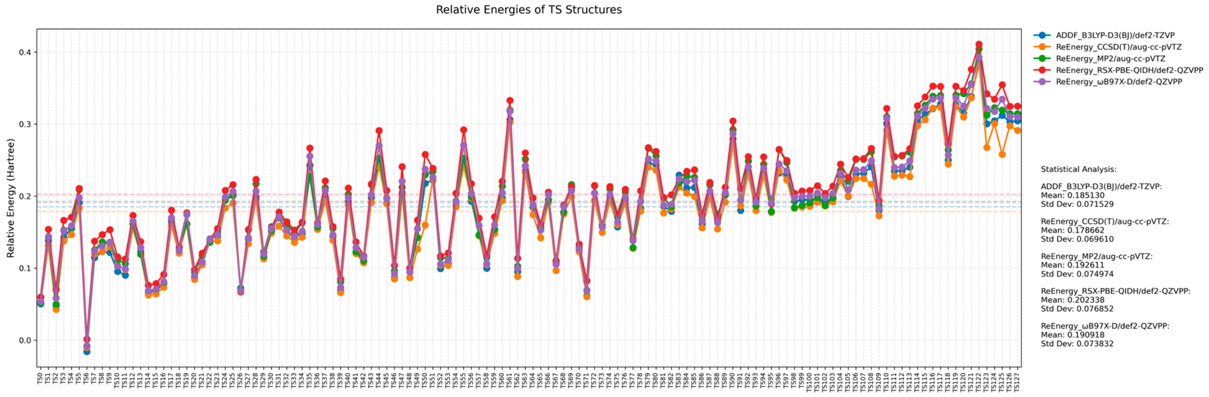

Figure S8. Comparative statistical analysis of energy distribution methods for TS structures, presented in relative energy normalized to the EQ0 reference isomer.

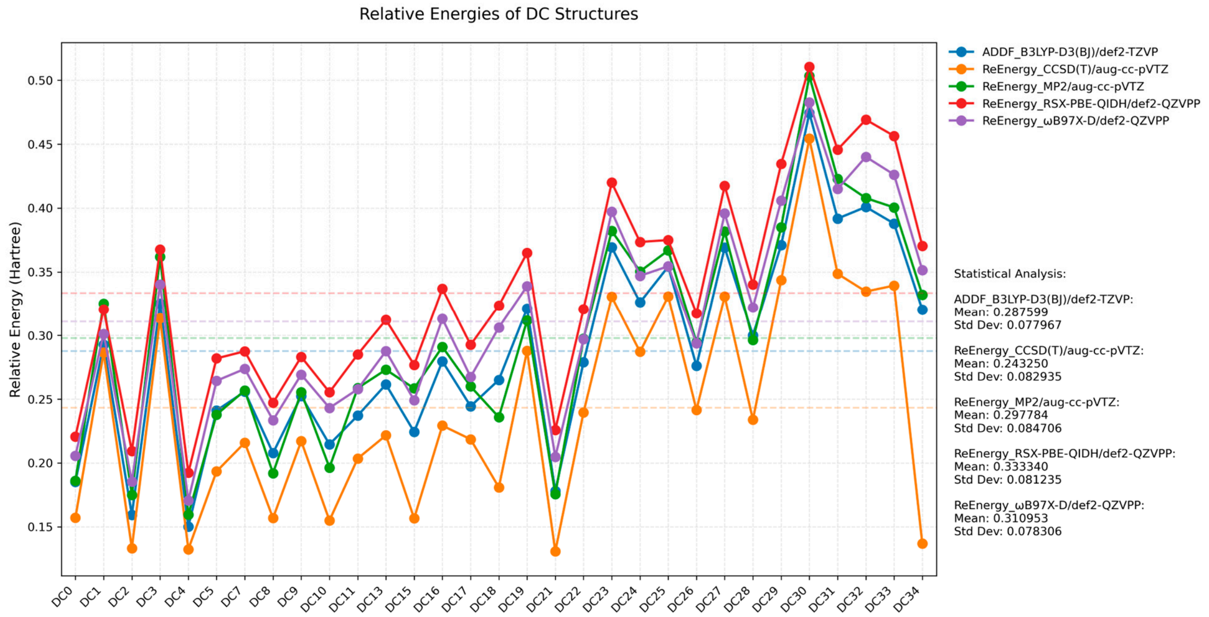

Figure S9. Comparative statistical analysis of energy distribution methods for DC structures, presented in relative energy normalized to the EQ0 reference isomer.

Table S3. Formation ratios of potential products in the MC-AFIR calculations for the reaction between EQ3 (H_2CCCO) + H_2O at different artificial force constants.

Product	Formation Ratio (100 kJ/mol)	Formation Ratio (200 kJ/mol)	Formation Ratio (400 kJ/mol)
OH^- + $H_2CC(H)CO^+$	0 %	20.00 %	15.00 %
$H_2C(OH_2)CCO$	0 %	3.00 %	0 %
$H_2CCC(OH_2)O$	0 %	0 %	44.00 %
$H_2C(OH)C(H)CO$	0 %	0 %	30.00 %
OH^- + H_2CCCOH^+	0 %	0 %	3.00 %
OH^- + $H_2CCC(H)O^+$	0 %	0 %	1.00 %
H_3O^+ + $HCCCO^-$	0 %	0 %	1.00 %
$H_3CC(OH)CO$	0 %	0 %	4.00 %

EQ3 (H_2CCCO) + H_2O

100 kJ/mol

EQ3-1, 43 EQ structures

no reaction

EQ3-2, 41 EQ structures

no reaction

200 kJ/mol

EQ3-1, 50 EQ structures

OH^- + $H_2CC(H)CO^+$, EQ2, EQ9, EQ13, EQ17, EQ18, EQ22, EQ27, EQ34, EQ35, EQ36, EQ42, EQ48, EQ49 (*13 structures*)

EQ3-2, 50 EQ structures

$H_2C(OH_2)CCO$, EQ4, EQ20, EQ37 (*3 structures*)

OH^- + $H_2CC(H)CO^+$, EQ8, EQ10, EQ12, EQ14, EQ19, EQ44, EQ49 (*7 structures*)

400 kJ/mol

EQ3-1, 50 EQ structures

$H_2CCC(OH_2)O$, EQ0, EQ1, EQ2, EQ5, EQ9, EQ11, EQ13, EQ15, EQ18, EQ21, EQ22, EQ26, EQ32, EQ33, EQ35, EQ39, EQ40, EQ43, EQ47 (*19 structures*)

$H_2C(OH)C(H)CO$, EQ4, EQ7, EQ14, EQ23, EQ27, EQ28, EQ29, EQ31, EQ34, EQ38, EQ42, EQ45, EQ48, EQ49 (*14 structures*)

$OH^- + H_2CC(H)CO^+$, EQ3, EQ8, EQ16, EQ19, EQ20, EQ24, EQ30, EQ36, EQ44 (*9 structures*)

$OH^- + H_2CCCOH^+$, EQ10, EQ37 (*2 structures*)

$OH^- + H_2CCC(H)O^+$, EQ17 (*1 structure*)

$H_3O^+ + HCCCO^-$, EQ25 (*1 structure*)

$H_3CC(OH)CO$, EQ6, EQ12, EQ46 (*3 structures*)

EQ3-2, 50 EQ structures

$H_2CCC(OH_2)O$, EQ0, EQ1, EQ2, EQ3, EQ4, EQ5, EQ10, EQ12, EQ15, EQ16, EQ17, EQ19, EQ20, EQ21, EQ22, EQ23, EQ24, EQ25, EQ26, EQ28, EQ32, EQ35, EQ38, EQ41, EQ47 (*25 structures*)

$H_2C(OH)C(H)CO$, EQ6, EQ8, EQ11, EQ14, EQ18, EQ29, EQ30, EQ31, EQ34, EQ36, EQ42, EQ43, EQ44, EQ45, EQ46, EQ48 (*16 structures*)

$OH^- + H_2CC(H)CO^+$, EQ7, EQ8, EQ13, EQ27, EQ33, EQ49 (*6 structures*)

$OH^- + H_2CCCOH^+$, EQ39 (*1 structure*)

$H_3CC(OH)CO$, EQ40 (*1 structure*)

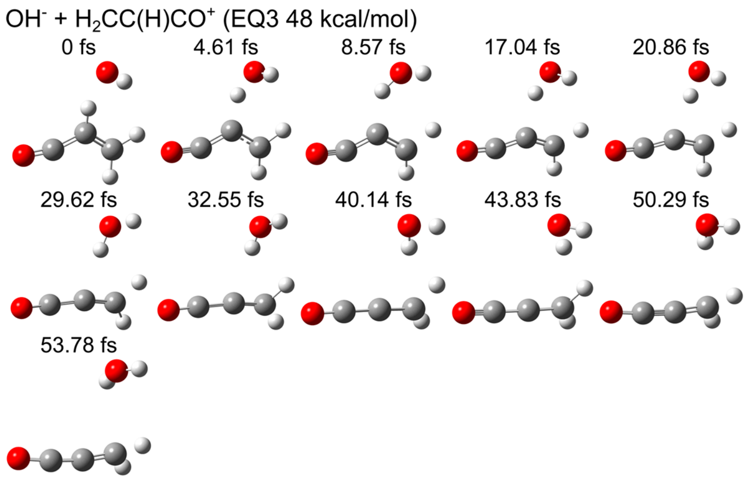

Figure S10. BOMD trajectory snapshots of OH^- + $H_2CC(H)CO^+$ system within 60 fs.

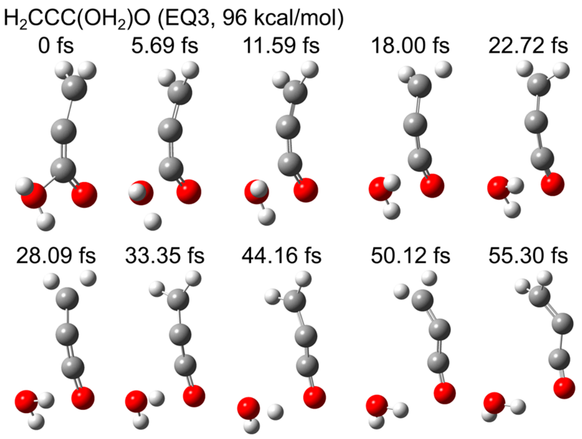

Figure S11. BOMD trajectory snapshots of $H_2CCC(OH_2)O$ system within 60 fs.

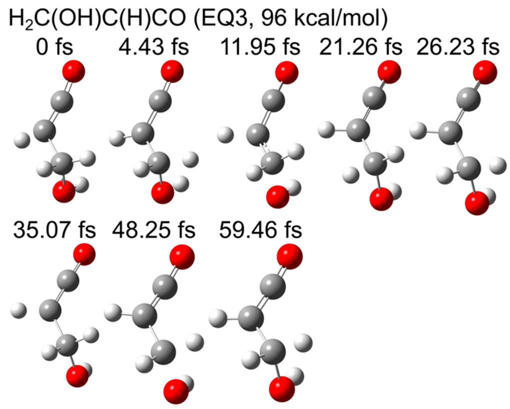

Figure S12. BOMD trajectory snapshots of $H_2C(OH)C(H)CO$ system within 60 fs.

Table S4. Formation ratios of potential products in the MC-AFIR calculations for the reaction between EQ7 (OC(H)CCH) + H_2O at different artificial force constants.

Product	Formation Ratio (100 kJ/mol)	Formation Ratio (200 kJ/mol)	Formation Ratio (400 kJ/mol)
HCC(OH)C(H)OH	0 %	8.42 %	9.00 %
H_3O^+ + CCC(O)H^-	0 %	2.11 %	3.00 %
HCCC$(OH)_2$H	0 %	0 %	33.00 %
HC(O)CC(OH_2)H	0 %	0 %	19.00 %
HC(O)C(OH_2)CH	0 %	0 %	5.00 %
OH^- + HCC(H)C(O)H^+	0 %	0 %	10.00 %
OH^- + H_2CCC(O)H^+	0 %	0 %	2.00 %
OH^- + HCCC(H)OH^+	0 %	0 %	13.00 %
HC(OH)CC(OH)H	0 %	0 %	1.00 %
H_3O^+ + HCCC$(O)^-$	0 %	0 %	1.00 %

EQ7 (OC(H)CCH) + H_2O

100 kJ/mol

EQ7-1, 43 EQ structures

no reaction

EQ7-2, 33 EQ structures

no reaction

200 kJ/mol

EQ7-1, 48 EQ structures

HCC(OH)C(H)OH, EQ3, EQ22, EQ29, EQ38 (4 *structures*)

H_3O^+ + CCC(O)H^-, EQ8, EQ23 (2 *structures*)

EQ7-2, 47 EQ structures

HCC(OH)C(H)OH, EQ4, EQ11, EQ36, EQ44 (4 *structures*)

400 kJ/mol

EQ7-1, 50 EQ structures

HCCC$(OH)_2$H, EQ0, EQ1, EQ11, EQ12, EQ13, EQ18, EQ24, EQ28, EQ29, EQ30, EQ32, EQ35, EQ43, EQ44, EQ45 (15 *structures*)

$HC(O)CC(OH_2)H$, EQ2, EQ5, EQ8, EQ17, EQ20, EQ21, EQ23, EQ25, EQ39, EQ42 (10 *structures*)

HCC(OH)C(H)OH, EQ4, EQ10, EQ19, EQ34, EQ47 (5 *structures*)

$HC(O)C(OH_2)CH$, EQ27, EQ31, EQ37 (3 *structures*)

$OH^- + HCC(H)C(O)H^+$, EQ3, EQ6, EQ16, EQ22, EQ26, EQ41, EQ46, EQ49 (8 *structures*)

$OH^- + H_2CCC(O)H^+$, EQ14 (1 *structure*)

$OH^- + HCCC(H)OH^+$, EQ7, EQ9, EQ36, EQ38, EQ48 (5 *structures*)

$H_3O^+ + CCC(O)H^-$, EQ33 (1 *structure*)

EQ7-2, 50 EQ structures

$HCCC(OH)_2H$, EQ1, EQ2, EQ9, EQ10, EQ14, EQ16, EQ18, EQ22, EQ23, EQ28, EQ31, EQ34, EQ36, EQ38, EQ39, EQ44, EQ46, EQ48 (18 *structures*)

$HC(O)CC(OH_2)H$, EQ6, EQ12, EQ13, EQ15, EQ27, EQ30, EQ32, EQ37, EQ40 (9 *structures*)

HCC(OH)C(H)OH, EQ4, EQ17, EQ29, EQ49 (4 *structures*)

$HC(O)C(OH_2)CH$, EQ5, EQ21 (2 *structures*)

$OH^- + H_2CCC(O)H^+$, EQ19 (1 *structure*)

HC(OH)CC(OH)H, EQ42 (1 *structure*)

$OH^- + HCCC(H)OH^+$, EQ7, EQ20, EQ24, EQ26, EQ33, EQ35, EQ41, EQ43 (8 *structures*)

$OH^- + HCC(H)C(O)H^+$, EQ8, EQ11 (2 *structures*)

$H_3O^+ + HCCC(O)^-$, EQ0 (1 *structure*)

$H_3O^+ + CCC(O)H^-$, EQ45, EQ47 (2 *structures*)

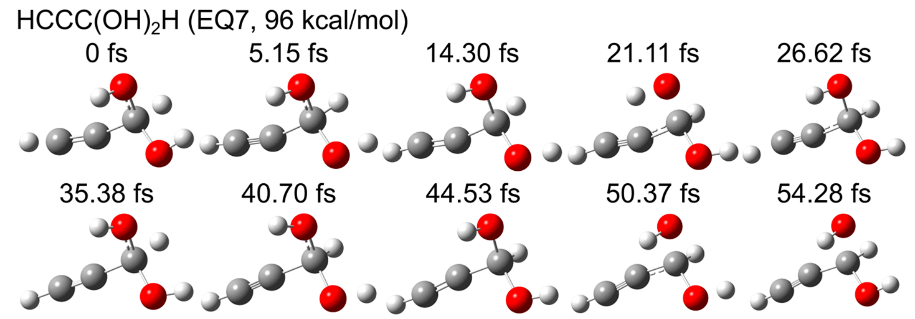

Figure S13. BOMD trajectory snapshots of HCCC(OH)$_2$H system within 60 fs.

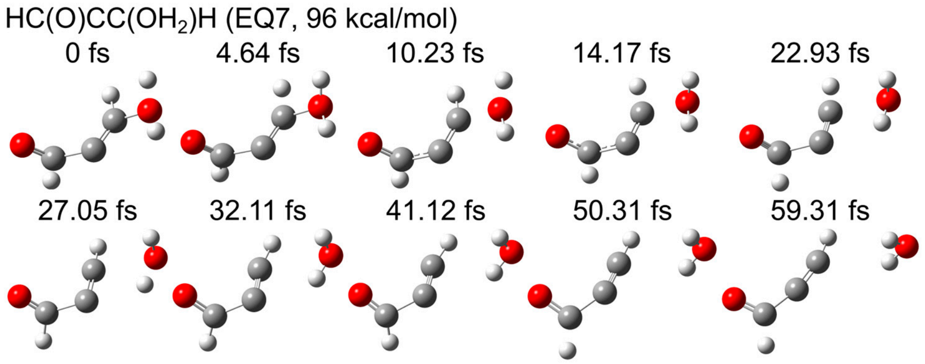

Figure S14. BOMD trajectory snapshots of HC(O)CC(OH$_2$)H system within 60 fs.

Table S5. Formation ratios of potential products in the MC-AFIR calculations for the reaction between EQ0 (H-*c*-CC(O)C-H) + H_2O at different artificial force constants.

Product	Formation Ratio (100 kJ/mol)	Formation Ratio (200 kJ/mol)	Formation Ratio (400 kJ/mol)
H_3O^+ + H-*c*-CC(O)C^-	0 %	2.11 %	6.06 %
OH^- + H-*c*-CC(OH)CH^+	0 %	0 %	33.33 %
H-*c*-CC(O)C(OH_2)-H	0 %	0 %	42.42 %
H-*c*-CC$(OH)_2$C-H	0 %	0 %	14.14 %
OH^- + H_2CC(H)CO^+	0 %	0 %	2.02 %
H_2C(OH)C(H)CO	0 %	0 %	2.02 %

EQ0 (H-*c*-CC(O)C-H) + H_2O

100 kJ/mol

EQ0-1, 48 EQ structures

no reaction

EQ0-2, 50 EQ structures

no reaction

200 kJ/mol

EQ0-1, 46 EQ structures

no reaction

EQ0-2, 49 EQ structures

H_3O^+ + H-*c*-CC(O)C^-, EQ27, EQ35, (2 *structures*)

400 kJ/mol

EQ0-1, 50 EQ structures

OH^- + H-*c*-CC(OH)CH^+, EQ0, EQ2, EQ4, EQ5, EQ6, EQ8, EQ10, EQ15, EQ16, EQ18, EQ22, EQ36, EQ37, EQ38, EQ43 (15 *structures*)

H-*c*-CC(O)C(OH_2)-H, EQ1, EQ3, EQ7, EQ11, EQ14, EQ20, EQ23, EQ25, EQ26, EQ27, EQ29, EQ31, EQ32, EQ33, EQ34, EQ39, EQ40, EQ41, EQ45, EQ46, EQ47, EQ48, EQ49 (23 *structures*)

H-*c*-CC$(OH)_2$C-H, EQ9, EQ17, EQ21, EQ24, EQ28, EQ35, EQ42 (7 *structures*)

H_3O^+ + H-*c*-CC(O)C^-, EQ12, EQ30 (2 *structures*)

OH^- + $H_2CC(H)CO^+$, EQ13, EQ19 (2 *structures*)

$H_2C(OH)C(H)CO$, EQ44 (1 *structure*)

EQ0-2, 49 EQ structures

OH^- + H-*c*-CC(OH)CH^+, EQ0, EQ1, EQ2, EQ8, EQ11, EQ16, EQ18, EQ19, EQ20, EQ24, EQ26, EQ28, EQ31, EQ37, EQ43, EQ44, EQ46, EQ47 (18 *structures*)

H-*c*-CC(O)C(OH_2)-H, EQ4, EQ5, EQ6, EQ7, EQ9, EQ12, EQ13, EQ14, EQ21, EQ23, EQ32, EQ33, EQ34, EQ36, EQ39, EQ40, EQ42, EQ45, EQ48 (19 *structures*)

H-*c*-CC$(OH)_2$C-H, EQ15, EQ17, EQ22, EQ27, EQ28, EQ38, EQ41 (7 *structures*)

H_3O^+ + H-*c*-CC(O)C^-, EQ10, EQ25, EQ30, EQ35 (4 *structures*)

$H_2C(OH)C(H)CO$, EQ3 (1 *structure*)

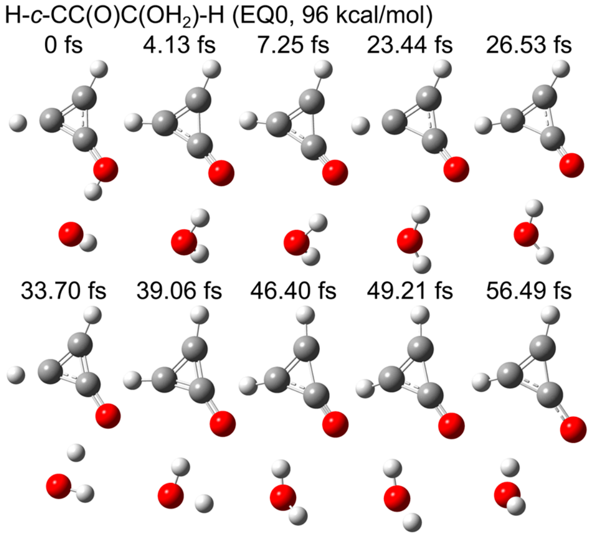

Figure S15. BOMD trajectory snapshots of H-*c*-CC(O)C(OH_2)-H system within 60 fs.

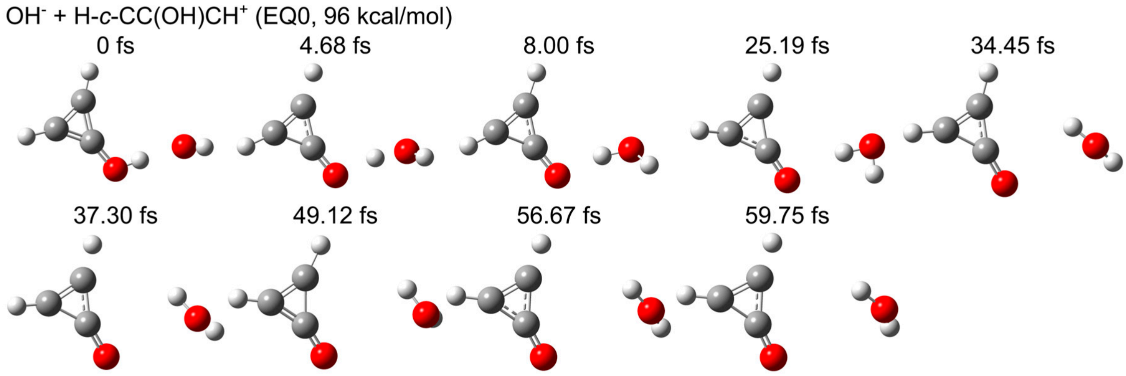

Figure S16. BOMD trajectory snapshots of OH^- + H-*c*-CC(OH)CH^+ system within 60 fs.

Table S6. Formation ratios of potential products in the MC-AFIR calculations for the reaction between EQ1 (HCC(H)CO) + H_2O at different artificial force constants.

Product	Formation Ratio (100 kJ/mol)	Formation Ratio (200 kJ/mol)	Formation Ratio (400 kJ/mol)
$OCCHCH_2OH$	43.33 %	21.43 %	24.24 %
$OCCHCHOH_2$	0 %	18.37 %	10.10 %
H_2CCCO (the same as explored EQ3) + H_2O	0 %	1.02 %	0 %
OH^- + $OCCHCH_2^+$	0 %	3.06 %	8.08 %
$HCCHC(O)OH_2$	0 %	0 %	41.41 %
OH^- + HCO^+ + HCCH	0 %	0 %	1.01 %
$H_2CCHC(O)OH$	0 %	2.04 %	2.02 %
HOCHCHCOH	0 %	1.02 %	0 %

EQ1 (HCC(H)CO) + H_2O

100 kJ/mol

EQ1-1, 44 EQ structures

$OCCHCH_2OH$, EQ5, EQ7, EQ8, EQ14, EQ17, EQ18, EQ19, EQ21, EQ22, EQ25, EQ29, EQ30, EQ31, EQ32, EQ33, EQ34, EQ39 (17 *structures*)

EQ1-2, 46 EQ structures

$OCCHCH_2OH$, EQ2, EQ3, EQ8, EQ9, EQ12, EQ14, EQ18, EQ20, EQ24, EQ25, EQ26, EQ27, EQ29, EQ30, EQ32, EQ35, EQ37, EQ38, EQ40, EQ41, EQ42, EQ45 (22 *structures*)

200 kJ/mol

EQ1-1, 49 EQ structures

$OCCHCH_2OH$, EQ5, EQ7, EQ9, EQ15, EQ18, EQ19, EQ21, EQ31, EQ33, EQ36, EQ39, EQ40, EQ44, EQ45 (14 *structures*)

$OCCHCHOH_2$, EQ1, EQ3, EQ6, EQ10, EQ25, EQ26, EQ27, EQ32 (8 *structures*)

H_2CCCO (the same as explored EQ3) + H_2O, EQ30 (1 *structure*)

OH^- + $OCCHCH_2^+$, EQ34 (1 *structure*)

EQ1-2, 49 EQ structures

$OCCHCH_2OH$, EQ4, EQ5, EQ7, EQ12, EQ17, EQ23, EQ35 (7 *structures*)

$OCCHCHOH_2$, EQ0, EQ11, EQ16, EQ18, EQ20, EQ24, EQ30, EQ33, EQ34, EQ40 (10 *structures*)

OH^- + $OCCHCH_2^+$, EQ29, EQ31 (2 *structures*)

$H_2CCHC(O)OH$, EQ42, EQ44 (2 *structures*)

HOCHCHCOH, EQ15 (1 *structure*)

400 kJ/mol

EQ1-1, 50 EQ structures

$OCCHCH_2OH$, EQ0, EQ1, EQ2, EQ14, EQ18, EQ27, EQ28, EQ34, EQ35, EQ37, EQ39, EQ41, EQ47, EQ49 (14 *structures*)

$OCCHCHOH_2$, EQ8, EQ9, EQ12, EQ15, EQ32, EQ40, EQ42 (7 *structures*)

OH^- + $OCCHCH_2^+$, EQ3, EQ16, EQ21 (3 *structures*)

$HCCHC(O)OH_2$, EQ4, EQ5, EQ7, EQ13, EQ17, EQ19, EQ20, EQ23, EQ24, EQ26, EQ30, EQ33, EQ36, EQ38, EQ43, EQ44, EQ45, EQ46, EQ48 (19 *structures*)

OH^- + HCO^+ + HCCH, EQ22 (1 *structure*)

EQ1-2, 49 EQ structures

$OCCHCH_2OH$, EQ3, EQ7, EQ10, EQ13, EQ22, EQ29, EQ31, EQ38, EQ41, EQ45 (10 *structures*)

$OCCHCHOH_2$, EQ1, EQ17, EQ44 (3 *structures*)

OH^- + $OCCHCH_2^+$, EQ25, EQ32, EQ43, EQ46, EQ48 (5 *structures*)

$HCCHC(O)OH_2$, EQ0, EQ2, EQ5, EQ6, EQ8, EQ9, EQ11, EQ12, EQ14, EQ15, EQ16, EQ18, EQ19, EQ21, EQ24, EQ26, EQ30, EQ36, EQ37, EQ39, EQ40, EQ42 (22 *structures*)

$H_2CCHC(O)OH$, EQ23, EQ34 (2 *structures*)

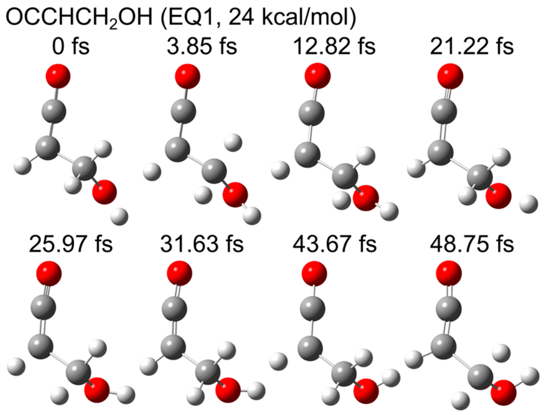

Figure S17. BOMD trajectory snapshots of OCCHCH$_2$OH system within 60 fs.

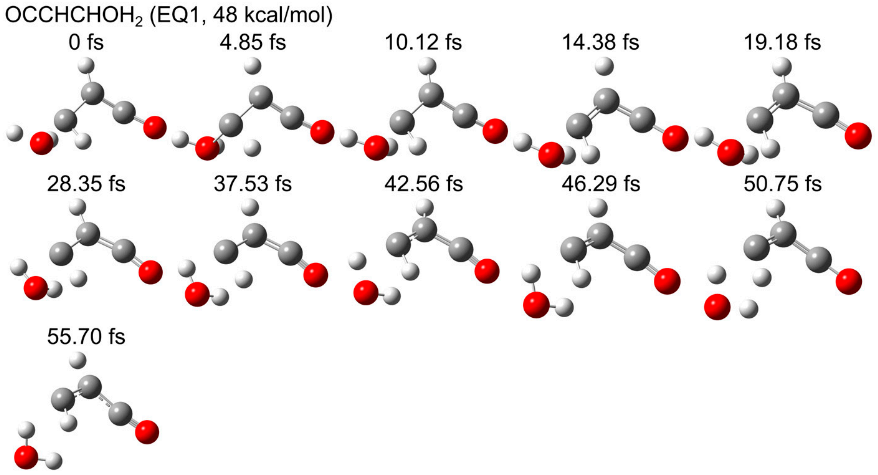

Figure S18. BOMD trajectory snapshots of OCCHCHOH$_2$ system within 60 fs.

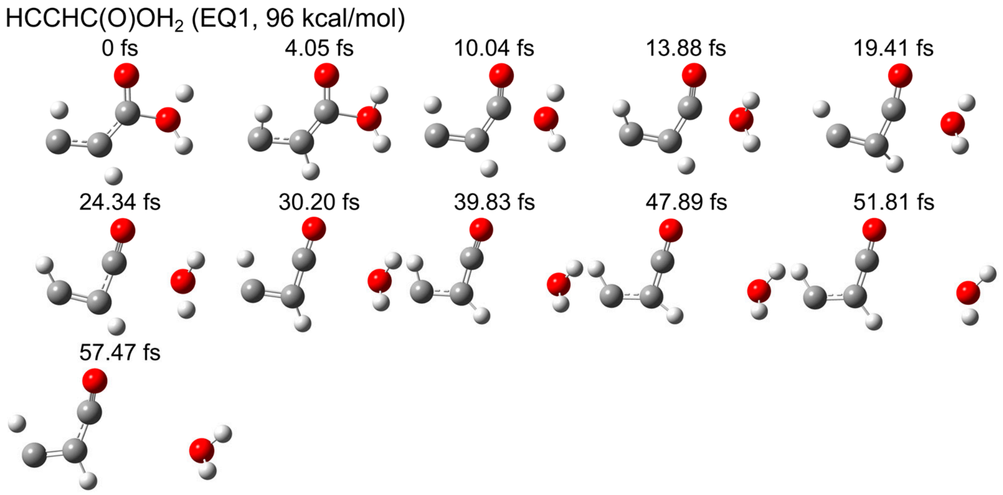

Figure S19. BOMD trajectory snapshots of HCCHC(O)OH$_2$ system within 60 fs.

Table S7. Formation ratios of potential products in the MC-AFIR calculations for the reaction between EQ12 (HO-*c*-CCC-H) + H_2O at different artificial force constants.

Product	Formation Ratio (100 kJ/mol)	Formation Ratio (200 kJ/mol)	Formation Ratio (400 kJ/mol)
H-*c*-CC(O)C-H (the same as explored EQ0) + H_2O	0 %	50.00 %	48.00 %
HO-*c*-CC(H)C(OH)-H	0 %	33.00 %	18.00 %
OH^- + H-*c*-CC(OH)CH^+	0 %	2.00 %	0 %
H-*c*-CCC(OH_2)-OH	0 %	1.00 %	4.00 %
H-*c*-C(OH_2)CC-OH	0 %	0 %	19.00 %
CC(H)C(H)$(OH)_2$	0 %	0 %	1.00 %
OH^- + H-*c*-CCC$(OH_2)^+$	0 %	0 %	1.00 %
H_3O^+ + HO-*c*-CCC^-	0 %	0 %	1.00 %
HO-*c*-CC(H)C(OH_2)	0 %	0 %	6.00 %
OH^- + H_2-*c*-CCC$(OH)^+$	0 %	0 %	2.00 %

EQ12 (HO-*c*-CCC-H) + H_2O

100 kJ/mol

EQ12-1, 50 EQ structures

no reaction

EQ12-2, 50 EQ structures

no reaction

200 kJ/mol

EQ12-1, 50 EQ structures

H-*c*-CC(O)C-H (the same as explored EQ0) + H_2O, EQ0, EQ2, EQ3, EQ6, EQ7, EQ8, EQ10, EQ13, EQ17, EQ19, EQ20, EQ21, EQ27, EQ30, EQ32, EQ33, EQ38, EQ39, EQ40, EQ41, EQ43, EQ44, EQ45, EQ46, EQ47, EQ48 (26 *structures*)

HO-*c*-CC(H)C(OH)-H, EQ1, EQ4, EQ9, EQ11, EQ14, EQ15, EQ16, EQ22, EQ23, EQ29, EQ24, EQ31, EQ34, EQ36, EQ49 (15 *structures*)

OH^- + H-*c*-CC(OH)CH^+, EQ5, EQ26 (2 *structures*)

H-*c*-CCC(OH_2)-OH, EQ18 (1 *structure*)

EQ12-2, 50 EQ structures

H-*c*-CC(O)C-H + H_2O, EQ2, EQ4, EQ7, EQ10, EQ11, EQ15, EQ17, EQ19, EQ20, EQ23, EQ24, EQ26, EQ27, EQ28, EQ29, EQ30, EQ31, EQ32, EQ37, EQ41, EQ43, EQ44, EQ47, EQ48 (24 *structures*)

HO-*c*-CC(H)C(OH)-H, EQ1, EQ3, EQ5, EQ8, EQ13, EQ14, EQ16, EQ18, EQ21, EQ22, EQ25, EQ34, EQ35, EQ36, EQ38, EQ40, EQ45, EQ49 (18 *structures*)

400 kJ/mol

EQ12-1, 50 EQ structures

H-*c*-CC(O)C-H + H_2O, EQ0, EQ2, EQ3, EQ6, EQ8, EQ12, EQ14, EQ15, EQ17, EQ18, EQ21, EQ22, EQ28, EQ30, EQ31, EQ34, EQ35, EQ38, EQ39, EQ40, EQ42, EQ43, EQ44, EQ46, EQ48, EQ49 (26 *structures*)

HO-*c*-CC(H)C(OH)-H, EQ9, EQ10, EQ11, EQ16, EQ19, EQ24, EQ25, EQ29, EQ45 (9 *structures*)

H-*c*-CCC(OH_2)-OH, EQ7, EQ27, EQ41 (3 *structures*)

H-*c*-C(OH_2)CC-OH, EQ1, EQ4, EQ13, EQ20, EQ23, EQ26, EQ32, EQ36, EQ47 (9 *structures*)

CC(H)C(H)$(OH)_2$, EQ5 (1 *structure*)

OH^- + H-*c*-CCC$(OH_2)^+$, EQ33 (1 *structure*)

H_3O^+ + HO-*c*-CCC$^-$, EQ37 (1 *structure*)

EQ12-2, 50 EQ structures

H-*c*-CC(O)C-H + H_2O, EQ0, EQ1, EQ2, EQ4, EQ5, EQ7, EQ11, EQ12, EQ20, EQ23, EQ24, EQ31, EQ35, EQ37, EQ39, EQ43, EQ44, EQ45, EQ46, EQ47, EQ48, EQ49 (22 *structures*)

HO-*c*-CC(H)C(OH)-H, EQ3, EQ9, EQ14, EQ16, EQ18, EQ21, EQ26, EQ38, EQ40 (9 *structures*)

H-*c*-CCC(OH_2)-OH, EQ42 (1 *structure*)

H-*c*-C(OH_2)CC-OH, EQ8, EQ15, EQ17, EQ19, EQ25, EQ29, EQ30, EQ33, EQ34, EQ41 (10 *structures*)

HO-*c*-CC(H)C(OH_2), EQ6, EQ13, EQ22, EQ28, EQ32, EQ36 (6 *structures*)

OH^- + H_2-*c*-CCC$(OH)^+$, EQ10, EQ27 (2 *structures*)

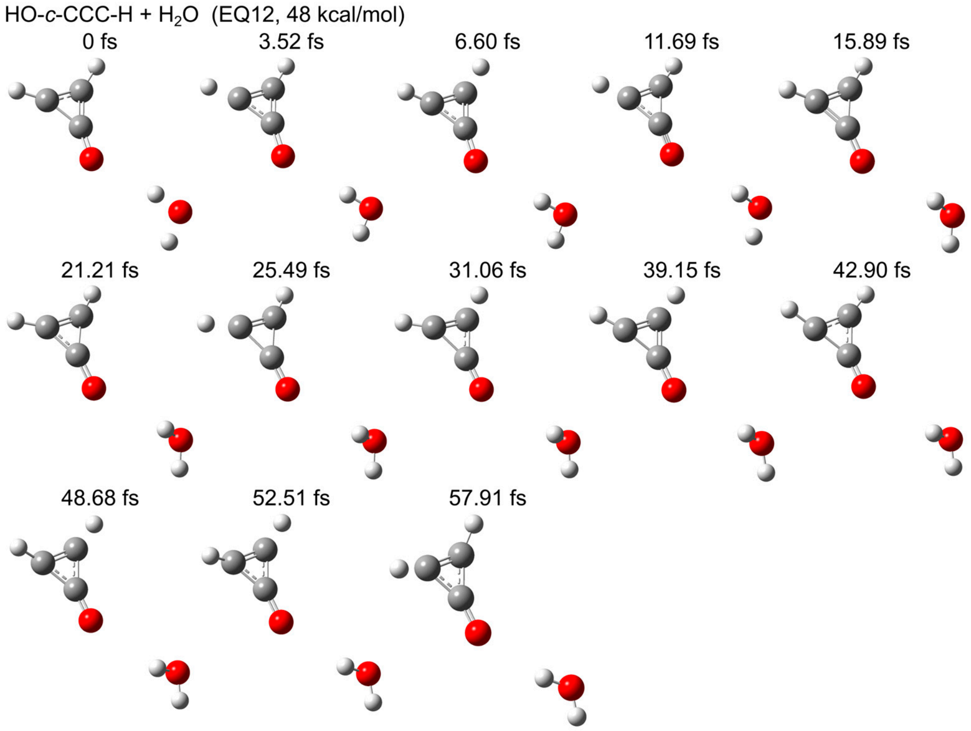

Figure S20. BOMD trajectory snapshots of HO-*c*-CCC-H + H_2O system within 60 fs.

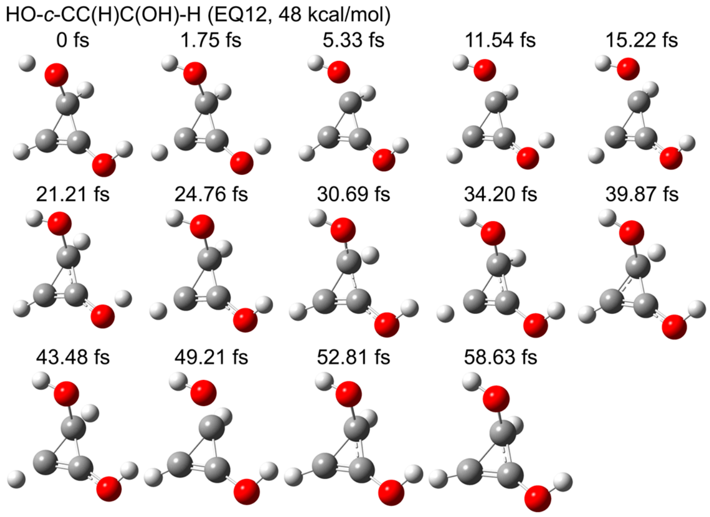

Figure S21. BOMD trajectory snapshots of HO-*c*-CC(H)C(OH)-H system within 60 fs.

Complete Pathways of C_3H_2O Isomerization

Pathways	Explored EQ*x*	Explored TS*n x-y*	Explored EQ*y*	Pathways	Explored EQ*x*	Explored TS*n x-y*	Explored EQ*y*
EQ1-TS0-EQ0	21.46	30.07	0	EQ20-TS50-EQ11	77.45	132.88	36.44
EQ4-TS3-EQ3	58.21	87.73	-10.56	EQ9-TS51-EQ6	77.33	136.70	75.25
EQ6-TS5-EQ5	75.25	116.97	38.93	EQ10-TS52-EQ8	40.96	60.39	43.38
EQ3-TS7-EQ1	-10.56	66.38	21.46	EQ8-TS53-EQ10	43.38	64.14	40.96
EQ4-TS8-EQ3	58.21	73.90	-10.56	EQ10-TS54-EQ15	40.96	113.02	55.21
EQ7-TS10-EQ1	-4.91	55.65	21.46	EQ15-TS55-EQ12	55.21	152.84	33.98
EQ7-TS11-EQ0	-4.91	52.42	0	EQ8-TS57-EQ3	43.38	87.86	-10.56
EQ2-TS12-EQ9	57.93	98.96	77.33	EQ10-TS58-EQ8	40.96	60.80	43.38
EQ7-TS13-EQ10	-4.91	72.76	40.96	EQ10-TS60-EQ13	40.96	120.64	36.34
EQ11-TS14-EQ7	36.44	37.85	-4.91	EQ2-TS66-EQ20	57.93	123.40	77.45
EQ7-TS15-EQ5	-4.91	39.25	38.93	EQ2-TS68-EQ25	57.93	114.52	97.58
EQ11-TS16-EQ7	36.44	47.30	-4.91	EQ2-TS69-EQ23	57.93	127.53	93.09
EQ7-TS17-EQ9	-4.91	99.23	77.33	EQ7-TS70-EQ3	-4.91	74.30	-10.56
EQ0-TS18-EQ3	0	75.58	-10.56	EQ25-TS73-EQ16	97.58	99.10	70.75
EQ11-TS20-EQ1	36.44	52.19	21.46	EQ6-TS74-EQ7	75.25	120.98	-4.91
EQ1-TS21-EQ0	21.46	62.64	0	EQ6-TS75-EQ9	75.25	95.33	77.33
EQ12-TS22-EQ0	33.98	84.14	0	EQ18-TS76-EQ6	117.86	118.70	75.25
EQ12-TS23-EQ8	33.98	88.51	43.38	EQ3-TS77-EQ20	-10.56	86.05	77.45
EQ15-TS24-EQ10	55.21	119.94	40.96	EQ20-TS78-EQ24	77.45	113.14	95.42
EQ8-TS25-EQ14	43.38	124.59	39.36	EQ5-TS79-EQ20	38.93	148.89	77.45
EQ13-TS26-EQ12	36.34	44.67	33.98	EQ25-TS83-EQ20	97.58	139.82	77.45
EQ10-TS27-EQ12	40.96	85.99	33.98	EQ6-TS86-EQ9	75.25	97.53	77.33
EQ12-TS28-EQ10	33.98	121.31	40.96	EQ2-TS89-EQ23	57.93	124.09	93.09
EQ12-TS29-EQ13	33.98	74.48	36.34	EQ24-TS92-EQ18	95.42	141.38	117.86
EQ12-TS30-EQ14	33.98	95.91	39.36	EQ25-TS97-EQ18	97.58	140.52	117.86
EQ8-TS32-EQ12	43.38	90.90	33.98	EQ26-TS99-EQ7	118.01	119.18	-4.91
EQ10-TS33-EQ13	40.96	88.19	36.34	EQ26-TS100-EQ7	118.01	119.07	-4.91
EQ13-TS34-EQ8	36.34	90.54	43.38	EQ26-TS101-EQ7	118.01	120.12	-4.91
EQ15-TS36-EQ13	55.21	99.10	36.34	EQ26-TS102-EQ17	118.01	118.39	118.16
EQ1-TS37-EQ12	21.46	119.47	33.98	EQ17-TS103-EQ7	118.16	119.73	-4.91
EQ13-TS38-EQ10	36.34	87.75	40.96	EQ13-TS109-EQ8	36.34	109.88	43.38
EQ11-TS39-EQ5	36.44	41.23	38.93	EQ27-TS110-EQ28	153.10	186.16	178.18
EQ10-TS40-EQ11	40.96	115.04	36.44	EQ19-TS113-EQ15	144.21	148.00	55.21
EQ14-TS41-EQ7	39.36	73.11	-4.91	EQ28-TS116-EQ24	178.18	195.89	95.42
EQ7-TS42-EQ16	-4.91	71.71	70.75	EQ9-TS117-EQ28	77.33	199.11	178.18
EQ7-TS43-EQ18	-4.91	120.95	117.86	EQ27-TS118-EQ14	153.10	154.15	39.36
EQ15-TS44-EQ12	55.21	152.72	33.98	EQ24-TS121-EQ29	95.42	206.40	200.29
EQ14-TS45-EQ8	39.36	114.74	43.38	EQ29-TS122-EQ28	200.29	236.89	178.18
EQ2-TS48-EQ5	57.93	58.12	38.93	EQ21-TS125-EQ4	180.83	188.65	58.21
EQ2-TS49-EQ0	57.93	87.45	0				

List of Equilibrium Structures

Geometry of EQ 0, SYMMETRY = C2v

C	-1.482634122737	-0.223779359132	-0.007761445330
H	-2.380152868931	-0.826314702295	-0.034175690634
C	-0.144300615699	-0.272211813207	0.019536660256
H	0.707645203017	-0.938082631910	0.028850542860
C	-0.768246765731	1.011406116297	0.023490942446
O	-0.725192529922	2.210489470251	0.040172790398

Energy = -190.728186922925

Spin(**2) = 0.000000000000

ZPVE = 0.037656735147

Normal mode eigenvalues : nmode = 12

0.008391299 0.010334370 0.022317105 0.025786595 0.027586288

0.036875453 0.041358613 0.050600436 0.092803089 0.140514576

0.384542100 0.392202837

Geometry of EQ 1, SYMMETRY = Cs

C	-1.294000008738	-0.129114309200	-0.002548885500
H	-2.281917954111	-0.571234523551	-0.029002682700
C	-0.079073131211	-0.782031310420	0.014195300059
H	0.781039666506	-0.097501990408	0.041180996435
C	-1.064529385632	1.211319321187	0.019923473256
O	-0.704394186508	2.303060937503	0.041840284111

Energy = -190.692923509648

Spin(**2) = 0.000000000000

ZPVE = 0.036587822179

Normal mode eigenvalues : nmode = 12

0.002831123 0.007635204 0.011085535 0.018500041 0.038380591

0.040479290 0.043229271 0.063638343 0.067703044 0.178260369

0.344972971 0.378628752

Geometry of EQ 2, SYMMETRY = C1

C	-1.475200339504	0.356046261160	-0.210351485724
H	-2.475380845622	0.013723530604	0.052817041582
C	-0.220203781254	-0.204237411010	-0.063237615590
H	0.160836246400	-1.192235630422	0.122389777970
C	-0.037407166092	1.034538810210	0.578597902768
O	-1.181375389073	1.670449057020	-0.173518873063

Energy = -190.634812507048

Spin(**2) = 0.000000000000

ZPVE = 0.036592594161

Normal mode eigenvalues : nmode = 12

0.005664948 0.011691560 0.021301250 0.027151446 0.035461012

0.038618101 0.049284531 0.059350318 0.063652410 0.080005286

0.367154096 0.408346231

Geometry of EQ 3, SYMMETRY = Cs

C	-0.940707151639	1.297800259725	-0.151183020219
H	-0.184526146806	-1.668613021299	1.310976713165
C	-0.232707378683	-1.054029276662	0.415999463858
H	0.100492132342	-1.530732679105	-0.504261971973
C	-0.669921184612	0.190838549661	0.456652367620
O	-1.239296742115	2.361527589264	-0.525808722427

Energy = -190.744715966220

Spin(**2) = 0.000000000000

ZPVE = 0.037354187026

Normal mode eigenvalues : nmode = 12

0.000842416 0.002684489 0.009508820 0.018411472 0.034187621

0.040234554 0.044022911 0.083953629 0.118422161 0.188595440

0.358927046 0.375896982

Geometry of EQ 4, SYMMETRY = Cs

C	-1.910595375746	1.140907333925	-0.258375199628
H	-0.309136747534	-1.663211132676	0.347802265630

C	0.060883208623	-0.648563668725	0.300469955209
H	1.113741841643	-0.467898509085	0.470848487668
C	-0.776293690026	0.326548953059	0.038883133915
O	-0.719093683833	1.732756142241	-0.109263343108

Energy = -190.652841310418

Spin(**2) = 0.000000000000

ZPVE = 0.055074234983

Normal mode eigenvalues : nmode = 12

0.004408125 0.005976863 0.019403461 0.021438125 0.029149096

0.029635624 0.044940683 0.057034822 0.079732725 0.128170737

0.376476228 0.398082123

Geometry of EQ 5, SYMMETRY = Cs

C	-1.307193719303	0.769981807170	0.659315833150
H	-1.842133880305	0.220691347584	1.453705137672
C	-0.438133079149	-0.102364096268	-0.184887882780
H	-0.398796230286	-1.176535960718	0.038616885747
C	0.341699690136	0.122234335499	-1.197224292853
O	-1.433340004122	1.953626682655	0.514499663006

Energy = -190.663168491009

Spin(**2) = 0.000000000000

ZPVE = 0.034670818603

Normal mode eigenvalues : nmode = 12

0.000253831 0.001536525 0.011010996 0.012490378 0.030203359

0.036617466 0.038360204 0.076592280 0.109510171 0.124963840

0.327795448 0.352860003

Geometry of EQ 6, SYMMETRY = Cs

C	-2.447927460609	0.061268186724	-0.024122362789
H	-3.368546811787	-0.466640636901	-0.050303476121
C	0.885985873322	0.731936466651	0.054296506337
H	1.571076279854	1.605753287860	0.080137245737

C	-1.395383834794	0.623533931696	0.005264890833
O	-0.295149018518	1.350411075946	0.037821949398

Energy = -190.605826680630

Spin(**2) = 0.000000000000

ZPVE = 0.035213857218

Normal mode eigenvalues : nmode = 12

0.002241176 0.004668398 0.012686215 0.015424205 0.015920259

0.020136819 0.034502533 0.057115034 0.078465789 0.195108889

0.316320556 0.458831229

Geometry of EQ 7, SYMMETRY = Cs

C	-1.114586989653	-1.113886579772	-0.264413715278
H	-1.251777286369	-2.124727379649	-0.565083817690
C	-0.951600190882	0.033241000292	0.054035295670
H	-1.070873822150	1.582492745750	1.542320554479
C	-0.777004832277	1.398329099388	0.493280941522
O	-0.348109752624	2.281799789900	-0.207507275801

Energy = -190.735492605503

Spin(**2) = 0.000000000000

ZPVE = 0.037138346615

Normal mode eigenvalues : nmode = 12

0.001792933 0.002927568 0.015281914 0.017689880 0.020924962

0.034734761 0.038824605 0.075830400 0.116457237 0.183384791

0.326772954 0.453763669

Geometry of EQ 8, SYMMETRY = Cs

C	-1.011777291317	-1.129277007694	-0.077005008393
H	-1.020542807253	-2.192325033141	-0.099242070156
C	-1.017831778559	0.081766710560	-0.055910197475
H	0.639216942695	1.672053316449	0.411495550038
C	-1.220614021844	1.476131114353	-0.083364683068
O	-0.164636564822	2.196756839076	0.209004326562

Energy = -190.657571189230

Spin(**2) = 0.000000000000

ZPVE = 0.036165997901

Normal mode eigenvalues : nmode = 12

0.001835761 0.002657847 0.011097424 0.015495413 0.023065528

0.025903123 0.030785210 0.065040227 0.068747148 0.168846182

0.452647623 0.455369505

Geometry of EQ 9, SYMMETRY = Cs

C	-1.073317558466	-0.935960414258	-0.541274975870
H	-1.299552110171	-1.956551318328	-0.727751515722
C	-0.823097581732	0.210897268386	-0.318180312441
H	-1.117065523519	1.336239216313	1.726176204308
C	-0.700681355771	2.107805160928	1.036944421492
O	-0.526819327124	1.487671542448	-0.129757131695

Energy = -190.602204969649

Spin(**2) = 0.000000000000

ZPVE = 0.034912177314

Normal mode eigenvalues : nmode = 12

0.002025878 0.004512272 0.013337529 0.015289326 0.016092441

0.019568111 0.033463543 0.055208024 0.077801082 0.192143724

0.303889041 0.457900037

Geometry of EQ 10, SYMMETRY = Cs

C	-1.097090779408	-1.099913854500	-0.308879025619
H	-1.232773121892	-2.109266683604	-0.613084682939
C	-0.937464338780	0.053118025876	0.024219549604
H	-0.328485771747	3.065522353005	0.296146172955
C	-0.844600347727	1.313736888489	0.643117687913
O	-0.369103218618	2.225450222622	-0.182647253774

Energy = -190.662064688389

Spin(**2) = 0.000000000000

ZPVE = 0.036804899510

Normal mode eigenvalues : nmode = 12

0.002208457 0.002805055 0.010506256 0.014944866 0.023494464

0.025016468 0.032419804 0.061646144 0.071388640 0.169638797

0.453903946 0.526376407

Geometry of EQ 11, SYMMETRY = Cs

C	-1.251098047605	-1.138963725704	0.143471006091
H	-0.501771148120	-0.004509607239	-1.376116209599
C	-0.835646990814	-0.008212843570	-0.337533802535
H	-1.118525152290	1.249232852681	1.450652150168
C	-0.774952151165	1.287506334112	0.407021400786
O	-0.378422225380	2.302234842051	-0.098007723291

Energy = -190.667672799928

Spin(**2) = 0.000000000000

ZPVE = 0.035216937705

Normal mode eigenvalues : nmode = 12

0.000790185 0.001825514 0.009915292 0.016569706 0.027732741

0.037927201 0.041532721 0.076630960 0.110140319 0.119515282

0.338952269 0.367699136

Geometry of EQ 12, SYMMETRY = Cs

C	-0.797381000744	-0.444902810722	-0.251636219104
H	-0.486603935238	-1.340290645539	-0.766242623647
C	-1.670478509107	0.105837020873	0.712099774371
H	-0.585975078667	2.687272227256	0.316206053371
C	-0.722468257298	0.851612606207	-0.004649860709
O	-0.166248284580	2.016440309457	-0.246748261641

Energy = -190.674664373056

Spin(**2) = 0.000000000000

ZPVE = 0.038282648033

Normal mode eigenvalues : nmode = 12

0.006631402 0.008927090 0.013170374 0.025906823 0.030634253
0.035184618 0.055099500 0.056889469 0.076930650 0.127252061
0.397396022 0.514107648

Geometry of EQ 13, SYMMETRY = Cs

C	-1.048902941045	-0.443450878810	-0.019776999714
H	-1.698970729601	-1.303896036544	-0.048248926854
C	-0.815265226138	0.860091222434	0.024014054919
H	-2.171266752988	2.170564517772	0.069441790614
C	0.292836882214	0.003221503261	-0.005868755586
O	-1.204631849230	2.116243072840	0.066752979420

Energy = -190.670808714717

Spin(**2) = 0.000000000000

ZPVE = 0.038196098336

Normal mode eigenvalues : nmode = 12

0.007223862 0.008983739 0.011788732 0.025776138 0.029946429
0.034000495 0.051019126 0.058718704 0.077863535 0.124178015
0.396104085 0.527444740

Geometry of EQ 14, SYMMETRY = Cs

C	-0.596724925232	0.437481887845	-0.606605598449
H	-0.543786544335	-0.417934786850	-1.273888157813
C	-1.119459472700	0.385343185785	0.623227876689
H	-0.144650479045	2.263194426809	-0.547319674651
C	-1.618551038294	0.312551654991	1.782981600532
O	-0.082096357611	1.517630407538	-1.168606076158

Energy = -190.665952463159

Spin(**2) = 0.000000000000

ZPVE = 0.038149255596

Normal mode eigenvalues : nmode = 12

0.001155657 0.001446753 0.013646316 0.019380809 0.037623371
0.041008643 0.059273377 0.068826242 0.084608993 0.163345365

0.377910147   0.514752871

Geometry of EQ 15, SYMMETRY = Cs

C	-0.698001766449	0.486681636747	-0.499450307092
H	-0.415452054176	-0.416846276451	-1.039802823310
C	-1.291069691481	0.429777157952	0.692982828519
H	0.014393349160	1.474251022972	-1.959330367654
C	-1.846785403135	0.239817738951	1.814178769944
O	-0.412998162070	1.632613664582	-1.105870873879

Energy = -190.658729883350

Spin(**2) = 0.000000000000

ZPVE = 0.056181642974

Normal mode eigenvalues : nmode = 12

0.001306260   0.001376617   0.012057261   0.013646247   0.035117841

0.041081410   0.062458926   0.062723152   0.087507789   0.164919128

0.364254407   0.533217575

Geometry of EQ 16, SYMMETRY = Cs

C	-0.949391935476	-0.432141610400	0.365941354968
H	-1.194897595923	0.452775592562	-1.712406664522
C	-0.752811918487	0.432622231481	-0.722960090951
H	-1.923670457314	1.430962071285	1.227640109025
C	-1.112284060595	0.915115787307	0.727233962426
O	-0.319435697505	1.680336277043	-0.180746546263

Energy = -190.614509584624

Spin(**2) = 0.000000000000

ZPVE = 0.036727557666

Normal mode eigenvalues : nmode = 12

0.007122377   0.012883964   0.024328536   0.031549873   0.037384699

0.044623791   0.045942065   0.053179419   0.058980462   0.073445124

0.384321922   0.386066683

Geometry of EQ 17, SYMMETRY = C2

C	-1.172740364766	0.316034777762	1.064105657123
H	-0.973573849053	1.518814615427	-1.885598790805
C	-0.536839663024	0.871476631282	-1.140805937525
H	-0.655591114191	-0.181851528916	1.869807360446
C	-1.132866660672	0.077516485951	-0.248585920172
O	-0.392207666684	1.452594513009	0.311425282424

Energy = -190.535608179414

Spin(**2) = 0.000000000000

ZPVE = 0.033375183228

Normal mode eigenvalues : nmode = 12

0.001545888 0.004295655 0.006250698 0.009942873 0.022224640

0.028554978 0.041180107 0.043510667 0.064795636 0.103617887

0.396930754 0.397201771

Geometry of EQ 18, SYMMETRY = Cs

C	-0.685111707503	-1.104483425336	-0.362178942494
H	-2.089510387648	0.308321459074	-0.599661040865
C	-1.069947699631	0.141284238672	-0.231851933008
H	-0.270063495017	3.040809864533	0.746445219145
C	-1.029157459823	2.370242552270	0.291695303789
O	-0.390028903199	1.219495177117	0.284160092522

Energy = -190.536210095666

Spin(**2) = 0.000000000000

ZPVE = 0.033506975705

Normal mode eigenvalues : nmode = 12

0.000891749 0.002141678 0.009603541 0.016103098 0.024295991

0.030749749 0.045512253 0.060970265 0.075848407 0.113694913

0.317103858 0.351587104

Geometry of EQ 19, SYMMETRY = C1

C	-0.551342172937	-0.224979759820	-0.885156029885

H	-1.385665180510	-0.153005503597	-1.605224029555
C	-0.911830699999	0.547101144891	0.285235226394
H	-1.025875973126	2.179794617345	-0.787889161140
C	-1.424675233585	0.666602109334	1.491759105831
O	-0.308792937465	1.616220673488	-0.451595867569

Energy = -190.494011720787

Spin(**2) = 0.000000000000

ZPVE = 0.033287616317

Normal mode eigenvalues : nmode = 12

0.001782763 0.002089057 0.004988344 0.012029312 0.023662716

0.029992744 0.032984383 0.042866613 0.056406304 0.105095034

0.332988583 0.518126451

Geometry of EQ 20, SYMMETRY = Cs

C	-1.352740716452	0.382016633324	0.897057878358
H	-2.325842181402	-0.071725954668	0.751244600778
C	-0.528613287244	0.645356053731	-0.292371958690
H	-0.846953150603	0.236791064765	1.844239912028
C	0.228908547224	0.032406396355	-1.144305957429
O	-1.187944931150	1.680467806486	0.307566961562

Energy = -190.603953284660

Spin(**2) = 0.000000000000

ZPVE = 0.036852987190

Normal mode eigenvalues : nmode = 12

0.001464871 0.004733335 0.020472078 0.031240200 0.034766839

0.044003098 0.047394706 0.051811491 0.085059434 0.124962477

0.367043290 0.393698387

Geometry of EQ 21, SYMMETRY = Cs

C	-0.820232797602	-1.044805394852	-0.024829553513
H	-0.934887048351	-2.099942963197	-0.074874658070
C	-0.688576678934	0.135943642754	0.032059757974

H	1.199672827785	1.402532028306	0.554477039718
C	-1.338896989322	2.348709222497	-0.098276353850
O	-0.507295484123	1.449214334545	0.104527144161

Energy = -190.430470510677

Spin(**2) = 0.000000000000

ZPVE = 0.028110954489

Normal mode eigenvalues : nmode = 12

0.002181277 0.003025236 0.007588256 0.007722909 0.013504863

0.016910372 0.017195982 0.022522428 0.036207399 0.080116967

0.199435791 0.457439018

Geometry of EQ 22, SYMMETRY = Cs

C	-0.893304764449	-1.118328433652	-0.060475350832
H	-1.155364987768	-2.146471877257	-0.106262085215
C	-0.615566497461	0.040122343031	-0.011375167225
H	0.596550693993	1.557430415554	0.169964437705
C	-1.428777309540	2.504879745777	-0.100073921459
O	-0.349701667150	1.346825188880	0.037522178553

Energy = -190.426717242709

Spin(**2) = 0.000000000000

ZPVE = 0.050894300766

Normal mode eigenvalues : nmode = 12

0.001200985 0.002429837 0.002974429 0.006238917 0.007213885

0.014447767 0.016864464 0.036347661 0.058781368 0.196016248

0.448085028 0.458077746

Geometry of EQ 23, SYMMETRY = Cs

C	-1.565114970316	0.524187749111	-0.587425286075
H	-0.740356136599	-1.376645467441	0.415598082934
C	-0.507751490902	-0.403774216297	-0.007285585875
H	0.310525741345	-0.447558592637	-0.725284604392
C	-0.294603892883	0.841404832685	0.841144560779

O	-0.835276301508	1.606189908991	-0.162299139922

Energy = -190.577931755642

Spin(**2) = 0.000000000000

ZPVE = 0.035745524283

Normal mode eigenvalues : nmode = 12

0.006986362 0.011735446 0.025095046 0.027548572 0.033840478

0.038026894 0.040193380 0.052906633 0.052945090 0.077592026

0.361842925 0.379925529

Geometry of EQ 24, SYMMETRY = Cs

C	-2.600731558524	1.215326221064	-0.391628205105
H	-0.035331332416	-1.179623258015	0.344080434323
C	0.507416631623	-0.242170287314	0.335851262680
H	1.565762611451	-0.134971589957	0.515095557780
C	-1.395445367498	0.960307809436	-0.144786664981
O	-0.117528467011	0.845118141311	0.099416079366

Energy = -190.574804721872

Spin(**2) = 0.000000000000

ZPVE = 0.036339703702

Normal mode eigenvalues : nmode = 12

0.001148154 0.001206958 0.010063329 0.016101857 0.030312442

0.032567207 0.059004773 0.075103294 0.086903293 0.151613471

0.371773429 0.411915106

Geometry of EQ 25, SYMMETRY = Cs

C	-1.506161549819	0.354491074227	0.272960314933
H	-2.543448233928	0.084889246357	0.323014503417
C	-0.265889904615	0.153843214773	-0.352463004364
H	0.288321733554	-0.373224821027	-1.104926244109
C	-0.284243733303	0.880767076391	0.952611603295
O	-0.942344189005	1.462216291151	-0.538999920752

Energy = -190.570794227238

Spin(**2) = 0.000000000000

ZPVE = 0.035767224984

Normal mode eigenvalues : nmode = 12

0.006017733 0.016130486 0.023597165 0.024299993 0.027398977

0.036808113 0.037263907 0.040252754 0.048396287 0.079967478

0.410625909 0.416643885

Geometry of EQ 26, SYMMETRY = C1

C	-1.162534993478	-0.022435608174	0.174139375006
H	-0.548355061702	0.226136608824	-1.998620110335
C	-0.435502649677	0.249771056440	-0.931501745810
H	-1.686566666670	1.482476857817	1.659059845051
C	-0.900304659428	0.917900999634	1.169531232878
O	-0.616083202271	1.522290714710	-0.219020464324

Energy = -190.535569115510

Spin(**2) = 0.000000000000

ZPVE = 0.033101858634

Normal mode eigenvalues : nmode = 12

0.000041240 0.004096929 0.009888028 0.013590944 0.022870327

0.031490498 0.041180130 0.049939239 0.059908850 0.076957008

0.382361074 0.415416626

Geometry of EQ 27, SYMMETRY = C1

C	-1.422860773607	0.141784684623	0.984131076163
H	-2.468911312577	0.291757306138	0.674021922256
C	-0.580992532849	0.491654977079	-0.138179183016
H	-0.034390858204	1.893767855452	1.111774781619
C	0.117784398016	0.209503693575	-1.217794134011
O	-0.713742801582	1.799914928615	0.423960856926

Energy = -190.498680821925

Spin(**2) = 0.000000000000

ZPVE = 0.052129601451

Normal mode eigenvalues : nmode = 12

0.001737720 0.002636448 0.005290810 0.011640702 0.026518351

0.032099592 0.033488041 0.042056948 0.058275640 0.106747899

0.340970524 0.521486663

Geometry of EQ 28, SYMMETRY = Cs

C	-1.775864743417	0.755912733086	1.488677421467
H	-2.037250212957	-0.218455436786	1.042276413759
C	-0.270259092222	0.651695610329	-0.536345479375
H	-0.457141445166	2.075580398474	0.849420873804
C	0.297961975306	0.266818684458	-1.578167490373
O	-0.817795111404	1.196788150711	0.607869237962

Energy = -190.441112339845

Spin(**2) = 0.000000000000

ZPVE = 0.034533017813

Normal mode eigenvalues : nmode = 12

0.000093496 0.000332306 0.008415903 0.010832035 0.024038734

0.031616243 0.037352549 0.061658761 0.081534423 0.151590316

0.340289967 0.479440243

Geometry of EQ 29, SYMMETRY = Cs

C	-0.337665787787	-0.003106769817	-1.488542069524
H	-0.064804236436	0.644622583319	-2.344787101247
C	-1.023212339897	0.760336029120	0.764454153567
H	-0.541340239819	1.911298483556	-0.747326202664
C	-1.381049020772	1.016693351669	1.934119328267
O	-0.619184697737	0.951937467660	-0.536020089327

Energy = -190.430697274050

Spin(**2) = 0.000000000000

ZPVE = 0.059352924079

Normal mode eigenvalues : nmode = 12

0.000490670 0.000553929 0.008880102 0.011256257 0.025630744

0.025644762 0.036612412 0.061697665 0.081995389 0.149558797
0.325784183 0.445878598

List of Transition Structures

Geometry of TS 0, SYMMETRY = Cs

C	-1.421107688518	-0.175705002123	-0.005792085655
H	-2.336849780289	-0.759333761703	-0.032558096223
C	-0.111039242051	-0.482940185218	0.017442965592
H	0.887161775616	-0.094997247179	0.043347923427
C	-0.940174589025	1.074088337695	0.020706174625
O	-0.690723077103	2.228556463126	0.041126109322

Energy = -190.677837662007

Spin(**2) = 0.000000000000

ZPVE = 0.035220190752

Normal mode eigenvalues : nmode = 12

-0.025144056 0.005781850 0.010155686 0.012992559 0.018195858

0.031536169 0.041975925 0.059258589 0.084112653 0.165849785

0.367655794 0.425460400

CONNECTION : 1 - 0

Geometry of TS 1, SYMMETRY = C1

C	-1.471303275108	-0.003973732252	-0.394882710775
H	-2.444614860705	0.046022227043	0.072452920931
C	-0.223290680844	-0.292138407056	-0.099945812022
H	0.507241575360	-1.074845069225	-0.108101532527
C	-0.237077329814	1.048974088802	0.506480051687
O	-1.014884293870	1.845847705235	-0.088859056635

Energy = -190.590137342947

Spin(**2) = 0.000000000000

ZPVE = 0.032899129536

Normal mode eigenvalues : nmode = 12

-0.028349207 0.002045345 0.011140167 0.013850201 0.017494510

0.030258596 0.037298610 0.039626564 0.076458983 0.087320439

0.384846098 0.422547186

CONNECTION : 2 - DC

Geometry of TS 2, SYMMETRY = Cs

C	-1.298128432608	-0.283887001241	0.073145399420
H	-2.350037549437	-0.469712515074	0.176358612423
C	-0.229389814309	-0.975459053230	-0.032165957864
H	0.813502385801	-0.732126270408	-0.134447408958
C	-0.980523444129	1.324330863061	0.042762605849
O	-0.107086619129	2.061349305118	-0.042688452070

Energy = -190.680539640670

Spin(**2) = 0.000000000000

ZPVE = 0.034260883370

Normal mode eigenvalues : nmode = 12

-0.012115901 0.001701237 0.003251876 0.013198652 0.018931748
0.024570683 0.033023791 0.056139486 0.104092257 0.158248723
0.406002368 0.411875147

CONNECTION : 1 - DC

Geometry of TS 3, SYMMETRY = C1

C	-1.971724429797	0.469290454967	-0.361607816861
H	-0.753236461795	-1.404746600782	0.517500782480
C	-0.195315515868	-0.554227881171	0.146636333707
H	0.754833656049	-0.722042994921	-0.348020806336
C	-0.764243488667	0.704948721117	0.268968354387
O	-0.439469688315	1.881725883633	-0.048105754348

Energy = -190.585495826301

Spin(**2) = 0.000000000000

ZPVE = 0.034764813935

Normal mode eigenvalues : nmode = 12

-0.003777699 0.004649703 0.007621494 0.016446678 0.029716181
0.035641680 0.048512314 0.072616053 0.080165170 0.096817046
0.371265294 0.397338300

CONNECTION : 4 - 3

Geometry of TS 4, SYMMETRY = Cs

C	-1.937464690608	1.008237083051	0.017115102956
H	-0.916210556337	-1.348188803306	0.018556122760
C	-0.108545514221	-0.629153743749	0.015482465650
H	0.902587405041	-1.026762387573	0.013753097337
C	-0.190001330525	0.696458384315	0.013511590264
O	-1.100515274627	1.888637259931	0.013702454778

Energy = -190.573270089063

Spin(**2) = 0.000000000000

ZPVE = 0.034181477897

Normal mode eigenvalues : nmode = 12

-0.022452315 0.001275061 0.005194315 0.016947222 0.020648257
0.032945225 0.042894064 0.081889327 0.093698895 0.110243561
0.363829937 0.385673562

CONNECTION : 4 - DC

Geometry of TS 5, SYMMETRY = Cs

C	-1.807811953498	-0.039194963791	-0.012133776541
H	-2.334231139716	-0.971601248008	-0.034166265979
C	0.262914355988	0.381089171164	0.037976317943
H	1.371730958908	0.505803051332	0.064142678116
C	-1.724058098319	1.239929880444	0.004423339498
O	-0.313528454352	1.533226721841	0.038486555248

Energy = -190.537271674921

Spin(**2) = 0.000000000000

ZPVE = 0.033152952898

Normal mode eigenvalues : nmode = 12

-0.013381430 0.002882224 0.010569135 0.012993957 0.023884012
0.030006821 0.033294521 0.066445297 0.071428599 0.104118703
0.301540972 0.426928506

CONNECTION : 6 - 5

Geometry of TS 6, SYMMETRY = C2v

C	-0.950279982529	1.321039822429	-0.142726817259
H	-0.305832505568	-1.438487242240	1.540322707088
C	-0.242856130801	-1.077256591186	0.515294591953
H	0.136796474428	-1.789646234352	-0.215117007264
C	-0.602099980920	0.140680598470	0.181078099769
O	-1.271015488062	2.408375378627	-0.441042208442

Energy = -190.744018868753

Spin(**2) = 0.000000000000

ZPVE = 0.036802919046

Normal mode eigenvalues : nmode = 12

-0.000531309 0.001858735 0.008001746 0.015996080 0.032743748

0.039729050 0.043453723 0.083651101 0.121564111 0.202017683

0.357051057 0.372059859

CONNECTION : 3 - 3

Geometry of TS 7, SYMMETRY = C1

C	-0.960065579034	1.303138263629	-0.087818186872
H	-0.543171366901	-1.333688884060	1.719086627449
C	-0.162242058355	-1.075740873920	0.723092783604
H	-0.247975254039	-0.798840334328	-0.664079214142
C	-0.461394552376	0.086494100482	0.118404600781
O	-1.232686056745	2.305313200598	-0.610835036333

Energy = -190.613795824213

Spin(**2) = 0.000000000000

ZPVE = 0.029052038982

Normal mode eigenvalues : nmode = 12

-0.106565602 0.001057596 0.001716138 0.002728529 0.005261323

0.019748531 0.027060088 0.038355850 0.093992901 0.173266649

0.225310743 0.340675597

CONNECTION : 3 - 1

Geometry of TS 8, SYMMETRY = Cs

C	-0.862976182056	1.406443937944	-0.786330733706
H	-0.462015112683	-0.985147618041	1.548535235853
C	-0.299462938407	-0.894164245084	0.480707360497
H	0.093029525225	-1.739559831039	-0.074621060692
C	-0.566608541660	0.204953745747	-0.136043358703
O	-1.272279347177	2.036946686946	0.269762550421

Energy = -190.605481122885

Spin(**2) = 0.000000000000

ZPVE = 0.032718821723

Normal mode eigenvalues : nmode = 12

-0.037950101 0.002048640 0.004049209 0.008975637 0.019125892
0.026926458 0.032381322 0.064222060 0.071504134 0.122083414
0.372869311 0.393192085

CONNECTION : 4 - 3

Geometry of TS 9, SYMMETRY = Cs

C	-0.926742516896	1.281747311728	-0.126958284114
H	-0.086292450454	-2.085656981973	0.773088613600
C	-0.189748064804	-0.938696111024	0.807596873812
H	-0.565229271388	-2.193628130780	-0.141839005668
C	-0.654170039215	0.053382203818	0.137881885274
O	-1.215975895977	2.361370711408	-0.452390035677

Energy = -190.606310712747

Spin(**2) = 0.000000000000

ZPVE = 0.027661939841

Normal mode eigenvalues : nmode = 12

-0.066007551 0.000646343 0.001709487 0.007993679 0.011755460
0.015601124 0.026120918 0.034907188 0.088046783 0.129405769
0.197501852 0.273783944

CONNECTION : 3 - DC

Geometry of TS 10, SYMMETRY = Cs

C	-1.300295915067	-1.267964194121	0.203045681179
H	-1.138320290670	-1.838194849896	-0.712608867856
C	-0.982218446499	-0.000432916328	0.124871841527
H	-1.162139679478	0.769058827973	1.240028842994
C	-0.660107154869	1.264365578097	0.032138866574
O	-0.344818728663	2.378052301876	-0.148201759802

Energy = -190.632828602469

Spin(**2) = 0.000000000000

ZPVE = 0.030979335610

Normal mode eigenvalues : nmode = 12

-0.045964059 0.001803703 0.004311433 0.014138264 0.025660040

0.032058296 0.033979548 0.034535678 0.103669464 0.120967962

0.186986365 0.368083425

CONNECTION : 7 - 1

Geometry of TS 11, SYMMETRY = Cs

C	-1.089647449770	-1.168324718886	-0.451004446580
H	-1.685688347904	-1.916771662210	0.061607724543
C	-1.011072842702	0.046165651666	0.012106672955
H	-1.578129878027	0.663879761499	1.132232741146
C	-0.720992893856	1.315351500694	0.192991425582
O	-0.387569730684	2.435278293697	0.239723731622

Energy = -190.637834017613

Spin(**2) = 0.000000000000

ZPVE = 0.030833870007

Normal mode eigenvalues : nmode = 12

-0.058102109 0.002068375 0.003883030 0.014258216 0.019963828

0.032896460 0.035986427 0.039111400 0.097863216 0.110515770

0.187164583 0.381544528

CONNECTION : 7 - 0

Geometry of TS 12, SYMMETRY = C1

C	-1.072020844053	-0.609379397059	-0.137674283921
H	-1.332557226985	-1.619829238151	0.064153087564
C	-0.743438851158	0.489853502692	-0.561975027182
H	-1.831356695622	1.500164185689	1.097122507727
C	-0.745401656905	1.277088024565	1.073997092240
O	-0.434968699773	1.718802819099	-0.369205807426

Energy = -190.566259148508

Spin(**2) = 0.000000000000

ZPVE = 0.033426980234

Normal mode eigenvalues : nmode = 12

-0.001169317 0.003910799 0.007931589 0.010178262 0.018060440

0.023523096 0.031432697 0.054022794 0.066852184 0.165515359

0.319929159 0.453425546

CONNECTION : 2 - 9

Geometry of TS 13, SYMMETRY = Cs

C	-1.164717639266	-1.172020417692	-0.241649207887
H	-1.372336258682	-2.181915556976	-0.502009118963
C	-0.925262941077	-0.020274032760	0.032217602887
H	-0.457492111960	2.426592336122	0.967053077494
C	-0.683575471624	1.263586224955	0.555023788121
O	-0.356940802462	2.274885891289	-0.206972712102

Energy = -190.605465957315

Spin(**2) = 0.000000000000

ZPVE = 0.030887483488

Normal mode eigenvalues : nmode = 12

-0.165692931 0.001824187 0.002402166 0.012560440 0.013540928

0.015093369 0.022550575 0.033539655 0.070919217 0.173693974

0.254865291 0.453284202

CONNECTION : 7 - 10

Geometry of TS 14, SYMMETRY = Cs

C	-1.155726460875	-1.191146633071	-0.191750079720
H	-0.695867387575	-0.487615858801	-1.118776837960
C	-0.957953234583	0.024204435711	0.067342633514
H	-1.067853402289	1.577748397533	1.529547899795
C	-0.771871507161	1.401632565722	0.479681576546
O	-0.341808613115	2.270325695779	-0.235389220310

Energy = -190.661971039055

Spin(**2) = 0.000000000000

ZPVE = 0.031757925711

Normal mode eigenvalues : nmode = 12

-0.014324781 0.002074300 0.002410562 0.007035907 0.016282003

0.032521353 0.037760029 0.074321447 0.116944501 0.145064667

0.213983272 0.328331138

CONNECTION : 11 - 7

Geometry of TS 15, SYMMETRY = Cs

C	-1.076577903505	-1.167116695892	-0.421275038317
H	-1.421043517859	-0.992199415048	0.778967902966
C	-0.963981697763	-0.008578677533	0.062489380249
H	-1.083573795403	1.552978266017	1.560797631605
C	-0.791590398886	1.363484272047	0.513677871912
O	-0.360789030719	2.223063501188	-0.210316984469

Energy = -190.659740822963

Spin(**2) = 0.000000000000

ZPVE = 0.031763701263

Normal mode eigenvalues : nmode = 12

-0.009317940 0.002419662 0.002868977 0.005287806 0.016586905

0.032109574 0.037705952 0.076187403 0.117229333 0.144485993

0.212805747 0.330464193

CONNECTION : 7 - 5

Geometry of TS 16, SYMMETRY = C1

C	-0.957673760901	-0.585500452604	-0.096244944205
H	-0.174881886278	-1.112142787643	-0.604409799016
C	-2.064759328259	-0.181857654558	0.338966458616
H	-0.522246840812	1.292570715847	1.392315778837
C	-0.584513816637	1.054368593012	0.327221842559
O	-0.141688663981	1.699248941821	-0.560894652597

Energy = -190.649567623192

Spin(**2) = 0.000000000000

ZPVE = 0.034411184220

Normal mode eigenvalues : nmode = 12

-0.006076645 0.001735129 0.006237465 0.014842389 0.016285138

0.028390914 0.035854334 0.065396005 0.121624587 0.129021363

0.355083636 0.423685225

CONNECTION : 11 - 7

Geometry of TS 17, SYMMETRY = C1

C	-0.306974213722	-0.348242215667	-0.644356728467
H	-1.178472809812	-0.791172102791	-1.171228386149
C	-0.667432527835	1.051621018249	0.242933777690
H	-1.434278569079	1.607202980232	2.311856529983
C	-1.140439478824	1.402309168929	1.311236976363
O	-0.332358893479	1.032882040469	-1.046221695517

Energy = -190.564358931477

Spin(**2) = 0.000000000000

ZPVE = 0.031969576185

Normal mode eigenvalues : nmode = 12

-0.006197879 0.001712161 0.006786640 0.009559948 0.013538706

0.016878102 0.026727385 0.043859042 0.066106821 0.159365206

0.313175814 0.453260429

CONNECTION : 7 - 9

Geometry of TS 18, SYMMETRY = C1

C	-1.400886405253	-0.350386502437	0.004536820358
H	-2.294642063452	-0.900879224483	0.283692048431
C	0.058230909403	-0.200878640037	0.409913311969
H	-0.730888011018	-1.015724555449	-0.657646846090
C	-0.775954212052	0.915396936534	-0.106616381238
O	-0.883525410578	2.107002908812	-0.135339787132

Energy = -190.602721171339

Spin(**2) = 0.000000000000

ZPVE = 0.032642314953

Normal mode eigenvalues : nmode = 12

-0.006691708 0.004566665 0.009518133 0.013484729 0.027286767
0.031625121 0.038944133 0.046280528 0.066502842 0.139948442
0.258061424 0.372852895

CONNECTION : 0 - 3

Geometry of TS 19, SYMMETRY = Cs

C	-1.237753788911	-0.521210469762	0.023671063441
H	-2.173919075000	-0.631578919873	-0.524954919272
C	-0.158246319339	0.473516460729	-0.361244428529
H	-0.835964813059	-1.408001132039	0.495353862414
C	-0.839625031092	0.753279630744	0.745207274328
O	-0.801565156210	1.765697865025	-0.285466314467

Energy = -190.553863216376

Spin(**2) = 0.000000000000

ZPVE = 0.034716626588

Normal mode eigenvalues : nmode = 12

-0.003251686 0.002919817 0.008696107 0.030793804 0.033145567
0.042881570 0.043313451 0.051102171 0.080764376 0.095802513
0.357413275 0.392927043

CONNECTION : 3 - 3

Geometry of TS 20, SYMMETRY = Cs

C	-1.357355512921	-0.138671382856	-0.004003920135
H	-2.393542520673	-0.435876676696	-0.029580166493
C	-0.199892383292	-0.827121578586	0.011096720459
H	0.491760588359	0.632349886190	0.044804516256
C	-0.631003691859	1.065590726902	0.027061034081
O	-0.780041514851	2.259112544038	0.039692944227

Energy = -190.641154114981

Spin(**2) = 0.000000000000

ZPVE = 0.033798878017

Normal mode eigenvalues : nmode = 12

-0.016483591 0.004379790 0.013765766 0.025444080 0.027097598
0.039669535 0.042312421 0.063895570 0.091095636 0.127521231
0.185400716 0.398568861

CONNECTION : 11 - 1

Geometry of TS 21, SYMMETRY = C1

C	-1.043808033095	0.066118671550	-0.172296606672
H	-2.001753602255	-0.361248780522	-0.481458941526
C	-0.067120356826	-1.005072332569	0.111694184545
H	0.492215517859	-1.189671018964	-0.830857046188
C	-1.013739325684	1.353863891096	0.026876484451
O	-0.977944444096	2.504665365827	0.217241383845

Energy = -190.623216912334

Spin(**2) = 0.000000000000

ZPVE = 0.032515839958

Normal mode eigenvalues : nmode = 12

-0.139296152 0.001630455 0.005505316 0.008236760 0.013957909
0.027489486 0.041710034 0.048421380 0.073377341 0.187168718
0.313145980 0.359862700

CONNECTION : 1 - 0

Geometry of TS 22, SYMMETRY = Cs

C	-0.859110358345	-0.396639047630	-0.329793231800
H	-0.680061856202	-1.389328606952	-0.723223773502
C	-1.699039588014	0.340764657352	0.484835838644
H	-1.301748622433	1.770634039516	0.567174829208
C	-0.545918806472	0.895641958444	-0.221458370302
O	-0.216147080648	2.124539011071	-0.142099888023

Energy = -190.587696739533

Spin(**2) = 0.000000000000

ZPVE = 0.031258192334

Normal mode eigenvalues : nmode = 12

-0.158626244 0.006620733 0.007540828 0.023888768 0.026415995
0.035971517 0.037358502 0.048676349 0.068350996 0.111567361
0.133600213 0.383554540

CONNECTION : 12 - 0

Geometry of TS 23, SYMMETRY = C1

C	-0.935783685298	-0.703455665576	-0.009105506369
H	-0.573855851984	-1.620352196917	-0.431425794581
C	-1.618930110158	0.263846741779	0.400240555407
H	-0.536128677957	2.247160987710	0.895624806313
C	-0.829746098264	1.020419488396	-0.547946655382
O	-0.267095693182	2.099394982781	-0.038219990469

Energy = -190.583807884889

Spin(**2) = 0.000000000000

ZPVE = 0.034321710906

Normal mode eigenvalues : nmode = 12

-0.023595546 0.002322823 0.008631569 0.013870814 0.021891861
0.024064108 0.039268805 0.057983038 0.069293423 0.119760347
0.423167570 0.448922650

CONNECTION : 12 - 8

Geometry of TS 24, SYMMETRY = C1

C	-0.736095753888	0.022210425650	-0.430049955284
H	0.133460871656	-0.353275689601	-0.943826484656
C	-1.691713546255	-0.138920752108	0.451859998440
H	0.186198792396	2.201434450343	-0.281694368207
C	-1.138853198529	1.060163184440	0.963975379743
O	-0.660320781841	1.742477795563	-0.450933212510

Energy = -190.533309980364

Spin(**2) = 0.000000000000

ZPVE = 0.033909547921

Normal mode eigenvalues : nmode = 12

-0.015286652 0.007166923 0.009846456 0.014207191 0.021239165
0.031229821 0.034721018 0.045775548 0.055910085 0.087628260
0.402645565 0.493808350

CONNECTION : 15 - 10

Geometry of TS 25, SYMMETRY = C1

C	-0.400315511930	0.008353978858	-0.075147097485
H	0.207876500427	-0.509201830597	-0.798229766104
C	-1.446659245902	0.058278960018	0.710522810672
H	0.111093458948	2.036321312117	0.306772585179
C	-1.778281697954	1.157638440993	-0.124972909842
O	-0.283129006588	1.729447027029	-0.523197648598

Energy = -190.525808159125

Spin(**2) = 0.000000000000

ZPVE = 0.033818368106

Normal mode eigenvalues : nmode = 12

-0.014397305 0.006795905 0.009786843 0.014058465 0.019816467
0.023360324 0.038562703 0.043221686 0.052847162 0.087225146
0.403688231 0.531972933

CONNECTION : 8 - 14

Geometry of TS 26, SYMMETRY = C1

C	-0.809986898598	-0.437278058594	-0.288192817925
H	-0.529132076785	-1.329578406192	-0.827736098182
C	-1.649449452284	0.085410898760	0.745069820711
H	-0.591083981965	2.548803932995	-0.859810065978
C	-0.746829366221	0.847570213028	0.002139884728
O	-0.153231591672	2.037554001602	-0.167819645674

Energy = -190.655922806663

Spin(**2) = 0.000000000000

ZPVE = 0.036578056795

Normal mode eigenvalues : nmode = 12

-0.013863043 0.007697787 0.008326207 0.024446768 0.031032350

0.032787165 0.048536493 0.054505582 0.068328438 0.122790169

0.393139616 0.545023512

CONNECTION : 13 - 12

Geometry of TS 27, SYMMETRY = Cs

C	-0.900902910160	-0.075451627398	-0.062864617381
H	-0.262977840009	-0.579608669369	-0.792287791754
C	-1.806934735501	-0.694171926790	0.645360319359
H	0.097627289299	2.848352179301	-0.292946346033
C	-0.870363005079	1.355787908423	0.259576096950
O	0.067567991827	1.902561592779	-0.496646013263

Energy = -190.587987006752

Spin(**2) = 0.000000000000

ZPVE = 0.034494666777

Normal mode eigenvalues : nmode = 12

-0.003527316 0.001144119 0.008864064 0.018488687 0.026244163

0.032260931 0.036858027 0.059693569 0.065335309 0.104413079

0.359347099 0.526291756

CONNECTION : 10 - 12

Geometry of TS 28, SYMMETRY = C1

C	-0.720854694624	-0.790811676704	-0.382516917093
H	-0.004693162673	-0.769901296549	-1.212336595772
C	-1.852257295899	0.875414151951	1.071384067862
H	0.043585191328	2.136518599056	0.232352249364
C	-1.054572664675	0.434874588132	0.101115001415
O	-0.744278765808	1.860215894750	-0.260190578000

Energy = -190.529758895850

Spin(**2) = 0.000000000000

ZPVE = 0.032545240800

Normal mode eigenvalues : nmode = 12

-0.005039497 0.001594466 0.004973408 0.011536424 0.018580987

0.019821559 0.033283362 0.047634837 0.050711811 0.112621342

0.355795632 0.527272283

CONNECTION : 12 - 10

Geometry of TS 29, SYMMETRY = Cs

C	-0.624591882707	-0.465604800515	-0.420700260364
H	-1.501454217585	-0.997412670861	0.281006780514
C	-1.684303571244	0.177802099934	0.742865778648
H	-0.623176891047	2.652715277734	0.343026722323
C	-0.744596836802	0.809350977612	0.005981592964
O	-0.199929405472	1.989411127060	-0.221420329457

Energy = -190.605617040601

Spin(**2) = 0.000000000000

ZPVE = 0.033771835430

Normal mode eigenvalues : nmode = 12

-0.011031192 0.009257881 0.010745883 0.011241619 0.018086813

0.031626903 0.036986831 0.057106290 0.080929545 0.121030621

0.225464662 0.528629226

CONNECTION : 12 - 13

Geometry of TS 30, SYMMETRY = Cs

C	-0.893695226773	-0.647329737012	-0.209884197296
H	-0.043784131784	0.064869722175	-0.841779030784
C	-1.601693547484	0.198405835449	0.669528466424
H	-0.605663285876	2.699515241879	0.337901520341
C	-0.678765930826	0.872623087968	-0.040983512602
O	-0.153839504742	2.064364280178	-0.246777187608

Energy = -190.570765712152

Spin(**2) = 0.000000000000

ZPVE = 0.033079365073

Normal mode eigenvalues : nmode = 12

-0.034752918 0.006383822 0.011984980 0.014268655 0.026975729

0.029350589 0.047055477 0.055097812 0.070561277 0.104568300

0.186620711 0.500735024

CONNECTION : 12 - 14

Geometry of TS 31, SYMMETRY = Cs

C	-0.704381445943	-0.313056857769	-0.032450524764
H	-0.837352279376	-1.367982010238	-0.202036349228
C	-1.082707291979	0.480470571997	1.040905159934
H	-0.985076753971	2.505005597095	0.327237210558
C	-0.796352071374	0.757932807004	-0.909619244559
O	-0.412865602156	1.716083068245	0.299007357213

Energy = -190.558524813014

Spin(**2) = 0.000000000000

ZPVE = 0.035611058176

Normal mode eigenvalues : nmode = 12

-0.000181993 0.009069758 0.017351023 0.021346937 0.031213162

0.040162164 0.040451067 0.042349335 0.071215110 0.072832568

0.403856407 0.506318949

CONNECTION : 13 - 13

Geometry of TS 32, SYMMETRY = C1

C	-0.882952642643	-0.080675783739	0.126412137274
H	-0.833338976232	-1.179114229206	-0.175958150612
C	-1.785116808554	-0.477601029511	0.951952883734
H	-0.213490275871	2.033216559860	0.823448935789
C	-0.350997786127	0.938374657112	-0.776198936583
O	-0.088679581526	2.054103970384	-0.148982179553

Energy = -190.578192033016

Spin(**2) = 0.000000000000

ZPVE = 0.032524531481

Normal mode eigenvalues : nmode = 12

-0.005695744 0.001785719 0.005301150 0.008967513 0.024981321

0.027557068 0.030828009 0.063433705 0.069012498 0.119451932

0.281386821 0.458666572

CONNECTION : 8 - 12

Geometry of TS 33, SYMMETRY = C1

C	-0.862338522994	-0.703951623748	-0.164536427651
H	-1.456870298339	-1.592667275870	-0.076701902099
C	-1.013263867555	0.950668520504	0.470907850630
H	-1.862994359941	2.592158505829	0.188084888581
C	0.053127910436	0.145642146449	-0.019482517864
O	-1.091673920355	2.123845069391	-0.158013777543

Energy = -190.584973060595

Spin(**2) = 0.000000000000

ZPVE = 0.034975962073

Normal mode eigenvalues : nmode = 12

-0.024887095 0.003066889 0.007960293 0.013460300 0.020741487

0.025973991 0.042741508 0.056715364 0.065664435 0.117132519

0.420118592 0.531826910

CONNECTION : 10 - 13

Geometry of TS 34, SYMMETRY = Cs

C	-0.803656890396	-0.087098925471	-0.007940069977
H	-1.799461128680	-0.547316905957	-0.022505676889
C	-0.502434257373	1.353592861196	0.040319864638
H	-2.439138710693	1.587458743612	0.050141525680
C	0.280385527636	-0.816725344682	-0.033523537506
O	-1.589919987284	2.083194713017	0.065994483522

Energy = -190.579761478465

Spin(**2) = 0.000000000000

ZPVE = 0.033516766823

Normal mode eigenvalues : nmode = 12

-0.003576831 0.000910948 0.009520902 0.016492608 0.025763207

0.029745023 0.037336685 0.059069547 0.067625957 0.104806213

0.345553793 0.442178646

CONNECTION : 13 - 8

Geometry of TS 35, SYMMETRY = Cs

C	-0.875867634994	-0.662182752170	-0.001338251404
H	-1.457201566390	-1.433762268145	-0.482380839529
C	-0.897689121321	0.454036003588	0.842095621256
H	-1.802757352345	2.202174553433	-0.728066545423
C	-0.065208870949	0.464013544839	-0.179974024702
O	-1.047921923584	2.122874441357	-0.114091889331

Energy = -190.485438056844

Spin(**2) = 0.000000000000

ZPVE = 0.031610589256

Normal mode eigenvalues : nmode = 12

-0.029909885 0.001678018 0.003772695 0.007428613 0.012687830

0.020284187 0.026083164 0.034379344 0.060601572 0.102623068

0.397277125 0.506757391

CONNECTION : 13 - 13

Geometry of TS 36, SYMMETRY = Cs

C	-0.962616885628	-0.695022135271	-0.028340638852
H	-1.954497219390	0.106183626792	-0.000432401362
C	-0.877039854539	0.858594835073	0.024019200092
H	-2.150813717387	2.285924645871	0.073316622801
C	0.217154770220	0.077429302452	-0.003302525229
O	-1.193108990722	2.141605665812	0.067600354392

Energy = -190.565370645764

Spin(**2) = 0.000000000000

ZPVE = 0.032772273513

Normal mode eigenvalues : nmode = 12

-0.038271511 0.006732279 0.009738344 0.012153000 0.025590694

0.027107867 0.047824698 0.056778037 0.063326571 0.105576896

0.180643339 0.524595914

CONNECTION : 15 - 13

Geometry of TS 37, SYMMETRY = C1

C	-1.007966840358	-0.278320231462	0.069797694524
H	-1.030905207713	-1.361282691743	0.103233433902
C	-1.825778652069	0.473329313663	-0.753600961106
H	-1.720368732467	1.603418661901	0.040668649592
C	0.002491435782	0.633053394336	0.425164767139
O	-0.476160266287	1.793884407889	0.437209266668

Energy = -190.529983142352

Spin(**2) = 0.000000000000

ZPVE = 0.029846563075

Normal mode eigenvalues : nmode = 12

-0.110699948 0.001735119 0.008768724 0.021252741 0.027829822

0.033055603 0.039541102 0.052177357 0.064958015 0.083914754

0.107479938 0.383457067

CONNECTION : 1 - 12

Geometry of TS 38, SYMMETRY = C1

C	-0.666520543865	-0.099596211677	0.082319693078
H	-1.006638516855	-1.182617476776	0.050828005489
C	-1.638525917618	0.950595858252	-0.203013427388
H	-1.708970317617	2.803471139152	-0.093817175140
C	0.539947966934	-0.544738619564	0.080319097683
O	-1.087038062869	2.097813074333	0.135635330246

Energy = -190.583926612086

Spin(**2) = 0.000000000000

ZPVE = 0.033241809149

Normal mode eigenvalues : nmode = 12

-0.004342993 0.000851764 0.006758329 0.009821802 0.024477941
0.028132876 0.032665867 0.062217415 0.068812027 0.122611246
0.287114724 0.526169717

CONNECTION : 13 - 10

Geometry of TS 39, SYMMETRY = C1

C	-1.577434590764	-0.909863336466	-0.072318757122
H	0.312499244874	-0.488466742958	-0.707256846463
C	-0.556931687284	-0.123694432540	-0.158725236912
H	-0.047524155653	1.283899591046	1.476283439952
C	-0.441905980498	1.270513525979	0.445843224320
O	-0.727542852678	2.263270388944	-0.146785466297

Energy = -190.659096606075

Spin(**2) = 0.000000000000

ZPVE = 0.034262781369

Normal mode eigenvalues : nmode = 12

-0.001673031 0.001940882 0.005882217 0.016487861 0.023737941
0.030656002 0.042926558 0.072224228 0.111355279 0.126438512
0.330581328 0.368726124

CONNECTION : 11 - 5

Geometry of TS 40, SYMMETRY = Cs

C	-1.298557046016	-1.250105172186	-0.000690887138
H	-0.674852942504	0.078046855249	-1.321549188186
C	-0.901241848060	-0.039127280686	-0.251910778892
H	-0.508376965723	2.218862601682	1.216587080386
C	-0.713688142581	1.137742494423	0.609062146748
O	-0.294948887779	2.231240828293	0.049777767888

Energy = -190.535873111469

Spin(**2) = 0.000000000000

ZPVE = 0.028666201079

Normal mode eigenvalues : nmode = 12

-0.157463781 0.000895833 0.001737553 0.008616676 0.010024391
0.016323776 0.024805730 0.039916522 0.071310435 0.112586450
0.256378836 0.347263404

CONNECTION : 10 - 11

Geometry of TS 41, SYMMETRY = Cs

C	-0.884426764777	-1.037398415617	-0.467233261973
H	-0.192578623010	0.643722290096	-0.714450916759
C	-0.962709118781	-0.044462233798	0.312068874460
H	-1.089604613464	1.722317560825	1.675149354477
C	-0.755802765975	1.260975865239	0.747845795135
O	-0.089609094660	1.900019711262	-0.112355722766

Energy = -190.605666371908

Spin(**2) = 0.000000000000

ZPVE = 0.031636747110

Normal mode eigenvalues : nmode = 12

-0.114439853 0.001297525 0.001592133 0.023766638 0.038019563
0.040571537 0.040712548 0.064137522 0.091875501 0.101701650
0.148310186 0.375560767

CONNECTION : 14 - 7

Geometry of TS 42, SYMMETRY = C1

C	-1.194073080792	-0.362132507819	0.138116919441
H	0.459780927264	0.437135714449	-1.156702192124
C	-0.452459932793	0.637206891488	-0.592996909371
H	-0.533497939977	1.007012184329	1.872524541522
C	-0.951191838741	0.698072884819	0.926517975785
O	-0.722626233723	1.875854887201	-0.204356444310

Energy = -190.611952114842

Spin(**2) = 0.000000000000

ZPVE = 0.035700347223

Normal mode eigenvalues : nmode = 12

-0.008265448 0.010720382 0.021932198 0.025761560 0.040265833
0.042708979 0.044743842 0.058268995 0.065039165 0.080945382
0.365928474 0.398965693

CONNECTION : 7 - 16

Geometry of TS 43, SYMMETRY = C1

C	-0.949636557873	-0.985549361550	0.052319724914
H	-1.121756746368	0.142928445137	-1.630311919263
C	-0.857706719924	0.160584005684	-0.572888279506
H	-0.421757802431	2.920092375088	0.973196457778
C	-1.087632586695	2.049678009633	0.776480119619
O	-0.363159441217	1.377847447904	-0.083518119711

Energy = -190.530516343948

Spin(**2) = 0.000000000000

ZPVE = 0.032737354079

Normal mode eigenvalues : nmode = 12

-0.000316607 0.002046533 0.007128264 0.017382207 0.022062524
0.026903720 0.044928443 0.058624179 0.075388419 0.108397727
0.309336255 0.368838195

CONNECTION : 7 - 18

Geometry of TS 44, SYMMETRY = C1

C	-0.810094796574	-0.001081681975	-0.747824644863
H	-0.439970910293	-0.720628730779	-1.448155015612
C	-1.252396349877	0.628547246084	0.248327621796
H	0.234803222562	2.361149573814	-0.268936132811
C	-1.363811512368	0.221194685401	1.510033531348
O	-0.632231175549	2.167871294001	-0.661008805464

Energy = -190.478215267915

Spin(**2) = 0.000000000000

ZPVE = 0.031055694501

Normal mode eigenvalues : nmode = 12

-0.021757103 0.000654329 0.002296212 0.005883789 0.010843580

0.012161733 0.017689578 0.022943944 0.063484888 0.134751088

0.434227186 0.525287711

CONNECTION : 15 - 12

Geometry of TS 45, SYMMETRY = Cs

C	-0.404558875715	0.521791869964	-0.659270857641
H	-0.866529847424	-0.640676922988	0.005891018417
C	-1.043286041728	0.485576911270	0.565762160906
H	-0.184661701198	2.415305200418	-0.715891576993
C	-1.615606626855	-0.079871724055	1.559684159177
O	0.008964977514	1.612513574177	-1.245242687582

Energy = -190.537551508174

Spin(**2) = 0.000000000000

ZPVE = 0.029877691908

Normal mode eigenvalues : nmode = 12

-0.102576312 0.000491498 0.002345819 0.002416408 0.010722053

0.021876699 0.035971779 0.067865099 0.071077538 0.131677404

0.175916576 0.463742166

CONNECTION : 14 - 8

Geometry of TS 46, SYMMETRY = C1

C	-0.619469496886	0.458544963199	-0.581238464796
H	-0.628341604044	-0.411811254022	-1.238642350774
C	-1.100791982187	0.395060937858	0.655371583953
H	-0.611744430203	2.131712729652	-1.580644423979
C	-1.628849032596	0.264438408784	1.805345136851
O	-0.011399860216	1.548673911802	-1.097123214885

Energy = -190.641107626004

Spin(**2) = 0.000000000000

ZPVE = 0.035729920549

Normal mode eigenvalues : nmode = 12

-0.020649005 0.001392759 0.001489135 0.014169060 0.028665059
0.041505615 0.046882085 0.061991762 0.078219594 0.161418128
0.363014815 0.538114533

CONNECTION : 15 - 14

Geometry of TS 47, SYMMETRY = C1

C	-0.571278929128	0.183926329263	-0.539417979651
H	0.060153976261	0.533855689708	-1.484522156726
C	-1.092945442153	0.342150369150	0.620999731261
H	0.500669132327	2.221840775696	-0.773938192496
C	-1.648812351992	0.316965912426	1.779533524431
O	-0.062886214820	1.816288949206	-1.451481592112

Energy = -190.523759050419

Spin(**2) = 0.000000000000

ZPVE = 0.029308425701

Normal mode eigenvalues : nmode = 12

-0.079218528 0.000851735 0.001294891 0.003458980 0.004498646
0.012562532 0.022658521 0.047728221 0.058038817 0.157312913
0.215510828 0.531093786

CONNECTION : 14 - DC

Geometry of TS 48, SYMMETRY = C1

C	-1.241018622178	0.644481517538	0.714403077114
H	-2.193896675494	0.475894620725	1.225231386387
C	-0.378734779795	-0.260651020470	0.080740389230
H	-0.247686369425	-1.328891684347	0.073242085264
C	-0.367447442426	0.670343477941	-0.933869523523
O	-0.924908415285	1.804859251434	0.246918299990

Energy = -190.633713807515

Spin(**2) = 0.000000000000

ZPVE = 0.035801245698

Normal mode eigenvalues : nmode = 12

-0.003144234 0.006801969 0.020475671 0.027933993 0.036218793

0.038538166 0.054332541 0.068012981 0.069733438 0.083144991

0.354898043 0.404147408

CONNECTION : 2 - 5

Geometry of TS 49, SYMMETRY = Cs

C	-1.432960643441	0.284173605583	0.971526752881
H	-2.000666941325	-0.156975174164	1.800141328069
C	-0.577157210522	-0.256671663987	0.057047733022
H	-0.278843877359	-1.297178632230	-0.025356597519
C	-0.212608001682	0.901237651432	-0.717483547531
O	-0.836841213730	1.809820515502	-0.193094960023

Energy = -190.585940350983

Spin(**2) = 0.000000000000

ZPVE = 0.034769782017

Normal mode eigenvalues : nmode = 12

-0.009700129 0.005906562 0.013239096 0.028073080 0.032648262

0.034886502 0.041597464 0.058012316 0.081013895 0.096260931

0.346041537 0.377151221

CONNECTION : 2 - 0

Geometry of TS 50, SYMMETRY = C1

C	-1.253341690615	0.526223836451	0.772353657991
H	-2.194931009626	-0.050744813932	0.782380887970
C	-0.363846571065	0.213909309102	-0.332410301414
H	-0.653884366021	0.150773964152	1.656473944250
C	0.094763965061	-0.155608846919	-1.455926735550
O	-1.295895218997	1.841259817827	0.859126071778

Energy = -190.510382875748

Spin(**2) = 0.000000000000

ZPVE = 0.031612830524

Normal mode eigenvalues : nmode = 12

-0.043398043 0.001640933 0.002183093 0.010528089 0.024819038

0.037589228 0.053820813 0.058028087 0.062880570 0.122859518

0.267782593 0.320975777

CONNECTION : 20 - 11

Geometry of TS 51, SYMMETRY = Cs

C	-1.775959819315	0.955889348253	1.350400757103
H	-2.306376084567	0.696189012600	2.232053535861
C	-0.372564503611	0.438963414516	-0.378192625404
H	0.965232837695	-0.906525670205	-1.598417668211
C	0.338387798235	-0.261284375602	-1.034087023175
O	-1.155364489837	1.320786490357	0.288277149191

Energy = -190.505922523223

Spin(**2) = 0.000000000000

ZPVE = 0.033230656214

Normal mode eigenvalues : nmode = 12

-0.094231773 0.001527141 0.003938647 0.013047590 0.015772770

0.015787832 0.024867925 0.025101309 0.071301383 0.187645911

0.457399062 0.459192044

CONNECTION : 9 - 6

Geometry of TS 52, SYMMETRY = Cs

C	-1.273083273554	-1.293416786122	0.100737907133
H	-1.120058688110	-1.888018224730	-0.806062663113
C	-0.938907863795	-0.013709267186	-0.019448732663
H	-0.591562065851	2.827652551814	0.946038092903
C	-0.670878236019	1.196879210285	0.017072712862
O	-0.398244134993	2.447265647217	0.067934534123

Energy = -190.628864611078

Spin(**2) = 0.000000000000

ZPVE = 0.034567793650

Normal mode eigenvalues : nmode = 12

-0.002966049 0.001680761 0.004806244 0.012075269 0.022575014

0.028584671 0.031277132 0.062353668 0.083517589 0.168862039

0.355177168 0.475801481

CONNECTION : 10 - 8

Geometry of TS 53, SYMMETRY = C1

C	-1.183039490460	-1.086278673950	-0.356660919890
H	-1.371994076051	-2.087188783366	-0.662322468549
C	-0.965758809150	0.056457371758	-0.002927213531
H	-0.974025097978	2.795301082807	-0.611807582206
C	-0.817565592412	1.287153094792	0.649909200411
O	-0.312442850414	2.268929915172	-0.145475988446

Energy = -190.621711063647

Spin(**2) = 0.000000000000

ZPVE = 0.033390559282

Normal mode eigenvalues : nmode = 12

-0.048938320 0.001853292 0.002204909 0.008774183 0.014997696

0.025554734 0.026275066 0.032073480 0.053610603 0.161669653

0.452012745 0.544811549

CONNECTION : 8 - 10

Geometry of TS 54, SYMMETRY = Cs

C	-1.088186361261	-1.152604696781	-0.239340391938
H	0.050940222490	-0.016811989614	0.606805138507
C	-0.962834088098	0.117646077696	-0.167917966962
H	-0.268748714826	3.143300714111	0.303433926743
C	-0.360283154661	1.275207398396	0.269097484711
O	-0.815295865233	2.453171362374	-0.099779719782

Energy = -190.540816200723

Spin(**2) = 0.000000000000

ZPVE = 0.030398990369

Normal mode eigenvalues : nmode = 12

-0.105392770 0.000606714 0.002630730 0.002654323 0.010828118
0.020115163 0.038637220 0.061695159 0.073121315 0.132953546
0.172350944 0.528998005

CONNECTION : 10 - 15

Geometry of TS 55, SYMMETRY = C1

C	-0.647202062873	-0.177769179079	-0.654758162329
H	-0.161990380739	-0.978066227716	-1.173494686066
C	-1.251246833770	0.569007289975	0.155108061630
H	-1.062442671535	2.293548173535	-1.451476325669
C	-1.609786707451	0.437798394407	1.431001903189
O	-0.423548195706	2.005314719126	-0.779873887196

Energy = -190.477849035565

Spin(**2) = 0.000000000000

ZPVE = 0.030886478363

Normal mode eigenvalues : nmode = 12

-0.021113880 0.000650470 0.002143358 0.005293901 0.011831252
0.012264893 0.015282649 0.021897258 0.060707297 0.137655680
0.434312440 0.526067197

CONNECTION : 15 - 12

Geometry of TS 56, SYMMETRY = Cs

C	-0.853236845958	0.764511939994	-0.582825990933
H	-1.090883368238	-0.264725634050	-1.422544213658
C	-1.303881336343	0.459956416529	0.620084690736
H	-0.622996125435	0.501592678078	-1.907654635700
C	-1.776678766776	0.043530570058	1.718977099827
O	-0.282895494630	1.647122057379	-1.261549422118

Energy = -190.535492777618

Spin(**2) = 0.000000000000

ZPVE = 0.027403093346

Normal mode eigenvalues : nmode = 12

-0.194290763 0.001037582 0.001044129 0.010634498 0.015339365
0.032392371 0.032745787 0.040356306 0.098268620 0.126475514
0.163846412 0.170362286

CONNECTION : 15 - DC

Geometry of TS 57, SYMMETRY = Cs

C	-0.718905538343	-0.738077450096	-0.023008912265
H	-0.583611942253	-1.806605702801	-0.017715167292
C	-1.573810027527	0.196858814122	-0.153739687048
H	0.339886808163	0.434586793921	0.177888865516
C	-1.253765557226	1.482037960265	-0.077426003311
O	-0.067083253646	1.765688333263	0.130498686356

Energy = -190.582104515760

Spin(**2) = 0.000000000000

ZPVE = 0.031591263006

Normal mode eigenvalues : nmode = 12

-0.105317899 0.006313316 0.008807710 0.018490257 0.022660545
0.030506316 0.042316207 0.060571741 0.088615114 0.104125443
0.125642103 0.405091587

CONNECTION : 8 - 3

Geometry of TS 58, SYMMETRY = Cs

C	-1.498532063520	-1.269685675950	-0.208219491546
H	-0.794987121790	-2.091718834230	-0.037626933941
C	-0.974448313467	-0.059813581073	-0.047126603570
H	0.628781442239	2.447994613316	0.423434851636
C	-0.672009288675	1.139616917844	0.055207765036
O	-0.310408328436	2.362702914113	0.173616975360

Energy = -190.628221402705

Spin(**2) = 0.000000000000

ZPVE = 0.034582754596

Normal mode eigenvalues : nmode = 12

-0.002948851 0.001718755 0.004830154 0.012327415 0.022142382

0.028679120 0.031720445 0.062000130 0.083253784 0.168209814

0.354480843 0.478518061

CONNECTION : 10 - 8

Geometry of TS 59, SYMMETRY = Cs

C	-0.865384108745	-1.243309727355	-0.071884771070
H	-1.117828953926	-2.270295106020	-0.201104028391
C	-0.569463342857	-0.069253500807	0.078813688673
H	0.138141695146	1.184585368510	0.396379996199
C	-1.257180015966	1.611969853761	-0.151622559981
O	-0.285499097230	2.271921803864	0.255774639162

Energy = -190.573963581837

Spin(**2) = 0.000000000000

ZPVE = 0.028611979084

Normal mode eigenvalues : nmode = 12

-0.208174317 0.000542542 0.001364003 0.005205539 0.014625378

0.017018621 0.021084192 0.034657018 0.086882455 0.131815102

0.161569198 0.446602410

CONNECTION : 8 - DC

Geometry of TS 60, SYMMETRY = C1

C	-0.585263514221	-0.942973502492	-0.052678483494
H	-1.489012589955	-1.516572756607	-0.290856162635
C	-0.828681730033	0.384970233342	-0.175848144338
H	0.460613317689	1.470017311089	0.961083596347
C	-1.712294703568	1.359177094585	0.021795453915
O	0.166282880844	1.497957612796	0.035702719761

Energy = -190.531073718034

Spin(**2) = 0.000000000000

ZPVE = 0.032790800195

Normal mode eigenvalues : nmode = 12

-0.004056145 0.001916587 0.006515350 0.015048939 0.016729961

0.019127464 0.036490604 0.048351078 0.051907670 0.108353584

0.355890783 0.519446725

CONNECTION : 10 - 13

Geometry of TS 61, SYMMETRY = Cs

C	-0.778350487703	-1.041958123823	-0.043994851741
H	-1.239664006656	-2.000995776602	-0.116590584247
C	-0.891497354131	0.179331270661	-0.047957488946
H	0.738367840577	1.487552546979	0.188282120864
C	-1.372457224472	2.253706424937	-0.094737817248
O	-0.274890150561	1.368741547026	0.048109542822

Energy = -190.421962736100

Spin(**2) = 0.000000000000

ZPVE = 0.029793758886

Normal mode eigenvalues : nmode = 12

-0.003829606 0.000772843 0.002847909 0.006082275 0.012238315

0.018051252 0.018863557 0.034006259 0.067817509 0.163389105

0.241963652 0.437831544

CONNECTION : 22 - 10

Geometry of TS 62, SYMMETRY = Cs

C	-1.828063563978	1.357713269055	-0.267901165136
H	0.102836508077	-1.682968980662	0.424646725448
C	0.145106055439	-0.603670714454	0.310677282169
H	1.104722211431	-0.119959693853	0.430057879952
C	-0.998900082517	-0.037618016911	0.039592770882
O	-0.709483526892	1.779452359865	-0.112727069919

Energy = -190.633059941707

Spin(**2) = 0.000000000000

ZPVE = 0.033457156303

Normal mode eigenvalues : nmode = 12

-0.007383643 0.000831790 0.004758431 0.015231214 0.015596489

0.030653680 0.030706845 0.071072581 0.104084572 0.113088508

0.370628271 0.392119473

CONNECTION : 4 - DC

Geometry of TS 63, SYMMETRY = Cs

C	-1.673744985493	1.350074376576	-0.451721453827
H	0.586643887516	-1.388790076795	0.795575151094
C	-0.541398955598	-1.051236263385	0.012075257997
H	0.912484136117	-0.719317686068	1.141941907918
C	-0.886863882283	0.201791787417	-0.047988306889
O	-0.654265437266	1.567440065088	0.338152569360

Energy = -190.493412476240

Spin(**2) = 0.000000000000

ZPVE = 0.024832465856

Normal mode eigenvalues : nmode = 12

-0.015949170 0.002773132 0.003599930 0.007465168 0.009812700

0.010399947 0.018974958 0.030790574 0.047502343 0.060507418

0.122687400 0.351574196

CONNECTION : 4 - DC

Geometry of TS 64, SYMMETRY = C1

C	-1.496630956837	1.218513823452	-0.638972976050
H	0.223003004856	-0.810636115527	1.259212847094
C	-0.032595116126	-0.257026458321	0.358150600769
H	0.775702862469	-0.090771753222	-0.342356323699
C	-1.398875999473	-0.023681119074	0.002723562629
O	-0.567460616548	1.419427624794	0.344790011077

Energy = -190.543939322784

Spin(**2) = 0.000000000000

ZPVE = 0.034580453740

Normal mode eigenvalues : nmode = 12

-0.016577089 0.005627470 0.010341024 0.026830402 0.030030973

0.041148980 0.047575465 0.052740537 0.072682303 0.086064142

0.363304268 0.387769042

CONNECTION : 20 - DC

Geometry of TS 65, SYMMETRY = C1

C	-1.546042841286	0.645909792705	-0.602837810392
H	-0.339934303377	-1.486204411147	0.263746013508
C	-0.296803843524	-0.451384538906	-0.061922608623
H	0.200666852921	-0.312773687082	-1.022153048356
C	-0.347045347812	0.587575304184	0.902823732615
O	-0.891876724719	1.599287481171	-0.078952586696

Energy = -190.574016394853

Spin(**2) = 0.000000000000

ZPVE = 0.034638288022

Normal mode eigenvalues : nmode = 12

-0.006184982 0.012805201 0.014561635 0.019010013 0.030084203

0.038108682 0.040818444 0.057474064 0.074544656 0.083433478

0.356727149 0.380069008

CONNECTION : 23 - DC

Geometry of TS 66, SYMMETRY = C1

C	-1.350408971428	0.903133680963	-0.430950684306
H	-0.800982835789	-0.806364666350	0.823737731634
C	-0.059002441386	0.313320063059	0.474650649524
H	0.992071065610	0.147791851862	0.242179957945
C	-1.247445395494	-0.440725617343	-0.257318758289
O	-0.559594607016	1.500121222850	0.714635780330

Energy = -190.525046805558

Spin(**2) = 0.000000000000

ZPVE = 0.031159955666

Normal mode eigenvalues : nmode = 12

-0.055523525 0.005256551 0.011680860 0.016210116 0.024254152

0.034416032 0.036334253 0.054161298 0.066134679 0.075914937

0.202194176 0.367140791

CONNECTION : 2 - 20

Geometry of TS 67, SYMMETRY = Cs

C	-1.497279493946	0.264952934238	-0.315879669255
H	-2.464249811858	-0.010000861031	-0.726502704568
C	-0.335615763459	-0.239163860062	0.069909453349
H	0.120955172220	-1.210555484741	0.121959574249
C	0.073075225603	1.196653856539	0.413207009951
O	-1.163080284728	1.644801172416	-0.008582727013

Energy = -190.620861750671

Spin(**2) = 0.000000000000

ZPVE = 0.035437065555

Normal mode eigenvalues : nmode = 12

-0.003128708 0.013248416 0.021573335 0.026274305 0.031864707

0.034546321 0.039096653 0.051725803 0.059927816 0.098822014

0.376324872 0.409305291

CONNECTION : 2 - 2

Geometry of TS 68, SYMMETRY = C1

C	-1.509877893432	0.329409789856	-0.133122655474
H	-2.545279175451	0.053340659947	-0.063207824238
C	-0.191762767785	-0.099749284754	-0.197213129967
H	0.489795460275	-0.806223030059	-0.656619834518
C	-0.385699489334	0.838166663088	0.875533707485
O	-0.943719924974	1.568616067582	-0.532656330759

Energy = -190.542127118864

Spin(**2) = 0.000000000000

ZPVE = 0.034090490714

Normal mode eigenvalues : nmode = 12

-0.027572531 0.008084139 0.015264190 0.019838599 0.031141024

0.031387771 0.040249817 0.049849779 0.056321208 0.080160939

0.382817531 0.409599764

CONNECTION : 2 - 25

Geometry of TS 69, SYMMETRY = C1

C	-1.598198290444	0.354756085532	-0.393984630189
H	-1.484193553861	-0.413144629886	0.728545528239
C	-0.382593118344	-0.236542422641	0.033370301876
H	0.050028572255	-1.224506726151	0.016611380451
C	-0.068577172475	1.131768835733	0.603116421323
O	-1.038995628400	1.679043781246	-0.171830354026

Energy = -190.518531807177

Spin(**2) = 0.000000000000

ZPVE = 0.031239805376

Normal mode eigenvalues : nmode = 12

-0.065853917 0.006101210 0.019746835 0.024558311 0.026902676

0.033127328 0.041318105 0.050498536 0.056178602 0.065995271

0.153759241 0.399661616

CONNECTION : 2 - 23

Geometry of TS 70, SYMMETRY = Cs

C	-1.112615744290	0.908942571271	-0.055356446082
H	-1.656955492667	-0.200819193318	-0.306880822853
C	-0.226991557869	-0.497528758881	0.211056437074
H	-0.286384418670	-1.574182658138	0.141097442758
C	0.234961999032	0.696402422504	0.435735871957
O	-1.874836983757	1.802308392216	-0.298763024055

Energy = -190.603897860879

Spin(**2) = 0.000000000000

ZPVE = 0.031778216441

Normal mode eigenvalues : nmode = 12

-0.037040707 0.002698831 0.009251657 0.015438176 0.019863279

0.028624868 0.044945082 0.067495349 0.100378074 0.115376050

0.139169563 0.396520166

CONNECTION : 7 - 3

Geometry of TS 71, SYMMETRY = Cs

C	-1.333343553580	-0.051617964338	-0.412904263341
H	-2.075550288901	-0.093144290304	-1.191995673264
C	-0.576430723209	-0.703913276006	0.356657345743
H	-0.297597851680	-1.707229195077	0.612751560150
C	-1.029808773106	1.538230010260	-0.037748010946
O	-0.454181663563	2.297184196687	0.592688180374

Energy = -190.666619415810

Spin(**2) = 0.000000000000

ZPVE = 0.033605011753

Normal mode eigenvalues : nmode = 12

-0.017185572 0.002065230 0.003134995 0.010885841 0.013527041

0.019056255 0.027255159 0.055522501 0.110532271 0.162100275

0.391676538 0.419627823

CONNECTION : 0 - DC

Geometry of TS 72, SYMMETRY = C1

C	-1.555755548119	-0.384988603784	-0.896726530741
H	-2.582041589997	-0.525489821208	-0.503279479622
C	-0.756355774556	0.160137310642	0.218350892159
H	-0.688438706258	-0.502509026893	1.086191752621
C	0.170465157242	1.303529699533	0.049274675331
O	-0.974219158786	1.591575925290	0.580094550568

Energy = -190.523912407337

Spin(**2) = 0.000000000000

ZPVE = 0.031859778792

Normal mode eigenvalues : nmode = 12

-0.028206026 0.002675269 0.005181802 0.018651298 0.024180863

0.032123311 0.040385377 0.052183915 0.062170834 0.080779986

0.325586481 0.350413393

CONNECTION : DC - DC

Geometry of TS 73, SYMMETRY = Cs

C	-0.637378843839	-0.340822928942	0.412899569863
H	-1.343148711679	0.540033015462	-1.757360465513
C	-1.018580350540	0.510405233096	-0.734230777975
H	-2.063161958052	1.506373751705	1.147100608004
C	-1.344030174127	0.947198750240	0.578604304805
O	-0.162667142499	1.520273990410	-0.088624879955

Energy = -190.567481071995

Spin(**2) = 0.000000000000

ZPVE = 0.034871394880

Normal mode eigenvalues : nmode = 12

-0.008736863 0.015657061 0.021392496 0.024758707 0.030619909

0.035764866 0.038587128 0.042142961 0.050814775 0.076268657

0.408097077 0.413079246

CONNECTION : 25 - 16

Geometry of TS 74, SYMMETRY = Cs

C	-1.359287795070	-0.187598471040	1.538994927294
H	-0.145634033159	1.559817436260	-2.203559850897
C	-0.402442959526	0.722823503141	-1.521920846549
H	-1.308692672489	1.216362128837	1.697203785029
C	-1.043954702874	0.673029937043	0.662580794256
O	-0.682117976900	1.394476667036	-0.405904301898

Energy = -190.528081902554

Spin(**2) = 0.000000000000

ZPVE = 0.030344560598

Normal mode eigenvalues : nmode = 12

-0.003679056 0.000839431 0.005121534 0.005707267 0.020279742

0.024000277 0.031589243 0.057395622 0.077240077 0.145908392

0.226118975 0.319227107

CONNECTION : 6 - 7

Geometry of TS 75, SYMMETRY = C1

C	-2.577532137010	0.233187779865	-0.020395493205
H	-3.605768019467	-0.025057142854	0.018222626159
C	1.067790040238	1.132357950715	0.185251049127
H	1.076191339610	2.247403552144	0.080450873263
C	-1.413864836983	0.529314813389	-0.055636139229
O	-0.182228283661	0.811953953040	-0.110972008757

Energy = -190.570876556317

Spin(**2) = 0.000000000000

ZPVE = 0.032270340214

Normal mode eigenvalues : nmode = 12

-0.011248574 0.000122322 0.004279522 0.006184398 0.008613080

0.016953182 0.027378299 0.070005374 0.083895911 0.193464738

0.291608162 0.459553385

CONNECTION : 6 - 9

Geometry of TS 76, SYMMETRY = Cs

C	-2.585067630793	0.137721838872	-0.025952930414
H	-1.427674958783	-0.630675208546	-0.011783655054
C	0.879761774238	0.723194961013	0.054134445821
H	1.567863780398	1.591277921315	0.079867254194
C	-1.402023594448	0.582915349902	0.004600646070
O	-0.300366086156	1.333172268580	0.037435684998

Energy = -190.531663004024

Spin(**2) = 0.000000000000

ZPVE = 0.030291778452

Normal mode eigenvalues : nmode = 12

-0.005456877 0.001760552 0.004020427 0.005780946 0.020195152
0.021126936 0.033443129 0.057765119 0.077310167 0.150029999
0.212259299 0.323142628

CONNECTION : 18 - 6

Geometry of TS 77, SYMMETRY = Cs

C	-1.135712927671	0.099921914912	0.683033068785
H	-2.141440150238	-0.270491233152	0.542643352416
C	-0.517665786215	1.003871171571	-0.408357602650
H	-0.648390043772	0.040910230062	1.646068151570
C	-0.107400100268	-0.188210017911	-0.627061532551
O	-1.237545172374	1.746130071054	0.356232618639

Energy = -190.589708952382

Spin(**2) = 0.000000000000

ZPVE = 0.036307613476

Normal mode eigenvalues : nmode = 12

-0.009638049 0.007375276 0.008835009 0.031137196 0.039674981
0.041984052 0.045356573 0.057705609 0.081413299 0.125967759
0.372283993 0.404904920

CONNECTION : 3 - 20

Geometry of TS 78, SYMMETRY = C1

C	-1.534526639117	0.563928880860	1.179761168392
H	-2.144320118481	-0.197428110585	0.713206924563
C	-0.537315777000	0.774098079149	-0.567086334032
H	-1.320696147094	0.593774414067	2.243076507551
C	0.154325686728	-0.041475133014	-1.235722261005
O	-1.043217429139	1.530933109559	0.443935542932

Energy = -190.544946071873

Spin(**2) = 0.000000000000

ZPVE = 0.034715550295

Normal mode eigenvalues : nmode = 12

-0.019932536 0.001055525 0.002246746 0.020056621 0.029846405

0.031521155 0.054911961 0.061426637 0.086807505 0.140165195

0.366690484 0.404273563

CONNECTION : 20 - 24

Geometry of TS 79, SYMMETRY = C1

C	-1.405558597698	0.568580667121	1.138853002552
H	-1.671369499905	-0.170966252299	-0.304711434644
C	-0.774618496015	0.554219073572	-0.504093165661
H	-0.580022683255	0.222063750272	1.789488625312
C	0.145395631844	0.148414101476	-1.332086859236
O	-0.958796079264	1.624191451029	0.364617903588

Energy = -190.482900284012

Spin(**2) = 0.000000000000

ZPVE = 0.029643127755

Normal mode eigenvalues : nmode = 12

-0.021970271 0.001393563 0.002357533 0.009862309 0.019368080

0.024959861 0.039622123 0.047140936 0.062712090 0.113300094

0.227529555 0.323416461

CONNECTION : 5 - 20

Geometry of TS 80, SYMMETRY = C1

C	-1.384694224840	0.192929847628	0.967980947541
H	-2.431262737772	0.152382996399	0.642090229342
C	-0.505350966066	0.560752237407	-0.142719078654
H	-0.842574356993	1.391494117721	1.486231274429
C	0.062130161506	0.128531906459	-1.241780776821
O	-0.708898611523	1.810870733284	0.435961467406

Energy = -190.484663355034

Spin(**2) = 0.000000000000

ZPVE = 0.029998017054

Normal mode eigenvalues : nmode = 12

-0.069736595 0.002194663 0.005959732 0.008241393 0.028165990
0.032870549 0.037306682 0.042040344 0.052522189 0.113181662
0.193951684 0.352127014

CONNECTION : 27 - 20

Geometry of TS 81, SYMMETRY = C1

C	-1.223311492315	0.194682672960	0.681387237277
H	-2.185240878456	-0.314674479331	0.612151221219
C	0.012497634716	1.174956289370	-0.458726221889
H	-0.694768557548	0.044107649984	1.615258198884
C	-0.380686387362	-0.061964008854	-0.669880284263
O	-1.261923230515	1.527209536691	0.230091138356

Energy = -190.542612481873

Spin(**2) = 0.000000000000

ZPVE = 0.034822364400

Normal mode eigenvalues : nmode = 12

-0.014933975 0.004842574 0.015522842 0.023193207 0.033595704
0.044130907 0.050342551 0.054351518 0.071691096 0.085815640
0.355391724 0.383555025

CONNECTION : 20 - 20

Geometry of TS 82, SYMMETRY = Cs

C	-1.158434668523	1.107297348313	0.561144561273
H	-1.008869979185	-0.444327790858	0.615271087946
C	-0.496127087452	0.443408864494	-0.451165530514
H	-1.513146210479	0.049906154765	1.388308695411
C	0.019322663490	-0.469613071022	-1.159054383421
O	-1.512345889136	2.150864228575	0.963149833255

Energy = -190.549378715173

Spin(**2) = 0.000000000000

ZPVE = 0.025827480871

Normal mode eigenvalues : nmode = 12

-0.155668147 0.000854696 0.001708026 0.006245218 0.013042236

0.023440522 0.032036292 0.042310748 0.074834159 0.126000402

0.139555057 0.161404711

CONNECTION : 7 - DC

Geometry of TS 83, SYMMETRY = C1

C	-1.298666149974	0.123517367481	0.385916692970
H	-2.143892656735	-0.416106978635	0.780162515058
C	0.144303203078	0.935033881393	0.323334796216
H	-0.472203151240	0.324674253764	1.368876015946
C	-0.465671151329	0.177114092064	-0.820295004558
O	-1.262287041250	1.466579401315	-0.061877242755

Energy = -190.499096777715

Spin(**2) = 0.000000000000

ZPVE = 0.031390721692

Normal mode eigenvalues : nmode = 12

-0.049701974 0.009242846 0.012137769 0.027959804 0.030503089

0.037151705 0.039456205 0.045544072 0.057398969 0.064496458

0.160518714 0.401087280

CONNECTION : 25 - 20

Geometry of TS 84, SYMMETRY = Cs

C	-1.114536968028	-0.570682729689	0.006218775403
H	-1.403038779582	-1.601438043983	0.097829321327
C	-0.705466526083	0.337883032943	-0.813454784093
H	-1.138633939128	0.304554218170	1.211472056801
C	-0.754913582153	1.658294934497	1.066092037015
O	-0.529931490476	1.531301246255	-0.164282984358

Energy = -190.516867068022

Spin(**2) = 0.000000000000

ZPVE = 0.029775558717

Normal mode eigenvalues : nmode = 12

-0.082373767 0.003613744 0.007002480 0.014417261 0.024410290

0.030720974 0.036742439 0.043871886 0.071609320 0.094703872

0.106423100 0.415342292

CONNECTION : 9 - DC

Geometry of TS 85, SYMMETRY = Cs

C	-0.908056522402	-0.867042283393	-0.643598470625
H	-1.460165934481	-1.744353205479	-0.333409774259
C	-0.953793368933	0.246196976853	-0.004989575181
H	-1.566818747697	0.607700199872	1.029718363990
C	-0.913181303541	2.014290170063	0.852661088004
O	-0.359519805392	1.509850369405	-0.173820134363

Energy = -190.516238248793

Spin(**2) = 0.000000000000

ZPVE = 0.029782364445

Normal mode eigenvalues : nmode = 12

-0.042002766 0.001986708 0.006081293 0.014870465 0.022769819

0.026432168 0.026748629 0.032824518 0.074630864 0.115744718

0.160937703 0.391705157

CONNECTION : 9 - DC

Geometry of TS 86, SYMMETRY = C1

C	-1.123755172223	-0.809411223842	-0.392367737754
H	-1.470872838362	-1.811154975856	-0.448623729487
C	-0.761957071963	0.338991648299	-0.380667014499
H	-0.083548570473	1.550317614023	1.699550207551
C	-0.961851842600	1.728033689992	1.033129475925
O	-0.365556543125	1.579364190457	-0.267384177840

Energy = -190.567033728426

Spin(**2) = 0.000000000000

ZPVE = 0.031920369112

Normal mode eigenvalues : nmode = 12

-0.005631961 0.001197538 0.005322846 0.006319941 0.010124627
0.018084511 0.030489606 0.043795742 0.070417537 0.182485899
0.303222728 0.457582840

CONNECTION : 6 - 9

Geometry of TS 87, SYMMETRY = C1

C	-1.166383737014	-1.025003800316	-0.292462425270
H	-0.502283469782	-0.334716982491	-1.397329163574
C	-0.760841219227	0.178947767024	-0.368137826065
H	-1.145047801177	1.292383929205	1.678711745215
C	-0.689793655574	2.066884296082	1.020026141053
O	-0.453407867941	1.480809118003	-0.145271358986

Energy = -190.524273708223

Spin(**2) = 0.000000000000

ZPVE = 0.029930999418

Normal mode eigenvalues : nmode = 12

-0.001124697 0.000003421 0.005168783 0.005892210 0.020152290
0.024832267 0.029798698 0.055034350 0.077186116 0.138482920
0.244945353 0.306569873

CONNECTION : 9 - DC

Geometry of TS 88, SYMMETRY = C2v

C	-1.480050495006	0.532744690848	-0.761478755423
H	-0.998014153994	-1.170813819473	0.599060773399
C	-0.524285419914	-0.386007505708	0.003482477777
H	0.263904130280	-0.851495876235	-0.594131433521
C	-0.116266716060	0.873243135220	0.771961537672
O	-1.020028008924	1.585272668167	0.006656594674

Energy = -190.561655879013

Spin(**2) = 0.000000000000

ZPVE = 0.034695013812

Normal mode eigenvalues : nmode = 12

-0.010998651 0.017353786 0.023765652 0.033225658 0.033939643
0.035600633 0.037105150 0.049635430 0.057818182 0.073414614
0.352832939 0.360872491

CONNECTION : 23 - 23

Geometry of TS 89, SYMMETRY = C1

C	-1.460930780645	0.558165127610	-0.743880072489
H	-0.552201950765	-1.480520179123	0.531866803358
C	-0.419221675854	-0.444243271202	0.283567302247
H	-0.502839885910	-0.094838813031	-1.170888192923
C	-0.450795300697	0.782439243908	0.769023362510
O	-0.735576632694	1.658224235855	-0.207170661794

Energy = -190.523932163997

Spin(**2) = 0.000000000000

ZPVE = 0.031148856512

Normal mode eigenvalues : nmode = 12

-0.046875136 0.002255388 0.014446097 0.014999946 0.027178465
0.033510543 0.041892951 0.056204921 0.058859183 0.086744293
0.157570994 0.417195797

CONNECTION : 2 - 23

Geometry of TS 90, SYMMETRY = C1

C	-1.277747018865	0.436736176183	-0.887656280940
H	-0.289994796811	-1.629731193821	0.070337757976
C	-0.841171301265	-0.242556044505	0.214998561908
H	0.175867280240	-1.632845459300	-0.580839721829
C	-0.036461113642	1.029844042673	0.615690281404
O	-1.065948790205	1.653347912076	0.159056591066

Energy = -190.448933806658

Spin(**2) = 0.000000000000

ZPVE = 0.023766579627

Normal mode eigenvalues : nmode = 12

-0.013830879 0.002077485 0.004267103 0.005955306 0.009135045

0.012646121 0.014165905 0.026176232 0.031385067 0.064996652

0.068137964 0.434796586

CONNECTION : 23 - DC

Geometry of TS 91, SYMMETRY = C2v

C	-2.572304267495	1.513539787926	-0.420364723164
H	0.794020815147	-1.179039679836	0.493823203508
C	0.759913404459	-0.101797413492	0.365577247207
H	1.634765051542	0.534815927063	0.451716577087
C	-1.460737821477	0.974693211489	-0.158176464160
O	-0.342803625664	0.432760320854	0.105497032604

Energy = -190.547909666900

Spin(**2) = 0.000000000000

ZPVE = 0.034427814049

Normal mode eigenvalues : nmode = 12

-0.008082029 0.000771694 0.002300561 0.005366039 0.028307491

0.028920105 0.055295048 0.088158872 0.096624714 0.146859156

0.359792130 0.392564684

CONNECTION : 24 - 24

Geometry of TS 92, SYMMETRY = Cs

C	-2.587111022242	0.977896877512	-0.303688503430
H	-0.967644492841	-0.511974447150	0.001603056508
C	0.473450644878	-0.158259250092	0.176749072661
H	1.565339198411	-0.148757885618	0.324295619447
C	-1.341715406035	0.838494082621	-0.126723120294
O	0.006046745669	1.039508577770	0.044534685363

Energy = -190.495061858206

Spin(**2) = 0.000000000000

ZPVE = 0.029839398552

Normal mode eigenvalues : nmode = 12

-0.098528853 0.000714352 0.001374936 0.016626257 0.020724346
0.025643101 0.041726422 0.063787642 0.074452444 0.128112664
0.145863333 0.338280900

CONNECTION : 24 - 18

Geometry of TS 93, SYMMETRY = Cs

C	-1.710188028743	0.482946007801	-0.266198803340
H	-0.156761151481	-0.973546080284	-0.624971072966
C	-0.087301163775	-0.151484324847	0.079032812933
H	0.551336722758	-0.292337187454	0.944211632479
C	-1.190143121411	0.983254322948	0.886247970691
O	-0.161161703341	1.121902801419	-0.440429949422

Energy = -190.533816459468

Spin(**2) = 0.000000000000

ZPVE = 0.034203383121

Normal mode eigenvalues : nmode = 12

-0.003859046 0.005343883 0.010903678 0.014382922 0.029116721
0.043520434 0.047489693 0.051588287 0.078967460 0.082958420
0.366676105 0.389774177

CONNECTION : 20 - 20

Geometry of TS 94, SYMMETRY = C2v

C	-1.612145945323	1.666024688710	-0.264635144112
H	0.805019021361	-0.901673024328	0.465310857784
C	0.647363244725	0.163357703934	0.314953692420
H	1.448121996745	0.896072310918	0.375519181606
C	-2.069834030606	0.386114897384	-0.200664211062
O	-0.500298036386	0.561299281709	0.062381331707

Energy = -190.498847165792

Spin(**2) = 0.000000000000

ZPVE = 0.033280046633

Normal mode eigenvalues : nmode = 12

-0.002809945 0.003231324 0.006592693 0.008192365 0.009610132

0.034920630 0.056430198 0.074595358 0.085246564 0.092064378

0.357500240 0.389842756

CONNECTION : 24 - 24

Geometry of TS 95, SYMMETRY = Cs

C	-1.454863167056	0.325207039457	0.298807843104
H	-2.502110773347	0.134433184312	0.433443314120
C	-0.357401865383	0.010241788034	-0.592169807484
H	0.574751584049	0.295959416275	0.296331316921
C	-0.341283626396	0.998996020374	0.933957426139
O	-0.887979734735	1.466736418728	-0.446775608298

Energy = -190.532898419402

Spin(**2) = 0.000000000000

ZPVE = 0.031776870878

Normal mode eigenvalues : nmode = 12

-0.031293507 0.009226041 0.014800428 0.022543101 0.028626165

0.032274203 0.040917067 0.049547162 0.059934026 0.069586317

0.176324440 0.413915984

CONNECTION : 25 - 25

Geometry of TS 96, SYMMETRY = Cs

C	-0.651747269361	-0.742451613509	0.311836463148
H	-1.749159628186	0.039509679939	-1.520852435952
C	-1.249866499363	0.040476319046	-0.561693347390
H	-0.516973732529	2.296918196434	1.638747122709
C	-1.045051728108	2.405470705359	0.662665101179
O	-1.118914094583	1.253092173781	0.115823920008

Energy = -190.496154048906

Spin(**2) = 0.000000000000

ZPVE = 0.031726876831

Normal mode eigenvalues : nmode = 12

-0.007751067 0.000293624 0.002821661 0.008558438 0.021676494

0.026413658 0.044608630 0.060785503 0.084893576 0.100788929

0.299755867 0.387589662

CONNECTION : 18 - DC

Geometry of TS 97, SYMMETRY = C1

C	-0.571462399895	-0.486578931564	-0.756932124052
H	-2.372811260749	0.101763152754	0.349767998487
C	-1.334188075541	0.224285729877	0.071266801153
H	-0.052714225394	2.561426992410	-0.601577256838
C	-0.967299112015	2.103679819067	-0.159054463922
O	-0.512833931212	1.169473641519	0.729837404082

Energy = -190.498490507783

Spin(**2) = 0.000000000000

ZPVE = 0.031892834652

Normal mode eigenvalues : nmode = 12

-0.010172089 0.000982705 0.003633157 0.014731091 0.021325785

0.036328534 0.045827762 0.047616208 0.067386567 0.094235380

0.305706209 0.386834236

CONNECTION : 25 - 18

Geometry of TS 98, SYMMETRY = C1

C	-1.166579315636	-0.019843724798	0.178887378858
H	-0.733388073351	0.274998815588	-1.967841197043
C	-0.400027456757	0.228377912197	-0.940999729580
H	-1.567925816247	1.502531724452	1.769621090669
C	-0.917262021329	0.925276974888	1.134236624504
O	-0.622758475435	1.526520001159	-0.197883189299

Energy = -190.535568639723

Spin(**2) = 0.000000000000

ZPVE = 0.033024882658

Normal mode eigenvalues : nmode = 12

-0.000005206 0.004302537 0.011127955 0.012804715 0.022167709

0.031239033 0.040849123 0.050639907 0.063526044 0.071809936

0.393809953 0.403778496

CONNECTION : 26 - 26

Geometry of TS 99, SYMMETRY = C1

C	-1.194854739048	-0.115463262630	0.161240039848
H	-0.435037855660	0.248363864063	-1.980592241046
C	-0.498166734189	0.272215895037	-0.912222096689
H	-1.578772759005	1.544646173537	1.669561481853
C	-0.832624864125	0.975408857704	1.116652532915
O	-0.625154614188	1.538533784714	-0.170447799975

Energy = -190.533469497060

Spin(**2) = 0.000000000000

ZPVE = 0.032868010653

Normal mode eigenvalues : nmode = 12

-0.014046143 0.001271222 0.008912563 0.017869850 0.024810171

0.031208666 0.041153380 0.048179487 0.058192793 0.082568174

0.368689291 0.423243109

CONNECTION : 26 - 7

Geometry of TS 100, SYMMETRY = C1

C	-1.193275882829	-0.075949665896	0.218211394961
H	-0.432834468045	0.226240440718	-1.964921871851
C	-0.439163475468	0.316628652397	-0.893599226868
H	-1.663549651262	1.464003548061	1.665948125534
C	-0.895267191288	0.893284459328	1.155675828487
O	-0.590152634037	1.531364988360	-0.266888897623

Energy = -190.533473508817

Spin(**2) = 0.000000000000

ZPVE = 0.032692123943

Normal mode eigenvalues : nmode = 12

-0.023240518 0.001301332 0.008068722 0.012801465 0.022244094

0.036552230 0.043839250 0.054539384 0.063856455 0.067195939

0.381189717 0.409581730

CONNECTION : 26 - 7

Geometry of TS 101, SYMMETRY = C1

C	-1.043806840937	0.106550412357	0.210637305844
H	-0.626644225269	0.025784516344	-2.004937705038
C	-0.501132891237	0.263292580863	-0.967004496890
H	-1.890991466802	1.378498626845	1.578154723867
C	-1.004508584373	0.761309934092	1.402920363482
O	-0.531971668802	1.578281353241	-0.382072015630

Energy = -190.531321419162

Spin(**2) = 0.000000000000

ZPVE = 0.032222192395

Normal mode eigenvalues : nmode = 12

-0.006438958 0.003296044 0.004790374 0.007862768 0.024879089

0.029314782 0.032295928 0.045204768 0.056931983 0.108944987

0.359355892 0.418311917

CONNECTION : 26 - 7

Geometry of TS 102, SYMMETRY = C1

C	-1.080071625448	-0.014009842585	0.184043547501
H	-0.024203953289	0.019428346785	-1.866007579243
C	-0.574626364788	0.259916772390	-0.983319696523
H	-1.836061507936	1.399175626947	1.579745899819
C	-0.931742946185	0.908877788726	1.219553200736
O	-0.575583192047	1.537546244325	-0.230755453755

Energy = -190.535072488618

Spin(**2) = 0.000000000000

ZPVE = 0.033203151431

Normal mode eigenvalues : nmode = 12

-0.001236476 0.003371171 0.006806204 0.014189737 0.025483075

0.029854331 0.042551315 0.043780487 0.056268028 0.106293932

0.368104636 0.432526270

CONNECTION : 26 - 17

Geometry of TS 103, SYMMETRY = C1

C	-1.290814206444	0.174590417399	1.131519937932
H	-1.022395857349	1.420374723413	-1.940305595549
C	-0.524812006967	0.893971706624	-1.140063997312
H	-0.732106747655	-0.416179695233	1.854414116199
C	-1.040004147150	0.151053404209	-0.174269987268
O	-0.311125634468	1.543167416442	0.185338947464

Energy = -190.532012887623

Spin(**2) = 0.000000000000

ZPVE = 0.032282322580

Normal mode eigenvalues : nmode = 12

-0.005561000 0.003772924 0.005103832 0.008228789 0.023561676

0.028260036 0.031719517 0.044073800 0.059413739 0.114102471

0.376305749 0.394898618

CONNECTION : 17 - 7

Geometry of TS 104, SYMMETRY = C1

C	-1.029560945247	0.229455334467	1.465527710064
H	-1.042076191812	1.265371708532	-2.254515120051
C	-0.952638756031	0.589831858882	-1.426482729160
H	-0.483446189200	-0.487196065333	0.456203117002
C	-0.636620629555	0.600420666742	-0.214871606405
O	-0.606956878977	1.373892751804	1.030314232743

Energy = -190.501242725236

Spin(**2) = 0.000000000000

ZPVE = 0.029128114454

Normal mode eigenvalues : nmode = 12

-0.054977634 0.001851991 0.004314632 0.005403973 0.012159236
0.022858472 0.030055722 0.039784459 0.069070811 0.127193602
0.163128981 0.419130060

CONNECTION : 9 - DC

Geometry of TS 105, SYMMETRY = C2

C	-1.173354530551	-0.038297010379	1.037682439325
H	-1.214423397478	0.739747133010	-2.186697790150
C	-0.407974412133	0.417638818202	-1.522775225021
H	-0.383714984600	-0.429933481936	1.684209530567
C	-0.746963026510	0.370486179760	-0.197285900292
O	-0.445344333594	1.618534364105	0.115117923394

Energy = -190.528796743275

Spin(**2) = 0.000000000000

ZPVE = 0.030997135134

Normal mode eigenvalues : nmode = 12

-0.002757300 0.001711018 0.004041961 0.017183557 0.021359641
0.024325745 0.030530971 0.032252387 0.054611661 0.097267042
0.365153433 0.365484832

CONNECTION : 7 - 7

Geometry of TS 106, SYMMETRY = C1

C	-1.426928365854	0.097768524400	0.968783998942
H	-2.470724957458	0.056392659456	0.612205556235
C	-0.565558656248	0.545535734409	-0.107946755323
H	-0.101118203184	1.911633182167	1.194177758245
C	0.045236329846	0.165483734856	-1.212502824035
O	-0.622758044953	1.873953545667	0.373675931452

Energy = -190.497867099979

Spin(**2) = 0.000000000000

ZPVE = 0.032370448182

Normal mode eigenvalues : nmode = 12

-0.005208564 0.001777527 0.005579403 0.008307311 0.014169010

0.032817848 0.034455937 0.043071423 0.058621628 0.105438137

0.336747061 0.512156989

CONNECTION : 13 - 27

Geometry of TS 107, SYMMETRY = C1

C	-1.463978289927	-0.034251777116	0.959070019065
H	-2.474227592668	0.369739251281	0.775059076039
C	-0.646800709014	0.514633821151	-0.092176809632
H	0.087233148469	1.960376730620	0.981531033093
C	0.124438232820	0.255695923047	-1.138078142660
O	-0.662134730615	1.858691315522	0.376493530269

Energy = -190.496337898227

Spin(**2) = 0.000000000000

ZPVE = 0.032182756165

Normal mode eigenvalues : nmode = 12

-0.007771078 0.001843792 0.003273272 0.007752708 0.012158058

0.032253670 0.037930647 0.042823657 0.060120688 0.098249397

0.336949180 0.531783624

CONNECTION : 12 - 27

Geometry of TS 108, SYMMETRY = C1

C	-1.377103241043	0.375143364147	0.927645234238
H	-2.457425088329	0.376262961104	0.806858227644
C	-0.503422120436	0.320470303724	-0.176321237204
H	-0.044208223706	1.873236950677	1.105338057475
C	0.146284123338	0.193872599678	-1.303438091456
O	-0.834136762146	1.757068448430	0.554731868319

Energy = -190.487774890787

Spin(**2) = 0.000000000000

ZPVE = 0.033874875483

Normal mode eigenvalues : nmode = 12

-0.024224096 0.002025431 0.004715940 0.015637753 0.026001120

0.028998788 0.040685565 0.047050262 0.056403274 0.109099164

0.370831486 0.528467013

CONNECTION : 14 - 27

Geometry of TS 109, SYMMETRY = C1

C	-1.400233968686	-0.541074829813	0.447106608478
H	-2.227330759960	0.159114085775	0.700171040776
C	-0.501030566686	0.958975623104	-0.185736829904
H	-0.200677126783	2.206581325203	1.198372134265
C	-0.490966864717	-0.239104497020	-0.656123349005
O	-0.422150420373	2.162180761940	0.249397729662

Energy = -190.548372634192

Spin(**2) = 0.000000000000

ZPVE = 0.032947279080

Normal mode eigenvalues : nmode = 12

-0.023693716 0.000871960 0.006638937 0.010818098 0.019960778

0.031759281 0.041992626 0.048077118 0.057943027 0.134570923

0.305491410 0.482225667

CONNECTION : 13 - 8

Geometry of TS 110, SYMMETRY = C1

C	-1.553419372973	0.461368883991	1.330273053821
H	-2.196727611520	0.065793507468	0.529739389679
C	-0.349202260004	0.929477232403	-0.619291945544
H	-0.195619466215	1.922428812459	1.134913906784
C	0.147425838101	-0.016729613328	-1.276878655068
O	-0.871452308481	1.457545400134	0.603140739357

Energy = -190.427348413457

Spin(**2) = 0.000000000000

ZPVE = 0.033477812256

Normal mode eigenvalues : nmode = 12

-0.011487959 0.000616571 0.000964699 0.014428643 0.021485752
0.029017494 0.031649890 0.057691029 0.081367373 0.139430045
0.345974118 0.491140054

CONNECTION : 27 - 28

Geometry of TS 111, SYMMETRY = C1

C	-0.584394731912	-0.338523867614	-0.860369025679
H	-1.322952444738	-0.131376970520	-1.657039855780
C	-0.918642407644	0.551659515964	0.230270120488
H	-1.026747835289	2.215707480532	-0.791416885495
C	-1.382849639848	0.677680765430	1.461801473650
O	-0.320598056767	1.682305447737	-0.393627187171

Energy = -190.493347891900

Spin(**2) = 0.000000000000

ZPVE = 0.032108901216

Normal mode eigenvalues : nmode = 12

-0.004438079 0.001944540 0.003885202 0.008564767 0.012303077
0.032240340 0.034871455 0.044156064 0.057566702 0.100721093
0.330200970 0.523523966

CONNECTION : 19 - 13

Geometry of TS 112, SYMMETRY = C1

C	-0.576147478993	-0.285129717945	-0.884524741142
H	-1.480833586901	-0.401100487186	-1.510894506409
C	-0.874712078891	0.580444643534	0.239559704717
H	-1.010826484054	2.304718341505	-0.638358000808
C	-1.441974504499	0.641679627431	1.428673155444
O	-0.288930047408	1.693771684607	-0.421277217999

Energy = -190.493086002370

Spin(**2) = 0.000000000000

ZPVE = 0.031968539286

Normal mode eigenvalues : nmode = 12

-0.007022384 0.001721780 0.004899884 0.008346331 0.010528112

0.031208316 0.032378131 0.043686649 0.057833459 0.101451835

0.330468144 0.525449881

CONNECTION : 19 - 12

Geometry of TS 113, SYMMETRY = C1

C	-0.537166715090	0.026979712054	-0.885569361579
H	-1.276593553869	-0.119273793217	-1.670642116667
C	-0.893274463521	0.427444687569	0.415111213085
H	-1.024044882940	2.107984942308	-0.857943229864
C	-1.494477036368	0.673279630140	1.550600723371
O	-0.287101607652	1.514557779643	-0.586985561650

Energy = -190.487922059723

Spin(**2) = 0.000000000000

ZPVE = 0.033242676213

Normal mode eigenvalues : nmode = 12

-0.015461545 0.002407348 0.004532443 0.015613467 0.025978580

0.030907606 0.039121845 0.044079301 0.055830370 0.108325759

0.367000159 0.464203504

CONNECTION : 19 - 15

Geometry of TS 114, SYMMETRY = C1

C	-0.639920117719	-0.006092535475	-0.854368494654
H	-1.648815051506	0.282850375536	-1.556328148852
C	-0.904476967747	0.760928407437	0.401534654656
H	-1.199549687201	1.130045856901	-1.500657578816
C	-1.420818782754	0.467690834214	1.555818784828
O	-0.275508024508	1.582938340088	-0.475535355142

Energy = -190.423136673539

Spin(**2) = 0.000000000000

ZPVE = 0.026201593893

Normal mode eigenvalues : nmode = 12

-0.056415491 0.001295857 0.004430097 0.010815434 0.016798387

0.031120235 0.035230333 0.038671693 0.045367100 0.105241870

0.124895981 0.202174243

CONNECTION : 19 - DC

Geometry of TS 115, SYMMETRY = C1

C	-0.452913511398	-0.091080954918	-1.293769846305
H	-1.103183903702	0.147595719380	-2.165927688146
C	-0.806524798342	0.824586534834	0.767573505207
H	-0.971276316566	1.925306204279	-0.894192923033
C	-1.545494862369	0.831849714335	1.778217538645
O	-0.479490740708	1.138836784295	-0.569584353360

Energy = -190.414805769008

Spin(**2) = 0.000000000000

ZPVE = 0.032474394877

Normal mode eigenvalues : nmode = 12

-0.012859984 0.000428998 0.001222861 0.012196016 0.020624555

0.026603029 0.030140074 0.055700805 0.075885752 0.142771708

0.313669844 0.458556708

CONNECTION : 19 - 29

Geometry of TS 116, SYMMETRY = C1

C	-1.634867304879	0.840603120736	1.558152533794
H	-2.181341215329	-0.020656361987	1.139231554657
C	-0.200025827063	0.584641490827	-0.468502294672
H	-0.963626331901	2.068382264196	1.093804150251
C	0.143471277987	0.280844547616	-1.636131079346
O	-0.554927453468	1.097266001999	0.712233621655

Energy = -190.406766609655

Spin(**2) = 0.000000000000

ZPVE = 0.028409283613

Normal mode eigenvalues : nmode = 12

-0.120458529 0.000605076 0.000683800 0.005678419 0.010323254
0.022272579 0.027465399 0.045014858 0.067371413 0.147563054
0.198375235 0.338669609

CONNECTION : 28 - 24

Geometry of TS 117, SYMMETRY = C1

C	-1.720024176879	0.757805193839	1.567187383514
H	-2.129274402606	-0.193654748910	1.166062554780
C	-0.407117840725	0.585040270839	-0.616568106984
H	0.055247893000	1.639576696328	0.234720881748
C	0.525322319170	0.565335452434	-1.465836810132
O	-0.952323654325	1.194022996179	0.560416835419

Energy = -190.400488895294

Spin(**2) = 0.000000000000

ZPVE = 0.027259949505

Normal mode eigenvalues : nmode = 12

-0.121966502 0.000285717 0.001136546 0.002890331 0.006996320
0.020861464 0.021481601 0.043444271 0.074398021 0.133767945
0.191038583 0.318892854

CONNECTION : 9 - 28

Geometry of TS 118, SYMMETRY = C1

C	-1.444191675024	0.310200540348	0.529472757725
H	-1.299603664432	-0.464875227137	1.276153509769
C	-0.543364329511	0.451240476637	-0.483882598636
H	-0.788591397449	2.300929684588	0.639742159271
C	0.072240134374	0.732628207785	-1.631430110637
O	-0.333129158606	1.460754897704	0.827211381765

Energy = -190.478036427390

Spin(**2) = 0.000000000000

ZPVE = 0.033166065899

Normal mode eigenvalues : nmode = 12

-0.065213629 0.002303184 0.005179958 0.013213666 0.022323973

0.025737672 0.036881517 0.039716245 0.052821667 0.115039728

0.374472669 0.511273445

CONNECTION : 27 - 14

Geometry of TS 119, SYMMETRY = Cs

C	-1.769101614006	0.196215890447	1.539377671080
H	-2.338056040982	1.003185100565	2.040816572662
C	-0.191550006692	0.454053361851	-0.367291115352
H	-0.718959973927	1.779931317619	-0.022216124402
C	0.584635706093	0.692117868960	-1.328803585803
O	-1.095522814519	0.885267292045	0.605625672106

Energy = -190.401135422711

Spin(**2) = 0.000000000000

ZPVE = 0.027437793828

Normal mode eigenvalues : nmode = 12

-0.111214426 0.000417596 0.001041694 0.002535955 0.007182410

0.022680009 0.025111360 0.045391349 0.073506512 0.139208594

0.173318601 0.327702020

CONNECTION : 6 - 29

Geometry of TS 120, SYMMETRY = Cs

C	-1.728822907238	0.952801338111	1.392369529763
H	-2.194922790205	0.026073894328	1.020526028833
C	0.327529025576	-0.025084333144	-0.555664643815
H	-0.223732746084	1.981814140141	0.756536422702
C	-0.486149935906	0.691652840534	-1.321938282257
O	-0.752004035783	1.185543792445	0.572721868143

Energy = -190.412399917791

Spin(**2) = 0.000000000000

ZPVE = 0.032580791378

Normal mode eigenvalues : nmode = 12

-0.001795686 0.000594331 0.002852138 0.005067872 0.006779889

0.039312316 0.052625995 0.057632117 0.077143691 0.093090407

0.340727157 0.513320494

CONNECTION : 28 - 28

Geometry of TS 121, SYMMETRY = C1

C	-0.015418453219	0.174841139948	-1.513694935024
H	-0.448192926239	0.525827519841	-2.474568949989
C	-0.999956056512	0.980631407922	0.657681324361
H	0.008532886533	1.608261219811	-1.023638255237
C	-1.343123934626	0.844771403346	1.862684269662
O	-0.908063619838	1.076444148944	-0.644024163697

Energy = -190.389662197358

Spin(**2) = 0.000000000000

ZPVE = 0.028045466759

Normal mode eigenvalues : nmode = 12

-0.058264199 0.000884529 0.001425140 0.007073539 0.011380042

0.018484923 0.030176720 0.038777250 0.070910131 0.146001831

0.171772162 0.321611133

CONNECTION : 24 - 29

Geometry of TS 122, SYMMETRY = Cs

C	-0.276769720179	0.383244467815	-1.601408141584
H	0.018591386973	-0.131313142696	-2.484922960004
C	-0.995937350555	0.619361144357	0.735532303843
H	-0.674271541707	2.039484317430	-0.588359445147
C	-1.363199350754	0.794012335497	1.919794149642
O	-0.623500163214	1.040903335339	-0.574032970894

Energy = -190.344775030732

Spin(**2) = 0.000000000000

ZPVE = 0.031750316259

Normal mode eigenvalues : nmode = 12

-0.078361243 0.000181807 0.000649246 0.008971332 0.013283047
0.018747849 0.021329166 0.066413892 0.077578934 0.141980516
0.355257466 0.449170369

CONNECTION : 29 - 28

Geometry of TS 123, SYMMETRY = Cs

C	-0.851812309139	-1.015634073425	-0.032604804606
H	-0.877021438557	-2.077886316369	-0.059152907835
C	-0.565823444835	0.145867799660	0.064687085449
H	1.196332138910	1.163484443851	0.549228915204
C	-1.353681230535	2.393674552858	-0.101359232431
O	-0.593757541914	1.456591041302	0.081846957420

Energy = -190.427908279727

Spin(**2) = 0.000000000000

ZPVE = 0.026974040142

Normal mode eigenvalues : nmode = 12

-0.013274364 0.002391435 0.003112445 0.005516574 0.010839025
0.013049536 0.018526610 0.027986367 0.035502670 0.092830594
0.189665695 0.454285569

CONNECTION : 21 - DC

Geometry of TS 124, SYMMETRY = Cs

C	-0.837931370140	-1.048061512024	-0.029564599242
H	-1.019519030874	-2.092499731534	-0.097085271944
C	-0.707581119321	0.135155371990	0.027024407382
H	0.692527275554	1.569913006712	0.423668336703
C	-1.397395304117	2.358940734870	-0.113532942005
O	-0.447512631706	1.443449018626	0.120203491338

Energy = -190.423660809731

Spin(**2) = 0.000000000000

ZPVE = 0.027707331968

Normal mode eigenvalues : nmode = 12

-0.031099736 0.002131244 0.003688430 0.006811211 0.012145666

0.016069813 0.016506239 0.037454056 0.046059565 0.080235823

0.196596005 0.457343266

CONNECTION : 21 - 22

Geometry of TS 125, SYMMETRY = Cs

C	-0.673497654625	-0.963092934823	0.015450867219
H	-0.740057975539	-2.025452385702	-0.022017991116
C	-0.979777128564	0.213326965730	-0.043415251943
H	1.246320971402	0.563089608872	0.551071276497
C	-1.262644658056	2.316627199600	-0.078724664712
O	-0.424780583169	1.410664037894	0.125590961980

Energy = -190.416188511458

Spin(**2) = 0.000000000000

ZPVE = 0.026292844162

Normal mode eigenvalues : nmode = 12

-0.017144760 0.001298806 0.001482359 0.006298697 0.006935724

0.016833791 0.019269479 0.026105034 0.047263945 0.081207254

0.165348757 0.447352303

CONNECTION : 21 - 4

Geometry of TS 126, SYMMETRY = Cs

C	-0.946448516719	-1.104898796334	-0.067652708237
H	-1.288602745982	-2.109667330001	-0.124222869714
C	-0.513391546485	0.009029077122	0.002453236322
H	0.575197363698	1.670081237895	0.167952146770
C	-1.401960186663	2.380366421191	-0.097580975706
O	-0.364322434530	1.418053137208	0.036240409676

Energy = -190.424959627717

Spin(**2) = 0.000000000000

ZPVE = 0.031384317076

Normal mode eigenvalues : nmode = 12

-0.014364231 0.001997208 0.002557148 0.008066703 0.009107325
0.015828381 0.016425303 0.025077044 0.067856303 0.176900643
0.399151802 0.454571123

CONNECTION : 22 - DC

Geometry of TS 127, SYMMETRY = C1

C	-0.821020155555	-1.131989354103	-0.042164390227
H	-1.082659800229	-2.155303508374	0.062978882384
C	-0.523544554317	0.019217390011	-0.155551593663
H	0.191849409147	1.741753483798	0.438203953045
C	-1.488699853762	2.665735841642	0.071488078068
O	-0.217606760884	1.285382977071	-0.331581328462

Energy = -190.423719712133

Spin(**2) = 0.000000000000

ZPVE = 0.031464297415

Normal mode eigenvalues : nmode = 12

-0.005856197 0.000591960 0.002791787 0.005232724 0.007386732
0.013630989 0.016420297 0.041656664 0.044799656 0.196498881
0.448504528 0.459230670

CONNECTION : 22 – 6

List of Dissociated Structures

Geometry of DC 0, SYMMETRY = Cs

C	-1.435837478688	-0.218848534700	-0.006721920380
H	-3.672670452745	-1.796482570295	-0.073782663145
C	-0.119831471269	-0.269428651492	0.020118582386
H	0.832079985138	-0.777708133832	0.033345805815
C	-0.791834790213	1.049961307063	0.023465321101
O	-0.687362386186	2.226800898744	0.041187897473

Energy = -190.542974656110

Spin(**2) = 0.000000000000

ZPVE = 0.028747844241

Normal mode eigenvalues : nmode = 11

0.002727477 0.003470295 0.010068389 0.012545501 0.019415239

0.024913771 0.035596724 0.043521613 0.134795905 0.191562503

0.399450442

CONNECTION : 0 - DC

Geometry of DC 1, SYMMETRY = C2v

C	-1.496364115159	-0.687991724478	-0.014118238049
H	-2.444720987601	-1.199666790162	-0.040607047463
C	-0.163893452906	-0.736156794636	0.013067668597
H	0.745284842315	-1.314999428261	0.024472792285
C	-0.786296498562	0.510277660914	0.016366757167
O	-0.684954119127	3.330068399813	0.055821096803

Energy = -190.435430533066

Spin(**2) = 0.000000000000

ZPVE = 0.032881066902

Normal mode eigenvalues : nmode = 11

0.001070401 0.001196917 0.025694256 0.028822953 0.033776147

0.038563673 0.050460265 0.051051839 0.096934638 0.398393812

0.405879575

CONNECTION : 0 - DC

Geometry of DC 2, SYMMETRY = Cs

C	-0.955510301478	1.329208474568	-0.129585301563
H	-0.099276195356	-1.855304146457	1.197614140081
C	-0.282579351602	-0.999561183226	0.585585270238
H	0.641000237160	-2.409248439676	-1.798970410642
C	-0.650974164690	0.174332257493	0.384296866978
O	-1.244418207346	2.376601253374	-0.526236800996

Energy = -190.568827212359

Spin(**2) = 0.000000000000

ZPVE = 0.027081990987

Normal mode eigenvalues : nmode = 11

0.001498164 0.001844300 0.006557149 0.006590951 0.013431813

0.015360312 0.017388213 0.039032671 0.151011388 0.203539281

0.435226584

CONNECTION : 3 - DC

Geometry of DC 3, SYMMETRY = Cs

C	-0.831348701552	0.872685747430	0.054689721011
H	-0.172373542117	-1.907592097979	1.689720037129
C	-0.112303107854	-1.538027009957	0.669992140297
H	0.263224099575	-2.223920596324	-0.084269613752
C	-0.480045350186	-0.307522685224	0.360289667228
O	-1.565145040615	3.476203310910	-0.848850048044

Energy = -190.403056914303

Spin(**2) = 0.000000000000

ZPVE = 0.031227098124

Normal mode eigenvalues : nmode = 11

0.000706616 0.000732169 0.002848332 0.003048105 0.036886909

0.041938721 0.042918594 0.082534630 0.149928551 0.365956417

0.385425227

CONNECTION : 3 - DC

Geometry of DC 4, SYMMETRY = Cs

C	-1.145210979666	-1.154020342848	-0.197820123874
H	-1.340855977845	-2.190636326249	-0.334720432420
C	-0.933098809261	0.021296993876	-0.012311282027
H	-1.492546334158	1.584129826549	2.858409988375
C	-0.717195962773	1.341419493541	0.265774869929
O	-0.351703850291	2.367616021617	-0.134449378315

Energy = -190.577991914003

Spin(**2) = 0.000000000000

ZPVE = 0.027855011237

Normal mode eigenvalues : nmode = 11

0.002132172 0.002963178 0.006579381 0.009053760 0.012566066

0.015884735 0.023223442 0.043885659 0.162959797 0.180843171

0.452562644

CONNECTION : 7 - DC

Geometry of DC 5, SYMMETRY = Cs

C	-1.139871659767	-1.112395635885	-0.184484115960
H	-1.476139616360	-3.748695564243	-1.035698620393
C	-0.918550048085	0.075967151007	-0.018258559242
H	-1.073994027678	1.560463106783	1.536220601766
C	-0.769093507161	1.421388743547	0.485226755820
O	-0.345537621352	2.335039020271	-0.177155981608

Energy = -190.487082760164

Spin(**2) = 0.000000000000

ZPVE = 0.028646989351

Normal mode eigenvalues : nmode = 11

0.001308114 0.001490103 0.003220090 0.003737580 0.012744382

0.037107099 0.040782492 0.072434038 0.113587005 0.318033261

0.331725837

CONNECTION : 7 - DC

Geometry of DC 6, SYMMETRY = Cs

C	-1.116105142175	-1.260349540320	-0.365221662803
H	-1.048556569338	-2.010840941010	-1.116632343399
C	-1.095901825381	-0.231584743130	0.313141214214
H	-1.324591109488	1.098370649110	1.984554481872
C	-1.034482572760	0.922761839996	0.953523347821
O	-0.042358696494	2.970481448181	-0.664672238115

Energy = -190.406049786835

Spin(**2) = 0.000000000000

ZPVE = 0.029596421662

Normal mode eigenvalues : nmode = 11

0.000776642 0.002412148 0.003209644 0.006143884 0.006882678

0.022249646 0.028798716 0.054098991 0.151042063 0.386925171

0.449094626

CONNECTION : 7 - DC

Geometry of DC 7, SYMMETRY = Cs

C	-0.839738033398	-0.420926374210	-0.205556040599
H	-0.062716389698	-2.833055276266	-1.534780551299
C	-1.666725683569	0.132326977684	0.715036263877
H	-0.583337882238	2.727333123738	0.323492213922
C	-0.710273797185	0.888007119581	-0.007287702635
O	-0.173309613159	2.042807459093	-0.233577658448

Energy = -190.472343753197

Spin(**2) = 0.000000000000

ZPVE = 0.029212361472

Normal mode eigenvalues : nmode = 11

0.002366325 0.003243011 0.007075861 0.008060768 0.010973435

0.028060578 0.045772861 0.059129311 0.088114946 0.225885449

0.509047574

CONNECTION : 12 - DC

Geometry of DC 8, SYMMETRY = Cs

C	-0.848934927739	-0.498269146266	-0.215804705311
H	-0.479072273070	-1.345640535901	-0.774718507898
C	-1.629559029803	0.108071662093	0.673815180202
H	-1.369535176836	3.673387202227	1.301757639469
C	-0.683955222515	0.920149315122	-0.024381929204
O	-0.138267026226	1.941585281692	-0.291668761762

Energy = -190.520452969653

Spin(**2) = 0.000000000000

ZPVE = 0.027295114466

Normal mode eigenvalues : nmode = 11

0.001239710 0.001926939 0.010934778 0.016087872 0.024885517

0.025461227 0.032970752 0.037969442 0.088366808 0.161393238

0.393726802

CONNECTION : 12 - DC

Geometry of DC 9, SYMMETRY = Cs

C	-0.987961201898	-0.422150778924	-0.019111600572
H	-2.596182949118	-2.664682937465	-0.093388020570
C	-0.812768056024	0.901087839375	0.025393394256
H	-2.164256582598	2.200805725615	0.070460507744
C	0.289853126262	0.024326282858	-0.005152931076
O	-1.193896807931	2.137508537946	0.067461883740

Energy = -190.475722625070

Spin(**2) = 0.000000000000

ZPVE = 0.029209407594

Normal mode eigenvalues : nmode = 11

0.002398889 0.003691644 0.007372072 0.007538669 0.010809831

0.027754293 0.044717645 0.059641200 0.088346938 0.223167620

0.512513052

CONNECTION : 13 - DC

Geometry of DC 10, SYMMETRY = Cs

C	-1.005196675021	-0.438166182635	-0.019635137861
H	-1.838709865798	-1.126354426799	-0.042140968723
C	-0.754302455132	0.964284427440	0.027481485543
H	-3.821732255219	2.360655944401	0.077283738799
C	0.275264872693	-0.088468578864	-0.008951484802
O	-1.157176221296	2.079733743517	0.065478960496

Energy = -190.513655370559

Spin(**2) = 0.000000000000

ZPVE = 0.027205100619

Normal mode eigenvalues : nmode = 11

0.001521231 0.002967234 0.010802243 0.012597357 0.021671937

0.024112245 0.028661675 0.037962531 0.091016604 0.175941553

0.391422429

CONNECTION : 13 - DC

Geometry of DC 11, SYMMETRY = Cs

C	-1.250103365841	-1.198646398784	0.097214898713
H	-0.548015514077	-0.143347844218	-1.331945275766
C	-0.855866774734	-0.015748051374	-0.279754475638
H	-1.624413814789	1.241427655754	3.022167461021
C	-0.726606811734	1.311508714519	0.273725082835
O	-0.365479946867	2.343896641757	-0.108451784054

Energy = -190.491097036325

Spin(**2) = 0.000000000000

ZPVE = 0.025184074757

Normal mode eigenvalues : nmode = 11

0.000637794 0.002073784 0.005099542 0.005169308 0.011121606

0.013482604 0.023557344 0.049115315 0.114404061 0.175351020

0.339560364

CONNECTION : 11 - DC

Geometry of DC 12, SYMMETRY = C1

C	-1.540319545620	-2.115748515094	0.447771661543
H	-1.163440148524	0.454528109511	-1.522266979862
C	-0.570502130043	0.455233822487	-0.611876765409
H	-1.272686588876	1.327623448646	1.318346700621
C	-0.809561468349	1.467276479591	0.330035791002
O	-0.282989031407	2.518627496222	-0.045180298123

Energy = -190.359183290544

Spin(**2) = 0.000000000000

ZPVE = 0.029859005936

Normal mode eigenvalues : nmode = 11

0.000497133 0.000781249 0.003098428 0.009663005 0.021784598

0.038560206 0.052087815 0.073987178 0.095392800 0.336978221

0.374440367

CONNECTION : 11 - DC

Geometry of DC 13, SYMMETRY = Cs

C	-0.599204945831	0.624779954984	-0.482542659189
H	-0.459972114572	-1.678635894886	-2.271116129724
C	-1.124402292159	0.466504907835	0.686358201745
H	0.005793509844	2.372049120479	-0.842062613186
C	-1.664289782641	0.187939623361	1.814889022961
O	-0.056972902805	1.482287084071	-1.251577923664

Energy = -190.466672461755

Spin(**2) = 0.000000000000

ZPVE = 0.027174219118

Normal mode eigenvalues : nmode = 11

0.001128593 0.001644991 0.004586842 0.004627106 0.012286973

0.013371798 0.041960950 0.079816256 0.095482762 0.165555733

0.463473272

CONNECTION : 14 - DC

Geometry of DC 14, SYMMETRY = Cs

C	-0.440340046285	0.528097495888	-0.927288006842
H	-0.404738603690	-0.357864121202	-1.572330529158
C	-0.995731040654	0.217898598235	0.219091892743
H	0.010514408573	2.382703610340	-0.846458446013
C	-2.140182885018	0.193710242420	2.967915943227
O	0.080562052115	1.653114696715	-1.476149710305

Energy = -190.342423903929

Spin(**2) = 0.000000000000

ZPVE = 0.030580912506

Normal mode eigenvalues : nmode = 11

0.000299867 0.000625231 0.003252171 0.005760656 0.020780074

0.030731875 0.046328272 0.068608396 0.096651783 0.354133033

0.537971772

CONNECTION : 14 - DC

Geometry of DC 15, SYMMETRY = Cs

C	-1.134658156135	0.853413717513	0.426807055031
H	-2.534756977228	-0.591655942496	2.507845091608
C	-0.444466260650	-0.276922651213	-0.132976456746
H	-0.554618167145	-1.276158770357	0.254740798995
C	0.239182024179	0.281263022075	-1.111833567548
O	-1.427658976307	1.960146366804	0.505889163694

Energy = -190.503811843090

Spin(**2) = 0.000000000000

ZPVE = 0.026420833420

Normal mode eigenvalues : nmode = 11

0.000459783 0.002861715 0.005296340 0.008645864 0.014232704

0.019066449 0.032874125 0.047448411 0.092296309 0.180838292

0.396673153

CONNECTION : 5 - DC

Geometry of DC 16, SYMMETRY = Cs

C	-1.048517962847	-1.048075698688	-0.422909262114
H	-1.590263024745	-3.705871270004	-0.650477455497
C	-1.001770284661	0.053197361329	0.224982635007
H	-0.331472452275	3.111127206132	0.337949402687
C	-0.814205812592	1.309086417029	0.544891443429
O	-0.357389467160	2.287715770264	-0.174302365355

Energy = -190.448512616665

Spin(**2) = 0.000000000000

ZPVE = 0.027768901865

Normal mode eigenvalues : nmode = 11

0.000864947 0.001020164 0.004327419 0.004723464 0.009812611

0.016107213 0.047387286 0.060016105 0.099002465 0.203936559

0.519066272

CONNECTION : 10 - DC

Geometry of DC 17, SYMMETRY = Cs

C	-0.751223818736	0.677887465186	-0.398202221444
H	-0.000618123940	-1.497115462647	-1.841160670119
C	-1.317392558364	0.502208007390	0.743675122023
H	0.031228936326	1.223363494058	-1.985470220313
C	-1.859475048408	0.124337113914	1.843084455658
O	-0.370999349068	1.605336271400	-1.189408667901

Energy = -190.483753886629

Spin(**2) = 0.000000000000

ZPVE = 0.028024479013

Normal mode eigenvalues : nmode = 11

0.001099291 0.001777514 0.006061384 0.006317063 0.012873827

0.012953180 0.042688208 0.073074592 0.098850603 0.173581201

0.525815855

CONNECTION : 15 - DC

Geometry of DC 18, SYMMETRY = Cs

C	-0.680110565433	0.499948908609	-0.535766134472
H	-0.400821184725	-0.414185288368	-1.069186415983
C	-1.300499876588	0.471170110682	0.710616116111
H	0.725070984904	1.261514062694	-3.379877291852
C	-1.850282187751	0.217844755086	1.821897112300
O	-0.462423548940	1.621327135732	-1.006286307363

Energy = -190.463068074243

Spin(**2) = 0.000000000000

ZPVE = 0.026787720666

Normal mode eigenvalues : nmode = 11

0.001320183 0.001426839 0.003397055 0.003850603 0.020133535

0.036937394 0.041262619 0.069902298 0.111214287 0.132093784

0.353351311

CONNECTION : 15 - DC

Geometry of DC 19, SYMMETRY = Cs

C	-0.372902114420	0.630491869730	-1.156472616780
H	-0.028852069952	-0.186283902836	-1.823509470044
C	-1.666719449991	0.317631254079	1.450965633501
H	0.281463221998	1.836488471658	-2.506814388276
C	-2.194936754439	-0.055673009171	2.521682186004
O	-0.155064140734	1.793194611047	-1.628534474035

Energy = -190.407069163125

Spin(**2) = 0.000000000000

ZPVE = 0.030673923918

Normal mode eigenvalues : nmode = 11

0.000221922 0.000269594 0.001048840 0.002357086 0.037747573

0.050926408 0.064863631 0.074927108 0.102417957 0.316062826

0.457548300

CONNECTION : 15 - DC

Geometry of DC 20, SYMMETRY = Cs

C	-0.554488442363	0.645840294034	-0.792364187912
H	-0.335872261842	-0.355927940133	-1.202023997127
C	-1.121983360304	0.384842742055	0.355325594766
H	0.190075451994	1.525433537336	-2.313283928589
C	-2.426092407303	-0.058038861595	2.985804702826
O	-0.228987873227	1.763318348924	-1.479267302879

Energy = -190.339511312989

Spin(**2) = 0.000000000000

ZPVE = 0.029831178074

Normal mode eigenvalues : nmode = 11

0.000342902 0.000558229 0.001020716 0.003611836 0.016953432

0.028422199 0.047860149 0.060177078 0.107216516 0.337406734

0.550461281

CONNECTION : 15 - DC

Geometry of DC 21, SYMMETRY = Cs

C	-1.074117612358	-1.157719364384	-0.094000717098
H	-1.122350590666	-2.220511577218	-0.126649515785
C	-1.042067703367	0.052267486480	-0.062864286538
H	1.933974803360	1.242438321510	0.745401875179
C	-1.022089264025	1.406310455947	-0.032245007710
O	-0.323791343328	2.321454642616	0.169308223815

Energy = -190.550214390040

Spin(**2) = 0.000000000000

ZPVE = 0.026778054426

Normal mode eigenvalues : nmode = 11

0.001072031 0.002214946 0.005261977 0.006610130 0.012602562

0.017264496 0.023044729 0.032367783 0.149833513 0.175522922

0.450964235

CONNECTION : 8 - DC

Geometry of DC 22, SYMMETRY = Cs

C	-2.489914405597	0.075060708699	-0.024813951813
H	-3.430938259140	-0.418359004416	-0.050958842193
C	0.853926050859	0.702043976745	0.053229594344
H	2.508693078941	2.842062383416	0.116086468347
C	-1.427916605168	0.613609504684	0.004452853413
O	-0.270336104840	1.288994683565	0.037526632357

Energy = -190.449177542081

Spin(**2) = 0.000000000000

ZPVE = 0.025797570435

Normal mode eigenvalues : nmode = 11

0.001360780 0.002365134 0.006083223 0.007113997 0.014861675

0.017272415 0.017861930 0.031677818 0.074889157 0.194370302

0.456316842

CONNECTION : 6 - DC

Geometry of DC 23, SYMMETRY = Cs

C	-2.361906611475	-0.018928577399	-0.023414627636
H	-4.836668195822	-1.209824287287	-0.090809427308
C	0.897028446395	0.718632924619	0.054348834002
H	1.643337648788	1.539104922687	0.080748116011
C	-1.448002246792	0.798223420125	0.006537449113
O	-0.240541503395	1.340526608664	0.038810751677

Energy = -190.358964057105

Spin(**2) = 0.000000000000

ZPVE = 0.025988682928

Normal mode eigenvalues : nmode = 11

0.001934981 0.002210764 0.002584007 0.003553766 0.008517337

0.016168987 0.042196137 0.052455252 0.072071757 0.282203399

0.318266634

CONNECTION : 6 - DC

Geometry of DC 24, SYMMETRY = C1

C	-1.459061748206	0.473660241813	0.782297637832
H	-2.415979843743	0.054209198490	1.054527802595
C	-0.509888350329	0.460564457956	-0.236601208530
H	-0.162041116807	0.036472181437	3.318903247689
C	0.270394395375	0.127852365371	-1.227615985044
O	-1.190827443550	1.683430623457	0.302298736481

Energy = -190.402081242984

Spin(**2) = 0.000000000000

ZPVE = 0.024966026683

Normal mode eigenvalues : nmode = 11

0.001876771 0.002525908 0.003882225 0.006543802 0.008749551

0.028414533 0.030332791 0.049786576 0.065503370 0.124042546

0.393822704

CONNECTION : 20 - DC

Geometry of DC 25, SYMMETRY = Cs

C	-1.597435640844	0.508791873270	1.192319424322
H	-2.603817054601	0.133465315811	1.071747676524
C	-0.767098408767	0.799351125073	-0.012685451902
H	-1.127552572991	0.441789698365	2.163366632612
C	0.988964198237	-0.447537291597	-2.037484358418
O	-1.360472398126	1.804049639444	0.506013113625

Energy = -190.374040366290

Spin(**2) = 0.000000000000

ZPVE = 0.032213492224

Normal mode eigenvalues : nmode = 11

0.000455131 0.000505025 0.022597309 0.028350494 0.030401202

0.046826406 0.046997911 0.060454924 0.085249260 0.372651829

0.399901195

CONNECTION : 20 - DC

Geometry of DC 26, SYMMETRY = Cs

C	-1.067553476158	-0.932462976463	-0.557513970707
H	-1.292339661567	-1.953301777817	-0.749121317176
C	-0.827177022326	0.207122702125	-0.307902696607
H	-1.660389129381	0.322331894500	2.618891768806
C	-0.641980693910	2.204393649870	0.928413908896
O	-0.538342827125	1.479095684550	-0.098763616607

Energy = -190.452069295818

Spin(**2) = 0.000000000000

ZPVE = 0.026152982481

Normal mode eigenvalues : nmode = 11

0.002196523 0.002991379 0.006422251 0.007429831 0.014309690

0.017727249 0.017964530 0.031999078 0.078822231 0.196210438

0.456884890

CONNECTION : 9 - DC

Geometry of DC 27, SYMMETRY = Cs

C	-1.051726438740	-0.923862147865	-0.602966298881
H	-1.651892977816	-3.551898531364	-1.023828182335
C	-0.839475183577	0.218311387492	-0.255953969243
H	-1.099738445009	1.466060925360	1.788614766134
C	-0.683325676294	2.149491636271	1.017109819732
O	-0.522642692483	1.534099233725	-0.100556518849

Energy = -190.359113896193

Spin(**2) = 0.000000000000

ZPVE = 0.026019394861

Normal mode eigenvalues : nmode = 11

0.000952951 0.001047541 0.002694172 0.002914933 0.008427430

0.018770820 0.034300874 0.054997420 0.070399528 0.310039625

0.338738231

CONNECTION : 9 - DC

Geometry of DC 28, SYMMETRY = C1

C	-1.528634270022	0.429361295538	-0.517549744440
H	-0.638513256365	-1.485678911929	0.298897259754
C	-0.691312933475	-0.445671329420	0.052709835957
H	1.848699061002	-0.269693131771	-1.090287540427
C	-0.330005524635	0.830301860931	0.693420879323
O	-0.801706937626	1.712758019720	-0.118553628359

Energy = -190.428110775712

Spin(**2) = 0.000000000000

ZPVE = 0.025157739508

Normal mode eigenvalues : nmode = 11

0.000247764 0.003166404 0.005796046 0.016111115 0.017465790

0.026686427 0.030980602 0.039815017 0.065662665 0.086000729

0.425871504

CONNECTION : 23 - DC

Geometry of DC 29, SYMMETRY = Cs

C	-2.639990075647	1.384099438315	-0.417903445779
H	0.425727642707	-1.247762586978	0.435208504939
C	0.622500742917	-0.173471604453	0.348852025450
H	3.365311344570	0.258526396213	0.795876824233
C	-1.466821126438	1.002101057338	-0.162409554089
O	-0.263305741493	0.615102135424	0.099162802902

Energy = -190.357169122303

Spin(**2) = 0.000000000000

ZPVE = 0.024578494186

Normal mode eigenvalues : nmode = 11

0.000218869 0.000758840 0.002077718 0.003218587 0.005472529

0.007844310 0.031246597 0.050795846 0.145768915 0.175470179

0.346611912

CONNECTION : 24 - DC

Geometry of DC 30, SYMMETRY = Cs

C	-0.446295279502	-2.328860388354	-0.545710947326
H	-2.213522499584	1.038088680901	-0.482947947569
C	-1.258288568150	0.543232356705	-0.215412994109
H	-0.122820719106	3.293796747905	0.862686190590
C	-0.972844049017	2.748110553151	0.400149991850
O	-0.471609838088	1.491099374138	0.313474634540

Energy = -190.253869670615

Spin(**2) = 0.000000000000

ZPVE = 0.027491774220

Normal mode eigenvalues : nmode = 11

0.000327006 0.000788204 0.001539380 0.009945352 0.010287836

0.038011575 0.043522643 0.064413804 0.076760931 0.316100835

0.318457133

CONNECTION : 18 - DC

Geometry of DC 31, SYMMETRY = C1

C	-1.319897130448	0.028018101929	0.990496396238
H	-3.893813618222	0.352280045927	0.188548565263
C	-0.586329244457	0.537192370015	-0.096456135671
H	0.071529959991	2.015120828813	0.999523531824
C	0.088206675419	0.185321368833	-1.194647842034
O	-0.681688110161	1.857785776917	0.408179960450

Energy = -190.336422247741

Spin(**2) = 0.000000000000

ZPVE = 0.023632035440

Normal mode eigenvalues : nmode = 11

-0.000015893 0.001549932 0.003090690 0.004463151 0.005155967

0.011113857 0.033377849 0.048295690 0.059692599 0.094786554

0.524615150

CONNECTION : 27 - DC

Geometry of DC 32, SYMMETRY = Cs

C	-1.731401226462	0.732326838764	1.412350322161
H	-2.155400905658	-0.259246902996	1.161503899827
C	-0.252428837889	0.717900933170	-0.505931999801
H	0.175244833984	3.566673721796	1.231809470242
C	0.254716811148	0.234036135011	-1.548890952739
O	-0.864487625851	1.098025943967	0.588744643269

Energy = -190.327348715194

Spin(**2) = 0.000000000000

ZPVE = 0.024016225903

Normal mode eigenvalues : nmode = 11

0.001074272 0.001315961 0.003333269 0.004624687 0.006896881

0.014360358 0.034580865 0.044211531 0.108924955 0.130391458

0.320046518

CONNECTION : 28 - DC

Geometry of DC 33, SYMMETRY = Cs

C	-0.359942421575	0.002471296899	-1.416116383011
H	-0.068690884500	0.520261800595	-2.337196747147
C	-1.006803909647	0.866622890790	0.715642192879
H	-0.360614730551	3.354131200487	-1.271020094032
C	-1.379096568806	0.923961241851	1.923981666919
O	-0.627389406247	0.854507573268	-0.513611136306

Energy = -190.340325167297

Spin(**2) = 0.000000000000

ZPVE = 0.024913734310

Normal mode eigenvalues : nmode = 11

0.000859180 0.001279414 0.002271220 0.006497626 0.010622743

0.018630797 0.038070225 0.059761862 0.081131013 0.142846903

0.350615397

CONNECTION : 29 - DC

Geometry of DC 34, SYMMETRY = Cs

C	-0.839767234533	-1.065442588871	-0.030378521027
H	-0.946634335483	-2.122071504258	-0.078401885404
C	-0.728697838539	0.118643506709	0.021140829128
H	2.452727253583	1.940887666976	0.895430303364
C	-1.357303618341	2.353671372603	-0.103055708852
O	-0.526943835343	1.441426582333	0.099206741299

Energy = -190.407801123737

Spin(**2) = 0.000000000000

ZPVE = 0.026531866006

Normal mode eigenvalues : nmode = 11

0.001300693 0.001368041 0.003185136 0.004435064 0.008781231

0.017291897 0.017766194 0.030792020 0.168836279 0.201772992

0.456199599

CONNECTION : 21 - DC

Reaction Kinetics and Rate Constants

Pathway: EQ1-TS0-EQ0
Reactant Energy: 21.46 kcal/mol
TS Energy: 30.07 kcal/mol
Product Energy: 0.0 kcal/mol
Barrier Height: 8.61 kcal/mol
Imaginary Frequency: 815.12 cm^-1

Temperature(K)	Wigner_Factor	Eckart_Factor	TST Rate(s^{-1})	Wigner Rate (s^{-1})	Eckart Rate (s^{-1})
150.00	3.5470	66.2572	8.921e-01	3.164e+00	5.911e+01
153.03	3.4472	51.5787	1.613e+00	5.559e+00	8.317e+01
156.06	3.3531	40.9946	2.850e+00	9.555e+00	1.168e+02
159.09	3.2643	33.2046	4.929e+00	1.609e+01	1.637e+02
162.12	3.1804	27.3617	8.357e+00	2.658e+01	2.287e+02
165.15	3.1011	22.9019	1.390e+01	4.311e+01	3.184e+02
168.18	3.0261	19.4425	2.271e+01	6.873e+01	4.416e+02
171.21	2.9550	16.7190	3.648e+01	1.078e+02	6.099e+02
174.24	2.8876	14.5451	5.765e+01	1.665e+02	8.385e+02
177.27	2.8236	12.7879	8.972e+01	2.533e+02	1.147e+03
180.30	2.7628	11.3509	1.376e+02	3.802e+02	1.562e+03
183.33	2.7050	10.1629	2.082e+02	5.631e+02	2.115e+03
186.36	2.6500	9.1711	3.107e+02	8.235e+02	2.850e+03
189.39	2.5977	8.3353	4.581e+02	1.190e+03	3.818e+03
192.42	2.5477	7.6250	6.673e+02	1.700e+03	5.088e+03
195.45	2.5001	7.0167	9.609e+02	2.402e+03	6.743e+03
198.48	2.4547	6.4918	1.369e+03	3.360e+03	8.886e+03
201.52	2.4112	6.0358	1.930e+03	4.653e+03	1.165e+04
204.55	2.3697	5.6373	2.693e+03	6.383e+03	1.518e+04
207.58	2.3300	5.2868	3.724e+03	8.677e+03	1.969e+04
210.61	2.2920	4.9771	5.102e+03	1.169e+04	2.539e+04
213.64	2.2556	4.7018	6.929e+03	1.563e+04	3.258e+04
216.67	2.2208	4.4561	9.332e+03	2.072e+04	4.158e+04
219.70	2.1873	4.2358	1.247e+04	2.727e+04	5.281e+04
222.73	2.1552	4.0375	1.653e+04	3.562e+04	6.674e+04
225.76	2.1244	3.8582	2.175e+04	4.621e+04	8.393e+04
228.79	2.0948	3.6956	2.843e+04	5.955e+04	1.051e+05
231.82	2.0664	3.5476	3.689e+04	7.624e+04	1.309e+05
234.85	2.0391	3.4124	4.757e+04	9.699e+04	1.623e+05
237.88	2.0128	3.2887	6.095e+04	1.227e+05	2.004e+05
240.91	1.9874	3.1750	7.762e+04	1.543e+05	2.464e+05
243.94	1.9631	3.0703	9.827e+04	1.929e+05	3.017e+05
246.97	1.9396	2.9737	1.237e+05	2.399e+05	3.679e+05
250.00	1.9169	2.8842	1.549e+05	2.969e+05	4.468e+05
253.03	1.8951	2.8013	1.929e+05	3.657e+05	5.405e+05
256.06	1.8740	2.7242	2.391e+05	4.481e+05	6.514e+05
259.09	1.8537	2.6524	2.949e+05	5.467e+05	7.822e+05

262.12	1.8341	2.5854	3.620e+05	6.639e+05	9.358e+05
265.15	1.8151	2.5227	4.423e+05	8.028e+05	1.116e+06
268.18	1.7968	2.4640	5.381e+05	9.668e+05	1.326e+06
271.21	1.7791	2.4090	6.518e+05	1.160e+06	1.570e+06
274.24	1.7620	2.3573	7.863e+05	1.385e+06	1.854e+06
277.27	1.7454	2.3086	9.448e+05	1.649e+06	2.181e+06
280.30	1.7294	2.2628	1.131e+06	1.956e+06	2.559e+06
283.33	1.7139	2.2195	1.349e+06	2.311e+06	2.993e+06
286.36	1.6988	2.1786	1.603e+06	2.722e+06	3.491e+06
289.39	1.6843	2.1400	1.898e+06	3.196e+06	4.061e+06
292.42	1.6702	2.1034	2.239e+06	3.740e+06	4.710e+06
295.45	1.6565	2.0686	2.634e+06	4.363e+06	5.448e+06
298.48	1.6432	2.0356	3.088e+06	5.074e+06	6.286e+06
301.52	1.6304	2.0043	3.609e+06	5.884e+06	7.234e+06
304.55	1.6179	1.9745	4.206e+06	6.804e+06	8.304e+06
307.58	1.6058	1.9461	4.887e+06	7.847e+06	9.510e+06
310.61	1.5940	1.9191	5.662e+06	9.025e+06	1.087e+07
313.64	1.5826	1.8933	6.542e+06	1.035e+07	1.239e+07
316.67	1.5715	1.8686	7.539e+06	1.185e+07	1.409e+07
319.70	1.5607	1.8451	8.665e+06	1.352e+07	1.599e+07
322.73	1.5502	1.8225	9.934e+06	1.540e+07	1.810e+07
325.76	1.5400	1.8010	1.136e+07	1.750e+07	2.046e+07
328.79	1.5301	1.7803	1.296e+07	1.983e+07	2.308e+07
331.82	1.5205	1.7605	1.475e+07	2.243e+07	2.598e+07
334.85	1.5111	1.7416	1.676e+07	2.532e+07	2.918e+07
337.88	1.5020	1.7233	1.899e+07	2.852e+07	3.272e+07
340.91	1.4931	1.7058	2.147e+07	3.206e+07	3.663e+07
343.94	1.4845	1.6890	2.423e+07	3.597e+07	4.092e+07
346.97	1.4760	1.6728	2.729e+07	4.028e+07	4.564e+07
350.00	1.4678	1.6572	3.067e+07	4.501e+07	5.082e+07
353.03	1.4598	1.6422	3.440e+07	5.022e+07	5.649e+07
356.06	1.4520	1.6278	3.852e+07	5.593e+07	6.270e+07
359.09	1.4444	1.6138	4.304e+07	6.218e+07	6.947e+07
362.12	1.4370	1.6004	4.802e+07	6.901e+07	7.685e+07
365.15	1.4298	1.5874	5.348e+07	7.646e+07	8.489e+07
368.18	1.4228	1.5749	5.945e+07	8.459e+07	9.363e+07
371.21	1.4159	1.5628	6.598e+07	9.343e+07	1.031e+08
374.24	1.4092	1.5512	7.312e+07	1.030e+08	1.134e+08
377.27	1.4026	1.5399	8.089e+07	1.135e+08	1.246e+08
380.30	1.3962	1.5289	8.936e+07	1.248e+08	1.366e+08
383.33	1.3900	1.5184	9.856e+07	1.370e+08	1.496e+08
386.36	1.3839	1.5081	1.085e+08	1.502e+08	1.637e+08
389.39	1.3780	1.4982	1.194e+08	1.645e+08	1.788e+08
392.42	1.3721	1.4886	1.311e+08	1.799e+08	1.951e+08
395.45	1.3665	1.4793	1.438e+08	1.965e+08	2.127e+08
398.48	1.3609	1.4703	1.575e+08	2.143e+08	2.315e+08
401.52	1.3555	1.4615	1.722e+08	2.334e+08	2.517e+08
404.55	1.3502	1.4530	1.881e+08	2.540e+08	2.734e+08

407.58	1.3450	1.4448	2.052e+08	2.761e+08	2.965e+08
410.61	1.3399	1.4367	2.237e+08	2.997e+08	3.213e+08
413.64	1.3350	1.4290	2.434e+08	3.249e+08	3.478e+08
416.67	1.3301	1.4214	2.646e+08	3.519e+08	3.761e+08
419.70	1.3253	1.4141	2.873e+08	3.808e+08	4.063e+08
422.73	1.3207	1.4069	3.116e+08	4.115e+08	4.384e+08
425.76	1.3162	1.4000	3.376e+08	4.443e+08	4.726e+08
428.79	1.3117	1.3932	3.653e+08	4.792e+08	5.090e+08
431.82	1.3073	1.3866	3.950e+08	5.164e+08	5.477e+08
434.85	1.3031	1.3802	4.265e+08	5.558e+08	5.887e+08
437.88	1.2989	1.3740	4.602e+08	5.977e+08	6.323e+08
440.91	1.2948	1.3679	4.960e+08	6.422e+08	6.784e+08
443.94	1.2908	1.3620	5.340e+08	6.893e+08	7.273e+08
446.97	1.2869	1.3562	5.745e+08	7.392e+08	7.791e+08
450.00	1.2830	1.3506	6.174e+08	7.921e+08	8.338e+08

Pathway: EQ11-TS14-EQ7
Reactant Energy: 36.44 kcal/mol
TS Energy: 37.85 kcal/mol
Product Energy: -4.91 kcal/mol
Barrier Height: 1.41 kcal/mol
Imaginary Frequency: 615.25 cm^-1

Temperature(K)	Wigner_Factor	Eckart_Factor	TST Rate(s^{-1})	Wigner Rate (s^{-1})	Eckart Rate (s^{-1})
150.00	2.4511	3.0158	2.758e+10	6.760e+10	8.318e+10
153.03	2.3942	2.8936	3.090e+10	7.398e+10	8.941e+10
156.06	2.3406	2.7827	3.448e+10	8.070e+10	9.595e+10
159.09	2.2900	2.6816	3.833e+10	8.777e+10	1.028e+11
162.12	2.2422	2.5893	4.246e+10	9.519e+10	1.099e+11
165.15	2.1971	2.5048	4.687e+10	1.030e+11	1.174e+11
168.18	2.1543	2.4271	5.157e+10	1.111e+11	1.252e+11
171.21	2.1138	2.3556	5.657e+10	1.196e+11	1.332e+11
174.24	2.0754	2.2896	6.187e+10	1.284e+11	1.417e+11
177.27	2.0390	2.2285	6.748e+10	1.376e+11	1.504e+11
180.30	2.0043	2.1719	7.341e+10	1.471e+11	1.594e+11
183.33	1.9714	2.1193	7.966e+10	1.570e+11	1.688e+11
186.36	1.9401	2.0703	8.624e+10	1.673e+11	1.785e+11
189.39	1.9102	2.0246	9.315e+10	1.779e+11	1.886e+11
192.42	1.8818	1.9819	1.004e+11	1.889e+11	1.990e+11
195.45	1.8546	1.9419	1.080e+11	2.002e+11	2.097e+11
198.48	1.8288	1.9045	1.159e+11	2.119e+11	2.207e+11
201.52	1.8040	1.8693	1.242e+11	2.240e+11	2.321e+11
204.55	1.7804	1.8363	1.328e+11	2.364e+11	2.438e+11
207.58	1.7577	1.8052	1.417e+11	2.491e+11	2.559e+11
210.61	1.7361	1.7758	1.511e+11	2.623e+11	2.683e+11
213.64	1.7154	1.7481	1.607e+11	2.757e+11	2.810e+11
216.67	1.6955	1.7219	1.708e+11	2.895e+11	2.940e+11
219.70	1.6764	1.6972	1.812e+11	3.037e+11	3.074e+11

222.73	1.6582	1.6737	1.919e+11	3.182e+11	3.212e+11
225.76	1.6406	1.6515	2.030e+11	3.331e+11	3.353e+11
228.79	1.6238	1.6304	2.145e+11	3.483e+11	3.497e+11
231.82	1.6076	1.6103	2.263e+11	3.638e+11	3.644e+11
234.85	1.5920	1.5912	2.385e+11	3.797e+11	3.795e+11
237.88	1.5770	1.5730	2.511e+11	3.959e+11	3.949e+11
240.91	1.5626	1.5557	2.640e+11	4.125e+11	4.107e+11
243.94	1.5487	1.5392	2.773e+11	4.294e+11	4.268e+11
246.97	1.5353	1.5234	2.909e+11	4.466e+11	4.432e+11
250.00	1.5224	1.5084	3.049e+11	4.642e+11	4.599e+11
253.03	1.5100	1.4940	3.193e+11	4.821e+11	4.770e+11
256.06	1.4980	1.4802	3.340e+11	5.003e+11	4.944e+11
259.09	1.4864	1.4670	3.491e+11	5.189e+11	5.121e+11
262.12	1.4752	1.4544	3.645e+11	5.378e+11	5.302e+11
265.15	1.4644	1.4423	3.803e+11	5.570e+11	5.485e+11
268.18	1.4540	1.4307	3.965e+11	5.765e+11	5.672e+11
271.21	1.4439	1.4195	4.130e+11	5.963e+11	5.862e+11
274.24	1.4341	1.4088	4.299e+11	6.165e+11	6.056e+11
277.27	1.4247	1.3985	4.471e+11	6.369e+11	6.252e+11
280.30	1.4156	1.3886	4.646e+11	6.577e+11	6.452e+11
283.33	1.4067	1.3791	4.825e+11	6.788e+11	6.655e+11
286.36	1.3981	1.3699	5.008e+11	7.002e+11	6.860e+11
289.39	1.3899	1.3610	5.194e+11	7.219e+11	7.069e+11
292.42	1.3818	1.3525	5.384e+11	7.439e+11	7.281e+11
295.45	1.3740	1.3443	5.576e+11	7.662e+11	7.496e+11
298.48	1.3665	1.3364	5.773e+11	7.888e+11	7.714e+11
301.52	1.3591	1.3287	5.972e+11	8.117e+11	7.936e+11
304.55	1.3520	1.3214	6.175e+11	8.349e+11	8.160e+11
307.58	1.3451	1.3142	6.381e+11	8.584e+11	8.387e+11
310.61	1.3384	1.3073	6.591e+11	8.821e+11	8.617e+11
313.64	1.3319	1.3007	6.804e+11	9.062e+11	8.849e+11
316.67	1.3256	1.2942	7.020e+11	9.305e+11	9.085e+11
319.70	1.3194	1.2880	7.239e+11	9.552e+11	9.324e+11
322.73	1.3135	1.2819	7.462e+11	9.801e+11	9.565e+11
325.76	1.3077	1.2761	7.687e+11	1.005e+12	9.810e+11
328.79	1.3020	1.2704	7.916e+11	1.031e+12	1.006e+12
331.82	1.2965	1.2649	8.148e+11	1.056e+12	1.031e+12
334.85	1.2912	1.2596	8.383e+11	1.082e+12	1.056e+12
337.88	1.2860	1.2545	8.621e+11	1.109e+12	1.082e+12
340.91	1.2809	1.2494	8.863e+11	1.135e+12	1.107e+12
343.94	1.2760	1.2446	9.107e+11	1.162e+12	1.133e+12
346.97	1.2712	1.2399	9.354e+11	1.189e+12	1.160e+12
350.00	1.2665	1.2353	9.604e+11	1.216e+12	1.186e+12
353.03	1.2620	1.2308	9.858e+11	1.244e+12	1.213e+12
356.06	1.2575	1.2265	1.011e+12	1.272e+12	1.240e+12
359.09	1.2532	1.2223	1.037e+12	1.300e+12	1.268e+12
362.12	1.2490	1.2182	1.063e+12	1.328e+12	1.296e+12
365.15	1.2449	1.2142	1.090e+12	1.357e+12	1.323e+12

368.18	1.2409	1.2104	1.117e+12	1.386e+12	1.352e+12
371.21	1.2369	1.2066	1.144e+12	1.415e+12	1.380e+12
374.24	1.2331	1.2030	1.171e+12	1.444e+12	1.409e+12
377.27	1.2294	1.1994	1.199e+12	1.474e+12	1.438e+12
380.30	1.2257	1.1959	1.227e+12	1.503e+12	1.467e+12
383.33	1.2222	1.1925	1.255e+12	1.533e+12	1.496e+12
386.36	1.2187	1.1892	1.283e+12	1.564e+12	1.526e+12
389.39	1.2153	1.1860	1.312e+12	1.594e+12	1.556e+12
392.42	1.2120	1.1829	1.341e+12	1.625e+12	1.586e+12
395.45	1.2088	1.1798	1.370e+12	1.656e+12	1.616e+12
398.48	1.2056	1.1769	1.399e+12	1.687e+12	1.647e+12
401.52	1.2025	1.1740	1.429e+12	1.719e+12	1.678e+12
404.55	1.1995	1.1711	1.459e+12	1.750e+12	1.709e+12
407.58	1.1965	1.1684	1.489e+12	1.782e+12	1.740e+12
410.61	1.1937	1.1657	1.520e+12	1.814e+12	1.772e+12
413.64	1.1908	1.1630	1.550e+12	1.846e+12	1.803e+12
416.67	1.1881	1.1605	1.581e+12	1.879e+12	1.835e+12
419.70	1.1854	1.1580	1.613e+12	1.912e+12	1.867e+12
422.73	1.1827	1.1555	1.644e+12	1.945e+12	1.900e+12
425.76	1.1801	1.1531	1.676e+12	1.978e+12	1.932e+12
428.79	1.1776	1.1508	1.708e+12	2.011e+12	1.965e+12
431.82	1.1751	1.1485	1.740e+12	2.045e+12	1.998e+12
434.85	1.1727	1.1462	1.772e+12	2.078e+12	2.031e+12
437.88	1.1703	1.1440	1.805e+12	2.112e+12	2.065e+12
440.91	1.1680	1.1419	1.838e+12	2.146e+12	2.098e+12
443.94	1.1657	1.1398	1.871e+12	2.181e+12	2.132e+12
446.97	1.1634	1.1378	1.904e+12	2.215e+12	2.166e+12
450.00	1.1612	1.1358	1.938e+12	2.250e+12	2.201e+12

Pathway: EQ7-TS17-EQ9
Reactant Energy: -4.91 kcal/mol
TS Energy: 99.23 kcal/mol
Product Energy: 77.33 kcal/mol
Barrier Height: 104.14 kcal/mol
Imaginary Frequency: 404.69 cm^-1

Temperature(K)	Wigner_Factor	Eckart_Factor	TST Rate(s^{-1})	Wigner Rate (s^{-1})	Eckart Rate (s^{-1})
150.00	1.6278	2.0608	5.836e-140	9.499e-140	1.203e-139
153.03	1.6032	1.9940	6.016e-137	9.645e-137	1.200e-136
156.06	1.5800	1.9338	4.739e-134	7.487e-134	9.164e-134
159.09	1.5581	1.8795	2.896e-131	4.513e-131	5.444e-131
162.12	1.5375	1.8302	1.393e-128	2.142e-128	2.550e-128
165.15	1.5179	1.7853	5.345e-126	8.113e-126	9.543e-126
168.18	1.4994	1.7443	1.655e-123	2.482e-123	2.887e-123
171.21	1.4819	1.7066	4.186e-121	6.203e-121	7.144e-121
174.24	1.4653	1.6720	8.735e-119	1.280e-118	1.460e-118
177.27	1.4495	1.6401	1.519e-116	2.202e-116	2.491e-116
180.30	1.4345	1.6105	2.221e-114	3.187e-114	3.578e-114

183.33	1.4203	1.5832	2.756e-112	3.914e-112	4.363e-112
186.36	1.4067	1.5577	2.924e-110	4.113e-110	4.555e-110
189.39	1.3938	1.5340	2.673e-108	3.725e-108	4.100e-108
192.42	1.3815	1.5119	2.120e-106	2.928e-106	3.205e-106
195.45	1.3698	1.4912	1.468e-104	2.011e-104	2.190e-104
198.48	1.3586	1.4719	8.938e-103	1.214e-102	1.316e-102
201.52	1.3479	1.4537	4.810e-101	6.483e-101	6.992e-101
204.55	1.3376	1.4367	2.300e-99	3.077e-99	3.305e-99
207.58	1.3278	1.4206	9.830e-98	1.305e-97	1.396e-97
210.61	1.3185	1.4055	3.771e-96	4.972e-96	5.300e-96
213.64	1.3095	1.3912	1.305e-94	1.708e-94	1.815e-94
216.67	1.3009	1.3777	4.089e-93	5.319e-93	5.633e-93
219.70	1.2927	1.3649	1.165e-91	1.507e-91	1.591e-91
222.73	1.2848	1.3528	3.033e-90	3.897e-90	4.103e-90
225.76	1.2772	1.3414	7.234e-89	9.238e-89	9.703e-89
228.79	1.2699	1.3305	1.586e-87	2.015e-87	2.111e-87
231.82	1.2629	1.3201	3.210e-86	4.054e-86	4.238e-86
234.85	1.2561	1.3103	6.011e-85	7.550e-85	7.876e-85
237.88	1.2496	1.3009	1.045e-83	1.306e-83	1.359e-83
240.91	1.2434	1.2920	1.690e-82	2.102e-82	2.184e-82
243.94	1.2374	1.2835	2.553e-81	3.159e-81	3.276e-81
246.97	1.2316	1.2753	3.607e-80	4.442e-80	4.600e-80
250.00	1.2260	1.2676	4.780e-79	5.861e-79	6.059e-79
253.03	1.2206	1.2601	5.956e-78	7.270e-78	7.505e-78
256.06	1.2154	1.2530	6.992e-77	8.498e-77	8.761e-77
259.09	1.2104	1.2462	7.749e-76	9.380e-76	9.657e-76
262.12	1.2056	1.2397	8.125e-75	9.796e-75	1.007e-74
265.15	1.2009	1.2334	8.075e-74	9.698e-74	9.960e-74
268.18	1.1964	1.2274	7.620e-73	9.117e-73	9.353e-73
271.21	1.1920	1.2216	6.840e-72	8.154e-72	8.356e-72
274.24	1.1878	1.2161	5.850e-71	6.949e-71	7.114e-71
277.27	1.1837	1.2107	4.774e-70	5.652e-70	5.781e-70
280.30	1.1798	1.2056	3.724e-69	4.394e-69	4.490e-69
283.33	1.1760	1.2007	2.780e-68	3.270e-68	3.338e-68
286.36	1.1723	1.1959	1.989e-67	2.332e-67	2.379e-67
289.39	1.1687	1.1913	1.366e-66	1.597e-66	1.628e-66
292.42	1.1652	1.1869	9.016e-66	1.051e-65	1.070e-65
295.45	1.1618	1.1827	5.725e-65	6.651e-65	6.771e-65
298.48	1.1586	1.1786	3.501e-64	4.056e-64	4.127e-64
301.52	1.1554	1.1746	2.065e-63	2.386e-63	2.426e-63
304.55	1.1523	1.1708	1.176e-62	1.355e-62	1.377e-62
307.58	1.1493	1.1671	6.471e-62	7.437e-62	7.552e-62
310.61	1.1464	1.1635	3.444e-61	3.949e-61	4.007e-61
313.64	1.1436	1.1600	1.775e-60	2.030e-60	2.059e-60
316.67	1.1409	1.1567	8.869e-60	1.012e-59	1.026e-59
319.70	1.1382	1.1534	4.298e-59	4.892e-59	4.957e-59
322.73	1.1356	1.1503	2.022e-58	2.296e-58	2.326e-58
325.76	1.1331	1.1472	9.245e-58	1.048e-57	1.061e-57

328.79	1.1307	1.1443	4.110e-57	4.647e-57	4.703e-57
331.82	1.1283	1.1414	1.778e-56	2.006e-56	2.030e-56
334.85	1.1260	1.1387	7.493e-56	8.437e-56	8.532e-56
337.88	1.1237	1.1360	3.077e-55	3.458e-55	3.496e-55
340.91	1.1215	1.1334	1.232e-54	1.382e-54	1.397e-54
343.94	1.1194	1.1308	4.818e-54	5.393e-54	5.448e-54
346.97	1.1173	1.1284	1.839e-53	2.055e-53	2.075e-53
350.00	1.1153	1.1260	6.859e-53	7.650e-53	7.723e-53
353.03	1.1133	1.1237	2.501e-52	2.785e-52	2.811e-52
356.06	1.1114	1.1214	8.924e-52	9.918e-52	1.001e-51
359.09	1.1095	1.1192	3.116e-51	3.458e-51	3.488e-51
362.12	1.1077	1.1171	1.066e-50	1.181e-50	1.191e-50
365.15	1.1059	1.1150	3.572e-50	3.950e-50	3.982e-50
368.18	1.1042	1.1130	1.173e-49	1.296e-49	1.306e-49
371.21	1.1025	1.1111	3.781e-49	4.169e-49	4.201e-49
374.24	1.1009	1.1091	1.196e-48	1.316e-48	1.326e-48
377.27	1.0992	1.1073	3.712e-48	4.080e-48	4.110e-48
380.30	1.0977	1.1055	1.132e-47	1.242e-47	1.251e-47
383.33	1.0961	1.1037	3.390e-47	3.716e-47	3.742e-47
386.36	1.0946	1.1020	9.985e-47	1.093e-46	1.100e-46
389.39	1.0932	1.1003	2.892e-46	3.161e-46	3.182e-46
392.42	1.0917	1.0987	8.238e-46	8.994e-46	9.051e-46
395.45	1.0903	1.0971	2.310e-45	2.519e-45	2.534e-45
398.48	1.0890	1.0955	6.377e-45	6.944e-45	6.986e-45
401.52	1.0876	1.0940	1.734e-44	1.885e-44	1.896e-44
404.55	1.0863	1.0925	4.643e-44	5.044e-44	5.072e-44
407.58	1.0850	1.0911	1.225e-43	1.330e-43	1.337e-43
410.61	1.0838	1.0897	3.189e-43	3.456e-43	3.475e-43
413.64	1.0826	1.0883	8.183e-43	8.858e-43	8.905e-43
416.67	1.0814	1.0869	2.071e-42	2.240e-42	2.251e-42
419.70	1.0802	1.0856	5.173e-42	5.588e-42	5.616e-42
422.73	1.0790	1.0843	1.275e-41	1.376e-41	1.383e-41
425.76	1.0779	1.0831	3.104e-41	3.346e-41	3.362e-41
428.79	1.0768	1.0819	7.461e-41	8.034e-41	8.072e-41
431.82	1.0758	1.0807	1.771e-40	1.906e-40	1.914e-40
434.85	1.0747	1.0795	4.156e-40	4.466e-40	4.486e-40
437.88	1.0737	1.0783	9.635e-40	1.035e-39	1.039e-39
440.91	1.0727	1.0772	2.208e-39	2.369e-39	2.379e-39
443.94	1.0717	1.0761	5.005e-39	5.364e-39	5.386e-39
446.97	1.0707	1.0750	1.122e-38	1.201e-38	1.206e-38
450.00	1.0698	1.0740	2.487e-38	2.661e-38	2.672e-38

Pathway: EQ11-TS20-EQ1
Reactant Energy: 36.44 kcal/mol
TS Energy: 52.19 kcal/mol
Product Energy: 21.46 kcal/mol
Barrier Height: 15.75 kcal/mol
Imaginary Frequency: 659.98 cm^-1

Temperature(K)	Wigner_Factor	Eckart_Factor	TST Rate(s^{-1})	Wigner Rate (s^{-1})	Eckart Rate (s^{-1})
150.00	2.6698	14.6909	3.529e-11	9.421e-11	5.184e-10
153.03	2.6043	12.2734	1.025e-10	2.669e-10	1.258e-09
156.06	2.5426	10.4613	2.858e-10	7.266e-10	2.989e-09
159.09	2.4844	9.0704	7.664e-10	1.904e-09	6.952e-09
162.12	2.4294	7.9801	1.982e-09	4.815e-09	1.582e-08
165.15	2.3774	7.1096	4.951e-09	1.177e-08	3.520e-08
168.18	2.3282	6.4033	1.197e-08	2.787e-08	7.665e-08
171.21	2.2816	5.8217	2.806e-08	6.403e-08	1.634e-07
174.24	2.2375	5.3367	6.388e-08	1.429e-07	3.409e-07
177.27	2.1955	4.9276	1.414e-07	3.105e-07	6.969e-07
180.30	2.1557	4.5789	3.050e-07	6.574e-07	1.396e-06
183.33	2.1178	4.2790	6.412e-07	1.358e-06	2.744e-06
186.36	2.0817	4.0188	1.317e-06	2.741e-06	5.291e-06
189.39	2.0474	3.7915	2.642e-06	5.409e-06	1.002e-05
192.42	2.0147	3.5915	5.189e-06	1.045e-05	1.864e-05
195.45	1.9834	3.4144	9.981e-06	1.980e-05	3.408e-05
198.48	1.9536	3.2568	1.882e-05	3.678e-05	6.131e-05
201.52	1.9252	3.1157	3.484e-05	6.707e-05	1.086e-04
204.55	1.8980	2.9888	6.333e-05	1.202e-04	1.893e-04
207.58	1.8719	2.8741	1.132e-04	2.118e-04	3.252e-04
210.61	1.8470	2.7702	1.989e-04	3.673e-04	5.509e-04
213.64	1.8232	2.6755	3.440e-04	6.272e-04	9.204e-04
216.67	1.8003	2.5890	5.862e-04	1.055e-03	1.518e-03
219.70	1.7784	2.5098	9.845e-04	1.751e-03	2.471e-03
222.73	1.7573	2.4369	1.630e-03	2.865e-03	3.973e-03
225.76	1.7371	2.3698	2.665e-03	4.629e-03	6.314e-03
228.79	1.7177	2.3077	4.299e-03	7.384e-03	9.920e-03
231.82	1.6991	2.2501	6.851e-03	1.164e-02	1.542e-02
234.85	1.6812	2.1967	1.079e-02	1.814e-02	2.370e-02
237.88	1.6639	2.1470	1.680e-02	2.795e-02	3.606e-02
240.91	1.6473	2.1005	2.587e-02	4.261e-02	5.434e-02
243.94	1.6314	2.0572	3.942e-02	6.430e-02	8.109e-02
246.97	1.6160	2.0166	5.945e-02	9.607e-02	1.199e-01
250.00	1.6011	1.9785	8.880e-02	1.422e-01	1.757e-01
253.03	1.5868	1.9427	1.314e-01	2.085e-01	2.552e-01
256.06	1.5730	1.9090	1.926e-01	3.030e-01	3.677e-01
259.09	1.5597	1.8773	2.799e-01	4.366e-01	5.255e-01
262.12	1.5468	1.8473	4.033e-01	6.239e-01	7.451e-01
265.15	1.5344	1.8190	5.764e-01	8.844e-01	1.048e+00
268.18	1.5224	1.7923	8.172e-01	1.244e+00	1.465e+00
271.21	1.5108	1.7669	1.150e+00	1.737e+00	2.032e+00
274.24	1.4995	1.7428	1.606e+00	2.408e+00	2.799e+00
277.27	1.4887	1.7200	2.227e+00	3.315e+00	3.830e+00
280.30	1.4782	1.6982	3.066e+00	4.532e+00	5.207e+00
283.33	1.4680	1.6776	4.193e+00	6.156e+00	7.035e+00
286.36	1.4581	1.6579	5.698e+00	8.309e+00	9.447e+00

289.39	1.4486	1.6391	7.694e+00	1.115e+01	1.261e+01
292.42	1.4393	1.6212	1.033e+01	1.486e+01	1.674e+01
295.45	1.4304	1.6041	1.378e+01	1.971e+01	2.210e+01
298.48	1.4217	1.5877	1.828e+01	2.598e+01	2.902e+01
301.52	1.4133	1.5721	2.411e+01	3.407e+01	3.790e+01
304.55	1.4051	1.5571	3.163e+01	4.444e+01	4.925e+01
307.58	1.3971	1.5427	4.128e+01	5.767e+01	6.368e+01
310.61	1.3894	1.5289	5.360e+01	7.448e+01	8.195e+01
313.64	1.3819	1.5157	6.926e+01	9.571e+01	1.050e+02
316.67	1.3747	1.5030	8.906e+01	1.224e+02	1.339e+02
319.70	1.3676	1.4909	1.140e+02	1.559e+02	1.699e+02
322.73	1.3607	1.4791	1.452e+02	1.976e+02	2.148e+02
325.76	1.3540	1.4679	1.842e+02	2.494e+02	2.704e+02
328.79	1.3475	1.4570	2.327e+02	3.135e+02	3.390e+02
331.82	1.3412	1.4466	2.926e+02	3.925e+02	4.233e+02
334.85	1.3351	1.4365	3.665e+02	4.894e+02	5.265e+02
337.88	1.3291	1.4268	4.573e+02	6.078e+02	6.525e+02
340.91	1.3233	1.4174	5.684e+02	7.522e+02	8.057e+02
343.94	1.3176	1.4084	7.038e+02	9.274e+02	9.912e+02
346.97	1.3121	1.3996	8.683e+02	1.139e+03	1.215e+03
350.00	1.3067	1.3912	1.067e+03	1.395e+03	1.485e+03
353.03	1.3014	1.3830	1.308e+03	1.702e+03	1.809e+03
356.06	1.2963	1.3751	1.597e+03	2.070e+03	2.196e+03
359.09	1.2914	1.3675	1.943e+03	2.509e+03	2.657e+03
362.12	1.2865	1.3601	2.357e+03	3.032e+03	3.206e+03
365.15	1.2818	1.3530	2.850e+03	3.653e+03	3.856e+03
368.18	1.2771	1.3460	3.436e+03	4.388e+03	4.624e+03
371.21	1.2726	1.3393	4.129e+03	5.255e+03	5.531e+03
374.24	1.2682	1.3328	4.949e+03	6.276e+03	6.596e+03
377.27	1.2640	1.3265	5.914e+03	7.475e+03	7.845e+03
380.30	1.2598	1.3204	7.048e+03	8.878e+03	9.306e+03
383.33	1.2557	1.3145	8.376e+03	1.052e+04	1.101e+04
386.36	1.2517	1.3087	9.928e+03	1.243e+04	1.299e+04
389.39	1.2478	1.3031	1.174e+04	1.465e+04	1.530e+04
392.42	1.2440	1.2976	1.384e+04	1.722e+04	1.796e+04
395.45	1.2402	1.2924	1.628e+04	2.020e+04	2.105e+04
398.48	1.2366	1.2872	1.911e+04	2.363e+04	2.460e+04
401.52	1.2330	1.2822	2.238e+04	2.759e+04	2.869e+04
404.55	1.2296	1.2774	2.614e+04	3.214e+04	3.339e+04
407.58	1.2262	1.2726	3.046e+04	3.735e+04	3.877e+04
410.61	1.2228	1.2680	3.542e+04	4.332e+04	4.492e+04
413.64	1.2196	1.2635	4.111e+04	5.013e+04	5.194e+04
416.67	1.2164	1.2592	4.760e+04	5.790e+04	5.994e+04
419.70	1.2133	1.2549	5.500e+04	6.674e+04	6.903e+04
422.73	1.2102	1.2508	6.343e+04	7.677e+04	7.934e+04
425.76	1.2073	1.2467	7.301e+04	8.814e+04	9.102e+04
428.79	1.2043	1.2428	8.386e+04	1.010e+05	1.042e+05
431.82	1.2015	1.2390	9.615e+04	1.155e+05	1.191e+05

434.85	1.1987	1.2352	1.100e+05	1.319e+05	1.359e+05
437.88	1.1959	1.2316	1.257e+05	1.503e+05	1.548e+05
440.91	1.1933	1.2280	1.433e+05	1.710e+05	1.760e+05
443.94	1.1906	1.2245	1.632e+05	1.943e+05	1.998e+05
446.97	1.1881	1.2212	1.854e+05	2.203e+05	2.264e+05
450.00	1.1855	1.2178	2.104e+05	2.494e+05	2.562e+05

Pathway: EQ13-TS26-EQ12
Reactant Energy: 36.34 kcal/mol
TS Energy: 44.67 kcal/mol
Product Energy: 33.98 kcal/mol
Barrier Height: 8.33 kcal/mol
Imaginary Frequency: 605.25 cm^-1

Temperature(K)	Wigner_Factor	Eckart_Factor	TST Rate(s^{-1})	Wigner Rate (s^{-1})	Eckart Rate (s^{-1})
150.00	2.4043	6.3233	2.282e+00	5.487e+00	1.443e+01
153.03	2.3492	5.7486	4.049e+00	9.513e+00	2.328e+01
156.06	2.2974	5.2677	7.029e+00	1.615e+01	3.703e+01
159.09	2.2484	4.8610	1.195e+01	2.687e+01	5.810e+01
162.12	2.2022	4.5139	1.993e+01	4.389e+01	8.996e+01
165.15	2.1585	4.2151	3.263e+01	7.043e+01	1.375e+02
168.18	2.1171	3.9558	5.249e+01	1.111e+02	2.077e+02
171.21	2.0779	3.7292	8.307e+01	1.726e+02	3.098e+02
174.24	2.0407	3.5299	1.294e+02	2.641e+02	4.568e+02
177.27	2.0055	3.3535	1.986e+02	3.984e+02	6.661e+02
180.30	1.9719	3.1966	3.006e+02	5.928e+02	9.610e+02
183.33	1.9401	3.0562	4.489e+02	8.709e+02	1.372e+03
186.36	1.9098	2.9302	6.618e+02	1.264e+03	1.939e+03
189.39	1.8809	2.8164	9.639e+02	1.813e+03	2.715e+03
192.42	1.8533	2.7133	1.388e+03	2.572e+03	3.765e+03
195.45	1.8271	2.6196	1.976e+03	3.610e+03	5.176e+03
198.48	1.8020	2.5341	2.784e+03	5.017e+03	7.055e+03
201.52	1.7781	2.4558	3.883e+03	6.904e+03	9.536e+03
204.55	1.7552	2.3840	5.364e+03	9.415e+03	1.279e+04
207.58	1.7333	2.3178	7.342e+03	1.273e+04	1.702e+04
210.61	1.7124	2.2567	9.960e+03	1.706e+04	2.248e+04
213.64	1.6923	2.2002	1.340e+04	2.268e+04	2.948e+04
216.67	1.6731	2.1477	1.788e+04	2.992e+04	3.840e+04
219.70	1.6546	2.0989	2.368e+04	3.918e+04	4.970e+04
222.73	1.6369	2.0535	3.112e+04	5.094e+04	6.390e+04
225.76	1.6200	2.0111	4.061e+04	6.578e+04	8.166e+04
228.79	1.6036	1.9714	5.262e+04	8.439e+04	1.037e+05
231.82	1.5880	1.9343	6.775e+04	1.076e+05	1.310e+05
234.85	1.5729	1.8994	8.667e+04	1.363e+05	1.646e+05
237.88	1.5584	1.8666	1.102e+05	1.717e+05	2.057e+05
240.91	1.5444	1.8357	1.393e+05	2.151e+05	2.557e+05
243.94	1.5310	1.8066	1.751e+05	2.681e+05	3.163e+05
246.97	1.5180	1.7792	2.189e+05	3.323e+05	3.894e+05

250.00	1.5056	1.7532	2.722e+05	4.098e+05	4.772e+05
253.03	1.4935	1.7286	3.367e+05	5.029e+05	5.821e+05
256.06	1.4819	1.7053	4.146e+05	6.143e+05	7.070e+05
259.09	1.4707	1.6832	5.080e+05	7.471e+05	8.551e+05
262.12	1.4599	1.6623	6.196e+05	9.046e+05	1.030e+06
265.15	1.4494	1.6423	7.525e+05	1.091e+06	1.236e+06
268.18	1.4393	1.6233	9.099e+05	1.310e+06	1.477e+06
271.21	1.4296	1.6052	1.096e+06	1.567e+06	1.759e+06
274.24	1.4201	1.5880	1.314e+06	1.867e+06	2.087e+06
277.27	1.4110	1.5715	1.571e+06	2.216e+06	2.468e+06
280.30	1.4022	1.5558	1.870e+06	2.622e+06	2.909e+06
283.33	1.3936	1.5408	2.218e+06	3.090e+06	3.417e+06
286.36	1.3853	1.5264	2.621e+06	3.631e+06	4.001e+06
289.39	1.3773	1.5126	3.088e+06	4.253e+06	4.671e+06
292.42	1.3695	1.4994	3.625e+06	4.965e+06	5.436e+06
295.45	1.3620	1.4868	4.243e+06	5.779e+06	6.309e+06
298.48	1.3547	1.4746	4.951e+06	6.706e+06	7.300e+06
301.52	1.3476	1.4630	5.759e+06	7.761e+06	8.425e+06
304.55	1.3407	1.4518	6.680e+06	8.956e+06	9.698e+06
307.58	1.3340	1.4410	7.726e+06	1.031e+07	1.113e+07
310.61	1.3275	1.4307	8.912e+06	1.183e+07	1.275e+07
313.64	1.3212	1.4207	1.025e+07	1.355e+07	1.456e+07
316.67	1.3151	1.4111	1.176e+07	1.547e+07	1.660e+07
319.70	1.3091	1.4018	1.346e+07	1.763e+07	1.887e+07
322.73	1.3034	1.3929	1.537e+07	2.004e+07	2.141e+07
325.76	1.2978	1.3843	1.751e+07	2.272e+07	2.424e+07
328.79	1.2923	1.3760	1.990e+07	2.571e+07	2.738e+07
331.82	1.2870	1.3680	2.256e+07	2.903e+07	3.086e+07
334.85	1.2818	1.3602	2.552e+07	3.272e+07	3.472e+07
337.88	1.2768	1.3527	2.881e+07	3.679e+07	3.898e+07
340.91	1.2719	1.3455	3.246e+07	4.129e+07	4.368e+07
343.94	1.2671	1.3385	3.650e+07	4.625e+07	4.885e+07
346.97	1.2625	1.3317	4.096e+07	5.170e+07	5.454e+07
350.00	1.2579	1.3251	4.587e+07	5.770e+07	6.078e+07
353.03	1.2535	1.3188	5.127e+07	6.427e+07	6.762e+07
356.06	1.2492	1.3126	5.721e+07	7.147e+07	7.510e+07
359.09	1.2450	1.3066	6.373e+07	7.934e+07	8.327e+07
362.12	1.2410	1.3009	7.086e+07	8.793e+07	9.218e+07
365.15	1.2370	1.2952	7.866e+07	9.730e+07	1.019e+08
368.18	1.2331	1.2898	8.717e+07	1.075e+08	1.124e+08
371.21	1.2293	1.2845	9.645e+07	1.186e+08	1.239e+08
374.24	1.2256	1.2794	1.065e+08	1.306e+08	1.363e+08
377.27	1.2220	1.2744	1.175e+08	1.436e+08	1.498e+08
380.30	1.2185	1.2695	1.294e+08	1.577e+08	1.643e+08
383.33	1.2150	1.2648	1.423e+08	1.729e+08	1.800e+08
386.36	1.2117	1.2602	1.563e+08	1.894e+08	1.970e+08
389.39	1.2084	1.2558	1.714e+08	2.071e+08	2.153e+08
392.42	1.2052	1.2514	1.877e+08	2.262e+08	2.349e+08

395.45	1.2020	1.2472	2.053e+08	2.468e+08	2.561e+08
398.48	1.1990	1.2431	2.242e+08	2.689e+08	2.788e+08
401.52	1.1960	1.2391	2.446e+08	2.926e+08	3.031e+08
404.55	1.1931	1.2352	2.665e+08	3.180e+08	3.292e+08
407.58	1.1902	1.2314	2.900e+08	3.452e+08	3.571e+08
410.61	1.1874	1.2277	3.152e+08	3.743e+08	3.870e+08
413.64	1.1847	1.2241	3.422e+08	4.054e+08	4.189e+08
416.67	1.1820	1.2206	3.711e+08	4.386e+08	4.529e+08
419.70	1.1794	1.2171	4.019e+08	4.740e+08	4.892e+08
422.73	1.1768	1.2138	4.349e+08	5.118e+08	5.278e+08
425.76	1.1743	1.2105	4.700e+08	5.520e+08	5.690e+08
428.79	1.1719	1.2073	5.075e+08	5.947e+08	6.127e+08
431.82	1.1695	1.2042	5.474e+08	6.401e+08	6.591e+08
434.85	1.1671	1.2012	5.898e+08	6.883e+08	7.084e+08
437.88	1.1648	1.1982	6.349e+08	7.395e+08	7.607e+08
440.91	1.1625	1.1953	6.827e+08	7.937e+08	8.161e+08
443.94	1.1603	1.1925	7.335e+08	8.511e+08	8.747e+08
446.97	1.1582	1.1897	7.873e+08	9.119e+08	9.367e+08
450.00	1.1560	1.1870	8.443e+08	9.761e+08	1.002e+09

Pathway: EQ8-TS32-EQ12
Reactant Energy: 43.38 kcal/mol
TS Energy: 90.9 kcal/mol
Product Energy: 33.98 kcal/mol
Barrier Height: 47.52 kcal/mol
Imaginary Frequency: 387.95 cm^-1

Temperature(K)	Wigner_Factor	Eckart_Factor	TST Rate(s^{-1})	Wigner Rate (s^{-1})	Eckart Rate (s^{-1})
150.00	1.5770	1.9368	1.819e-57	2.868e-57	3.522e-57
153.03	1.5543	1.8799	4.359e-56	6.775e-56	8.195e-56
156.06	1.5330	1.8285	9.240e-55	1.416e-54	1.689e-54
159.09	1.5129	1.7819	1.744e-53	2.639e-53	3.108e-53
162.12	1.4939	1.7394	2.951e-52	4.408e-52	5.133e-52
165.15	1.4760	1.7006	4.502e-51	6.644e-51	7.655e-51
168.18	1.4590	1.6651	6.227e-50	9.085e-50	1.037e-49
171.21	1.4429	1.6323	7.852e-49	1.133e-48	1.282e-48
174.24	1.4276	1.6022	9.068e-48	1.295e-47	1.453e-47
177.27	1.4131	1.5743	9.635e-47	1.361e-46	1.517e-46
180.30	1.3993	1.5484	9.458e-46	1.323e-45	1.465e-45
183.33	1.3862	1.5244	8.612e-45	1.194e-44	1.313e-44
186.36	1.3738	1.5021	7.299e-44	1.003e-43	1.096e-43
189.39	1.3619	1.4812	5.780e-43	7.872e-43	8.561e-43
192.42	1.3506	1.4617	4.289e-42	5.792e-42	6.269e-42
195.45	1.3398	1.4434	2.991e-41	4.008e-41	4.318e-41
198.48	1.3295	1.4263	1.967e-40	2.615e-40	2.805e-40
201.52	1.3197	1.4102	1.222e-39	1.613e-39	1.724e-39
204.55	1.3103	1.3951	7.197e-39	9.430e-39	1.004e-38
207.58	1.3013	1.3808	4.025e-38	5.237e-38	5.557e-38

210.61	1.2927	1.3674	2.142e-37	2.769e-37	2.929e-37
213.64	1.2844	1.3546	1.088e-36	1.397e-36	1.474e-36
216.67	1.2765	1.3426	5.279e-36	6.739e-36	7.088e-36
219.70	1.2690	1.3312	2.453e-35	3.113e-35	3.266e-35
222.73	1.2617	1.3204	1.093e-34	1.380e-34	1.444e-34
225.76	1.2547	1.3102	4.683e-34	5.876e-34	6.136e-34
228.79	1.2480	1.3004	1.930e-33	2.409e-33	2.510e-33
231.82	1.2416	1.2911	7.669e-33	9.521e-33	9.901e-33
234.85	1.2354	1.2823	2.940e-32	3.633e-32	3.771e-32
237.88	1.2294	1.2739	1.090e-31	1.340e-31	1.388e-31
240.91	1.2237	1.2659	3.908e-31	4.782e-31	4.947e-31
243.94	1.2182	1.2582	1.358e-30	1.654e-30	1.709e-30
246.97	1.2128	1.2509	4.578e-30	5.552e-30	5.726e-30
250.00	1.2077	1.2439	1.498e-29	1.810e-29	1.864e-29
253.03	1.2028	1.2372	4.768e-29	5.735e-29	5.900e-29
256.06	1.1980	1.2308	1.477e-28	1.769e-28	1.817e-28
259.09	1.1934	1.2247	4.454e-28	5.315e-28	5.454e-28
262.12	1.1889	1.2188	1.310e-27	1.557e-27	1.596e-27
265.15	1.1846	1.2131	3.758e-27	4.452e-27	4.559e-27
268.18	1.1805	1.2077	1.053e-26	1.243e-26	1.272e-26
271.21	1.1765	1.2025	2.884e-26	3.393e-26	3.468e-26
274.24	1.1726	1.1975	7.727e-26	9.060e-26	9.252e-26
277.27	1.1689	1.1926	2.026e-25	2.368e-25	2.416e-25
280.30	1.1652	1.1880	5.203e-25	6.063e-25	6.181e-25
283.33	1.1617	1.1835	1.310e-24	1.522e-24	1.550e-24
286.36	1.1583	1.1792	3.234e-24	3.745e-24	3.813e-24
289.39	1.1550	1.1751	7.834e-24	9.049e-24	9.206e-24
292.42	1.1518	1.1711	1.864e-23	2.147e-23	2.183e-23
295.45	1.1487	1.1672	4.356e-23	5.004e-23	5.085e-23
298.48	1.1457	1.1635	1.001e-22	1.147e-22	1.165e-22
301.52	1.1428	1.1599	2.262e-22	2.585e-22	2.624e-22
304.55	1.1400	1.1564	5.030e-22	5.734e-22	5.817e-22
307.58	1.1372	1.1530	1.101e-21	1.252e-21	1.270e-21
310.61	1.1346	1.1498	2.374e-21	2.694e-21	2.730e-21
313.64	1.1320	1.1466	5.044e-21	5.710e-21	5.784e-21
316.67	1.1295	1.1436	1.056e-20	1.193e-20	1.208e-20
319.70	1.1270	1.1406	2.182e-20	2.459e-20	2.489e-20
322.73	1.1246	1.1378	4.445e-20	5.000e-20	5.058e-20
325.76	1.1223	1.1350	8.940e-20	1.003e-19	1.015e-19
328.79	1.1201	1.1323	1.775e-19	1.988e-19	2.010e-19
331.82	1.1179	1.1297	3.480e-19	3.891e-19	3.932e-19
334.85	1.1158	1.1272	6.742e-19	7.523e-19	7.600e-19
337.88	1.1137	1.1248	1.291e-18	1.438e-18	1.452e-18
340.91	1.1117	1.1224	2.443e-18	2.716e-18	2.742e-18
343.94	1.1097	1.1201	4.573e-18	5.075e-18	5.122e-18
346.97	1.1078	1.1178	8.467e-18	9.380e-18	9.465e-18
350.00	1.1060	1.1157	1.551e-17	1.716e-17	1.731e-17
353.03	1.1042	1.1136	2.813e-17	3.106e-17	3.132e-17

356.06	1.1024	1.1115	5.049e-17	5.566e-17	5.612e-17
359.09	1.1007	1.1095	8.974e-17	9.878e-17	9.957e-17
362.12	1.0990	1.1076	1.580e-16	1.736e-16	1.750e-16
365.15	1.0974	1.1057	2.756e-16	3.024e-16	3.047e-16
368.18	1.0958	1.1038	4.764e-16	5.220e-16	5.258e-16
371.21	1.0942	1.1020	8.162e-16	8.930e-16	8.994e-16
374.24	1.0927	1.1003	1.386e-15	1.515e-15	1.525e-15
377.27	1.0912	1.0986	2.335e-15	2.548e-15	2.565e-15
380.30	1.0898	1.0969	3.900e-15	4.250e-15	4.278e-15
383.33	1.0883	1.0953	6.462e-15	7.033e-15	7.078e-15
386.36	1.0870	1.0937	1.062e-14	1.155e-14	1.162e-14
389.39	1.0856	1.0922	1.733e-14	1.882e-14	1.893e-14
392.42	1.0843	1.0907	2.806e-14	3.043e-14	3.061e-14
395.45	1.0830	1.0893	4.511e-14	4.886e-14	4.914e-14
398.48	1.0818	1.0878	7.200e-14	7.788e-14	7.832e-14
401.52	1.0805	1.0865	1.141e-13	1.233e-13	1.240e-13
404.55	1.0793	1.0851	1.796e-13	1.938e-13	1.949e-13
407.58	1.0781	1.0838	2.808e-13	3.028e-13	3.043e-13
410.61	1.0770	1.0825	4.362e-13	4.698e-13	4.722e-13
413.64	1.0759	1.0812	6.732e-13	7.243e-13	7.279e-13
416.67	1.0748	1.0800	1.033e-12	1.110e-12	1.115e-12
419.70	1.0737	1.0788	1.574e-12	1.690e-12	1.698e-12
422.73	1.0726	1.0776	2.385e-12	2.559e-12	2.571e-12
425.76	1.0716	1.0765	3.593e-12	3.851e-12	3.868e-12
428.79	1.0706	1.0753	5.382e-12	5.762e-12	5.788e-12
431.82	1.0696	1.0742	8.017e-12	8.575e-12	8.612e-12
434.85	1.0687	1.0732	1.188e-11	1.269e-11	1.274e-11
437.88	1.0677	1.0721	1.750e-11	1.868e-11	1.876e-11
440.91	1.0668	1.0711	2.564e-11	2.735e-11	2.746e-11
443.94	1.0659	1.0701	3.738e-11	3.985e-11	4.000e-11
446.97	1.0650	1.0691	5.423e-11	5.775e-11	5.798e-11
450.00	1.0641	1.0681	7.828e-11	8.330e-11	8.361e-11

Pathway: EQ7-TS42-EQ16
Reactant Energy: -4.91 kcal/mol
TS Energy: 71.71 kcal/mol
Product Energy: 70.75 kcal/mol
Barrier Height: 76.62 kcal/mol
Imaginary Frequency: 467.34 cm^-1

Temperature(K)	Wigner_Factor	Eckart_Factor	TST Rate(s^{-1})	Wigner Rate (s^{-1})	Eckart Rate (s^{-1})
150.00	1.8373	1.8106	7.276e-100	1.337e-99	1.317e-99
153.03	1.8044	1.7697	1.205e-97	2.175e-97	2.133e-97
156.06	1.7735	1.7320	1.638e-95	2.905e-95	2.837e-95
159.09	1.7443	1.6971	1.847e-93	3.222e-93	3.135e-93
162.12	1.7167	1.6647	1.746e-91	2.997e-91	2.906e-91
165.15	1.6907	1.6345	1.397e-89	2.362e-89	2.284e-89
168.18	1.6660	1.6064	9.550e-88	1.591e-87	1.534e-87

171.21	1.6426	1.5802	5.623e-86	9.237e-86	8.886e-86
174.24	1.6205	1.5556	2.874e-84	4.658e-84	4.471e-84
177.27	1.5995	1.5327	1.285e-82	2.055e-82	1.969e-82
180.30	1.5795	1.5111	5.054e-81	7.983e-81	7.638e-81
183.33	1.5605	1.4909	1.762e-79	2.749e-79	2.627e-79
186.36	1.5424	1.4718	5.473e-78	8.441e-78	8.055e-78
189.39	1.5252	1.4539	1.523e-76	2.324e-76	2.215e-76
192.42	1.5088	1.4369	3.820e-75	5.764e-75	5.489e-75
195.45	1.4931	1.4209	8.670e-74	1.295e-73	1.232e-73
198.48	1.4782	1.4058	1.789e-72	2.645e-72	2.515e-72
201.52	1.4639	1.3915	3.372e-71	4.936e-71	4.692e-71
204.55	1.4503	1.3779	5.826e-70	8.450e-70	8.028e-70
207.58	1.4372	1.3651	9.266e-69	1.332e-68	1.265e-68
210.61	1.4247	1.3528	1.361e-67	1.939e-67	1.841e-67
213.64	1.4128	1.3412	1.853e-66	2.618e-66	2.485e-66
216.67	1.4013	1.3302	2.345e-65	3.287e-65	3.120e-65
219.70	1.3903	1.3196	2.768e-64	3.849e-64	3.653e-64
222.73	1.3797	1.3096	3.056e-63	4.217e-63	4.002e-63
225.76	1.3696	1.3000	3.164e-62	4.333e-62	4.113e-62
228.79	1.3599	1.2909	3.079e-61	4.187e-61	3.975e-61
231.82	1.3505	1.2822	2.824e-60	3.814e-60	3.621e-60
234.85	1.3416	1.2738	2.446e-59	3.282e-59	3.116e-59
237.88	1.3329	1.2658	2.006e-58	2.674e-58	2.540e-58
240.91	1.3246	1.2582	1.561e-57	2.067e-57	1.964e-57
243.94	1.3166	1.2509	1.154e-56	1.519e-56	1.443e-56
246.97	1.3089	1.2438	8.125e-56	1.063e-55	1.011e-55
250.00	1.3014	1.2371	5.457e-55	7.102e-55	6.751e-55
253.03	1.2942	1.2306	3.502e-54	4.533e-54	4.310e-54
256.06	1.2873	1.2244	2.151e-53	2.769e-53	2.634e-53
259.09	1.2806	1.2185	1.267e-52	1.622e-52	1.543e-52
262.12	1.2742	1.2127	7.159e-52	9.122e-52	8.682e-52
265.15	1.2679	1.2072	3.890e-51	4.932e-51	4.696e-51
268.18	1.2619	1.2019	2.034e-50	2.567e-50	2.445e-50
271.21	1.2561	1.1968	1.026e-49	1.288e-49	1.227e-49
274.24	1.2505	1.1918	4.989e-49	6.239e-49	5.946e-49
277.27	1.2450	1.1871	2.345e-48	2.919e-48	2.783e-48
280.30	1.2398	1.1825	1.066e-47	1.321e-47	1.260e-47
283.33	1.2347	1.1781	4.691e-47	5.792e-47	5.527e-47
286.36	1.2297	1.1738	2.001e-46	2.461e-46	2.349e-46
289.39	1.2249	1.1697	8.283e-46	1.015e-45	9.688e-46
292.42	1.2203	1.1657	3.329e-45	4.062e-45	3.881e-45
295.45	1.2158	1.1619	1.300e-44	1.581e-44	1.511e-44
298.48	1.2114	1.1581	4.942e-44	5.987e-44	5.724e-44
301.52	1.2072	1.1545	1.829e-43	2.207e-43	2.111e-43
304.55	1.2031	1.1510	6.592e-43	7.932e-43	7.588e-43
307.58	1.1991	1.1477	2.318e-42	2.779e-42	2.660e-42
310.61	1.1953	1.1444	7.952e-42	9.504e-42	9.100e-42
313.64	1.1915	1.1412	2.664e-41	3.174e-41	3.040e-41

316.67	1.1879	1.1382	8.722e-41	1.036e-40	9.927e-41
319.70	1.1843	1.1352	2.792e-40	3.307e-40	3.170e-40
322.73	1.1809	1.1323	8.748e-40	1.033e-39	9.905e-40
325.76	1.1775	1.1295	2.683e-39	3.159e-39	3.030e-39
328.79	1.1743	1.1268	8.061e-39	9.466e-39	9.083e-39
331.82	1.1711	1.1242	2.374e-38	2.780e-38	2.669e-38
334.85	1.1680	1.1216	6.857e-38	8.009e-38	7.691e-38
337.88	1.1650	1.1191	1.943e-37	2.264e-37	2.175e-37
340.91	1.1621	1.1167	5.407e-37	6.283e-37	6.038e-37
343.94	1.1592	1.1144	1.478e-36	1.713e-36	1.647e-36
346.97	1.1565	1.1121	3.968e-36	4.589e-36	4.413e-36
350.00	1.1538	1.1099	1.048e-35	1.209e-35	1.163e-35
353.03	1.1512	1.1078	2.720e-35	3.131e-35	3.013e-35
356.06	1.1486	1.1057	6.950e-35	7.982e-35	7.684e-35
359.09	1.1461	1.1036	1.748e-34	2.003e-34	1.929e-34
362.12	1.1437	1.1016	4.329e-34	4.951e-34	4.769e-34
365.15	1.1413	1.0997	1.056e-33	1.205e-33	1.162e-33
368.18	1.1390	1.0978	2.540e-33	2.893e-33	2.788e-33
371.21	1.1367	1.0960	6.020e-33	6.843e-33	6.598e-33
374.24	1.1345	1.0942	1.407e-32	1.597e-32	1.540e-32
377.27	1.1324	1.0925	3.245e-32	3.675e-32	3.546e-32
380.30	1.1303	1.0908	7.386e-32	8.348e-32	8.056e-32
383.33	1.1282	1.0891	1.659e-31	1.872e-31	1.807e-31
386.36	1.1262	1.0875	3.681e-31	4.145e-31	4.003e-31
389.39	1.1242	1.0860	8.065e-31	9.067e-31	8.758e-31
392.42	1.1223	1.0844	1.746e-30	1.960e-30	1.893e-30
395.45	1.1205	1.0829	3.736e-30	4.186e-30	4.045e-30
398.48	1.1186	1.0815	7.901e-30	8.838e-30	8.545e-30
401.52	1.1169	1.0800	1.652e-29	1.845e-29	1.785e-29
404.55	1.1151	1.0787	3.418e-29	3.811e-29	3.687e-29
407.58	1.1134	1.0773	6.995e-29	7.788e-29	7.535e-29
410.61	1.1117	1.0760	1.416e-28	1.575e-28	1.524e-28
413.64	1.1101	1.0747	2.839e-28	3.151e-28	3.051e-28
416.67	1.1085	1.0734	5.633e-28	6.244e-28	6.046e-28
419.70	1.1069	1.0722	1.107e-27	1.225e-27	1.187e-27
422.73	1.1054	1.0710	2.154e-27	2.381e-27	2.306e-27
425.76	1.1039	1.0698	4.152e-27	4.583e-27	4.441e-27
428.79	1.1025	1.0687	7.929e-27	8.742e-27	8.474e-27
431.82	1.1010	1.0675	1.501e-26	1.652e-26	1.602e-26
434.85	1.0996	1.0664	2.816e-26	3.096e-26	3.003e-26
437.88	1.0983	1.0653	5.237e-26	5.752e-26	5.580e-26
440.91	1.0969	1.0643	9.659e-26	1.059e-25	1.028e-25
443.94	1.0956	1.0633	1.767e-25	1.935e-25	1.878e-25
446.97	1.0943	1.0623	3.205e-25	3.507e-25	3.404e-25
450.00	1.0930	1.0613	5.768e-25	6.305e-25	6.121e-25

Pathway: EQ2-TS49-EQ0
Reactant Energy: 57.93 kcal/mol

TS Energy: 87.45 kcal/mol
Product Energy: 0.0 kcal/mol
Barrier Height: 29.52 kcal/mol
Imaginary Frequency: 506.28 cm^-1

Temperature(K)	Wigner_Factor	Eckart_Factor	TST Rate(s^{-1})	Wigner Rate (s^{-1})	Eckart Rate (s^{-1})
150.00	1.9826	3.5676	3.056e-31	6.059e-31	1.090e-30
153.03	1.9441	3.3358	2.216e-30	4.308e-30	7.391e-30
156.06	1.9078	3.1376	1.488e-29	2.839e-29	4.669e-29
159.09	1.8735	2.9665	9.299e-29	1.742e-28	2.758e-28
162.12	1.8412	2.8175	5.428e-28	9.993e-28	1.529e-27
165.15	1.8106	2.6867	2.970e-27	5.378e-27	7.980e-27
168.18	1.7816	2.5711	1.530e-26	2.725e-26	3.933e-26
171.21	1.7542	2.4682	7.435e-26	1.304e-25	1.835e-25
174.24	1.7282	2.3763	3.421e-25	5.913e-25	8.130e-25
177.27	1.7035	2.2936	1.495e-24	2.547e-24	3.429e-24
180.30	1.6801	2.2190	6.217e-24	1.045e-23	1.380e-23
183.33	1.6578	2.1513	2.468e-23	4.091e-23	5.309e-23
186.36	1.6366	2.0897	9.367e-23	1.533e-22	1.957e-22
189.39	1.6163	2.0333	3.408e-22	5.508e-22	6.929e-22
192.42	1.5971	1.9817	1.191e-21	1.902e-21	2.360e-21
195.45	1.5787	1.9342	4.003e-21	6.320e-21	7.743e-21
198.48	1.5612	1.8904	1.297e-20	2.025e-20	2.452e-20
201.52	1.5444	1.8499	4.058e-20	6.268e-20	7.508e-20
204.55	1.5284	1.8124	1.228e-19	1.877e-19	2.225e-19
207.58	1.5131	1.7775	3.597e-19	5.443e-19	6.394e-19
210.61	1.4984	1.7450	1.022e-18	1.531e-18	1.783e-18
213.64	1.4844	1.7146	2.819e-18	4.185e-18	4.834e-18
216.67	1.4709	1.6863	7.562e-18	1.112e-17	1.275e-17
219.70	1.4580	1.6597	1.974e-17	2.878e-17	3.276e-17
222.73	1.4457	1.6348	5.022e-17	7.260e-17	8.209e-17
225.76	1.4338	1.6114	1.246e-16	1.787e-16	2.008e-16
228.79	1.4224	1.5893	3.019e-16	4.294e-16	4.798e-16
231.82	1.4114	1.5685	7.147e-16	1.009e-15	1.121e-15
234.85	1.4008	1.5489	1.655e-15	2.319e-15	2.564e-15
237.88	1.3907	1.5304	3.753e-15	5.219e-15	5.744e-15
240.91	1.3809	1.5128	8.337e-15	1.151e-14	1.261e-14
243.94	1.3715	1.4962	1.816e-14	2.491e-14	2.717e-14
246.97	1.3625	1.4804	3.881e-14	5.288e-14	5.746e-14
250.00	1.3537	1.4654	8.146e-14	1.103e-13	1.194e-13
253.03	1.3453	1.4511	1.680e-13	2.260e-13	2.437e-13
256.06	1.3372	1.4376	3.405e-13	4.553e-13	4.895e-13
259.09	1.3293	1.4247	6.790e-13	9.027e-13	9.674e-13
262.12	1.3218	1.4123	1.333e-12	1.762e-12	1.883e-12
265.15	1.3145	1.4006	2.577e-12	3.387e-12	3.609e-12
268.18	1.3074	1.3893	4.909e-12	6.417e-12	6.820e-12
271.21	1.3006	1.3786	9.218e-12	1.199e-11	1.271e-11
274.24	1.2940	1.3683	1.707e-11	2.209e-11	2.336e-11

277.27	1.2876	1.3585	3.120e-11	4.017e-11	4.239e-11
280.30	1.2814	1.3491	5.629e-11	7.213e-11	7.594e-11
283.33	1.2754	1.3400	1.003e-10	1.279e-10	1.344e-10
286.36	1.2696	1.3314	1.765e-10	2.241e-10	2.350e-10
289.39	1.2640	1.3231	3.071e-10	3.882e-10	4.063e-10
292.42	1.2585	1.3151	5.283e-10	6.648e-10	6.947e-10
295.45	1.2533	1.3074	8.987e-10	1.126e-09	1.175e-09
298.48	1.2481	1.3000	1.513e-09	1.888e-09	1.966e-09
301.52	1.2432	1.2929	2.520e-09	3.132e-09	3.257e-09
304.55	1.2384	1.2860	4.155e-09	5.145e-09	5.343e-09
307.58	1.2337	1.2794	6.786e-09	8.371e-09	8.681e-09
310.61	1.2292	1.2730	1.098e-08	1.349e-08	1.397e-08
313.64	1.2248	1.2669	1.759e-08	2.155e-08	2.229e-08
316.67	1.2205	1.2610	2.795e-08	3.411e-08	3.524e-08
319.70	1.2163	1.2552	4.402e-08	5.354e-08	5.525e-08
322.73	1.2123	1.2497	6.874e-08	8.333e-08	8.591e-08
325.76	1.2083	1.2444	1.065e-07	1.287e-07	1.325e-07
328.79	1.2045	1.2392	1.636e-07	1.971e-07	2.027e-07
331.82	1.2008	1.2342	2.494e-07	2.995e-07	3.079e-07
334.85	1.1972	1.2294	3.775e-07	4.519e-07	4.640e-07
337.88	1.1937	1.2247	5.670e-07	6.768e-07	6.944e-07
340.91	1.1902	1.2202	8.456e-07	1.006e-06	1.032e-06
343.94	1.1869	1.2158	1.252e-06	1.487e-06	1.523e-06
346.97	1.1836	1.2115	1.842e-06	2.181e-06	2.232e-06
350.00	1.1805	1.2074	2.692e-06	3.178e-06	3.251e-06
353.03	1.1774	1.2034	3.909e-06	4.603e-06	4.705e-06
356.06	1.1744	1.1995	5.641e-06	6.625e-06	6.766e-06
359.09	1.1715	1.1958	8.090e-06	9.477e-06	9.673e-06
362.12	1.1686	1.1921	1.153e-05	1.348e-05	1.375e-05
365.15	1.1658	1.1886	1.635e-05	1.906e-05	1.943e-05
368.18	1.1631	1.1851	2.304e-05	2.679e-05	2.730e-05
371.21	1.1604	1.1818	3.228e-05	3.746e-05	3.815e-05
374.24	1.1579	1.1785	4.500e-05	5.211e-05	5.304e-05
377.27	1.1553	1.1753	6.241e-05	7.210e-05	7.335e-05
380.30	1.1529	1.1722	8.609e-05	9.925e-05	1.009e-04
383.33	1.1505	1.1692	1.182e-04	1.359e-04	1.382e-04
386.36	1.1481	1.1663	1.614e-04	1.853e-04	1.883e-04
389.39	1.1458	1.1635	2.194e-04	2.514e-04	2.553e-04
392.42	1.1436	1.1607	2.969e-04	3.395e-04	3.446e-04
395.45	1.1414	1.1580	3.998e-04	4.564e-04	4.630e-04
398.48	1.1392	1.1554	5.361e-04	6.108e-04	6.194e-04
401.52	1.1371	1.1528	7.157e-04	8.139e-04	8.251e-04
404.55	1.1351	1.1504	9.514e-04	1.080e-03	1.094e-03
407.58	1.1331	1.1479	1.259e-03	1.427e-03	1.446e-03
410.61	1.1311	1.1456	1.660e-03	1.878e-03	1.902e-03
413.64	1.1292	1.1432	2.180e-03	2.462e-03	2.493e-03
416.67	1.1273	1.1410	2.852e-03	3.215e-03	3.254e-03
419.70	1.1255	1.1388	3.716e-03	4.182e-03	4.231e-03

422.73	1.1237	1.1366	4.824e-03	5.420e-03	5.483e-03
425.76	1.1220	1.1345	6.239e-03	7.000e-03	7.078e-03
428.79	1.1202	1.1325	8.040e-03	9.007e-03	9.105e-03
431.82	1.1186	1.1305	1.033e-02	1.155e-02	1.167e-02
434.85	1.1169	1.1285	1.321e-02	1.476e-02	1.491e-02
437.88	1.1153	1.1266	1.686e-02	1.880e-02	1.899e-02
440.91	1.1137	1.1248	2.143e-02	2.387e-02	2.410e-02
443.94	1.1122	1.1229	2.715e-02	3.020e-02	3.049e-02
446.97	1.1107	1.1212	3.430e-02	3.810e-02	3.846e-02
450.00	1.1092	1.1194	4.320e-02	4.791e-02	4.836e-02

Pathway: EQ3-TS77-EQ20
Reactant Energy: -10.56 kcal/mol
TS Energy: 86.05 kcal/mol
Product Energy: 77.45 kcal/mol
Barrier Height: 96.61 kcal/mol
Imaginary Frequency: 504.66 cm^-1

Temperature(K)	Wigner_Factor	Eckart_Factor	TST Rate(s^{-1})	Wigner Rate (s^{-1})	Eckart Rate (s^{-1})
150.00	1.9763	3.2244	5.458e-129	1.079e-128	1.760e-128
153.03	1.9380	3.0466	3.412e-126	6.613e-126	1.040e-125
156.06	1.9020	2.8912	1.662e-123	3.161e-123	4.805e-123
159.09	1.8679	2.7544	6.396e-121	1.195e-120	1.762e-120
162.12	1.8358	2.6333	1.971e-118	3.619e-118	5.191e-118
165.15	1.8054	2.5255	4.925e-116	8.892e-116	1.244e-115
168.18	1.7766	2.4290	1.009e-113	1.792e-113	2.450e-113
171.21	1.7494	2.3421	1.712e-111	2.995e-111	4.010e-111
174.24	1.7235	2.2637	2.431e-109	4.190e-109	5.504e-109
177.27	1.6990	2.1925	2.915e-107	4.953e-107	6.392e-107
180.30	1.6757	2.1278	2.977e-105	4.988e-105	6.334e-105
183.33	1.6536	2.0686	2.609e-103	4.315e-103	5.398e-103
186.36	1.6325	2.0144	1.978e-101	3.229e-101	3.985e-101
189.39	1.6124	1.9645	1.306e-99	2.106e-99	2.566e-99
192.42	1.5933	1.9186	7.559e-98	1.204e-97	1.450e-97
195.45	1.5750	1.8761	3.858e-96	6.077e-96	7.239e-96
198.48	1.5576	1.8367	1.747e-94	2.721e-94	3.209e-94
201.52	1.5410	1.8002	7.055e-93	1.087e-92	1.270e-92
204.55	1.5250	1.7662	2.554e-91	3.895e-91	4.511e-91
207.58	1.5098	1.7344	8.327e-90	1.257e-89	1.444e-89
210.61	1.4953	1.7048	2.456e-88	3.673e-88	4.188e-88
213.64	1.4813	1.6771	6.584e-87	9.754e-87	1.104e-86
216.67	1.4679	1.6511	1.610e-85	2.364e-85	2.659e-85
219.70	1.4551	1.6266	3.606e-84	5.247e-84	5.865e-84
222.73	1.4428	1.6036	7.422e-83	1.071e-82	1.190e-82
225.76	1.4310	1.5820	1.409e-81	2.016e-81	2.228e-81
228.79	1.4197	1.5616	2.474e-80	3.512e-80	3.863e-80
231.82	1.4088	1.5423	4.031e-79	5.678e-79	6.216e-79
234.85	1.3983	1.5240	6.112e-78	8.547e-78	9.315e-78

237.88	1.3882	1.5067	8.651e-77	1.201e-76	1.303e-76
240.91	1.3785	1.4903	1.146e-75	1.579e-75	1.707e-75
243.94	1.3692	1.4748	1.423e-74	1.948e-74	2.098e-74
246.97	1.3602	1.4600	1.662e-73	2.260e-73	2.426e-73
250.00	1.3515	1.4460	1.828e-72	2.471e-72	2.644e-72
253.03	1.3431	1.4326	1.900e-71	2.552e-71	2.722e-71
256.06	1.3350	1.4198	1.868e-70	2.494e-70	2.652e-70
259.09	1.3272	1.4077	1.741e-69	2.311e-69	2.451e-69
262.12	1.3197	1.3961	1.542e-68	2.035e-68	2.152e-68
265.15	1.3125	1.3850	1.299e-67	1.705e-67	1.799e-67
268.18	1.3054	1.3743	1.043e-66	1.362e-66	1.433e-66
271.21	1.2986	1.3642	7.995e-66	1.038e-65	1.091e-65
274.24	1.2921	1.3545	5.860e-65	7.571e-65	7.937e-65
277.27	1.2857	1.3452	4.112e-64	5.287e-64	5.531e-64
280.30	1.2796	1.3362	2.767e-63	3.540e-63	3.697e-63
283.33	1.2736	1.3276	1.788e-62	2.277e-62	2.373e-62
286.36	1.2679	1.3194	1.110e-61	1.408e-61	1.465e-61
289.39	1.2623	1.3115	6.638e-61	8.380e-61	8.706e-61
292.42	1.2569	1.3039	3.825e-60	4.808e-60	4.987e-60
295.45	1.2516	1.2966	2.126e-59	2.661e-59	2.757e-59
298.48	1.2466	1.2895	1.142e-58	1.423e-58	1.472e-58
301.52	1.2416	1.2827	5.928e-58	7.360e-58	7.604e-58
304.55	1.2368	1.2762	2.978e-57	3.684e-57	3.801e-57
307.58	1.2322	1.2699	1.450e-56	1.787e-56	1.841e-56
310.61	1.2277	1.2638	6.844e-56	8.402e-56	8.649e-56
313.64	1.2233	1.2580	3.135e-55	3.835e-55	3.944e-55
316.67	1.2191	1.2523	1.395e-54	1.701e-54	1.747e-54
319.70	1.2149	1.2468	6.036e-54	7.334e-54	7.526e-54
322.73	1.2109	1.2415	2.541e-53	3.077e-53	3.155e-53
325.76	1.2070	1.2364	1.041e-52	1.257e-52	1.288e-52
328.79	1.2032	1.2315	4.159e-52	5.005e-52	5.122e-52
331.82	1.1995	1.2267	1.620e-51	1.943e-51	1.987e-51
334.85	1.1959	1.2220	6.155e-51	7.361e-51	7.522e-51
337.88	1.1924	1.2176	2.284e-50	2.723e-50	2.781e-50
340.91	1.1890	1.2132	8.280e-50	9.845e-50	1.004e-49
343.94	1.1857	1.2090	2.934e-49	3.479e-49	3.548e-49
346.97	1.1825	1.2049	1.017e-48	1.203e-48	1.226e-48
350.00	1.1793	1.2009	3.452e-48	4.071e-48	4.146e-48
353.03	1.1763	1.1971	1.147e-47	1.349e-47	1.373e-47
356.06	1.1733	1.1934	3.736e-47	4.383e-47	4.458e-47
359.09	1.1704	1.1898	1.192e-46	1.396e-46	1.419e-46
362.12	1.1675	1.1862	3.734e-46	4.359e-46	4.429e-46
365.15	1.1648	1.1828	1.147e-45	1.336e-45	1.357e-45
368.18	1.1620	1.1795	3.460e-45	4.021e-45	4.081e-45
371.21	1.1594	1.1763	1.025e-44	1.189e-44	1.206e-44
374.24	1.1568	1.1731	2.984e-44	3.453e-44	3.501e-44
377.27	1.1543	1.1701	8.541e-44	9.859e-44	9.994e-44
380.30	1.1519	1.1671	2.404e-43	2.769e-43	2.806e-43

383.33	1.1495	1.1642	6.656e-43	7.652e-43	7.750e-43
386.36	1.1472	1.1614	1.814e-42	2.081e-42	2.107e-42
389.39	1.1449	1.1587	4.868e-42	5.573e-42	5.640e-42
392.42	1.1426	1.1560	1.286e-41	1.470e-41	1.487e-41
395.45	1.1405	1.1534	3.350e-41	3.820e-41	3.864e-41
398.48	1.1383	1.1509	8.597e-41	9.786e-41	9.894e-41
401.52	1.1363	1.1484	2.175e-40	2.472e-40	2.498e-40
404.55	1.1342	1.1460	5.429e-40	6.157e-40	6.221e-40
407.58	1.1322	1.1436	1.336e-39	1.513e-39	1.528e-39
410.61	1.1303	1.1413	3.247e-39	3.670e-39	3.706e-39
413.64	1.1284	1.1391	7.787e-39	8.787e-39	8.871e-39
416.67	1.1265	1.1369	1.844e-38	2.077e-38	2.097e-38
419.70	1.1247	1.1348	4.313e-38	4.851e-38	4.895e-38
422.73	1.1229	1.1327	9.967e-38	1.119e-37	1.129e-37
425.76	1.1212	1.1307	2.276e-37	2.552e-37	2.573e-37
428.79	1.1195	1.1287	5.137e-37	5.751e-37	5.798e-37
431.82	1.1178	1.1268	1.146e-36	1.281e-36	1.292e-36
434.85	1.1162	1.1249	2.530e-36	2.824e-36	2.846e-36
437.88	1.1146	1.1230	5.522e-36	6.155e-36	6.201e-36
440.91	1.1130	1.1212	1.193e-35	1.327e-35	1.337e-35
443.94	1.1115	1.1195	2.549e-35	2.833e-35	2.853e-35
446.97	1.1100	1.1177	5.392e-35	5.985e-35	6.026e-35
450.00	1.1085	1.1160	1.129e-34	1.252e-34	1.260e-34

Pathway: EQ26-TS100-EQ7
Reactant Energy: 118.01 kcal/mol
TS Energy: 119.07 kcal/mol
Product Energy: -4.91 kcal/mol
Barrier Height: 1.06 kcal/mol
Imaginary Frequency: 783.66 cm^-1

Temperature(K)	Wigner_Factor	Eckart_Factor	TST Rate(s^{-1})	Wigner Rate (s^{-1})	Eckart Rate (s^{-1})
150.00	3.3542	3.3338	8.923e+10	2.993e+11	2.975e+11
153.03	3.2619	3.1972	9.768e+10	3.186e+11	3.123e+11
156.06	3.1749	3.0727	1.066e+11	3.384e+11	3.275e+11
159.09	3.0929	2.9588	1.160e+11	3.587e+11	3.431e+11
162.12	3.0154	2.8543	1.258e+11	3.794e+11	3.591e+11
165.15	2.9421	2.7582	1.361e+11	4.006e+11	3.755e+11
168.18	2.8727	2.6696	1.470e+11	4.222e+11	3.923e+11
171.21	2.8070	2.5878	1.582e+11	4.442e+11	4.095e+11
174.24	2.7447	2.5120	1.700e+11	4.666e+11	4.270e+11
177.27	2.6856	2.4416	1.823e+11	4.895e+11	4.450e+11
180.30	2.6294	2.3762	1.950e+11	5.127e+11	4.633e+11
183.33	2.5760	2.3153	2.082e+11	5.363e+11	4.820e+11
186.36	2.5251	2.2585	2.219e+11	5.603e+11	5.011e+11
189.39	2.4767	2.2053	2.361e+11	5.847e+11	5.206e+11
192.42	2.4306	2.1556	2.507e+11	6.094e+11	5.404e+11
195.45	2.3866	2.1089	2.659e+11	6.345e+11	5.607e+11

198.48	2.3445	2.0651	2.815e+11	6.599e+11	5.812e+11
201.52	2.3044	2.0239	2.975e+11	6.857e+11	6.022e+11
204.55	2.2661	1.9851	3.141e+11	7.118e+11	6.235e+11
207.58	2.2294	1.9485	3.311e+11	7.382e+11	6.452e+11
210.61	2.1942	1.9139	3.486e+11	7.649e+11	6.672e+11
213.64	2.1606	1.8813	3.666e+11	7.920e+11	6.896e+11
216.67	2.1284	1.8503	3.850e+11	8.194e+11	7.123e+11
219.70	2.0974	1.8211	4.038e+11	8.470e+11	7.354e+11
222.73	2.0678	1.7933	4.232e+11	8.750e+11	7.588e+11
225.76	2.0393	1.7669	4.429e+11	9.033e+11	7.826e+11
228.79	2.0120	1.7419	4.631e+11	9.318e+11	8.067e+11
231.82	1.9857	1.7180	4.838e+11	9.607e+11	8.312e+11
234.85	1.9604	1.6953	5.049e+11	9.898e+11	8.560e+11
237.88	1.9361	1.6737	5.264e+11	1.019e+12	8.811e+11
240.91	1.9127	1.6531	5.484e+11	1.049e+12	9.065e+11
243.94	1.8902	1.6334	5.708e+11	1.079e+12	9.323e+11
246.97	1.8684	1.6146	5.936e+11	1.109e+12	9.584e+11
250.00	1.8475	1.5966	6.168e+11	1.140e+12	9.848e+11
253.03	1.8273	1.5794	6.404e+11	1.170e+12	1.011e+12
256.06	1.8079	1.5629	6.645e+11	1.201e+12	1.039e+12
259.09	1.7891	1.5472	6.889e+11	1.233e+12	1.066e+12
262.12	1.7710	1.5320	7.138e+11	1.264e+12	1.093e+12
265.15	1.7534	1.5175	7.390e+11	1.296e+12	1.121e+12
268.18	1.7365	1.5036	7.646e+11	1.328e+12	1.150e+12
271.21	1.7201	1.4902	7.906e+11	1.360e+12	1.178e+12
274.24	1.7043	1.4774	8.170e+11	1.392e+12	1.207e+12
277.27	1.6890	1.4650	8.438e+11	1.425e+12	1.236e+12
280.30	1.6742	1.4531	8.710e+11	1.458e+12	1.266e+12
283.33	1.6598	1.4417	8.985e+11	1.491e+12	1.295e+12
286.36	1.6459	1.4306	9.264e+11	1.525e+12	1.325e+12
289.39	1.6325	1.4200	9.546e+11	1.558e+12	1.356e+12
292.42	1.6194	1.4098	9.832e+11	1.592e+12	1.386e+12
295.45	1.6068	1.3999	1.012e+12	1.626e+12	1.417e+12
298.48	1.5945	1.3903	1.041e+12	1.661e+12	1.448e+12
301.52	1.5827	1.3811	1.071e+12	1.695e+12	1.479e+12
304.55	1.5711	1.3722	1.101e+12	1.730e+12	1.511e+12
307.58	1.5599	1.3636	1.131e+12	1.765e+12	1.543e+12
310.61	1.5490	1.3553	1.162e+12	1.800e+12	1.575e+12
313.64	1.5385	1.3473	1.193e+12	1.835e+12	1.607e+12
316.67	1.5282	1.3395	1.224e+12	1.871e+12	1.640e+12
319.70	1.5183	1.3320	1.256e+12	1.907e+12	1.673e+12
322.73	1.5086	1.3247	1.288e+12	1.943e+12	1.706e+12
325.76	1.4992	1.3177	1.320e+12	1.979e+12	1.739e+12
328.79	1.4900	1.3108	1.353e+12	2.015e+12	1.773e+12
331.82	1.4811	1.3042	1.385e+12	2.052e+12	1.807e+12
334.85	1.4724	1.2978	1.419e+12	2.089e+12	1.841e+12
337.88	1.4640	1.2915	1.452e+12	2.126e+12	1.875e+12
340.91	1.4558	1.2855	1.486e+12	2.163e+12	1.910e+12

343.94	1.4478	1.2796	1.520e+12	2.200e+12	1.945e+12
346.97	1.4400	1.2739	1.554e+12	2.238e+12	1.980e+12
350.00	1.4324	1.2684	1.589e+12	2.276e+12	2.015e+12
353.03	1.4250	1.2630	1.623e+12	2.313e+12	2.050e+12
356.06	1.4178	1.2578	1.659e+12	2.352e+12	2.086e+12
359.09	1.4108	1.2527	1.694e+12	2.390e+12	2.122e+12
362.12	1.4039	1.2478	1.730e+12	2.428e+12	2.158e+12
365.15	1.3973	1.2430	1.766e+12	2.467e+12	2.195e+12
368.18	1.3908	1.2383	1.802e+12	2.506e+12	2.231e+12
371.21	1.3844	1.2337	1.838e+12	2.545e+12	2.268e+12
374.24	1.3782	1.2293	1.875e+12	2.584e+12	2.305e+12
377.27	1.3722	1.2250	1.912e+12	2.623e+12	2.342e+12
380.30	1.3662	1.2208	1.949e+12	2.663e+12	2.379e+12
383.33	1.3605	1.2167	1.986e+12	2.703e+12	2.417e+12
386.36	1.3548	1.2127	2.024e+12	2.742e+12	2.455e+12
389.39	1.3493	1.2089	2.062e+12	2.782e+12	2.493e+12
392.42	1.3440	1.2051	2.100e+12	2.823e+12	2.531e+12
395.45	1.3387	1.2014	2.139e+12	2.863e+12	2.569e+12
398.48	1.3336	1.1978	2.177e+12	2.903e+12	2.608e+12
401.52	1.3286	1.1943	2.216e+12	2.944e+12	2.647e+12
404.55	1.3237	1.1909	2.255e+12	2.985e+12	2.685e+12
407.58	1.3189	1.1875	2.294e+12	3.026e+12	2.725e+12
410.61	1.3142	1.1843	2.334e+12	3.067e+12	2.764e+12
413.64	1.3096	1.1811	2.374e+12	3.108e+12	2.803e+12
416.67	1.3051	1.1780	2.413e+12	3.150e+12	2.843e+12
419.70	1.3007	1.1749	2.454e+12	3.191e+12	2.883e+12
422.73	1.2964	1.1720	2.494e+12	3.233e+12	2.923e+12
425.76	1.2922	1.1691	2.534e+12	3.275e+12	2.963e+12
428.79	1.2881	1.1663	2.575e+12	3.317e+12	3.003e+12
431.82	1.2841	1.1635	2.616e+12	3.359e+12	3.044e+12
434.85	1.2801	1.1608	2.657e+12	3.402e+12	3.084e+12
437.88	1.2763	1.1581	2.699e+12	3.444e+12	3.125e+12
440.91	1.2725	1.1556	2.740e+12	3.487e+12	3.166e+12
443.94	1.2688	1.1530	2.782e+12	3.529e+12	3.208e+12
446.97	1.2651	1.1506	2.824e+12	3.572e+12	3.249e+12
450.00	1.2616	1.1481	2.866e+12	3.615e+12	3.290e+12

Pathway: EQ17-TS103-EQ7
Reactant Energy: 118.16 kcal/mol
TS Energy: 119.73 kcal/mol
Product Energy: -4.91 kcal/mol
Barrier Height: 1.57 kcal/mol
Imaginary Frequency: 383.34 cm^-1

Temperature(K)	Wigner_Factor	Eckart_Factor	TST Rate(s^{-1})	Wigner Rate (s^{-1})	Eckart Rate (s^{-1})
150.00	1.5633	1.6491	1.612e+10	2.521e+10	2.659e+10
153.03	1.5412	1.6160	1.826e+10	2.814e+10	2.951e+10
156.06	1.5204	1.5854	2.058e+10	3.130e+10	3.263e+10

159.09	1.5008	1.5572	2.311e+10	3.468e+10	3.598e+10
162.12	1.4822	1.5310	2.584e+10	3.830e+10	3.956e+10
165.15	1.4647	1.5067	2.878e+10	4.216e+10	4.337e+10
168.18	1.4481	1.4841	3.195e+10	4.627e+10	4.742e+10
171.21	1.4324	1.4630	3.534e+10	5.063e+10	5.171e+10
174.24	1.4175	1.4433	3.898e+10	5.525e+10	5.625e+10
177.27	1.4033	1.4249	4.285e+10	6.013e+10	6.106e+10
180.30	1.3899	1.4077	4.697e+10	6.528e+10	6.612e+10
183.33	1.3771	1.3915	5.135e+10	7.071e+10	7.145e+10
186.36	1.3649	1.3762	5.599e+10	7.642e+10	7.705e+10
189.39	1.3534	1.3619	6.089e+10	8.240e+10	8.293e+10
192.42	1.3423	1.3484	6.606e+10	8.868e+10	8.908e+10
195.45	1.3318	1.3357	7.151e+10	9.524e+10	9.552e+10
198.48	1.3217	1.3236	7.725e+10	1.021e+11	1.022e+11
201.52	1.3121	1.3122	8.326e+10	1.093e+11	1.093e+11
204.55	1.3029	1.3014	8.957e+10	1.167e+11	1.166e+11
207.58	1.2942	1.2912	9.617e+10	1.245e+11	1.242e+11
210.61	1.2858	1.2815	1.031e+11	1.325e+11	1.321e+11
213.64	1.2777	1.2723	1.103e+11	1.409e+11	1.403e+11
216.67	1.2700	1.2635	1.178e+11	1.496e+11	1.488e+11
219.70	1.2626	1.2552	1.256e+11	1.585e+11	1.576e+11
222.73	1.2555	1.2472	1.337e+11	1.678e+11	1.667e+11
225.76	1.2487	1.2396	1.421e+11	1.775e+11	1.762e+11
228.79	1.2421	1.2324	1.509e+11	1.874e+11	1.859e+11
231.82	1.2359	1.2255	1.599e+11	1.976e+11	1.960e+11
234.85	1.2298	1.2189	1.693e+11	2.082e+11	2.063e+11
237.88	1.2240	1.2126	1.790e+11	2.191e+11	2.170e+11
240.91	1.2184	1.2065	1.890e+11	2.303e+11	2.280e+11
243.94	1.2130	1.2008	1.993e+11	2.418e+11	2.393e+11
246.97	1.2078	1.1952	2.100e+11	2.536e+11	2.510e+11
250.00	1.2028	1.1899	2.210e+11	2.658e+11	2.629e+11
253.03	1.1980	1.1848	2.323e+11	2.782e+11	2.752e+11
256.06	1.1933	1.1799	2.439e+11	2.910e+11	2.878e+11
259.09	1.1888	1.1752	2.558e+11	3.041e+11	3.007e+11
262.12	1.1845	1.1706	2.681e+11	3.176e+11	3.139e+11
265.15	1.1803	1.1663	2.807e+11	3.313e+11	3.274e+11
268.18	1.1762	1.1621	2.937e+11	3.454e+11	3.413e+11
271.21	1.1723	1.1581	3.069e+11	3.598e+11	3.554e+11
274.24	1.1685	1.1542	3.205e+11	3.745e+11	3.699e+11
277.27	1.1649	1.1504	3.344e+11	3.895e+11	3.847e+11
280.30	1.1613	1.1468	3.486e+11	4.049e+11	3.998e+11
283.33	1.1579	1.1433	3.632e+11	4.205e+11	4.152e+11
286.36	1.1546	1.1400	3.781e+11	4.365e+11	4.310e+11
289.39	1.1513	1.1367	3.933e+11	4.528e+11	4.470e+11
292.42	1.1482	1.1336	4.088e+11	4.694e+11	4.634e+11
295.45	1.1452	1.1306	4.246e+11	4.863e+11	4.801e+11
298.48	1.1423	1.1276	4.408e+11	5.035e+11	4.970e+11
301.52	1.1394	1.1248	4.573e+11	5.210e+11	5.143e+11

304.55	1.1367	1.1220	4.741e+11	5.388e+11	5.319e+11
307.58	1.1340	1.1194	4.912e+11	5.570e+11	5.498e+11
310.61	1.1314	1.1168	5.086e+11	5.754e+11	5.680e+11
313.64	1.1289	1.1143	5.263e+11	5.942e+11	5.865e+11
316.67	1.1264	1.1119	5.444e+11	6.132e+11	6.053e+11
319.70	1.1240	1.1096	5.627e+11	6.325e+11	6.244e+11
322.73	1.1217	1.1073	5.814e+11	6.522e+11	6.438e+11
325.76	1.1194	1.1051	6.004e+11	6.721e+11	6.635e+11
328.79	1.1172	1.1030	6.197e+11	6.923e+11	6.835e+11
331.82	1.1151	1.1009	6.393e+11	7.129e+11	7.038e+11
334.85	1.1130	1.0989	6.591e+11	7.337e+11	7.243e+11
337.88	1.1110	1.0970	6.793e+11	7.548e+11	7.452e+11
340.91	1.1091	1.0951	6.998e+11	7.762e+11	7.664e+11
343.94	1.1071	1.0932	7.206e+11	7.978e+11	7.878e+11
346.97	1.1053	1.0914	7.417e+11	8.198e+11	8.095e+11
350.00	1.1035	1.0897	7.631e+11	8.420e+11	8.315e+11
353.03	1.1017	1.0880	7.847e+11	8.645e+11	8.538e+11
356.06	1.1000	1.0863	8.067e+11	8.873e+11	8.763e+11
359.09	1.0983	1.0847	8.289e+11	9.104e+11	8.992e+11
362.12	1.0967	1.0832	8.515e+11	9.338e+11	9.223e+11
365.15	1.0951	1.0817	8.743e+11	9.574e+11	9.457e+11
368.18	1.0935	1.0802	8.974e+11	9.813e+11	9.693e+11
371.21	1.0920	1.0787	9.207e+11	1.005e+12	9.932e+11
374.24	1.0905	1.0773	9.444e+11	1.030e+12	1.017e+12
377.27	1.0890	1.0760	9.683e+11	1.055e+12	1.042e+12
380.30	1.0876	1.0746	9.925e+11	1.079e+12	1.067e+12
383.33	1.0863	1.0733	1.017e+12	1.105e+12	1.092e+12
386.36	1.0849	1.0721	1.042e+12	1.130e+12	1.117e+12
389.39	1.0836	1.0708	1.067e+12	1.156e+12	1.142e+12
392.42	1.0823	1.0696	1.092e+12	1.182e+12	1.168e+12
395.45	1.0810	1.0685	1.118e+12	1.208e+12	1.194e+12
398.48	1.0798	1.0673	1.143e+12	1.235e+12	1.220e+12
401.52	1.0786	1.0662	1.169e+12	1.261e+12	1.247e+12
404.55	1.0774	1.0651	1.196e+12	1.288e+12	1.274e+12
407.58	1.0763	1.0640	1.222e+12	1.316e+12	1.301e+12
410.61	1.0752	1.0630	1.249e+12	1.343e+12	1.328e+12
413.64	1.0741	1.0620	1.276e+12	1.371e+12	1.355e+12
416.67	1.0730	1.0610	1.304e+12	1.399e+12	1.383e+12
419.70	1.0720	1.0600	1.331e+12	1.427e+12	1.411e+12
422.73	1.0709	1.0591	1.359e+12	1.455e+12	1.439e+12
425.76	1.0699	1.0581	1.387e+12	1.484e+12	1.468e+12
428.79	1.0689	1.0572	1.415e+12	1.513e+12	1.496e+12
431.82	1.0680	1.0564	1.444e+12	1.542e+12	1.525e+12
434.85	1.0670	1.0555	1.473e+12	1.571e+12	1.554e+12
437.88	1.0661	1.0546	1.502e+12	1.601e+12	1.584e+12
440.91	1.0652	1.0538	1.531e+12	1.631e+12	1.613e+12
443.94	1.0643	1.0530	1.560e+12	1.661e+12	1.643e+12
446.97	1.0634	1.0522	1.590e+12	1.691e+12	1.673e+12

450.00	1.0626	1.0514	1.620e+12	1.722e+12	1.703e+12

Pathway: EQ1-TS2-DC
Reactant Energy: 21.46 kcal/mol
TS Energy: 27.77 kcal/mol
Product Energy: None kcal/mol
Barrier Height: 6.31 kcal/mol
Imaginary Frequency: 565.83 cm^-1

Temperature(K)	Wigner_Factor	Eckart_Factor	TST Rate(s^{-1})	Wigner Rate (s^{-1})	Eckart Rate (s^{-1})
150.00	2.2273	4.4246	2.002e+03	4.459e+03	8.858e+03
153.03	2.1792	4.1287	3.106e+03	6.768e+03	1.282e+04
156.06	2.1339	3.8722	4.739e+03	1.011e+04	1.835e+04
159.09	2.0911	3.6484	7.118e+03	1.488e+04	2.597e+04
162.12	2.0507	3.4518	1.053e+04	2.160e+04	3.636e+04
165.15	2.0125	3.2781	1.537e+04	3.093e+04	5.038e+04
168.18	1.9763	3.1237	2.213e+04	4.374e+04	6.913e+04
171.21	1.9421	2.9858	3.147e+04	6.112e+04	9.396e+04
174.24	1.9096	2.8621	4.422e+04	8.444e+04	1.266e+05
177.27	1.8787	2.7506	6.143e+04	1.154e+05	1.690e+05
180.30	1.8495	2.6497	8.443e+04	1.561e+05	2.237e+05
183.33	1.8216	2.5580	1.149e+05	2.092e+05	2.938e+05
186.36	1.7951	2.4745	1.547e+05	2.777e+05	3.829e+05
189.39	1.7699	2.3981	2.065e+05	3.655e+05	4.953e+05
192.42	1.7458	2.3281	2.732e+05	4.770e+05	6.361e+05
195.45	1.7229	2.2637	3.584e+05	6.175e+05	8.114e+05
198.48	1.7010	2.2042	4.665e+05	7.934e+05	1.028e+06
201.52	1.6800	2.1493	6.024e+05	1.012e+06	1.295e+06
204.55	1.6600	2.0984	7.722e+05	1.282e+06	1.620e+06
207.58	1.6409	2.0510	9.830e+05	1.613e+06	2.016e+06
210.61	1.6226	2.0070	1.243e+06	2.017e+06	2.494e+06
213.64	1.6051	1.9659	1.561e+06	2.506e+06	3.070e+06
216.67	1.5883	1.9276	1.949e+06	3.096e+06	3.758e+06
219.70	1.5721	1.8916	2.419e+06	3.804e+06	4.577e+06
222.73	1.5567	1.8579	2.986e+06	4.648e+06	5.548e+06
225.76	1.5418	1.8263	3.665e+06	5.651e+06	6.693e+06
228.79	1.5276	1.7965	4.475e+06	6.835e+06	8.038e+06
231.82	1.5139	1.7684	5.436e+06	8.229e+06	9.613e+06
234.85	1.5007	1.7420	6.571e+06	9.861e+06	1.145e+07
237.88	1.4880	1.7170	7.907e+06	1.177e+07	1.358e+07
240.91	1.4758	1.6933	9.472e+06	1.398e+07	1.604e+07
243.94	1.4641	1.6709	1.130e+07	1.654e+07	1.888e+07
246.97	1.4528	1.6497	1.342e+07	1.949e+07	2.214e+07
250.00	1.4418	1.6295	1.587e+07	2.289e+07	2.587e+07
253.03	1.4313	1.6104	1.871e+07	2.677e+07	3.012e+07
256.06	1.4212	1.5922	2.196e+07	3.121e+07	3.496e+07
259.09	1.4114	1.5748	2.569e+07	3.626e+07	4.045e+07
262.12	1.4019	1.5583	2.994e+07	4.198e+07	4.666e+07

265.15	1.3928	1.5425	3.479e+07	4.845e+07	5.366e+07
268.18	1.3840	1.5275	4.028e+07	5.575e+07	6.153e+07
271.21	1.3754	1.5131	4.650e+07	6.396e+07	7.036e+07
274.24	1.3672	1.4993	5.352e+07	7.317e+07	8.024e+07
277.27	1.3592	1.4862	6.141e+07	8.346e+07	9.126e+07
280.30	1.3515	1.4735	7.026e+07	9.495e+07	1.035e+08
283.33	1.3440	1.4615	8.017e+07	1.077e+08	1.172e+08
286.36	1.3368	1.4499	9.122e+07	1.219e+08	1.323e+08
289.39	1.3297	1.4387	1.035e+08	1.377e+08	1.490e+08
292.42	1.3229	1.4281	1.172e+08	1.551e+08	1.674e+08
295.45	1.3163	1.4178	1.324e+08	1.743e+08	1.877e+08
298.48	1.3100	1.4079	1.492e+08	1.954e+08	2.100e+08
301.52	1.3038	1.3984	1.677e+08	2.186e+08	2.345e+08
304.55	1.2977	1.3893	1.881e+08	2.441e+08	2.613e+08
307.58	1.2919	1.3804	2.105e+08	2.720e+08	2.906e+08
310.61	1.2862	1.3720	2.351e+08	3.024e+08	3.226e+08
313.64	1.2807	1.3638	2.620e+08	3.356e+08	3.574e+08
316.67	1.2754	1.3559	2.915e+08	3.718e+08	3.952e+08
319.70	1.2702	1.3482	3.236e+08	4.111e+08	4.363e+08
322.73	1.2651	1.3409	3.586e+08	4.537e+08	4.809e+08
325.76	1.2602	1.3337	3.967e+08	4.999e+08	5.291e+08
328.79	1.2555	1.3269	4.380e+08	5.499e+08	5.812e+08
331.82	1.2508	1.3202	4.828e+08	6.039e+08	6.374e+08
334.85	1.2463	1.3138	5.313e+08	6.621e+08	6.980e+08
337.88	1.2419	1.3075	5.837e+08	7.249e+08	7.632e+08
340.91	1.2376	1.3015	6.402e+08	7.924e+08	8.333e+08
343.94	1.2334	1.2957	7.012e+08	8.648e+08	9.085e+08
346.97	1.2294	1.2900	7.667e+08	9.426e+08	9.891e+08
350.00	1.2254	1.2845	8.372e+08	1.026e+09	1.075e+09
353.03	1.2216	1.2792	9.128e+08	1.115e+09	1.168e+09
356.06	1.2178	1.2740	9.939e+08	1.210e+09	1.266e+09
359.09	1.2142	1.2690	1.081e+09	1.312e+09	1.371e+09
362.12	1.2106	1.2641	1.174e+09	1.421e+09	1.484e+09
365.15	1.2071	1.2594	1.273e+09	1.536e+09	1.603e+09
368.18	1.2037	1.2548	1.378e+09	1.659e+09	1.730e+09
371.21	1.2004	1.2503	1.491e+09	1.790e+09	1.865e+09
374.24	1.1972	1.2460	1.611e+09	1.929e+09	2.008e+09
377.27	1.1940	1.2418	1.739e+09	2.076e+09	2.159e+09
380.30	1.1909	1.2377	1.874e+09	2.232e+09	2.320e+09
383.33	1.1879	1.2337	2.018e+09	2.397e+09	2.490e+09
386.36	1.1850	1.2298	2.171e+09	2.572e+09	2.669e+09
389.39	1.1821	1.2260	2.332e+09	2.757e+09	2.859e+09
392.42	1.1793	1.2223	2.503e+09	2.952e+09	3.060e+09
395.45	1.1766	1.2187	2.684e+09	3.158e+09	3.271e+09
398.48	1.1739	1.2153	2.875e+09	3.375e+09	3.493e+09
401.52	1.1713	1.2118	3.076e+09	3.603e+09	3.728e+09
404.55	1.1687	1.2085	3.288e+09	3.843e+09	3.974e+09
407.58	1.1662	1.2053	3.512e+09	4.096e+09	4.233e+09

410.61	1.1638	1.2021	3.748e+09	4.361e+09	4.505e+09
413.64	1.1614	1.1990	3.995e+09	4.640e+09	4.791e+09
416.67	1.1591	1.1960	4.256e+09	4.933e+09	5.090e+09
419.70	1.1568	1.1931	4.529e+09	5.239e+09	5.404e+09
422.73	1.1545	1.1902	4.816e+09	5.560e+09	5.732e+09
425.76	1.1523	1.1874	5.117e+09	5.896e+09	6.076e+09
428.79	1.1502	1.1847	5.432e+09	6.248e+09	6.436e+09
431.82	1.1481	1.1820	5.762e+09	6.616e+09	6.811e+09
434.85	1.1460	1.1794	6.108e+09	7.000e+09	7.204e+09
437.88	1.1440	1.1769	6.469e+09	7.401e+09	7.614e+09
440.91	1.1421	1.1744	6.847e+09	7.820e+09	8.041e+09
443.94	1.1401	1.1720	7.241e+09	8.256e+09	8.487e+09
446.97	1.1382	1.1696	7.653e+09	8.711e+09	8.951e+09
450.00	1.1364	1.1673	8.083e+09	9.185e+09	9.434e+09

Pathway: EQ0-TS71-DC
Reactant Energy: 0.0 kcal/mol
TS Energy: 36.09 kcal/mol
Product Energy: None kcal/mol
Barrier Height: 36.09 kcal/mol
Imaginary Frequency: 673.89 cm^-1

Temperature(K)	Wigner_Factor	Eckart_Factor	TST Rate(s^{-1})	Wigner Rate (s^{-1})	Eckart Rate (s^{-1})
150.00	2.7409	27.3535	8.182e-41	2.243e-40	2.238e-39
153.03	2.6726	20.6746	9.179e-40	2.453e-39	1.898e-38
156.06	2.6083	16.2774	9.378e-39	2.446e-38	1.526e-37
159.09	2.5476	13.2468	8.772e-38	2.235e-37	1.162e-36
162.12	2.4903	11.0752	7.551e-37	1.880e-36	8.363e-36
165.15	2.4361	9.4669	6.008e-36	1.464e-35	5.687e-35
168.18	2.3848	8.2415	4.437e-35	1.058e-34	3.657e-34
171.21	2.3362	7.2851	3.054e-34	7.136e-34	2.225e-33
174.24	2.2902	6.5229	1.967e-33	4.504e-33	1.283e-32
177.27	2.2464	5.9045	1.188e-32	2.670e-32	7.017e-32
180.30	2.2049	5.3947	6.763e-32	1.491e-31	3.648e-31
183.33	2.1654	4.9688	3.634e-31	7.869e-31	1.806e-30
186.36	2.1278	4.6086	1.850e-30	3.935e-30	8.524e-30
189.39	2.0920	4.3007	8.938e-30	1.870e-29	3.844e-29
192.42	2.0579	4.0351	4.111e-29	8.460e-29	1.659e-28
195.45	2.0253	3.8039	1.804e-28	3.654e-28	6.862e-28
198.48	1.9943	3.6012	7.568e-28	1.509e-27	2.725e-27
201.52	1.9646	3.4222	3.042e-27	5.976e-27	1.041e-26
204.55	1.9362	3.2632	1.173e-26	2.272e-26	3.829e-26
207.58	1.9091	3.1212	4.353e-26	8.310e-26	1.359e-25
210.61	1.8831	2.9937	1.555e-25	2.929e-25	4.656e-25
213.64	1.8582	2.8786	5.360e-25	9.961e-25	1.543e-24
216.67	1.8344	2.7744	1.785e-24	3.275e-24	4.953e-24
219.70	1.8115	2.6796	5.752e-24	1.042e-23	1.541e-23
222.73	1.7896	2.5931	1.796e-23	3.213e-23	4.656e-23

225.76	1.7685	2.5138	5.438e-23	9.617e-23	1.367e-22
228.79	1.7483	2.4409	1.599e-22	2.796e-22	3.904e-22
231.82	1.7289	2.3738	4.574e-22	7.908e-22	1.086e-21
234.85	1.7102	2.3117	1.273e-21	2.178e-21	2.944e-21
237.88	1.6922	2.2542	3.454e-21	5.845e-21	7.787e-21
240.91	1.6749	2.2009	9.139e-21	1.531e-20	2.011e-20
243.94	1.6582	2.1512	2.361e-20	3.915e-20	5.078e-20
246.97	1.6422	2.1048	5.959e-20	9.785e-20	1.254e-19
250.00	1.6267	2.0615	1.471e-19	2.393e-19	3.032e-19
253.03	1.6118	2.0209	3.553e-19	5.727e-19	7.181e-19
256.06	1.5974	1.9828	8.408e-19	1.343e-18	1.667e-18
259.09	1.5835	1.9471	1.950e-18	3.088e-18	3.797e-18
262.12	1.5701	1.9134	4.436e-18	6.966e-18	8.489e-18
265.15	1.5571	1.8817	9.906e-18	1.543e-17	1.864e-17
268.18	1.5446	1.8518	2.173e-17	3.356e-17	4.023e-17
271.21	1.5325	1.8235	4.682e-17	7.176e-17	8.538e-17
274.24	1.5208	1.7967	9.922e-17	1.509e-16	1.783e-16
277.27	1.5095	1.7713	2.069e-16	3.123e-16	3.664e-16
280.30	1.4985	1.7472	4.246e-16	6.362e-16	7.418e-16
283.33	1.4879	1.7244	8.582e-16	1.277e-15	1.480e-15
286.36	1.4777	1.7026	1.709e-15	2.525e-15	2.910e-15
289.39	1.4677	1.6819	3.355e-15	4.925e-15	5.644e-15
292.42	1.4581	1.6622	6.497e-15	9.473e-15	1.080e-14
295.45	1.4487	1.6434	1.241e-14	1.798e-14	2.040e-14
298.48	1.4397	1.6255	2.340e-14	3.369e-14	3.804e-14
301.52	1.4309	1.6083	4.357e-14	6.235e-14	7.008e-14
304.55	1.4223	1.5920	8.014e-14	1.140e-13	1.276e-13
307.58	1.4140	1.5763	1.457e-13	2.060e-13	2.296e-13
310.61	1.4060	1.5612	2.617e-13	3.679e-13	4.085e-13
313.64	1.3982	1.5469	4.649e-13	6.500e-13	7.191e-13
316.67	1.3906	1.5330	8.169e-13	1.136e-12	1.252e-12
319.70	1.3832	1.5198	1.420e-12	1.965e-12	2.159e-12
322.73	1.3761	1.5071	2.444e-12	3.363e-12	3.683e-12
325.76	1.3691	1.4948	4.164e-12	5.701e-12	6.225e-12
328.79	1.3623	1.4831	7.026e-12	9.572e-12	1.042e-11
331.82	1.3558	1.4718	1.174e-11	1.592e-11	1.728e-11
334.85	1.3493	1.4609	1.945e-11	2.624e-11	2.841e-11
337.88	1.3431	1.4504	3.191e-11	4.287e-11	4.629e-11
340.91	1.3370	1.4403	5.192e-11	6.942e-11	7.478e-11
343.94	1.3311	1.4305	8.376e-11	1.115e-10	1.198e-10
346.97	1.3254	1.4211	1.340e-10	1.776e-10	1.904e-10
350.00	1.3198	1.4120	2.127e-10	2.807e-10	3.003e-10
353.03	1.3143	1.4032	3.349e-10	4.402e-10	4.699e-10
356.06	1.3090	1.3948	5.233e-10	6.850e-10	7.299e-10
359.09	1.3038	1.3866	8.117e-10	1.058e-09	1.125e-09
362.12	1.2987	1.3786	1.250e-09	1.623e-09	1.723e-09
365.15	1.2938	1.3710	1.911e-09	2.472e-09	2.620e-09
368.18	1.2890	1.3635	2.901e-09	3.740e-09	3.956e-09

371.21	1.2843	1.3563	4.375e-09	5.619e-09	5.934e-09
374.24	1.2797	1.3494	6.555e-09	8.389e-09	8.845e-09
377.27	1.2752	1.3426	9.758e-09	1.244e-08	1.310e-08
380.30	1.2708	1.3361	1.444e-08	1.834e-08	1.929e-08
383.33	1.2666	1.3297	2.122e-08	2.688e-08	2.822e-08
386.36	1.2624	1.3236	3.102e-08	3.916e-08	4.105e-08
389.39	1.2583	1.3176	4.507e-08	5.671e-08	5.938e-08
392.42	1.2544	1.3118	6.511e-08	8.167e-08	8.541e-08
395.45	1.2505	1.3061	9.354e-08	1.170e-07	1.222e-07
398.48	1.2467	1.3006	1.337e-07	1.666e-07	1.738e-07
401.52	1.2430	1.2953	1.900e-07	2.361e-07	2.461e-07
404.55	1.2393	1.2901	2.686e-07	3.329e-07	3.465e-07
407.58	1.2358	1.2851	3.778e-07	4.669e-07	4.855e-07
410.61	1.2323	1.2802	5.288e-07	6.517e-07	6.770e-07
413.64	1.2289	1.2754	7.366e-07	9.052e-07	9.395e-07
416.67	1.2256	1.2708	1.021e-06	1.252e-06	1.298e-06
419.70	1.2224	1.2663	1.409e-06	1.722e-06	1.784e-06
422.73	1.2192	1.2619	1.935e-06	2.359e-06	2.442e-06
425.76	1.2161	1.2576	2.646e-06	3.218e-06	3.328e-06
428.79	1.2130	1.2534	3.603e-06	4.370e-06	4.516e-06
431.82	1.2101	1.2493	4.884e-06	5.910e-06	6.102e-06
434.85	1.2071	1.2454	6.593e-06	7.959e-06	8.211e-06
437.88	1.2043	1.2415	8.864e-06	1.068e-05	1.101e-05
440.91	1.2015	1.2377	1.187e-05	1.426e-05	1.469e-05
443.94	1.1987	1.2341	1.583e-05	1.898e-05	1.954e-05
446.97	1.1961	1.2305	2.103e-05	2.516e-05	2.588e-05
450.00	1.1934	1.2270	2.784e-05	3.323e-05	3.416e-05

Pathway: EQ4-TS63-DC
Reactant Energy: 58.21 kcal/mol
TS Energy: 139.28 kcal/mol
Product Energy: None kcal/mol
Barrier Height: 81.07 kcal/mol
Imaginary Frequency: 649.19 cm^-1

Temperature(K)	Wigner_Factor	Eckart_Factor	TST Rate(s^{-1})	Wigner Rate (s^{-1})	Eckart Rate (s^{-1})
150.00	2.6156	24.3507	2.390e-106	6.251e-106	5.820e-105
153.03	2.5523	17.8460	5.321e-104	1.358e-103	9.496e-103
156.06	2.4926	13.8525	9.608e-102	2.395e-101	1.331e-100
159.09	2.4362	11.2226	1.424e-99	3.469e-99	1.598e-98
162.12	2.3831	9.3918	1.751e-97	4.173e-97	1.644e-96
165.15	2.3328	8.0597	1.805e-95	4.211e-95	1.455e-94
168.18	2.2852	7.0552	1.576e-93	3.600e-93	1.112e-92
171.21	2.2401	6.2752	1.174e-91	2.630e-91	7.368e-91
174.24	2.1973	5.6548	7.534e-90	1.656e-89	4.261e-89
177.27	2.1567	5.1513	4.195e-88	9.047e-88	2.161e-87
180.30	2.1182	4.7355	2.041e-86	4.323e-86	9.664e-86
183.33	2.0815	4.3872	8.735e-85	1.818e-84	3.832e-84

186.36	2.0466	4.0917	3.310e-83	6.774e-83	1.354e-82
189.39	2.0134	3.8382	1.117e-81	2.248e-81	4.286e-81
192.42	1.9817	3.6187	3.373e-80	6.684e-80	1.221e-79
195.45	1.9515	3.4270	9.168e-79	1.789e-78	3.142e-78
198.48	1.9227	3.2582	2.254e-77	4.333e-77	7.343e-77
201.52	1.8952	3.1087	5.033e-76	9.538e-76	1.564e-75
204.55	1.8688	2.9753	1.025e-74	1.916e-74	3.050e-74
207.58	1.8437	2.8558	1.913e-73	3.527e-73	5.463e-73
210.61	1.8196	2.7482	3.282e-72	5.972e-72	9.019e-72
213.64	1.7965	2.6508	5.195e-71	9.333e-71	1.377e-70
216.67	1.7743	2.5622	7.614e-70	1.351e-69	1.951e-69
219.70	1.7531	2.4815	1.036e-68	1.817e-68	2.572e-68
222.73	1.7328	2.4075	1.314e-67	2.278e-67	3.164e-67
225.76	1.7132	2.3396	1.557e-66	2.668e-66	3.643e-66
228.79	1.6945	2.2771	1.728e-65	2.929e-65	3.935e-65
231.82	1.6764	2.2193	1.801e-64	3.020e-64	3.998e-64
234.85	1.6591	2.1658	1.768e-63	2.933e-63	3.829e-63
237.88	1.6424	2.1161	1.637e-62	2.689e-62	3.464e-62
240.91	1.6263	2.0698	1.434e-61	2.331e-61	2.967e-61
243.94	1.6109	2.0267	1.190e-60	1.916e-60	2.411e-60
246.97	1.5960	1.9863	9.375e-60	1.496e-59	1.862e-59
250.00	1.5816	1.9486	7.028e-59	1.112e-58	1.370e-58
253.03	1.5678	1.9132	5.021e-58	7.872e-58	9.607e-58
256.06	1.5544	1.8799	3.425e-57	5.324e-57	6.438e-57
259.09	1.5415	1.8486	2.234e-56	3.443e-56	4.129e-56
262.12	1.5291	1.8191	1.395e-55	2.133e-55	2.538e-55
265.15	1.5170	1.7913	8.358e-55	1.268e-54	1.497e-54
268.18	1.5054	1.7649	4.809e-54	7.240e-54	8.488e-54
271.21	1.4942	1.7400	2.661e-53	3.977e-53	4.631e-53
274.24	1.4833	1.7164	1.418e-52	2.104e-52	2.434e-52
277.27	1.4728	1.6940	7.288e-52	1.073e-51	1.235e-51
280.30	1.4627	1.6727	3.615e-51	5.288e-51	6.047e-51
283.33	1.4528	1.6525	1.733e-50	2.518e-50	2.864e-50
286.36	1.4433	1.6332	8.039e-50	1.160e-49	1.313e-49
289.39	1.4341	1.6149	3.611e-49	5.178e-49	5.831e-49
292.42	1.4251	1.5974	1.572e-48	2.241e-48	2.512e-48
295.45	1.4164	1.5807	6.644e-48	9.411e-48	1.050e-47
298.48	1.4080	1.5648	2.727e-47	3.840e-47	4.267e-47
301.52	1.3999	1.5495	1.088e-46	1.523e-46	1.686e-46
304.55	1.3919	1.5349	4.224e-46	5.879e-46	6.483e-46
307.58	1.3843	1.5209	1.596e-45	2.210e-45	2.428e-45
310.61	1.3768	1.5075	5.880e-45	8.096e-45	8.865e-45
313.64	1.3695	1.4947	2.112e-44	2.893e-44	3.157e-44
316.67	1.3625	1.4823	7.404e-44	1.009e-43	1.098e-43
319.70	1.3557	1.4705	2.535e-43	3.436e-43	3.728e-43
322.73	1.3490	1.4591	8.481e-43	1.144e-42	1.237e-42
325.76	1.3426	1.4482	2.774e-42	3.725e-42	4.018e-42
328.79	1.3363	1.4376	8.881e-42	1.187e-41	1.277e-41

331.82	1.3302	1.4275	2.783e-41	3.702e-41	3.973e-41
334.85	1.3242	1.4177	8.546e-41	1.132e-40	1.212e-40
337.88	1.3184	1.4083	2.572e-40	3.390e-40	3.622e-40
340.91	1.3128	1.3992	7.589e-40	9.963e-40	1.062e-39
343.94	1.3073	1.3905	2.197e-39	2.873e-39	3.056e-39
346.97	1.3020	1.3820	6.246e-39	8.133e-39	8.633e-39
350.00	1.2967	1.3738	1.744e-38	2.261e-38	2.396e-38
353.03	1.2917	1.3659	4.784e-38	6.179e-38	6.534e-38
356.06	1.2867	1.3583	1.290e-37	1.660e-37	1.752e-37
359.09	1.2819	1.3509	3.421e-37	4.386e-37	4.622e-37
362.12	1.2772	1.3438	8.928e-37	1.140e-36	1.200e-36
365.15	1.2726	1.3369	2.293e-36	2.918e-36	3.065e-36
368.18	1.2682	1.3302	5.799e-36	7.354e-36	7.713e-36
371.21	1.2638	1.3237	1.445e-35	1.826e-35	1.912e-35
374.24	1.2595	1.3174	3.546e-35	4.466e-35	4.671e-35
377.27	1.2554	1.3113	8.580e-35	1.077e-34	1.125e-34
380.30	1.2513	1.3054	2.047e-34	2.562e-34	2.672e-34
383.33	1.2474	1.2996	4.818e-34	6.010e-34	6.262e-34
386.36	1.2435	1.2941	1.119e-33	1.391e-33	1.448e-33
389.39	1.2397	1.2887	2.565e-33	3.180e-33	3.305e-33
392.42	1.2361	1.2834	5.805e-33	7.175e-33	7.450e-33
395.45	1.2324	1.2783	1.297e-32	1.599e-32	1.659e-32
398.48	1.2289	1.2733	2.865e-32	3.521e-32	3.648e-32
401.52	1.2255	1.2685	6.251e-32	7.661e-32	7.930e-32
404.55	1.2221	1.2638	1.348e-31	1.648e-31	1.704e-31
407.58	1.2188	1.2593	2.875e-31	3.504e-31	3.620e-31
410.61	1.2156	1.2548	6.063e-31	7.370e-31	7.608e-31
413.64	1.2125	1.2505	1.265e-30	1.533e-30	1.581e-30
416.67	1.2094	1.2463	2.610e-30	3.157e-30	3.253e-30
419.70	1.2064	1.2422	5.331e-30	6.431e-30	6.623e-30
422.73	1.2034	1.2382	1.078e-29	1.297e-29	1.335e-29
425.76	1.2005	1.2343	2.158e-29	2.590e-29	2.663e-29
428.79	1.1977	1.2306	4.277e-29	5.123e-29	5.263e-29
431.82	1.1949	1.2269	8.398e-29	1.004e-28	1.030e-28
434.85	1.1922	1.2233	1.634e-28	1.948e-28	1.998e-28
437.88	1.1896	1.2197	3.149e-28	3.746e-28	3.840e-28
440.91	1.1870	1.2163	6.014e-28	7.139e-28	7.316e-28
443.94	1.1844	1.2130	1.139e-27	1.349e-27	1.381e-27
446.97	1.1820	1.2097	2.138e-27	2.527e-27	2.586e-27
450.00	1.1795	1.2065	3.980e-27	4.694e-27	4.802e-27

Pathway: EQ20-TS64-DC
Reactant Energy: 77.45 kcal/mol
TS Energy: 113.69 kcal/mol
Product Energy: None kcal/mol
Barrier Height: 36.24 kcal/mol
Imaginary Frequency: 661.85 cm^-1

Temperature(K)	Wigner_Factor	Eckart_Factor	TST Rate(s^{-1})	Wigner Rate (s^{-1})	Eckart Rate (s^{-1})
150.00	2.6792	21.4898	4.947e-41	1.325e-40	1.063e-39
153.03	2.6134	16.7419	5.605e-40	1.465e-39	9.384e-39
156.06	2.5513	13.5158	5.782e-39	1.475e-38	7.814e-38
159.09	2.4928	11.2306	5.458e-38	1.361e-37	6.130e-37
162.12	2.4375	9.5538	4.740e-37	1.155e-36	4.529e-36
165.15	2.3853	8.2861	3.804e-36	9.073e-36	3.152e-35
168.18	2.3358	7.3029	2.833e-35	6.616e-35	2.069e-34
171.21	2.2889	6.5235	1.965e-34	4.499e-34	1.282e-33
174.24	2.2445	5.8938	1.275e-33	2.862e-33	7.516e-33
177.27	2.2023	5.3768	7.763e-33	1.710e-32	4.174e-32
180.30	2.1622	4.9461	4.449e-32	9.620e-32	2.201e-31
183.33	2.1241	4.5829	2.408e-31	5.114e-31	1.103e-30
186.36	2.0879	4.2732	1.234e-30	2.575e-30	5.271e-30
189.39	2.0533	4.0065	6.000e-30	1.232e-29	2.404e-29
192.42	2.0204	3.7748	2.777e-29	5.611e-29	1.048e-28
195.45	1.9890	3.5720	1.226e-28	2.439e-28	4.379e-28
198.48	1.9590	3.3932	5.174e-28	1.014e-27	1.756e-27
201.52	1.9304	3.2346	2.091e-27	4.037e-27	6.765e-27
204.55	1.9031	3.0931	8.113e-27	1.544e-26	2.509e-26
207.58	1.8769	2.9661	3.026e-26	5.679e-26	8.975e-26
210.61	1.8518	2.8517	1.087e-25	2.012e-25	3.099e-25
213.64	1.8278	2.7481	3.765e-25	6.882e-25	1.035e-24
216.67	1.8048	2.6540	1.260e-24	2.274e-24	3.344e-24
219.70	1.7828	2.5681	4.079e-24	7.273e-24	1.048e-23
222.73	1.7616	2.4894	1.279e-23	2.254e-23	3.185e-23
225.76	1.7413	2.4172	3.892e-23	6.778e-23	9.409e-23
228.79	1.7218	2.3507	1.150e-22	1.980e-22	2.703e-22
231.82	1.7031	2.2893	3.303e-22	5.625e-22	7.561e-22
234.85	1.6850	2.2324	9.234e-22	1.556e-21	2.061e-21
237.88	1.6677	2.1796	2.515e-21	4.194e-21	5.482e-21
240.91	1.6510	2.1305	6.681e-21	1.103e-20	1.423e-20
243.94	1.6349	2.0847	1.732e-20	2.833e-20	3.612e-20
246.97	1.6195	2.0419	4.389e-20	7.108e-20	8.963e-20
250.00	1.6045	2.0018	1.087e-19	1.745e-19	2.177e-19
253.03	1.5901	1.9643	2.637e-19	4.193e-19	5.179e-19
256.06	1.5762	1.9290	6.261e-19	9.869e-19	1.208e-18
259.09	1.5628	1.8958	1.457e-18	2.277e-18	2.763e-18
262.12	1.5499	1.8645	3.326e-18	5.156e-18	6.202e-18
265.15	1.5374	1.8350	7.452e-18	1.146e-17	1.367e-17
268.18	1.5253	1.8071	1.640e-17	2.501e-17	2.963e-17
271.21	1.5137	1.7807	3.545e-17	5.365e-17	6.312e-17
274.24	1.5024	1.7557	7.535e-17	1.132e-16	1.323e-16
277.27	1.4915	1.7320	1.576e-16	2.350e-16	2.729e-16
280.30	1.4809	1.7095	3.243e-16	4.803e-16	5.545e-16
283.33	1.4707	1.6881	6.575e-16	9.669e-16	1.110e-15
286.36	1.4607	1.6677	1.313e-15	1.918e-15	2.190e-15
289.39	1.4511	1.6483	2.585e-15	3.751e-15	4.261e-15

292.42	1.4418	1.6298	5.019e-15	7.236e-15	8.180e-15
295.45	1.4328	1.6122	9.613e-15	1.377e-14	1.550e-14
298.48	1.4241	1.5953	1.817e-14	2.588e-14	2.899e-14
301.52	1.4156	1.5792	3.392e-14	4.802e-14	5.357e-14
304.55	1.4074	1.5638	6.255e-14	8.803e-14	9.781e-14
307.58	1.3994	1.5490	1.140e-13	1.595e-13	1.765e-13
310.61	1.3916	1.5349	2.052e-13	2.856e-13	3.150e-13
313.64	1.3841	1.5213	3.654e-13	5.058e-13	5.559e-13
316.67	1.3768	1.5083	6.436e-13	8.861e-13	9.708e-13
319.70	1.3697	1.4958	1.122e-12	1.536e-12	1.678e-12
322.73	1.3628	1.4838	1.934e-12	2.636e-12	2.870e-12
325.76	1.3560	1.4722	3.303e-12	4.479e-12	4.863e-12
328.79	1.3495	1.4611	5.585e-12	7.537e-12	8.160e-12
331.82	1.3432	1.4504	9.353e-12	1.256e-11	1.357e-11
334.85	1.3370	1.4401	1.552e-11	2.075e-11	2.235e-11
337.88	1.3310	1.4302	2.553e-11	3.397e-11	3.651e-11
340.91	1.3251	1.4206	4.161e-11	5.514e-11	5.911e-11
343.94	1.3194	1.4114	6.726e-11	8.874e-11	9.493e-11
346.97	1.3138	1.4025	1.078e-10	1.416e-10	1.512e-10
350.00	1.3084	1.3938	1.714e-10	2.243e-10	2.389e-10
353.03	1.3032	1.3855	2.704e-10	3.524e-10	3.747e-10
356.06	1.2980	1.3775	4.234e-10	5.495e-10	5.832e-10
359.09	1.2930	1.3697	6.578e-10	8.505e-10	9.010e-10
362.12	1.2881	1.3622	1.015e-09	1.307e-09	1.382e-09
365.15	1.2834	1.3549	1.554e-09	1.994e-09	2.105e-09
368.18	1.2787	1.3479	2.363e-09	3.022e-09	3.186e-09
371.21	1.2742	1.3410	3.570e-09	4.549e-09	4.788e-09
374.24	1.2698	1.3344	5.358e-09	6.803e-09	7.150e-09
377.27	1.2655	1.3280	7.989e-09	1.011e-08	1.061e-08
380.30	1.2612	1.3218	1.184e-08	1.493e-08	1.565e-08
383.33	1.2571	1.3157	1.743e-08	2.191e-08	2.293e-08
386.36	1.2531	1.3099	2.551e-08	3.197e-08	3.342e-08
389.39	1.2492	1.3042	3.713e-08	4.638e-08	4.842e-08
392.42	1.2453	1.2987	5.372e-08	6.690e-08	6.976e-08
395.45	1.2416	1.2933	7.729e-08	9.596e-08	9.996e-08
398.48	1.2379	1.2881	1.106e-07	1.369e-07	1.425e-07
401.52	1.2344	1.2830	1.574e-07	1.943e-07	2.020e-07
404.55	1.2309	1.2781	2.229e-07	2.743e-07	2.848e-07
407.58	1.2274	1.2733	3.139e-07	3.853e-07	3.997e-07
410.61	1.2241	1.2686	4.400e-07	5.386e-07	5.582e-07
413.64	1.2208	1.2641	6.137e-07	7.493e-07	7.758e-07
416.67	1.2176	1.2596	8.519e-07	1.037e-06	1.073e-06
419.70	1.2145	1.2553	1.177e-06	1.430e-06	1.478e-06
422.73	1.2114	1.2511	1.619e-06	1.961e-06	2.025e-06
425.76	1.2084	1.2471	2.216e-06	2.678e-06	2.764e-06
428.79	1.2055	1.2431	3.021e-06	3.642e-06	3.756e-06
431.82	1.2026	1.2392	4.101e-06	4.932e-06	5.082e-06
434.85	1.1998	1.2354	5.543e-06	6.650e-06	6.847e-06

437.88	1.1971	1.2317	7.461e-06	8.931e-06	9.189e-06
440.91	1.1944	1.2281	1.000e-05	1.195e-05	1.228e-05
443.94	1.1917	1.2246	1.336e-05	1.592e-05	1.636e-05
446.97	1.1891	1.2212	1.777e-05	2.113e-05	2.169e-05
450.00	1.1866	1.2178	2.354e-05	2.793e-05	2.867e-05

Pathway: EQ23-TS65-DC
Reactant Energy: 93.09 kcal/mol
TS Energy: 94.85 kcal/mol
Product Energy: None kcal/mol
Barrier Height: 1.76 kcal/mol
Imaginary Frequency: 404.27 cm^-1

Temperature(K)	Wigner_Factor	Eckart_Factor	TST Rate(s^{-1})	Wigner Rate (s^{-1})	Eckart Rate (s^{-1})
150.00	1.6265	1.9765	8.524e+09	1.386e+10	1.685e+10
153.03	1.6020	1.9281	9.775e+09	1.566e+10	1.885e+10
156.06	1.5788	1.8836	1.115e+10	1.761e+10	2.101e+10
159.09	1.5570	1.8426	1.267e+10	1.972e+10	2.334e+10
162.12	1.5363	1.8046	1.433e+10	2.201e+10	2.585e+10
165.15	1.5168	1.7693	1.613e+10	2.447e+10	2.854e+10
168.18	1.4984	1.7366	1.809e+10	2.711e+10	3.142e+10
171.21	1.4809	1.7061	2.022e+10	2.994e+10	3.450e+10
174.24	1.4643	1.6777	2.252e+10	3.297e+10	3.777e+10
177.27	1.4486	1.6511	2.499e+10	3.619e+10	4.126e+10
180.30	1.4336	1.6263	2.764e+10	3.962e+10	4.495e+10
183.33	1.4194	1.6029	3.048e+10	4.327e+10	4.886e+10
186.36	1.4059	1.5810	3.352e+10	4.712e+10	5.299e+10
189.39	1.3930	1.5604	3.675e+10	5.120e+10	5.735e+10
192.42	1.3807	1.5409	4.019e+10	5.550e+10	6.194e+10
195.45	1.3690	1.5226	4.385e+10	6.003e+10	6.676e+10
198.48	1.3578	1.5053	4.772e+10	6.479e+10	7.183e+10
201.52	1.3471	1.4889	5.181e+10	6.979e+10	7.714e+10
204.55	1.3369	1.4734	5.612e+10	7.503e+10	8.269e+10
207.58	1.3272	1.4587	6.067e+10	8.052e+10	8.850e+10
210.61	1.3178	1.4447	6.546e+10	8.626e+10	9.456e+10
213.64	1.3089	1.4314	7.048e+10	9.225e+10	1.009e+11
216.67	1.3003	1.4188	7.575e+10	9.849e+10	1.075e+11
219.70	1.2921	1.4068	8.126e+10	1.050e+11	1.143e+11
222.73	1.2842	1.3953	8.703e+10	1.118e+11	1.214e+11
225.76	1.2766	1.3844	9.305e+10	1.188e+11	1.288e+11
228.79	1.2693	1.3740	9.932e+10	1.261e+11	1.365e+11
231.82	1.2623	1.3640	1.059e+11	1.336e+11	1.444e+11
234.85	1.2556	1.3545	1.127e+11	1.415e+11	1.526e+11
237.88	1.2491	1.3453	1.197e+11	1.496e+11	1.611e+11
240.91	1.2429	1.3366	1.271e+11	1.579e+11	1.698e+11
243.94	1.2369	1.3282	1.347e+11	1.666e+11	1.789e+11
246.97	1.2311	1.3202	1.426e+11	1.755e+11	1.882e+11
250.00	1.2255	1.3125	1.507e+11	1.847e+11	1.978e+11

253.03	1.2202	1.3051	1.592e+11	1.942e+11	2.077e+11
256.06	1.2150	1.2980	1.679e+11	2.040e+11	2.179e+11
259.09	1.2100	1.2911	1.769e+11	2.140e+11	2.284e+11
262.12	1.2052	1.2845	1.862e+11	2.244e+11	2.391e+11
265.15	1.2005	1.2782	1.957e+11	2.350e+11	2.502e+11
268.18	1.1960	1.2721	2.056e+11	2.459e+11	2.615e+11
271.21	1.1916	1.2662	2.157e+11	2.571e+11	2.732e+11
274.24	1.1874	1.2605	2.262e+11	2.685e+11	2.851e+11
277.27	1.1834	1.2551	2.369e+11	2.803e+11	2.973e+11
280.30	1.1794	1.2498	2.479e+11	2.923e+11	3.098e+11
283.33	1.1756	1.2447	2.592e+11	3.047e+11	3.226e+11
286.36	1.1719	1.2397	2.707e+11	3.173e+11	3.356e+11
289.39	1.1683	1.2350	2.826e+11	3.302e+11	3.490e+11
292.42	1.1649	1.2304	2.948e+11	3.434e+11	3.627e+11
295.45	1.1615	1.2259	3.072e+11	3.568e+11	3.766e+11
298.48	1.1582	1.2216	3.200e+11	3.706e+11	3.909e+11
301.52	1.1551	1.2174	3.330e+11	3.846e+11	4.054e+11
304.55	1.1520	1.2133	3.463e+11	3.990e+11	4.202e+11
307.58	1.1490	1.2094	3.599e+11	4.136e+11	4.353e+11
310.61	1.1461	1.2056	3.738e+11	4.285e+11	4.507e+11
313.64	1.1433	1.2019	3.880e+11	4.436e+11	4.664e+11
316.67	1.1406	1.1983	4.025e+11	4.591e+11	4.823e+11
319.70	1.1379	1.1949	4.173e+11	4.748e+11	4.986e+11
322.73	1.1353	1.1915	4.323e+11	4.909e+11	5.151e+11
325.76	1.1328	1.1882	4.477e+11	5.071e+11	5.319e+11
328.79	1.1304	1.1850	4.633e+11	5.237e+11	5.490e+11
331.82	1.1280	1.1819	4.792e+11	5.406e+11	5.664e+11
334.85	1.1257	1.1789	4.954e+11	5.577e+11	5.841e+11
337.88	1.1235	1.1760	5.119e+11	5.751e+11	6.020e+11
340.91	1.1213	1.1731	5.287e+11	5.928e+11	6.202e+11
343.94	1.1192	1.1704	5.457e+11	6.108e+11	6.387e+11
346.97	1.1171	1.1677	5.631e+11	6.290e+11	6.575e+11
350.00	1.1151	1.1651	5.807e+11	6.475e+11	6.765e+11
353.03	1.1131	1.1625	5.986e+11	6.663e+11	6.958e+11
356.06	1.1112	1.1600	6.167e+11	6.853e+11	7.154e+11
359.09	1.1093	1.1576	6.352e+11	7.046e+11	7.352e+11
362.12	1.1075	1.1552	6.539e+11	7.242e+11	7.554e+11
365.15	1.1057	1.1529	6.729e+11	7.440e+11	7.758e+11
368.18	1.1040	1.1507	6.921e+11	7.641e+11	7.964e+11
371.21	1.1023	1.1485	7.117e+11	7.845e+11	8.173e+11
374.24	1.1006	1.1463	7.315e+11	8.051e+11	8.385e+11
377.27	1.0990	1.1442	7.515e+11	8.260e+11	8.600e+11
380.30	1.0975	1.1422	7.719e+11	8.471e+11	8.817e+11
383.33	1.0959	1.1402	7.925e+11	8.685e+11	9.036e+11
386.36	1.0944	1.1383	8.134e+11	8.902e+11	9.258e+11
389.39	1.0930	1.1364	8.345e+11	9.121e+11	9.483e+11
392.42	1.0915	1.1345	8.559e+11	9.342e+11	9.710e+11
395.45	1.0901	1.1327	8.775e+11	9.567e+11	9.940e+11

398.48	1.0888	1.1309	8.995e+11	9.793e+11	1.017e+12
401.52	1.0874	1.1292	9.216e+11	1.002e+12	1.041e+12
404.55	1.0861	1.1275	9.441e+11	1.025e+12	1.064e+12
407.58	1.0849	1.1259	9.667e+11	1.049e+12	1.088e+12
410.61	1.0836	1.1242	9.897e+11	1.072e+12	1.113e+12
413.64	1.0824	1.1227	1.013e+12	1.096e+12	1.137e+12
416.67	1.0812	1.1211	1.036e+12	1.120e+12	1.162e+12
419.70	1.0800	1.1196	1.060e+12	1.145e+12	1.187e+12
422.73	1.0789	1.1181	1.084e+12	1.169e+12	1.212e+12
425.76	1.0778	1.1166	1.108e+12	1.194e+12	1.237e+12
428.79	1.0767	1.1152	1.132e+12	1.219e+12	1.263e+12
431.82	1.0756	1.1138	1.157e+12	1.245e+12	1.289e+12
434.85	1.0745	1.1125	1.182e+12	1.270e+12	1.315e+12
437.88	1.0735	1.1111	1.207e+12	1.296e+12	1.341e+12
440.91	1.0725	1.1098	1.233e+12	1.322e+12	1.368e+12
443.94	1.0715	1.1085	1.258e+12	1.348e+12	1.395e+12
446.97	1.0706	1.1073	1.284e+12	1.375e+12	1.422e+12
450.00	1.0696	1.1060	1.310e+12	1.401e+12	1.449e+12

Pathway: EQ23-TS90-DC
Reactant Energy: 93.09 kcal/mol
TS Energy: 166.52 kcal/mol
Product Energy: None kcal/mol
Barrier Height: 73.43 kcal/mol
Imaginary Frequency: 604.55 cm^-1

Temperature(K)	Wigner_Factor	Eckart_Factor	TST Rate(s^{-1})	Wigner Rate (s^{-1})	Eckart Rate (s^{-1})
150.00	2.4011	9.9165	3.233e-95	7.763e-95	3.206e-94
153.03	2.3461	8.3578	4.333e-93	1.017e-92	3.622e-92
156.06	2.2944	7.2159	4.804e-91	1.102e-90	3.466e-90
159.09	2.2455	6.3497	4.453e-89	9.999e-89	2.827e-88
162.12	2.1994	5.6738	3.486e-87	7.666e-87	1.978e-86
165.15	2.1558	5.1337	2.326e-85	5.014e-85	1.194e-84
168.18	2.1145	4.6936	1.334e-83	2.822e-83	6.263e-83
171.21	2.0754	4.3291	6.636e-82	1.377e-81	2.873e-81
174.24	2.0383	4.0228	2.881e-80	5.873e-80	1.159e-79
177.27	2.0031	3.7622	1.100e-78	2.204e-78	4.139e-78
180.30	1.9697	3.5382	3.718e-77	7.322e-77	1.315e-76
183.33	1.9379	3.3438	1.119e-75	2.168e-75	3.740e-75
186.36	1.9076	3.1737	3.014e-74	5.749e-74	9.564e-74
189.39	1.8788	3.0237	7.309e-73	1.373e-72	2.210e-72
192.42	1.8514	2.8907	1.604e-71	2.969e-71	4.636e-71
195.45	1.8252	2.7719	3.198e-70	5.837e-70	8.865e-70
198.48	1.8002	2.6653	5.822e-69	1.048e-68	1.552e-68
201.52	1.7763	2.5692	9.716e-68	1.726e-67	2.496e-67
204.55	1.7535	2.4822	1.492e-66	2.616e-66	3.703e-66
207.58	1.7316	2.4030	2.116e-65	3.664e-65	5.084e-65
210.61	1.7107	2.3307	2.781e-64	4.757e-64	6.481e-64

213.64	1.6907	2.2645	3.398e-63	5.744e-63	7.694e-63
216.67	1.6715	2.2036	3.872e-62	6.471e-62	8.531e-62
219.70	1.6531	2.1475	4.126e-61	6.821e-61	8.861e-61
222.73	1.6355	2.0957	4.124e-60	6.744e-60	8.642e-60
225.76	1.6185	2.0476	3.875e-59	6.272e-59	7.935e-59
228.79	1.6022	2.0030	3.432e-58	5.499e-58	6.875e-58
231.82	1.5866	1.9614	2.872e-57	4.557e-57	5.633e-57
234.85	1.5716	1.9227	2.275e-56	3.576e-56	4.375e-56
237.88	1.5571	1.8864	1.710e-55	2.663e-55	3.227e-55
240.91	1.5432	1.8525	1.222e-54	1.886e-54	2.264e-54
243.94	1.5298	1.8206	8.320e-54	1.273e-53	1.515e-53
246.97	1.5168	1.7907	5.403e-53	8.196e-53	9.676e-53
250.00	1.5044	1.7625	3.354e-52	5.046e-52	5.912e-52
253.03	1.4924	1.7360	1.993e-51	2.975e-51	3.460e-51
256.06	1.4808	1.7109	1.136e-50	1.682e-50	1.943e-50
259.09	1.4696	1.6872	6.215e-50	9.133e-50	1.049e-49
262.12	1.4588	1.6648	3.270e-49	4.770e-49	5.444e-49
265.15	1.4484	1.6435	1.657e-48	2.399e-48	2.723e-48
268.18	1.4383	1.6233	8.091e-48	1.164e-47	1.313e-47
271.21	1.4286	1.6041	3.815e-47	5.450e-47	6.120e-47
274.24	1.4192	1.5859	1.738e-46	2.467e-46	2.757e-46
277.27	1.4100	1.5686	7.664e-46	1.081e-45	1.202e-45
280.30	1.4012	1.5520	3.272e-45	4.585e-45	5.079e-45
283.33	1.3927	1.5363	1.355e-44	1.887e-44	2.081e-44
286.36	1.3844	1.5212	5.443e-44	7.535e-44	8.280e-44
289.39	1.3764	1.5069	2.124e-43	2.924e-43	3.201e-43
292.42	1.3686	1.4931	8.061e-43	1.103e-42	1.204e-42
295.45	1.3611	1.4800	2.977e-42	4.051e-42	4.405e-42
298.48	1.3538	1.4674	1.070e-41	1.449e-41	1.571e-41
301.52	1.3468	1.4553	3.752e-41	5.053e-41	5.461e-41
304.55	1.3399	1.4437	1.283e-40	1.719e-40	1.852e-40
307.58	1.3332	1.4326	4.282e-40	5.709e-40	6.135e-40
310.61	1.3268	1.4219	1.396e-39	1.853e-39	1.985e-39
313.64	1.3205	1.4117	4.450e-39	5.876e-39	6.282e-39
316.67	1.3144	1.4018	1.387e-38	1.823e-38	1.945e-38
319.70	1.3084	1.3923	4.233e-38	5.539e-38	5.894e-38
322.73	1.3027	1.3832	1.265e-37	1.648e-37	1.750e-37
325.76	1.2971	1.3744	3.704e-37	4.805e-37	5.091e-37
328.79	1.2916	1.3659	1.064e-36	1.374e-36	1.453e-36
331.82	1.2863	1.3577	2.996e-36	3.854e-36	4.067e-36
334.85	1.2812	1.3498	8.282e-36	1.061e-35	1.118e-35
337.88	1.2761	1.3422	2.248e-35	2.869e-35	3.018e-35
340.91	1.2712	1.3348	5.997e-35	7.624e-35	8.005e-35
343.94	1.2665	1.3277	1.572e-34	1.991e-34	2.088e-34
346.97	1.2619	1.3208	4.054e-34	5.115e-34	5.354e-34
350.00	1.2573	1.3142	1.028e-33	1.293e-33	1.351e-33
353.03	1.2529	1.3077	2.567e-33	3.216e-33	3.357e-33
356.06	1.2487	1.3015	6.309e-33	7.878e-33	8.211e-33

359.09	1.2445	1.2955	1.527e-32	1.901e-32	1.979e-32
362.12	1.2404	1.2896	3.644e-32	4.520e-32	4.700e-32
365.15	1.2364	1.2840	8.570e-32	1.060e-31	1.100e-31
368.18	1.2325	1.2785	1.987e-31	2.450e-31	2.541e-31
371.21	1.2288	1.2732	4.546e-31	5.586e-31	5.788e-31
374.24	1.2251	1.2680	1.026e-30	1.257e-30	1.301e-30
377.27	1.2215	1.2630	2.286e-30	2.793e-30	2.888e-30
380.30	1.2180	1.2581	5.030e-30	6.126e-30	6.328e-30
383.33	1.2145	1.2534	1.093e-29	1.327e-29	1.370e-29
386.36	1.2112	1.2488	2.346e-29	2.842e-29	2.930e-29
389.39	1.2079	1.2443	4.977e-29	6.012e-29	6.193e-29
392.42	1.2047	1.2400	1.044e-28	1.257e-28	1.294e-28
395.45	1.2016	1.2358	2.164e-28	2.600e-28	2.674e-28
398.48	1.1985	1.2317	4.438e-28	5.319e-28	5.467e-28
401.52	1.1955	1.2277	9.004e-28	1.076e-27	1.105e-27
404.55	1.1926	1.2238	1.808e-27	2.156e-27	2.212e-27
407.58	1.1898	1.2200	3.591e-27	4.273e-27	4.382e-27
410.61	1.1870	1.2163	7.064e-27	8.385e-27	8.593e-27
413.64	1.1842	1.2128	1.376e-26	1.629e-26	1.669e-26
416.67	1.1816	1.2093	2.654e-26	3.136e-26	3.209e-26
419.70	1.1790	1.2059	5.072e-26	5.979e-26	6.116e-26
422.73	1.1764	1.2025	9.602e-26	1.130e-25	1.155e-25
425.76	1.1739	1.1993	1.802e-25	2.115e-25	2.161e-25
428.79	1.1715	1.1961	3.351e-25	3.925e-25	4.008e-25
431.82	1.1691	1.1931	6.178e-25	7.222e-25	7.370e-25
434.85	1.1667	1.1901	1.129e-24	1.318e-24	1.344e-24
437.88	1.1644	1.1871	2.048e-24	2.384e-24	2.431e-24
440.91	1.1622	1.1843	3.682e-24	4.279e-24	4.361e-24
443.94	1.1600	1.1815	6.570e-24	7.620e-24	7.762e-24
446.97	1.1578	1.1787	1.163e-23	1.346e-23	1.371e-23
450.00	1.1557	1.1761	2.043e-23	2.361e-23	2.403e-23

Pathway: EQ18-TS96-DC
Reactant Energy: 117.86 kcal/mol
TS Energy: 141.88 kcal/mol
Product Energy: None kcal/mol
Barrier Height: 24.02 kcal/mol
Imaginary Frequency: 452.57 cm^-1

Temperature(K)	Wigner_Factor	Eckart_Factor	TST Rate(s^{-1})	Wigner Rate (s^{-1})	Eckart Rate (s^{-1})
150.00	1.7852	2.5757	3.151e-23	5.626e-23	8.117e-23
153.03	1.7544	2.4621	1.586e-22	2.782e-22	3.904e-22
156.06	1.7254	2.3616	7.495e-22	1.293e-21	1.770e-21
159.09	1.6980	2.2723	3.341e-21	5.673e-21	7.591e-21
162.12	1.6722	2.1923	1.409e-20	2.356e-20	3.088e-20
165.15	1.6477	2.1204	5.636e-20	9.287e-20	1.195e-19
168.18	1.6246	2.0554	2.146e-19	3.486e-19	4.411e-19
171.21	1.6027	1.9965	7.794e-19	1.249e-18	1.556e-18

174.24	1.5819	1.9429	2.708e-18	4.284e-18	5.261e-18
177.27	1.5622	1.8938	9.018e-18	1.409e-17	1.708e-17
180.30	1.5434	1.8489	2.885e-17	4.453e-17	5.334e-17
183.33	1.5256	1.8075	8.885e-17	1.355e-16	1.606e-16
186.36	1.5087	1.7694	2.638e-16	3.980e-16	4.668e-16
189.39	1.4925	1.7342	7.569e-16	1.130e-15	1.313e-15
192.42	1.4771	1.7015	2.101e-15	3.103e-15	3.575e-15
195.45	1.4624	1.6711	5.651e-15	8.265e-15	9.444e-15
198.48	1.4484	1.6428	1.475e-14	2.137e-14	2.424e-14
201.52	1.4350	1.6165	3.743e-14	5.371e-14	6.050e-14
204.55	1.4222	1.5918	9.238e-14	1.314e-13	1.471e-13
207.58	1.4100	1.5687	2.221e-13	3.132e-13	3.485e-13
210.61	1.3983	1.5470	5.209e-13	7.284e-13	8.059e-13
213.64	1.3871	1.5267	1.193e-12	1.654e-12	1.821e-12
216.67	1.3763	1.5075	2.669e-12	3.673e-12	4.023e-12
219.70	1.3660	1.4895	5.842e-12	7.980e-12	8.701e-12
222.73	1.3561	1.4724	1.252e-11	1.698e-11	1.843e-11
225.76	1.3466	1.4563	2.629e-11	3.541e-11	3.829e-11
228.79	1.3375	1.4411	5.415e-11	7.242e-11	7.803e-11
231.82	1.3287	1.4266	1.095e-10	1.454e-10	1.562e-10
234.85	1.3203	1.4129	2.173e-10	2.869e-10	3.070e-10
237.88	1.3122	1.3999	4.240e-10	5.564e-10	5.936e-10
240.91	1.3044	1.3876	8.137e-10	1.061e-09	1.129e-09
243.94	1.2969	1.3758	1.537e-09	1.993e-09	2.114e-09
246.97	1.2896	1.3646	2.857e-09	3.685e-09	3.899e-09
250.00	1.2827	1.3539	5.235e-09	6.715e-09	7.088e-09
253.03	1.2759	1.3437	9.454e-09	1.206e-08	1.270e-08
256.06	1.2694	1.3340	1.684e-08	2.138e-08	2.246e-08
259.09	1.2632	1.3247	2.959e-08	3.738e-08	3.920e-08
262.12	1.2571	1.3158	5.134e-08	6.454e-08	6.756e-08
265.15	1.2513	1.3073	8.797e-08	1.101e-07	1.150e-07
268.18	1.2456	1.2992	1.489e-07	1.855e-07	1.935e-07
271.21	1.2402	1.2914	2.492e-07	3.091e-07	3.218e-07
274.24	1.2349	1.2839	4.124e-07	5.092e-07	5.294e-07
277.27	1.2298	1.2767	6.749e-07	8.300e-07	8.616e-07
280.30	1.2248	1.2698	1.093e-06	1.339e-06	1.388e-06
283.33	1.2201	1.2631	1.752e-06	2.138e-06	2.213e-06
286.36	1.2154	1.2567	2.782e-06	3.381e-06	3.496e-06
289.39	1.2109	1.2506	4.373e-06	5.296e-06	5.469e-06
292.42	1.2066	1.2447	6.813e-06	8.220e-06	8.479e-06
295.45	1.2024	1.2390	1.052e-05	1.265e-05	1.303e-05
298.48	1.1983	1.2335	1.610e-05	1.929e-05	1.985e-05
301.52	1.1943	1.2282	2.443e-05	2.917e-05	3.000e-05
304.55	1.1905	1.2231	3.677e-05	4.377e-05	4.497e-05
307.58	1.1867	1.2181	5.490e-05	6.515e-05	6.687e-05
310.61	1.1831	1.2134	8.134e-05	9.624e-05	9.870e-05
313.64	1.1796	1.2088	1.196e-04	1.411e-04	1.446e-04
316.67	1.1762	1.2043	1.747e-04	2.054e-04	2.103e-04

319.70	1.1729	1.2000	2.532e-04	2.970e-04	3.038e-04
322.73	1.1696	1.1959	3.645e-04	4.264e-04	4.359e-04
325.76	1.1665	1.1918	5.213e-04	6.081e-04	6.213e-04
328.79	1.1634	1.1879	7.407e-04	8.617e-04	8.799e-04
331.82	1.1605	1.1842	1.046e-03	1.214e-03	1.238e-03
334.85	1.1576	1.1805	1.467e-03	1.699e-03	1.732e-03
337.88	1.1547	1.1769	2.047e-03	2.363e-03	2.409e-03
340.91	1.1520	1.1735	2.838e-03	3.270e-03	3.331e-03
343.94	1.1493	1.1702	3.913e-03	4.498e-03	4.579e-03
346.97	1.1467	1.1669	5.366e-03	6.154e-03	6.262e-03
350.00	1.1442	1.1638	7.319e-03	8.374e-03	8.517e-03
353.03	1.1418	1.1607	9.929e-03	1.134e-02	1.153e-02
356.06	1.1393	1.1578	1.340e-02	1.527e-02	1.552e-02
359.09	1.1370	1.1549	1.800e-02	2.047e-02	2.079e-02
362.12	1.1347	1.1521	2.406e-02	2.730e-02	2.772e-02
365.15	1.1325	1.1494	3.200e-02	3.624e-02	3.678e-02
368.18	1.1303	1.1467	4.237e-02	4.790e-02	4.859e-02
371.21	1.1282	1.1442	5.585e-02	6.301e-02	6.390e-02
374.24	1.1261	1.1417	7.330e-02	8.254e-02	8.368e-02
377.27	1.1241	1.1392	9.577e-02	1.077e-01	1.091e-01
380.30	1.1221	1.1369	1.246e-01	1.398e-01	1.417e-01
383.33	1.1202	1.1346	1.615e-01	1.809e-01	1.832e-01
386.36	1.1183	1.1323	2.084e-01	2.331e-01	2.360e-01
389.39	1.1165	1.1301	2.680e-01	2.992e-01	3.029e-01
392.42	1.1147	1.1280	3.432e-01	3.826e-01	3.871e-01
395.45	1.1130	1.1259	4.379e-01	4.874e-01	4.931e-01
398.48	1.1113	1.1239	5.568e-01	6.187e-01	6.257e-01
401.52	1.1096	1.1219	7.053e-01	7.826e-01	7.913e-01
404.55	1.1079	1.1200	8.904e-01	9.865e-01	9.972e-01
407.58	1.1063	1.1181	1.120e+00	1.239e+00	1.253e+00
410.61	1.1048	1.1162	1.405e+00	1.552e+00	1.568e+00
413.64	1.1033	1.1144	1.756e+00	1.937e+00	1.957e+00
416.67	1.1018	1.1127	2.187e+00	2.410e+00	2.434e+00
419.70	1.1003	1.1110	2.717e+00	2.989e+00	3.018e+00
422.73	1.0989	1.1093	3.364e+00	3.696e+00	3.731e+00
425.76	1.0975	1.1077	4.152e+00	4.557e+00	4.599e+00
428.79	1.0961	1.1061	5.111e+00	5.602e+00	5.653e+00
431.82	1.0947	1.1045	6.273e+00	6.867e+00	6.929e+00
434.85	1.0934	1.1030	7.678e+00	8.395e+00	8.469e+00
437.88	1.0921	1.1015	9.371e+00	1.023e+01	1.032e+01
440.91	1.0909	1.1001	1.141e+01	1.244e+01	1.255e+01
443.94	1.0896	1.0986	1.385e+01	1.509e+01	1.522e+01
446.97	1.0884	1.0973	1.677e+01	1.825e+01	1.840e+01
450.00	1.0872	1.0959	2.026e+01	2.202e+01	2.220e+01

Pathway: EQ21-TS123-DC
Reactant Energy: 180.83 kcal/mol
TS Energy: 181.72 kcal/mol

Product Energy: None kcal/mol
Barrier Height: 0.89 kcal/mol
Imaginary Frequency: 592.26 cm^-1

Temperature(K)	Wigner_Factor	Eckart_Factor	TST Rate(s^{-1})	Wigner Rate (s^{-1})	Eckart Rate (s^{-1})
150.00	2.3447	3.1707	1.578e+11	3.701e+11	5.005e+11
153.03	2.2919	3.0688	1.708e+11	3.915e+11	5.243e+11
156.06	2.2423	2.9748	1.844e+11	4.135e+11	5.486e+11
159.09	2.1954	2.8878	1.985e+11	4.359e+11	5.734e+11
162.12	2.1511	2.8072	2.133e+11	4.588e+11	5.987e+11
165.15	2.1093	2.7323	2.285e+11	4.821e+11	6.244e+11
168.18	2.0697	2.6625	2.444e+11	5.058e+11	6.507e+11
171.21	2.0321	2.5974	2.608e+11	5.300e+11	6.774e+11
174.24	1.9965	2.5366	2.778e+11	5.546e+11	7.046e+11
177.27	1.9628	2.4797	2.953e+11	5.796e+11	7.323e+11
180.30	1.9307	2.4263	3.134e+11	6.050e+11	7.603e+11
183.33	1.9002	2.3762	3.320e+11	6.308e+11	7.889e+11
186.36	1.8711	2.3290	3.512e+11	6.571e+11	8.179e+11
189.39	1.8435	2.2846	3.709e+11	6.837e+11	8.473e+11
192.42	1.8171	2.2427	3.911e+11	7.106e+11	8.771e+11
195.45	1.7920	2.2031	4.118e+11	7.380e+11	9.073e+11
198.48	1.7680	2.1656	4.331e+11	7.657e+11	9.380e+11
201.52	1.7450	2.1301	4.549e+11	7.938e+11	9.690e+11
204.55	1.7231	2.0965	4.772e+11	8.223e+11	1.000e+12
207.58	1.7022	2.0646	5.000e+11	8.511e+11	1.032e+12
210.61	1.6821	2.0343	5.233e+11	8.802e+11	1.065e+12
213.64	1.6629	2.0054	5.471e+11	9.097e+11	1.097e+12
216.67	1.6445	1.9779	5.713e+11	9.396e+11	1.130e+12
219.70	1.6268	1.9517	5.961e+11	9.697e+11	1.163e+12
222.73	1.6099	1.9268	6.213e+11	1.000e+12	1.197e+12
225.76	1.5936	1.9029	6.470e+11	1.031e+12	1.231e+12
228.79	1.5780	1.8801	6.731e+11	1.062e+12	1.266e+12
231.82	1.5630	1.8583	6.997e+11	1.094e+12	1.300e+12
234.85	1.5486	1.8375	7.268e+11	1.125e+12	1.335e+12
237.88	1.5347	1.8175	7.543e+11	1.158e+12	1.371e+12
240.91	1.5213	1.7983	7.822e+11	1.190e+12	1.407e+12
243.94	1.5084	1.7799	8.105e+11	1.223e+12	1.443e+12
246.97	1.4960	1.7623	8.393e+11	1.256e+12	1.479e+12
250.00	1.4841	1.7453	8.685e+11	1.289e+12	1.516e+12
253.03	1.4726	1.7290	8.980e+11	1.322e+12	1.553e+12
256.06	1.4614	1.7133	9.280e+11	1.356e+12	1.590e+12
259.09	1.4507	1.6982	9.584e+11	1.390e+12	1.628e+12
262.12	1.4403	1.6837	9.892e+11	1.425e+12	1.666e+12
265.15	1.4303	1.6697	1.020e+12	1.459e+12	1.704e+12
268.18	1.4207	1.6562	1.052e+12	1.494e+12	1.742e+12
271.21	1.4113	1.6431	1.084e+12	1.530e+12	1.781e+12
274.24	1.4023	1.6306	1.116e+12	1.565e+12	1.820e+12
277.27	1.3935	1.6184	1.149e+12	1.601e+12	1.859e+12

280.30	1.3851	1.6067	1.182e+12	1.637e+12	1.899e+12
283.33	1.3769	1.5953	1.215e+12	1.673e+12	1.939e+12
286.36	1.3689	1.5844	1.249e+12	1.710e+12	1.979e+12
289.39	1.3613	1.5737	1.283e+12	1.746e+12	2.019e+12
292.42	1.3538	1.5635	1.317e+12	1.783e+12	2.060e+12
295.45	1.3466	1.5535	1.352e+12	1.821e+12	2.100e+12
298.48	1.3396	1.5439	1.387e+12	1.858e+12	2.141e+12
301.52	1.3328	1.5345	1.422e+12	1.896e+12	2.183e+12
304.55	1.3262	1.5254	1.458e+12	1.934e+12	2.224e+12
307.58	1.3198	1.5166	1.494e+12	1.972e+12	2.266e+12
310.61	1.3136	1.5081	1.530e+12	2.010e+12	2.308e+12
313.64	1.3076	1.4998	1.567e+12	2.049e+12	2.350e+12
316.67	1.3017	1.4918	1.604e+12	2.088e+12	2.393e+12
319.70	1.2960	1.4840	1.641e+12	2.127e+12	2.435e+12
322.73	1.2905	1.4764	1.679e+12	2.166e+12	2.478e+12
325.76	1.2851	1.4690	1.716e+12	2.206e+12	2.521e+12
328.79	1.2799	1.4618	1.755e+12	2.246e+12	2.565e+12
331.82	1.2748	1.4548	1.793e+12	2.286e+12	2.608e+12
334.85	1.2698	1.4480	1.831e+12	2.326e+12	2.652e+12
337.88	1.2650	1.4414	1.870e+12	2.366e+12	2.696e+12
340.91	1.2603	1.4349	1.909e+12	2.407e+12	2.740e+12
343.94	1.2558	1.4287	1.949e+12	2.447e+12	2.784e+12
346.97	1.2513	1.4225	1.989e+12	2.488e+12	2.829e+12
350.00	1.2470	1.4166	2.028e+12	2.529e+12	2.873e+12
353.03	1.2428	1.4108	2.069e+12	2.571e+12	2.918e+12
356.06	1.2386	1.4051	2.109e+12	2.612e+12	2.963e+12
359.09	1.2346	1.3996	2.150e+12	2.654e+12	3.009e+12
362.12	1.2307	1.3942	2.191e+12	2.696e+12	3.054e+12
365.15	1.2269	1.3889	2.232e+12	2.738e+12	3.100e+12
368.18	1.2232	1.3838	2.273e+12	2.780e+12	3.145e+12
371.21	1.2196	1.3787	2.315e+12	2.823e+12	3.191e+12
374.24	1.2160	1.3738	2.356e+12	2.865e+12	3.237e+12
377.27	1.2126	1.3691	2.398e+12	2.908e+12	3.284e+12
380.30	1.2092	1.3644	2.441e+12	2.951e+12	3.330e+12
383.33	1.2059	1.3598	2.483e+12	2.994e+12	3.377e+12
386.36	1.2027	1.3553	2.526e+12	3.038e+12	3.423e+12
389.39	1.1995	1.3510	2.569e+12	3.081e+12	3.470e+12
392.42	1.1965	1.3467	2.612e+12	3.125e+12	3.517e+12
395.45	1.1935	1.3425	2.655e+12	3.169e+12	3.564e+12
398.48	1.1905	1.3384	2.699e+12	3.213e+12	3.612e+12
401.52	1.1877	1.3344	2.742e+12	3.257e+12	3.659e+12
404.55	1.1849	1.3305	2.786e+12	3.301e+12	3.707e+12
407.58	1.1821	1.3267	2.830e+12	3.346e+12	3.755e+12
410.61	1.1795	1.3229	2.874e+12	3.390e+12	3.803e+12
413.64	1.1768	1.3192	2.919e+12	3.435e+12	3.851e+12
416.67	1.1743	1.3156	2.963e+12	3.480e+12	3.899e+12
419.70	1.1718	1.3121	3.008e+12	3.525e+12	3.947e+12
422.73	1.1693	1.3087	3.053e+12	3.570e+12	3.996e+12

425.76	1.1669	1.3053	3.098e+12	3.616e+12	4.044e+12
428.79	1.1646	1.3020	3.144e+12	3.661e+12	4.093e+12
431.82	1.1623	1.2987	3.189e+12	3.707e+12	4.142e+12
434.85	1.1600	1.2955	3.235e+12	3.753e+12	4.191e+12
437.88	1.1578	1.2924	3.281e+12	3.799e+12	4.240e+12
440.91	1.1556	1.2893	3.327e+12	3.845e+12	4.289e+12
443.94	1.1535	1.2863	3.373e+12	3.891e+12	4.339e+12
446.97	1.1514	1.2833	3.419e+12	3.937e+12	4.388e+12
450.00	1.1494	1.2804	3.466e+12	3.984e+12	4.438e+12
